# Supplementary material for: Ultra-inert lanthanide chelates as mass tags for multiplexed bioanalysis
Source: Nat Commun. 2024 Nov 13;15:9836. doi: 10.1038/s41467-024-53867-1 (PMC11561307; doi:10.1038/s41467-024-53867-1)
Supplement: Supplementary file 1 — Supplementary Information [file 41467_2024_53867_MOESM1_ESM.pdf]

Supplementary Information for

Ultra-inert lanthanide chelates as mass tags for multiplexed bioanalysis

Tomáš David,[1] Miroslava Šedinová,[1] Aneta Myšková,[1,2] Jaroslav Kuneš,[1,3] Lenka Maletínská,[1] Radek Pohl,[1] Martin Dračínský,[1] Helena Mertlíková-Kaiserová,[1] Karel Čížek,[1] Blanka Klepetářová,[1] Miroslava Litecká,[4] Antonín Kaňa,[2] David Sýkora,[2] Adam Jaroš,[1] Michal Straka,[1] Miloslav Polasek[1*]

[1] Institute of Organic Chemistry and Biochemistry, Czech Academy of Sciences, Prague, Czech Republic.

[2] University of Chemistry and Technology Prague, Prague, Czech Republic.

[3] Institute of Physiology, Czech Academy of Sciences, Czech Republic.

[4] Institute of Inorganic Chemistry, Czech Academy of Sciences, Husinec-Řež, Czech Republic.

[*] E-mail: **miloslav.polasek@uochb.cas.cz**

Table of content

Supplementary Methods 6
Supplementary Fig. 1. High-inertness lanthanide chelate system 8
Supplementary Fig. 2. ClickZip principle notation 9
Supplementary Fig. 3. Effect of **L**[1] substitution on Ln^{III} ClickZip 10
Supplementary Fig. 4. Stability of ligand **PhL**[1] 11
Supplementary Fig. 5. Effect of absolute concentration on Lu^{III} ClickZip 12
Supplementary Fig. 6. ClickZip on large scale 13
Supplementary Fig. 7. Theoretical calculations for ClickZip with La^{III} and Lu^{III} 14
Supplementary Fig. 8. Theoretical calculations for ClickZip with selected metals 15
Supplementary Fig. 9. Formation of empty cages 16
Supplementary Fig. 10. Solid-state structures of empty cages 17
Supplementary Fig. 11. Direct complexation 18
Supplementary Fig. 12. Inapplicability of direct Ln^{III} complexation with ***1,5-cz*-PhL**[1] 19
Supplementary Fig. 13. Comparison of Ln^{III} ClickZip with Ln^{III} direct complexation 20
Supplementary Fig. 14. Kinetic inertness half-life comparison 21
Supplementary Fig. 15. ClickZip kinetic inertness comparison (0.1 M HCl, 25 °C) 22
Supplementary Fig. 16. ClickZip kinetic inertness comparison (0.1 M HCl, 80 °C) 23
Supplementary Fig. 17. ClickZip kinetic inertness comparison (1.0 M HCl, 80 °C) 24
Supplementary Fig. 18. ClickZip kinetic inertness comparison (6.0 M HCl, 80 °C) 25
Supplementary Fig. 19. Transmetallation 26
Supplementary Fig. 20. Isostructurality of **Ph**{**Ln**} in solid state 27
Supplementary Fig. 21. Isostructurality of **Ph**{**Ln**} in solution 28
Supplementary Fig. 22. ClickZip chirality inversion (Λλλλλ ↔ Δδδδδ) 29
Supplementary Fig. 23. Degradation of **Cl**{**Lu**} when isolated as FA salt 30
Supplementary Fig. 24. Acid hydrolysis of model hexapeptides with ClickZip tags 31
Supplementary Fig. 25. LC-MS (single quadrupole) quantification of **HO_2CPh**{**Ln**} tags 32
Supplementary Fig. 26. Solid-phase extraction of **HO_2CPh**{**Ln**} tags 33
Supplementary Fig. 27. Quantification of **HO_2CPh**{**Ln**} tags using LC-MS/MS 34
Supplementary Fig. 28. LC-MS/MS parameters for quantification of **HO_2CPh**{**Ln**} tags 37
Supplementary Fig. 29. LOD and LOQ for **HO_2CPh**{**Ln**} tags using LC-MS/MS 36
Supplementary Fig. 30. LOD and LOQ for **HO_2CPh**{**Ln**} tags using ICP-MS 37
Supplementary Fig. 31. Speciation analysis in mouse liver by ICP-MS with LC-MS 38

Synthesis of pyridine intermediates bearing alkyne or azide group and their precursors:

Supplementary Fig. 32. Synthesis of pyridine intermediates (overall scheme) 39
Supplementary Fig. 33. Synthesis and characterization of intermediate **1** 40
Supplementary Fig. 34. Synthesis and characterization of intermediate **2** 41
Supplementary Fig. 35. Synthesis and characterization of intermediate **3** 42
Supplementary Fig. 36. Synthesis and characterization of intermediate **4** 43
Supplementary Fig. 37. Synthesis and characterization of intermediate **5** 44
Supplementary Fig. 38. Synthesis and characterization of intermediate **6** 45
Supplementary Fig. 39. Synthesis and characterization of intermediate **7** 46
Supplementary Fig. 40. Synthesis and characterization of intermediate **8** 47

Table of content

Synthesis of pyridine intermediates bearing alkyne or azide group and their precursors (continuation):

Supplementary Fig. 41. Synthesis and characterization of intermediate **9** 48
Supplementary Fig. 42. Synthesis and characterization of intermediate **10** 49
Supplementary Fig. 43. Synthesis and characterization of intermediate **11** 50
Supplementary Fig. 44. Synthesis and characterization of intermediate **12** 51
Supplementary Fig. 45. Synthesis and characterization of intermediate **13** 52
Supplementary Fig. 46. Synthesis and characterization of intermediate **14** 53
Supplementary Fig. 47. Synthesis and characterization of intermediate **15** 54

Synthesis of ligands bearing alkyne and azide group and their precursors:

Supplementary Fig. 48. Synthesis of ligands (overall scheme) 55
Supplementary Fig. 49. Synthesis and characterization of intermediate ***t*-BuDO$_2$A≡Py** 56
Supplementary Fig. 50. Large scale synthesis of intermediate ***t*-BuDO$_2$A≡Py** 57
Supplementary Fig. 51. Synthesis and characterization of ligand **L^1** 58
Supplementary Fig. 52. Synthesis and characterization of ligand **PhL^1** 59
Supplementary Fig. 53. Synthesis and characterization of ligand **HO_2CPhL^1** 60
Supplementary Fig. 54. Synthesis and characterization of ligand **ClL^1** 61
Supplementary Fig. 55. Large scale synthesis of ligand **ClL^1** 62

Synthesis of ClickZips:

Supplementary Fig. 56. Synthesis of ClickZips (overall scheme) 63
Supplementary Fig. 57. Synthesis and characterization of {**Lu**} 64
Supplementary Fig. 58. Synthesis and characterization of **Ph**{**Y**} 65
Supplementary Fig. 59. Synthesis and characterization of **Ph**{**Lu**} 66
Supplementary Fig. 60. Synthesis and characterization of **Ph**{**Yb**} 67
Supplementary Fig. 61. Synthesis and characterization of **Ph**{**Tm**} 68
Supplementary Fig. 62. Synthesis and characterization of **Ph**{**Er**} 69
Supplementary Fig. 63. Synthesis and characterization of **Ph**{**Ho**} 70
Supplementary Fig. 64. Synthesis and characterization of **Ph**{**Dy**} 71
Supplementary Fig. 65. Synthesis and characterization of **Ph**{**Tb**} 72
Supplementary Fig. 66. Synthesis and characterization of **Ph**{**Gd**} 73
Supplementary Fig. 67. Synthesis and characterization of **Ph**{**Eu**} 74
Supplementary Fig. 68. Synthesis and characterization of **Ph**{**Sm**} 75
Supplementary Fig. 69. Synthesis and characterization of **Ph**{**Nd**} 76
Supplementary Fig. 70. Synthesis and characterization of **Ph**{**Pr**} 77
Supplementary Fig. 71. Synthesis and characterization of **Ph**{**Ce**} 78
Supplementary Fig. 72. Synthesis and characterization of **Ph**{**La**} 79
Supplementary Fig. 73. Synthesis and characterization of **HO_2CPh**{**Y**} 80
Supplementary Fig. 74. Synthesis and characterization of **HO_2CPh**{**Lu**} 81
Supplementary Fig. 75. Synthesis and characterization of **HO_2CPh**{**Yb**} 82
Supplementary Fig. 76. Synthesis and characterization of **HO_2CPh**{**Tm**} 83
Supplementary Fig. 77. Synthesis and characterization of **Cl**{**Lu**} 84
Supplementary Fig. 78. Large scale synthesis of **Cl**{**Lu**} 85
Supplementary Fig. 79. Synthesis and characterization of **Cl**{**Tm**} 86

Table of content

Synthesis of empty cages and their complexes:

Supplementary Fig. 80. Empty cages and their direct complexation (scheme) 87
Supplementary Fig. 81. Synthesis and characterization of ***1,5-cz*-PhL**[1] 88
Supplementary Fig. 82. Synthesis and characterization of **Ph{Ca}** 89
Supplementary Fig. 83. Synthesis and characterization of ***1,4-cz*-PhL**[1] 90
Supplementary Fig. 84. Synthesis and characterization of ***1,4-cz*-[Lu(PhL^1)]** 91
Supplementary Fig. 85. Synthesis and characterization of ***1,4-cz*-[Gd(PhL^1)]** 92
Supplementary Fig. 86. Characterization of ***1,5-cz*-ClL**[1] 93

Synthesis and identification of dimers encountered during ClickZip synthesis:

Supplementary Fig. 87. ClickZip dimer by-products (scheme) 94
Supplementary Fig. 88. Characterization of ***1,4-open*-(PhL^1)$_2$** 95
Supplementary Fig. 89. Synthesis and characterization of ***1,4/1,4-cz*-[Lu(PhL^1)]$_2$** 96
Supplementary Fig. 90. Characterization of ***1,4/1,4-cz*-[La(PhL^1)]$_2$** 97
Supplementary Fig. 91. Synthesis and characterization of ***1,4/1,4-cz*-(PhL^1)$_2$** 98
Supplementary Fig. 92. Synthesis and characterization of ***1,4/1,5-cz*-(PhL^1)$_2$** 99
Supplementary Fig. 93. Synthesis and characterization of ***1,5/1,5-cz*-(PhL^1)$_2$** 100

Post ClickZip synthesis:

Supplementary Fig. 94. Post ClickZip synthetic examples (scheme) 101
Supplementary Fig. 95. Synthesis and characterization of **Ph{Lu-d_{10}}** 102
Supplementary Fig. 96. Synthesis and characterization of **Ph{Lu-red}** 103
Supplementary Fig. 97. Synthesis and epimer separation of **BocCys{Lu}** 104
Supplementary Fig. 98. Synthesis and characterization of **N_3{Lu}** 105
Supplementary Fig. 99. Synthesis and characterization of **H_2NTrz{Lu}** 106
Supplementary Fig. 100. Synthesis and characterization of **HO_2CNO_2Ph{Lu}** 107
Supplementary Fig. 101. Synthesis and characterization of **HO_2CBn{Lu}** 108
Supplementary Fig. 102. Synthesis and characterization of **HO_2CBn{Tm}** 109
Supplementary Fig. 103. ClickZip hexapeptide conjugates (scheme) 110
Supplementary Fig. 104. Synthesis and characterization of **{Y}PhCO-AYFHVG-NH_2** 111
Supplementary Fig. 105. Synthesis and characterization of **{Lu}PhCO-AYFHVG-NH_2** 112
Supplementary Fig. 106. Synthesis and characterization of **{Lu}BnCO-AYFHVG-NH_2** 113
Supplementary Fig. 107. Synthesis and characterization of **{Tm}BnCO-AYFHVG-NH_2** 114
Supplementary Fig. 108. Synthesis and characterization of **{Lu}R_6N_3** 115
Supplementary Fig. 109. Synthesis and characterization of **{Tm}R_6N_3** 116
Supplementary Fig. 110. Synthesis and characterization of **{Lu}R_6** 117
Supplementary Fig. 111. Synthesis and characterization of **{Tm}R_6** 118
Supplementary Fig. 112. Anti-obesity peptide ClickZip conjugates (scheme) 119
Supplementary Fig. 113. Synthesis and characterization of **{Lu}PrRP31** 120
Supplementary Fig. 114. Synthesis and characterization of **{Lu}palm^{11}-PrRP31** 121
Supplementary Fig. 115. Synthesis and characterization of **{Yb}palm^{11}-PrRP31** 122
Supplementary Fig. 116. Synthesis and characterization of **{Tm}palm^{11}-PrRP31** 123

Table of content

Synthesis of supporting and reference compounds:

Supplementary Fig. 117. Synthesis of hexaarginine CPP intermediate (overall scheme) 124
Supplementary Fig. 118. Synthesis and characterization of Fmoc-D-R(Pbf)R(NO_2)-OH...................... 125
Supplementary Fig. 119. Synthesis and characterization of H-D-R(Pbf)R(NO_2)-NH_2.......................... 126
Supplementary Fig. 120. Synthesis and characterization of H-[D-R(Pbf)R(NO_2)]$_3$-NH_2...................... 127
Supplementary Fig. 121. Preparation of [**Lu(NO$_2$BnDOTA)**] stock solution.. 128
Supplementary Fig. 122. Preparation of [**Gd(NO$_2$BnDOTA)**] stock solution 129
Supplementary Fig. 123. Preparation of [**La(NO$_2$BnDOTA)**] stock solution.. 130
Supplementary Fig. 124. Synthesis and characterization of **PhL^1-NMM** adduct 131

Solid-state structures from X-ray crystallography:

Supplementary Fig. 125. Solid-state structure of **Ph**{**Y**}.. 132
Supplementary Fig. 126. Solid-state structure of **Ph**{**Yb**} 133
Supplementary Fig. 127. Solid-state structure of **Ph**{**Tm**} 134
Supplementary Fig. 128. Solid-state structure of **Ph**{**Er**} 135
Supplementary Fig. 129. Solid-state structure of **Ph**{**Ho**} 136
Supplementary Fig. 130. Solid-state structure of **Ph**{**Dy**}.. 137
Supplementary Fig. 131. Solid-state structure of **Ph**{**Tb**}.. 138
Supplementary Fig. 132. Solid-state structure of **Ph**{**Gd**} 139
Supplementary Fig. 133. Solid-state structure of **Ph**{**Eu**}.. 140
Supplementary Fig. 134. Solid-state structure of **Ph**{**Sm**} 141
Supplementary Fig. 135. Solid-state structure of **Ph**{**Pr**} 142
Supplementary Fig. 136. Solid-state structure of **Ph**{**Ca**}.. 143
Supplementary Fig. 137. Solid-state structure of **Cl**{**Lu**} 144
Supplementary Fig. 138. Solid-state structure of **HO$_2$CPh**{**Lu**} 145
Supplementary Fig. 139. Solid-state structure of **HO$_2$CBn**{**Tm**}... 146
Supplementary Fig. 140. Selected crystallographic parameters.. 147

References:

Supplementary References 148

Supplementary Methods

General: All chemicals were purchased from *Sigma-Aldrich* and *Fluorochem*, dry solvents from *Acros Organics*, deuterated solvents from *Eurisotop*, nitric acid for mineralization before ICP-MS analysis (65%, high-purity Analpure®) from *Analytika spol. s. r. o*. Throughout the text, H_2O means Milli-Q water (18.2 mΩ·cm) and organic solvents mean HPLC grade or distilled PA grade. Titrated stock solution of HCl (6.53 M) was used for stock solutions of lower concentrations. Stock solutions of $LnCl_3$ (including YCl_3, $ScCl_3$) with concentration determined by ICP-OES were used for 100 mM stock solutions (stabilized by 5 mM HCl). Following abbreviations are used throughtout the text: EIC (extracted ion chromatogram), TEA (Et_3N), FA (HCOOH), AF ($HCOONH_4$), TFA (CF_3COOH), PYAOP ((*7*-azabenzotriazol-*1*-yloxy)tripyrrolidinophosphonium hexafluorophosphate), MOPS (*3*-(*N*-morpholino)propanesulfonic acid), NMM (*N*-Methylmorpholine), ESI (electrospray ionization).

NMR spectroscopy: ^{1}H and ^{13}C NMR spectra were recorded on Bruker Avance III™ HD 400 MHz spectrometer (400.1 MHz for ^{1}H, 100.6 MHz for ^{13}C) equipped with broad-band Prodigy cryo-probe with ATM module (5 mm CPBBO BB-^{1}H/^{19}F/D Z-GRD) or inversion broadband probe with ATM module (5 mm PA BBI ^{1}H/D-BB Z-GRD), Bruker Avance III™ HD 500 MHz spectrometer (500.0 MHz for ^{1}H, 125.7 MHz for ^{13}C) equipped with broad-band cryo-probe with ATM module (5 mm CPBBO BB-^{1}H/^{19}F/D Z-GRD) or Bruker Avance III™ HD 600 MHz spectrometer (600.1 MHz for ^{1}H, 150.9 MHz for ^{13}C) equipped with inverse triple resonance cryo-probe with ATM module (5 mm CPTCI ^{1}H/^{13}C/^{15}N/D Z-GRD). Unless stated otherwise, spectra were recorded at T = 300 K. Chemical shifts are in ppm, coupling constants in Hz. Spectra were referenced using residual ^{1}H solvent signal in ^{1}H NMR (δ(DMSO-d_6) = 2.50 ppm; δ(CD_3CN) = 1.94 ppm) and ^{13}C solvent signal in ^{13}C NMR (δ(DMSO-d_6) = 39.52 ppm; δ(CD_3CN) = 1.32 ppm). NMR spectra measured in D_2O were referenced to the signal of *t*-BuOH as the external standard (1.25 ppm in ^{1}H; 32.43 ppm in ^{13}C). Integrals in showcased NMR spectra were rounded to integers for clarity (obscured signals that cannot be exactly integrated but their presence can be deduced from 2D NMR experiments are coloured grey).

Liquid chromatography: Analytical HPLC experiments were performed on 1260 Infinity II with LC/MSD single quadrupole system (G6125B) from *Agilent* (referred to as LC-MS or HPLC) equipped with Luna® Omega Polar column (5 µm, 100 Å, 150 × 4.6 mm) using H_2O–MeCN gradients (1 mL min^{-1} flow rate) with FA (0.1%) as an additive. Preparative HPLC experiments were performed on 1260 Infinity II (*Agilent*) equipped with YMC-Actus Triart C18 column (5 µm, 100 Å, 250 × 20.0 mm) using H_2O–MeCN gradients (20 mL min^{-1} flow rate) with FA (0.1%) or TFA (0.1%). Normal phase flash chromatography (SiO_2, 40–60 µm, 60 Å) was performed on Combi*Flash*® NEXTGEN 300+ system from *Teledyne ISCO*.

Elemental analysis: CHN elemental analysis was performed on PE 2400 Series II CHN Analyzer from *Perkin Elmer*. Fluorine elemental analysis was performed by combustion of the sample in quartz vessel, followed by adsorption of HF in H_2O and determining its concentration by potentiometry using F^--selective electrode. Lanthanide content was determined by ICP-OES (SPECTRO Arcos from *SPECTRO Analytical Instruments*). All EA data are presented in calcd. (found) format.

Supplementary Methods

High-resolution mass spectra: HRMS (with ESI ionization) were recorded on an *Agilent* 5975C MSD Quadrupol, Q-Tof micro from *Waters* or LTQ Orbitrap XL from *Thermo Fisher Scientific*.

X-Ray diffraction: Single-crystal data of all structures were collected on either XtaLAB Synergy S diffractometer (*Rigaku*) equipped with Mo (Mo/K_α radiation; λ = 0.71073 Å) and Cu (Cu/K_α radiation; λ = 1.54184 Å) micro-focus X-ray source and Hybrid Pixel Array Detector (HyPix-6000HE) or D8 VENTURE diffractometer (*Bruker*) equipped with a Photon 100 CMOS detector, a multilayer monochromator, and a Cu (Cu/K_α radiation; λ = 1.54178 Å) Incoatec micro-focus sealed tube using combined ϕ and ω. The structures were solved either with ShelXT structure solution program[1] using Intrinsic Phasing and refined with the ShelXL refinement package[2] using Least Squares minimisation implemented in Olex2[3] or with CRYSTALS[4] by direct methods with SIR92[5] or by charge-flipping methods using Superflip[6] and refined by full-matrix least-squares on F or F2. Solid-state structures of compounds not displayed in the main text are provided in Supplementary Fig. 125–139. Selected crystallographic parameters for all compounds and additional information are given in Supplementary Fig. 140. Crystallographic data for structural analysis have been deposited with the Cambridge Crystallographic Data Centre (CCDCs are in Supplementary Fig. 140). Copies of this information may be obtained free of charge from http://www.ccdc.cam.ac.uk. Deposited structures are free of any A-alerts (CheckCif). The rationale for the B-alerts are: 2334544 (related to poor quality of the twinned crystal), 2334544, 2334545, 2334550, 2334563, 2334559 (related to the hydrogen atoms of lattice water molecules – due to the low scattering power of hydrogen atoms it is difficult to locate them accurately), 2334547, 2334548 (related to the imperfect absorption correction of crystal containing Lu ion and also to the low quaility of crystals), 2334555 (the higher value of Rint is caused by weak diffractions from the crystal in spite of collecting the data using Cu-radiation and with long exposure times), 2334552 (the residual electron density is caused by the presence of heavy atom, the higher peaks lie in the vicinity of the ytterbium atom and don't make any chemical sense).

Theoretical calculations: Density functional theory (DFT) calculations were performed using the Gaussian 16, C.01 software package. Structures were minimized at the PBE0/def2-TZVP level, i.e. using the Perdew-Burke-Ernzerhof functional in hybrid form that includes 25% of exact-exchange admixture (PBE0)[7] with the def2-TZVP basis set[8], that includes multireference Wood-Boring 46-electron core (MWB46)[9,10] pseudopotential for La atom and Wood-Boring 60-electron core (MWB60)[9] pseudopotential for Lu atom. Frequency calculations were performed to ensure that the optimized structures are true minima. Solvent effects were modelled by conductor-like polarizable continuum model (CPCM)[11]. Grimme's empirical dispersion correction (D3)[12] broader range of applicability, and less empiricism. The main new ingredients are atom-pairwise specific dispersion coefficients and cutoff radii that are both computed from first principles. The coefficients for new eighth-order dispersion terms are computed using established recursion relations. System (geometry was included to model the dispersion interactions. Gibbs energies were obtained as a sum of gas-phase PBE0/def2-TZVP electronic energies, thermal corrections at 298.15 K (calculated in gas-phase mode), and solvation energies using CPCM approximation.

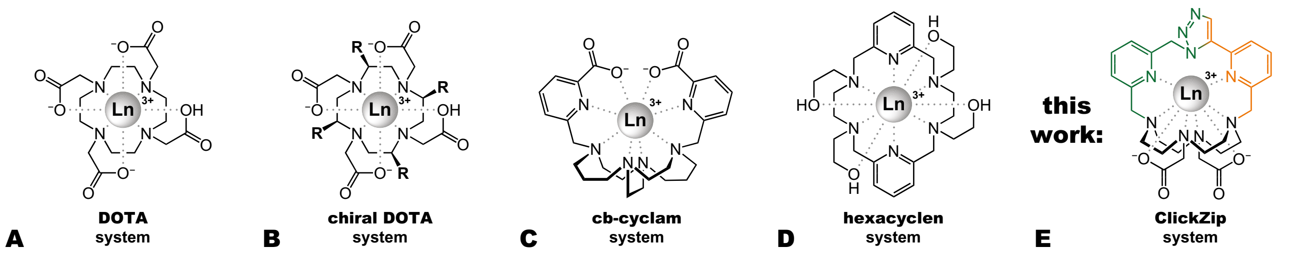

Supplementary Fig. 1. High-inertness lanthanide chelate systems. A. DOTA system, a gold standard for chelation of lanthanides and a benchmark of kinetic inertness compatible with medical *in vivo* use. **B.** Chiral DOTA system, significantly more inert than DOTA but burdened with coordination isomerism[13]. **C.** Cross-bridged cyclam system, significantly more inert than DOTA but burdened with difficult complexation[14]. **D.** 18-membered macrocyclic system[15]. **E.** This work: ClickZip system providing extremely high inertness via an irreversible entrapment mechanism.

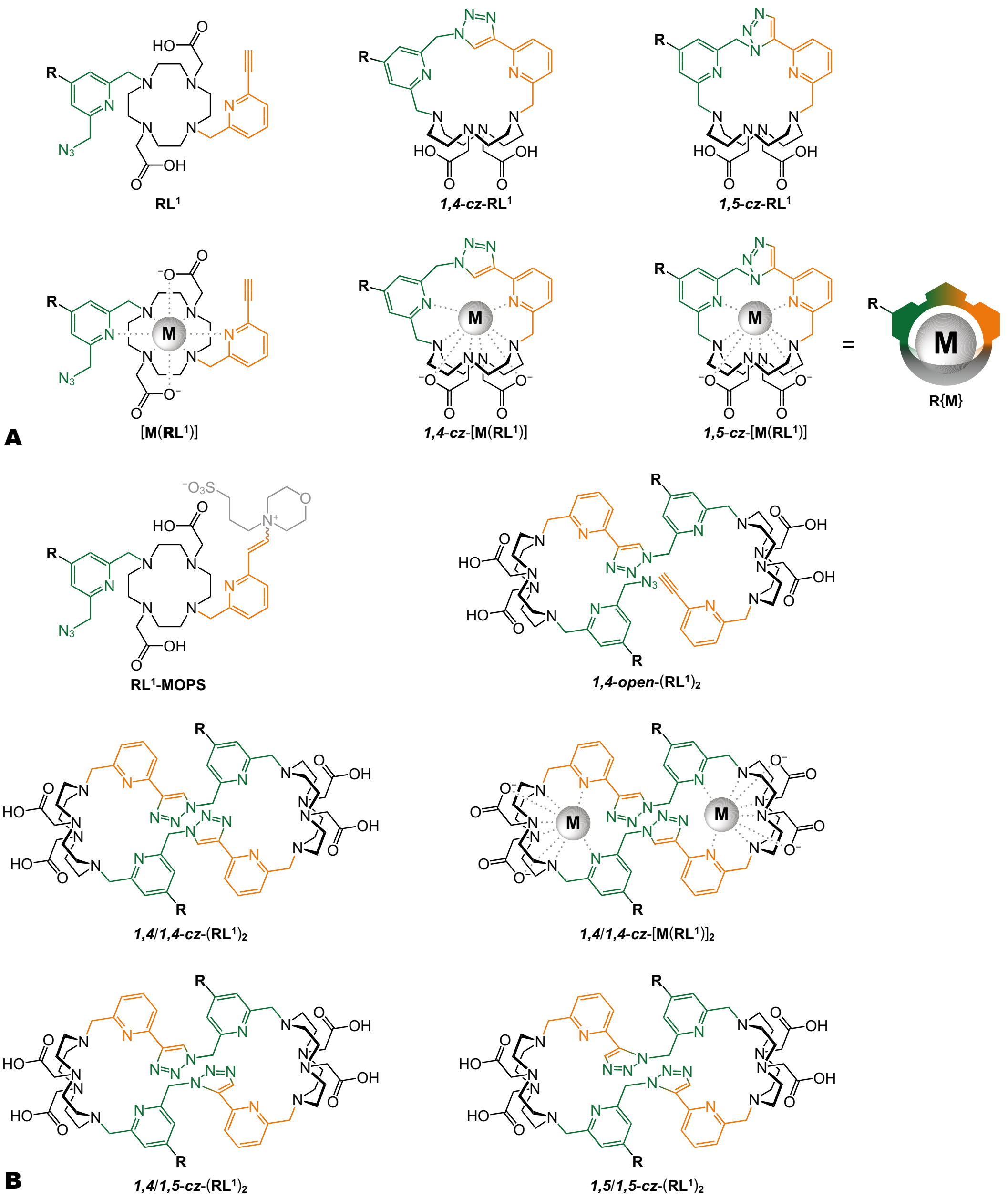

Supplementary Fig. 2. ClickZip principle notation. List of structures and their abbreviations used in the text. **A.** Main species related to the ClickZip synthesis. The metal symbol represents either monovalent (alkali metals), divalent (Ca^{II}) or trivalent (Ln^{III}, Y^{III}, Sc^{III}) cations. In most cases, the formal notation ***1,5-cz*-[M(RL^1)]** is further abbreviated to **R{M}**. **B.** Side products encountered during ClickZip synthesis. In the case of the **RL^1-MOPS** buffer adduct, both cis and trans isomer formation was observed.

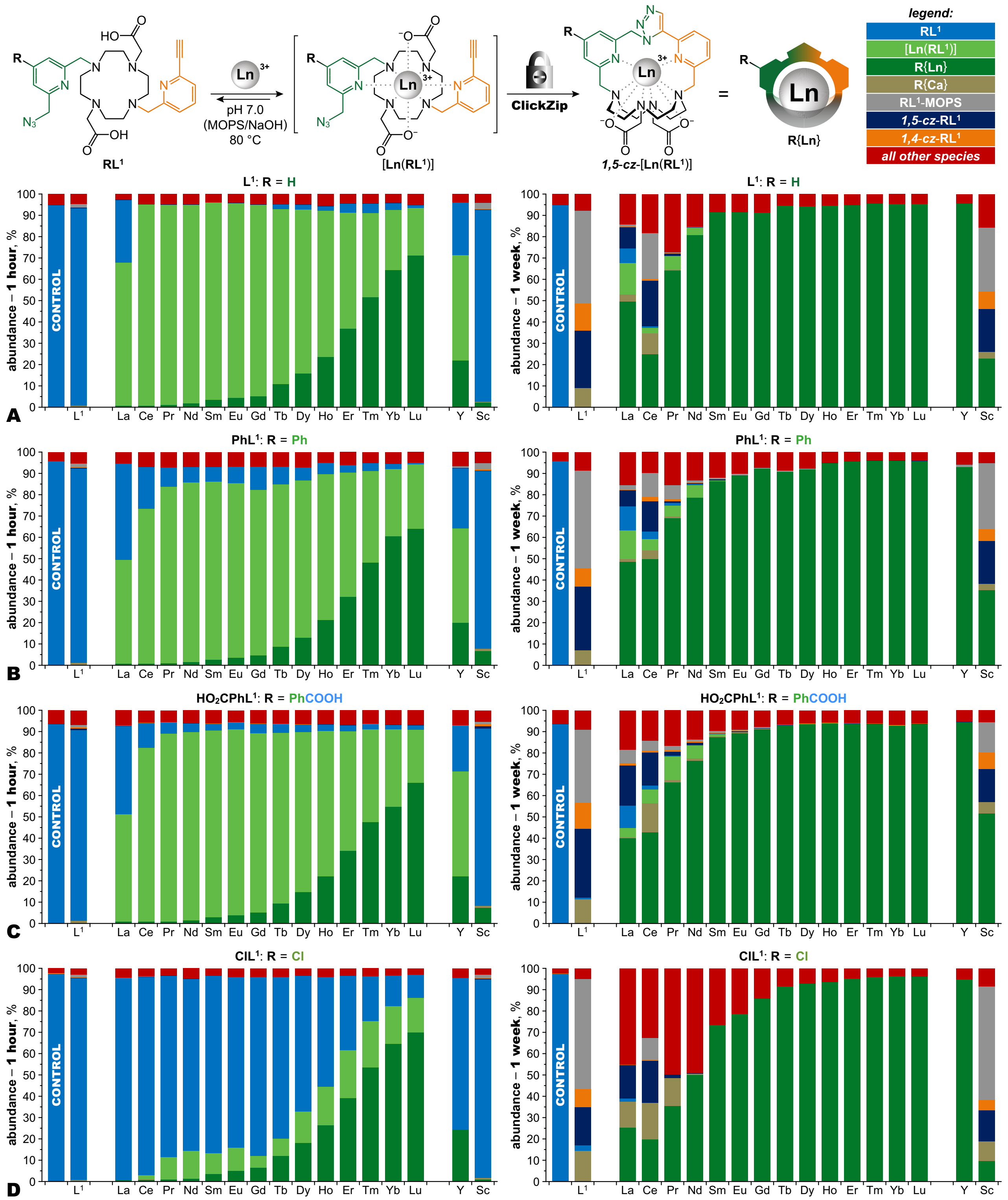

Supplementary Fig. 3. Effect of L^1 substitution on Ln^{III} ClickZip. Comparison of ClickZip reaction efficiencies for ions of the lanthanide series within the **L^1** ligand family (parent **L^1** and three substituted variants) at reaction time 1 h (kinetic point) and 1 week (thermodynamic point). The substitution affects stability of [(**Ln**)**RL^1**] intermediate (it is partially hydrolyzed back to **RL^1** during HPLC analysis), but does not impact the ClickZip chelates, as they are much more inert. ***Conditions:*** 0.5 mM **L^1** derivative and 1.0 mM Ln^{III} salt (including Y^{III} and Sc^{III}) in 50 mM aq. MOPS/NaOH buffer (pH 7.0) at 80 °C (except for column L, where no metal was added). ***Analysis:*** HPLC with UV detection at λ_{max} of the given chelate (H_2O–MeCN gradient with FA additive). Purity of the starting **L^1** derivative just prior to the experiment is labelled CONTROL. Identified species are distinguished by colored bars (all other species are summarized by a red bar). **A. L^1. B. PhL^1. C. HO$_2$CPhL^1. D. ClL^1.** Source data available in Supplementary Data 2.

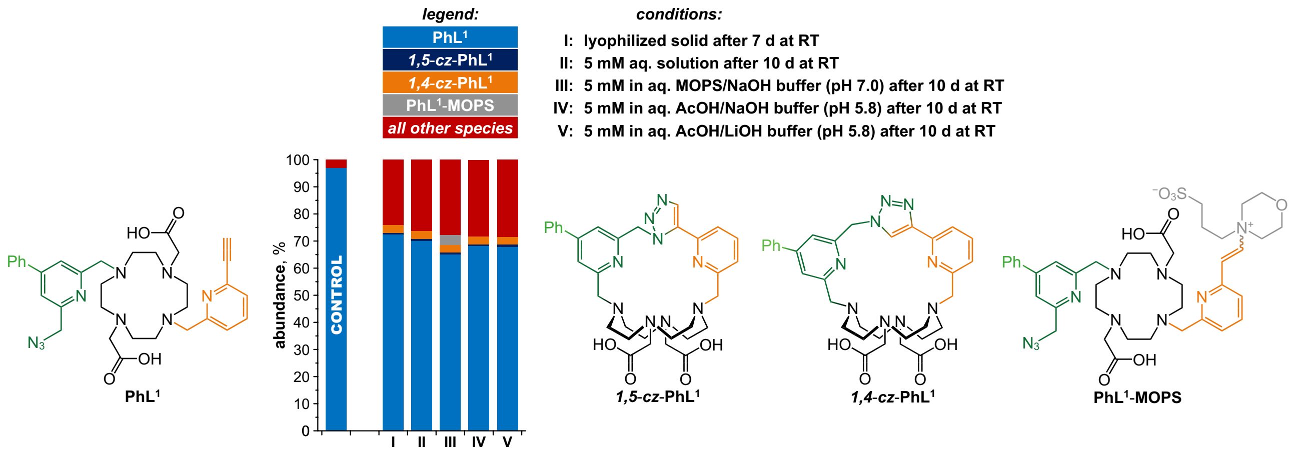

Supplementary Fig. 4. Stability of ClickZip ligands demonstrated on PhL¹. The ability of the ligand to undergo ClickZip reaction with metal ions is counterweighted by its limited stability, which results from the presence of two reactive functional groups in one molecule. Degradation over time due to both intramolecular and intermolecular reactions cannot be completely prevented and storage of the ligand at room temperature (either as a solid or as a dilute aqueous solution) leads to the formation of by-products. Further decomposition occurs in the presence of MOPS buffer by reaction of the terminal acetylene moiety with the tertiary amine group of MOPS, yielding **PhL¹-MOPS** adduct (other amine-contaning molecules are likely to react in a similar manner, for example, **PhL¹-NMM** adduct with NMM; Supplementary Fig. 124). However, the use of MOPS buffer for the ClickZip reaction with metal ions has been shown to be appropriate, as the complexation of the metal ion protects the alkyne moiety and significantly suppresses or even completely prevents (depending on the choice of metal ion) the formation of this undesirable by-product. Frozen dilute aqueous solutions (unbuffered) decomposed much more slowly and the ligand was still the most abundant species after several months of such storage. However, for maximum ClickZip reaction efficiency, it is preferable to use a freshly isolated batch of ligand with metal ion(s). The substituent on the azide-bearing pyridine had no significant effect on stability so the above applies to the entire **L¹** ligand family. ***Analysis:*** HPLC with UV detection at $\lambda = 280$ nm (H_2O–MeCN gradient with FA additive). Purity of **PhL¹** just after isolation is labelled CONTROL. Source data available in Supplementary Data 2.

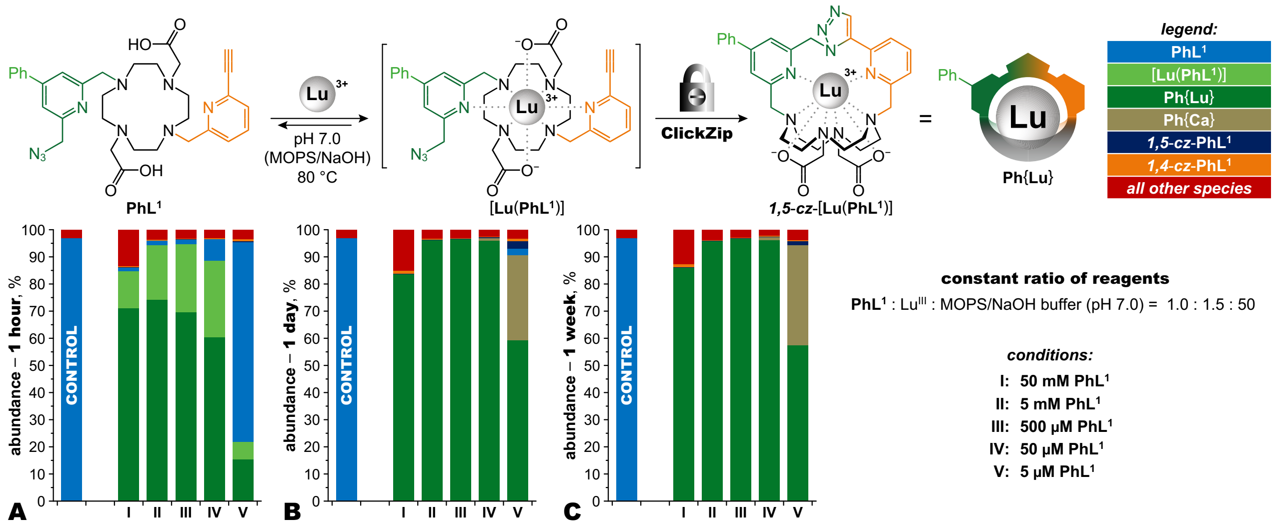

Supplementary Fig. 5. Effect of absolute concentration on Lu^{III} ClickZip. The ClickZip principle consists of two steps – initial complexation of the Ln^{III} ion with the ligand (concentration-dependent reaction) and subsequent intramolecular Huisgen cycloaddition reaction (concentration-independent reaction). Therefore, the reliability of the ClickZip reaction was tested at different concentrations (across 5 orders of magnitude with initial ligand concentration 5 μM–50 mM) with **PhL^1**–Lu^{III}–MOPS/NaOH buffer (pH 7.0) system at 80 °C while keeping the constant ratio of reagents (1.0 : 1.5 : 50). **A.** After one hour, conditions **I** to **IV** (50 mM–50 μM) yielded similar amounts of **Ph**{**Lu**}, indicating that the complexation step was still faster than the cycloaddition under these conditions (at 50 mM, the reaction proceeded as a suspension and also produced slightly more intermolecular by-products). Conditions **V** (5 μM) yielded significantly less amount of **Ph**{**Lu**} as the complexation became rate-limiting step due to high dilution (note the **PhL^1**/[**Lu**(**PhL^1**)] ratio). **B.** After one day, the active reagents **PhL^1** and [**Lu**(**PhL^1**)] were consumed, and conditions **I** to **IV** showed high **Ph**{**Lu**} conversion. For the most dilute mixture, **Ph**{**Lu**} was still the most abundant species, although significant amounts of **Ph**{**Ca**} were also observed, resulting from Ca^{II} leached from reaction vessel glass. **C.** After one week, no significant changes were observed compared with one day, consistent with the irreversibility of the ClickZip process. ***Analysis:*** HPLC with UV detection at λ = 280 nm (H_2O–MeCN gradient with FA additive). Purity of **PhL^1** just after isolation is labelled CONTROL. Source data available in Supplementary Data 2.

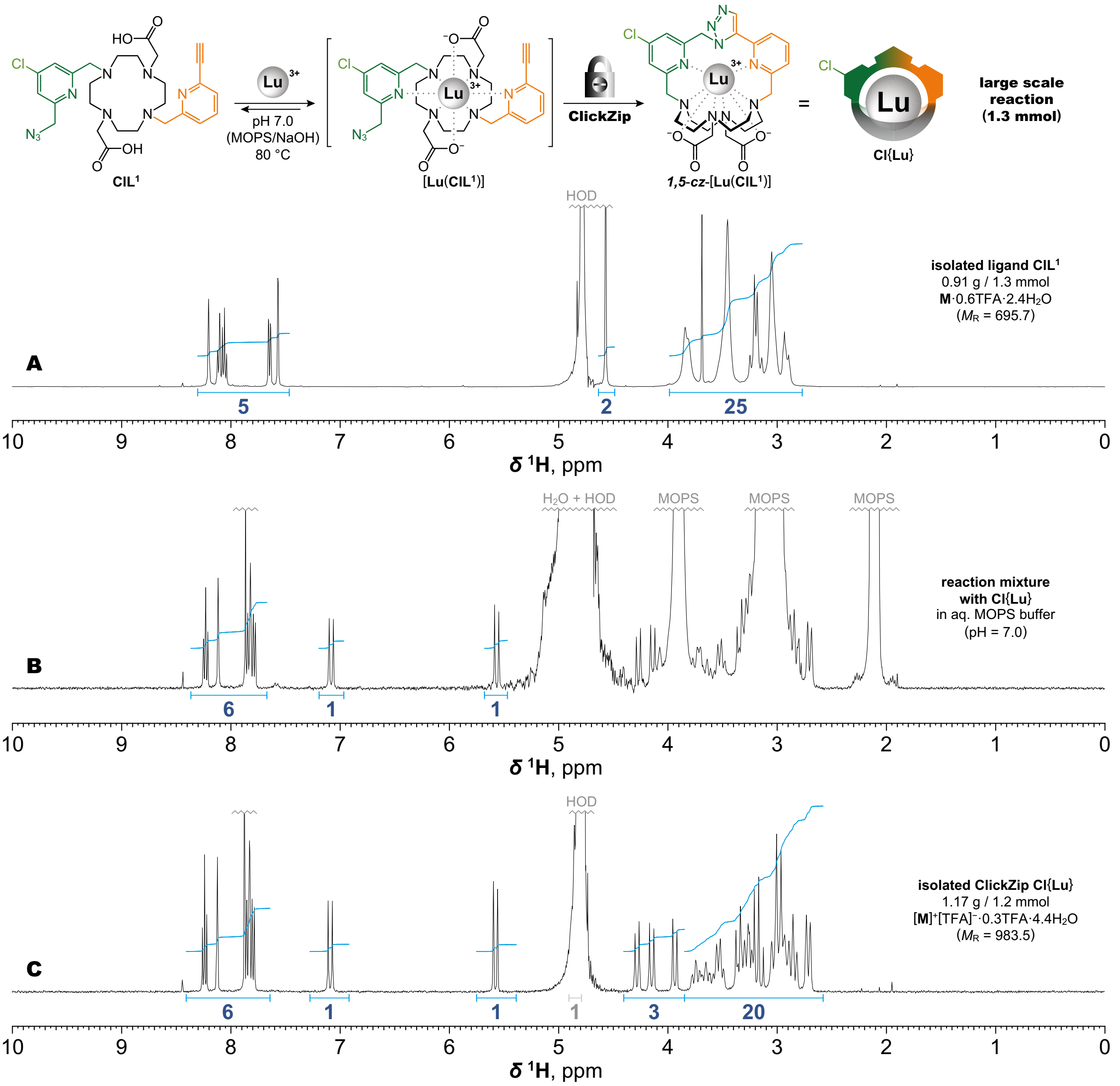

Supplementary Fig. 6. ClickZip on large scale. Demonstration of the effectivness of the ClickZip reaction for **Cl**{**Lu**} synthesis on a large scale (starting from 1.3 mmol of **ClL**[1]). **A.** Control ^{1}H NMR (401.0 MHz) of the input **ClL**[1] ligand in D_2O. **B.** ^{1}H NMR (401.0 MHz) of the reaction mixture (in aq. MOPS/NaOH buffer pH 7.0; diluted to 10% D_2O content before analysis) after 12 h at 80 °C with practically complete conversion of **ClL**[1] to **Cl**{**Lu**} (signals in the aliphatic region overlaid with buffer signals). **C.** ^{1}H NMR (401.0 MHz) of the isolated **Cl**{**Lu**} in D_2O with 91% yield.

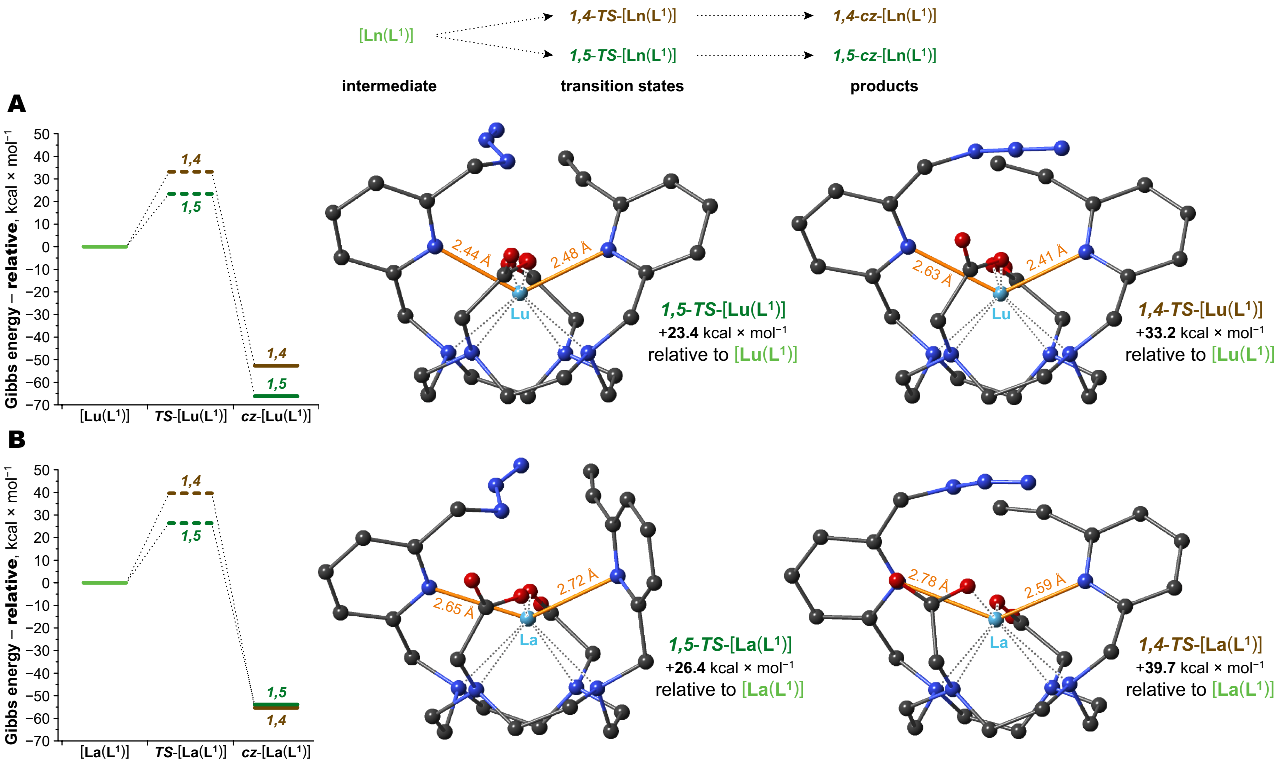

Supplementary Fig. 7. Theoretical PBE-D3/def2TZVP/CPCM calculations for ClickZip with La^{III} and Lu^{III}. Exclusive formation of the *1,5*-triazole during Ln^{III} ClickZip is supported by theoretical calculations on the **L^1** + Ln^{III} system (represented by largest La^{III} and smallest Lu^{III}). The Gibbs free energies (all relative to [**Ln(L^1)**] intermediate) of both ***1,5-cz***-[**Ln(L^1)**] and ***1,4-cz***-[**Ln(L^1)**] show clear thermodynamic preference for the formation of the triazole ring. The origin of the regioselectivity mainly lies in the lower activation barrier of transition state ***1,5-TS***-[**Ln(L^1)**] compared to transition state ***1,4-TS***-[**Ln(L^1)**]. Formation of the triazole requires pre-organization of the alkyne and azide moieties, which, in turn, requires temporary decoordination of the pyridine rings (the accompanying structures of transition states are shown with a distance between the Ln^{III} ion and nitrogen donor instead of a coordination bond). **A.** The Gibbs free energies of the **L^1** + Lu^{III} system where formation of ***1,5-cz***-[**Lu(L^1)**] is favoured from both the thermodynamic (***1,5-cz***-[**Lu(L^1)**] < ***1,4-cz***-[**Lu(L^1)**]) and kinetic perspective (***1,5-TS***-[**Lu(L^1)**] < ***1,4-TS***-[**Lu(L^1)**]). **B.** Despite the slight thermodynamic preference for ***1,4-cz***-[**La(L^1)**]), the exclusive formation of ***1,5-cz***-[**La(L^1)**]) during La^{III} ClickZip indicates dominant effect of the kinetics (***1,5-TS***-[**La(L^1)**] < ***1,4-TS***-[**La(L^1)**]) for the reaction outcome.

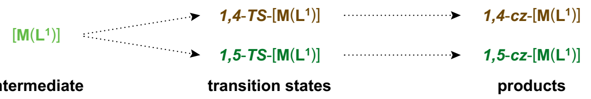

	Gibbs energy – relative to [M(L^1)], kcal × mol^{-1}					
**system**	***1,5-TS*-[M(L^1)]**	***1,4-TS*-[M(L^1)]**	***1,5-TS*-[M(L^1)] − *1,4-TS*-[M(L^1)]**	***1,5-cz*-[M(L^1)]**	***1,4-cz*-[M(L^1)]**	***1,5-cz*-[M(L^1)] − *1,4-cz*-[M(L^1)]**
**L^1 + Lu^{III}**	+23.4	+33.2	**−9.8**	−66.1	−52.7	**−13.4**
**L^1 + La^{III}**	+26.4	+39.7	**−13.3**	−53.8	−55.3	**+1.4**
**L^1 + Ca^{II}**	+25.0	+35.4	**−10.4**	−66.1	−61.5	**−4.6**
**L^1 + Li^{I}**	+28.4	+31.9	**−3.4**	−74.2	−67.7	**−6.5**
**L^1 + Na^{I}**	+25.1	+31.9	**−6.7**	−73.3	−69.7	**−3.5**
**L^1 + K^{I}**	+30.1	+29.7	**+0.5**	−56.4	−65.7	**+9.3**

Supplementary Fig. 8. Theoretical PBE-D3/def2TZVP/CPCM calculations for ClickZip with selected metals. Table with Gibbs free energies of **L^1** + La^{III}/Lu^{III} systems (Supplementary Fig. 7) accompanied by **L^1** + Ca^{II}/Li^{I}/Na^{I}/K^{I} systems. The differences in energy of transition states for Ca^{II} and Na^{I} ions follow the same trend as for Lu^{III} that correlates well with the experimental data. No ***1,4-cz***-[**Ca**(**PhL^1**)] was observed during the Ca^{II} ClickZip (data not shown) and strong templating effects towards the formation of empty cage ***1,5-cz*-PhL^1** were seen in the presence of Na^{I} ions. Due to the mismatch between the typical coordination environment of Li^{I} ions (coordination number 5–6) and number of available donors in ligand **L^1** (8), it is not easy to explain the behaviour of **PhL^1** in the presence of Li^{I} ions (detrimental effect on formation of both empty cages ***1,4-cz*-PhL^1** and ***1,5-cz*-PhL^1**) by the values obtained from theoretical calculations. The only predicted preference for formation of *1,4*-triazole (from both the thermodynamic and kinetic perspective) was in the presence of K^{I} ions. However, the seemingly matching experimental observation (preference for formation of empty cage ***1,4-cz*-PhL^1**) is more likely the result of a reaction pH on the reaction output (same ratios of ***1,4-cz*-PhL^1**/***1,5-cz*-PhL^1** were observed in presence of K^{I}, Rb^{I} and Cs^{I} ions) than effect of the K^{I} ions themselves (K^{I} ion is probably too large to even form [**K**(**PhL^1**)] intermediate).

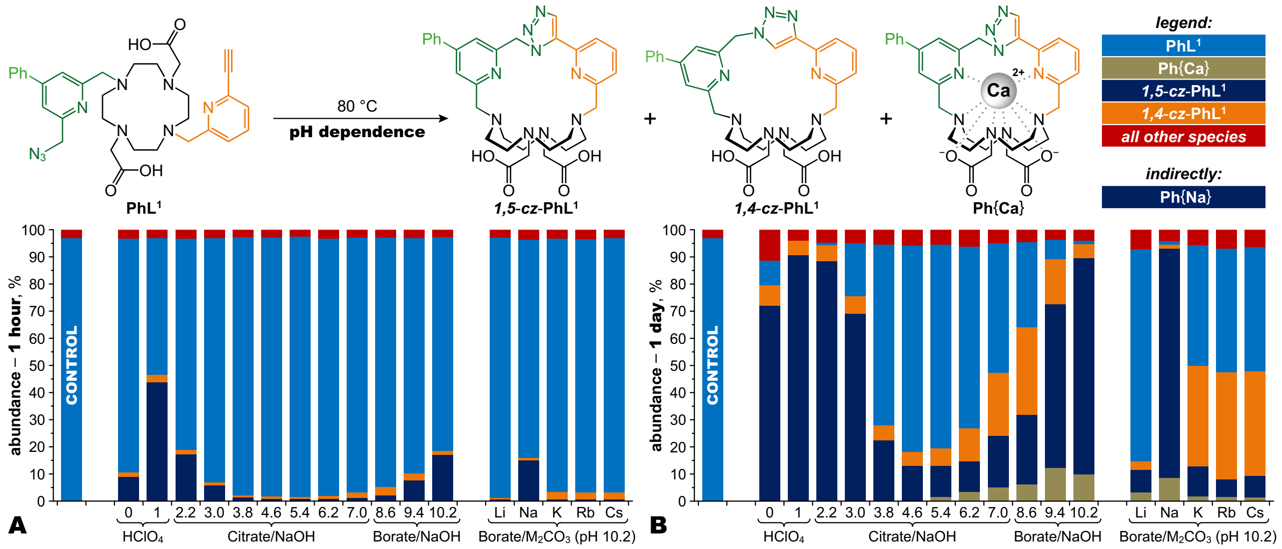

Supplementary Fig. 9. Formation of empty cages. Both of the expected Huisgen cycloaddition adducts (empty cages ***1,4-cz*-PhL¹** and ***1,5-cz*-PhL¹**) were obtained by simple heating of **PhL¹** in aqueous environment. Unlike the intermolecular reaction between the alkyne and azide groups (where regioselectivity is governed mainly by electronic factors) the intramolecular reaction is controlled predominantly by steric effects. Due to the presence of a cyclen moiety capable of forming rich networks of intramolecular hydrogen bonds, regioselectivity can be further tuned by simple pH control. ***Conditions:*** 0.5 mM **PhL¹** in 50 mM aq. buffer (except for 1.0 M and 0.1 M $HClO_4$) at 80 °C. ***Analysis:*** λ = 280 nm (HPLC, H_2O–MeCN, FA additive). Purity of the starting ligand **PhL¹** just prior to the experiment is labelled CONTROL. Identified species are distinguished by coloured bars (all other species are summarized by a red bar). **A.** Preferential formation of ***1,5-cz*-PhL¹** over ***1,4-cz*-PhL¹** under both acidic and alkaline conditions after 1 hour was observed. The drop in the formation rate of ***1,5-cz*-PhL¹** from pH 1 to 0 indicates the templating effect of H^+ ions through hydrogen bonding (as opposed to acid catalysis). **B.** High conversions of ***1,5-cz*-PhL¹** were observed under both highly acidic and highly alkaline conditions after 1 day. At or around neutral pH (typically suitable for metal ion complexation), starting ligand **PhL¹** was still the most abundant species. However, above pH 5, formation of unexpected **Ph{Ca}** (formally ***1,5-cz*-[Ca(PhL¹)]**) was observed, even though no Ca^{II} ions were purposely given into the mixtures and Milli-Q water as well as high-quality reagents were used for the experiment. Although a series of additional experimental points with Borate/M_2CO_3 buffers (pH 10.2, M = Li–Cs) in plastic Eppendorf tubes (glass vials used for the early points) did not fully suppress the **Ph{Ca}**, the clear dependence of the cycloaddition on the choice of alkali metal cation was observed. Despite the strong templating effect of Na^I ions (similar in size to Ca^{II} ions), no **Ph{Na}** was directly detected (likely due to low stability in acidic conditions, resulting in detection as ***1,5-cz*-PhL¹** instead). The binding of much smaller Li^I ion apparently prevents the pyridine arms reaching each other, effectively slowing the cycloaddition. The uniform behaviour of all larger alkali metal cations hints at their inability to affect the cycloaddition even as short-lived intermediates, and therefore reveal the true effect of alkaline pH (preference for ***1,4-cz*-PhL¹** in contrast to acidic conditions) in a way that Li^I, Na^I and Ca^{II} ions prevent. Source data available in Supplementary Data 2.

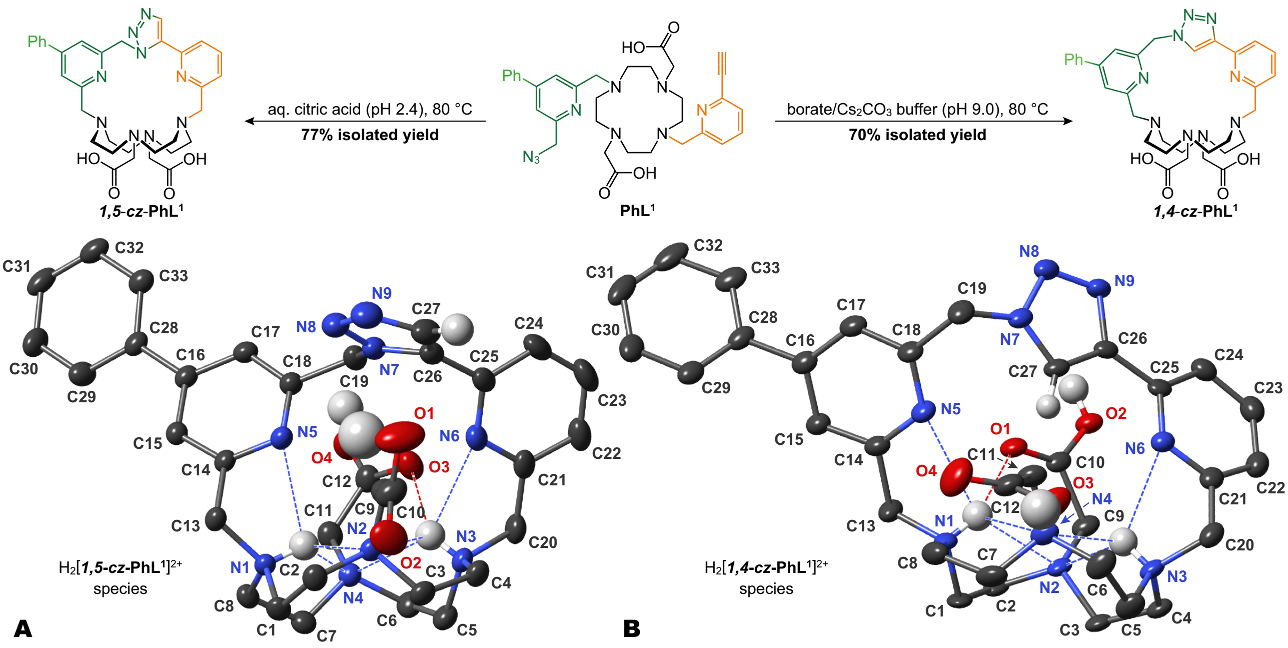

C

Intramolecular hydrogen bonds (*1,5-cz*-PhL[1])

D–H–A	N1–H–N2	N1–H–N4	N1–H–N5	N3–H–N2	N3–H–N4	N3–H–N6	N3–H–O3
D ↔ H, Å	0.90	0.90	0.90	0.88	0.88	0.88	0.88
A ↔ H, Å	2.43	2.46	2.34	2.65	2.56	2.39	2.69
D ↔ A, Å	2.90	2.93	2.81	3.01	2.99	2.72	3.23
∢DHA, °	113	112	112	106	111	102	120

Intramolecular hydrogen bonds (*1,4-cz*-PhL[1])

D–H–A	N1–H–N2	N1–H–N4	N1–H–N5	N1–H–O1	N3–H–N2	N3–H–N4	N3–H–N6
D ↔ H, Å	0.89	0.89	0.89	0.89	0.88	0.88	0.88
A ↔ H, Å	2.63	2.71	2.57	2.51	2.42	2.57	2.34
D ↔ A, Å	3.03	3.05	2.86	3.18	2.89	3.02	2.80
∢DHA, °	109	104	100	133	114	112	113

Supplementary Fig. 10. Solid-state structures of empty cages. Empty cage isomers ***1,5-cz*-PhL[1]** and ***1,4-cz*-PhL[1]** were obtained from **PhL[1]** in high yields under optimized conditions and their structures were unambiguously determined by X-ray analysis. Both structures were obtained in the same protonated state (H_2**L**)$^{2+}$ with rich networks of intramolecular hydrogen bonds (dashed) inside the cavities. The perchlorate anions, water molecules, and carbon-bound hydrogen atoms (except for the hydrogen atom on the triazole ring) were omitted for clarity. Thermal ellipsoids were set at 50% probability. **A.** Structure of [H_2(***1,5-cz*-PhL[1]**)]$^{2+}$ cation found in the crystal structure of [H_2(***1,5-cz*-PhL[1]**)]$^{2+}$[ClO_4]$_2^{-}$·7.0H_2O. **B.** Structure of [H_2(***1,4-cz*-PhL[1]**)]$^{2+}$ cation found in the crystal structure of [H_2(***1,4-cz*-PhL[1]**)]$^{2+}$[ClO_4]$_2^{-}$. **C.** Parameters of all intramolecular hydrogen bonds.

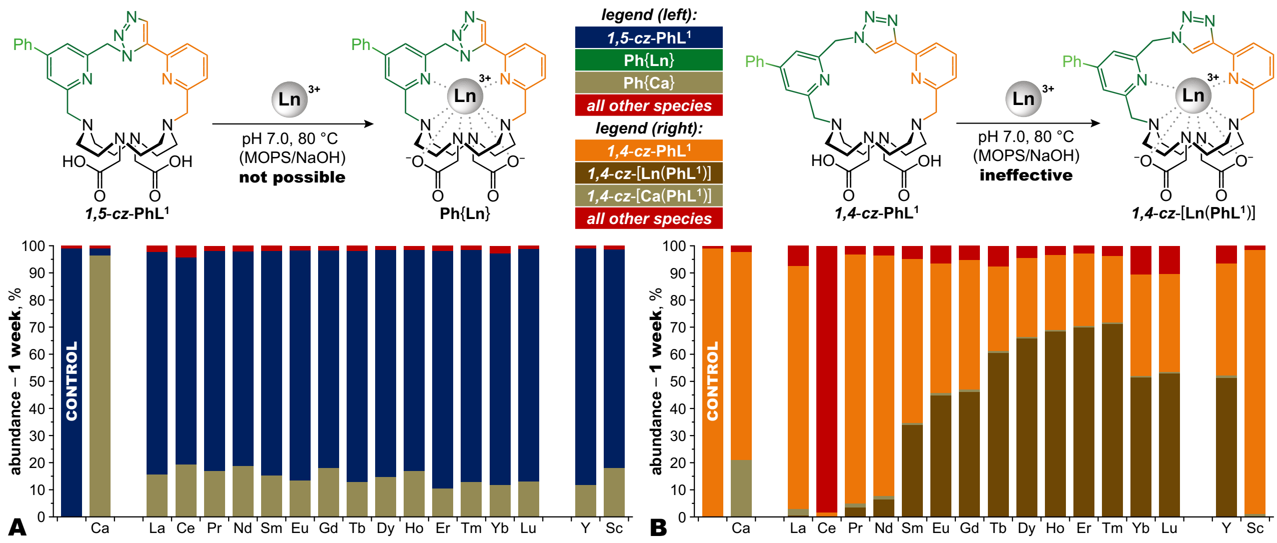

Supplementary Fig. 11. Direct complexation. Comparison of the ability of empty cages ***1,5-cz*-PhL^{1}** and ***1,4-cz*-PhL^{1}** to complex Ln^{III} ions. ***Conditions:*** 0.5 mM ***1,5-cz*-PhL^{1}** or ***1,4-cz*-PhL^{1}** and 1.0 mM Ln^{III} salt (including Y^{III} and Sc^{III}) in 50 mM aq. MOPS/NaOH buffer (pH 7.0) at 80 °C. ***Analysis:*** λ = 280 nm for ***1,5-cz*-PhL^{1}** series and λ = 275 nm for ***1,4-cz*-PhL^{1}** series (HPLC, H_2O–MeCN, FA additive). Purity of the corresponding starting empty cage is labelled CONTROL. **A.** No formation of **Ph**{**Ln**} was observed with any Ln^{III} ions when starting with ***1,5-cz*-PhL^{1}** after 1 week at 80 °C, not even with the smallest Sc^{III}. Instead, some **Ph**{**Ca**} formed (and was verified by quantitative formation of the same when using Ca^{II} instead of Ln^{III}; column Ca). Prolonged heating of two samples (Gd^{III} and Lu^{III}) for 6 months did not show any improvement – not even a trace of **Ph**{**Gd**} or **Ph**{**Lu**} was observed (Supplementary Fig. 12). **B.** A slightly larger cavity of ***1,4-cz*-PhL^{1}** allowed for most of the Ln^{III} ions to be accommodated by direct complexation. While ≥50% conversion was achieved for most of the Ln^{III} ions after 1 week at 80 °C, the process was later found to be inefficient as quantitative conversion was not reached after prolonged heating even with larger excess of the Ln^{III} ions. Although direct complexation with Ca^{II} ions (column Ca) led to formation of some ***1,4-cz*-[Ca(PhL^{1})]**, the conversion was much lower compared to **Ph**{**Ca**}. Source data available in Supplementary Data 2.

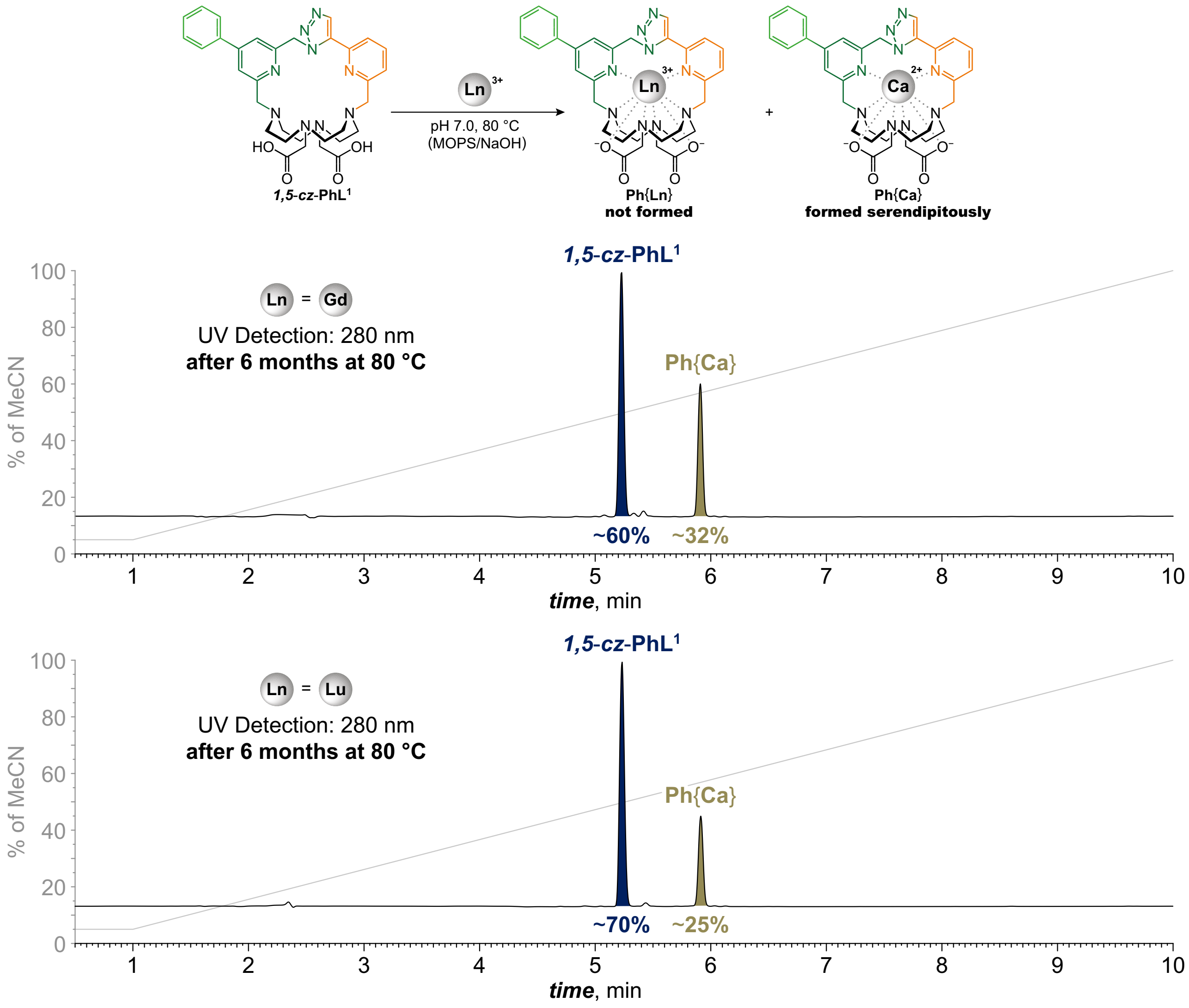

Supplementary Fig. 12. Inapplicability of direct Ln^{III} complexation with *1,5-cz*-PhL^1. Attempts at direct complexation of Ln^{III} (using Gd^{III} and Lu^{III} as examples) with the empty cage ***1,5-cz*-PhL^1** did not lead to the formation of **Ph{Ln}** even after 6 months at 80 °C. From the HPLC chromatograms, only serendipitous **Ph{Ca}** formation was observed along with minor degradation of the starting ***1,5-cz*-PhL^1**. This highlights the necessity of using the ClickZip principle for the synthesis of otherwise inaccessible cryptate-type **Ph{Ln}** complexes. ***Conditions:*** 0.5 mM ***1,5-cz*-PhL^1** and 1.0 mM Gd^{III} or Lu^{III} salt in mM aq. MOPS/NaOH buffer (pH 7.0) at 80 °C. ***Analysis:*** HPLC with UV detection at λ = 280 nm (H_2O–MeCN gradient with FA additive).

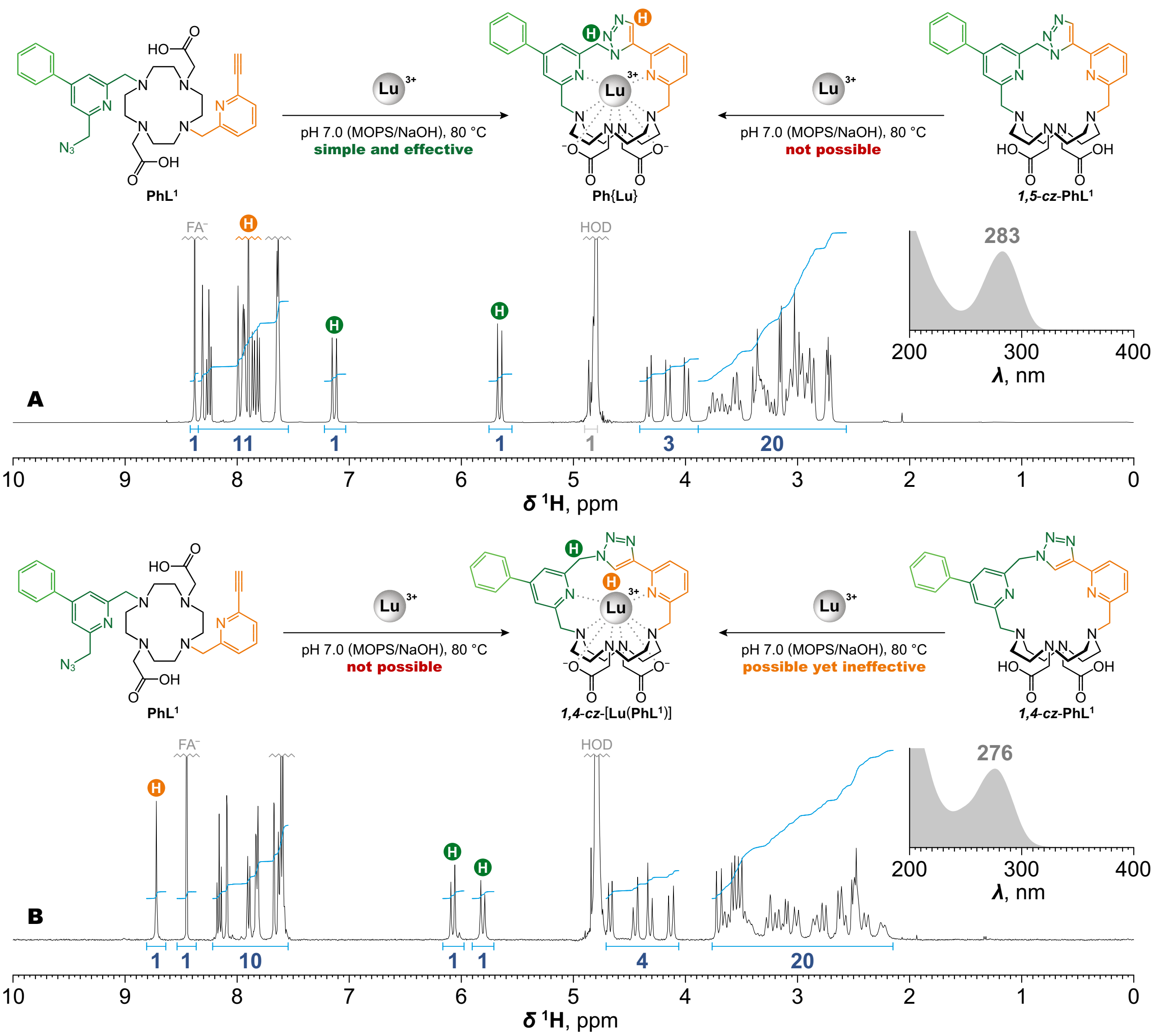

Supplementary Fig. 13. Comparison of Ln^{III} ClickZip with Ln^{III} direct complexation. Contrast in the complexation possibilities of Ln^{III} ions between different forms of the ligand (open form **L^1** and empty cage forms ***1,5-cz*-PhL^1** and ***1,4-cz*-PhL^1**). This overview is shown on the example of the Lu^{III}–**PhL^1** system, but is valid for the whole family of **L^1** ligands and all Ln^{III} (including Y^{III}). **A.** Extremely stable **Ph{Lu}** can be readily obtained using the ClickZip principle from **PhL^1**, but it is not possible to obtain it by direct complexation from ***1,5-cz*-PhL^1**. The sharp nature of ^{1}H NMR signals (unusual for Ln^{III} chelates) indicates high rigidity of the chelate. A characteristic feature of ^{1}H NMR (401.0 MHz) spectrum of **Ph{Lu}** is the shift of the triazole signal (around 7.9 ppm; orange) and the large difference in the shift at the bridging methylene group (around 1.5 ppm from each other; green). **B.** The much less stable ***1,4-cz*-[Lu(PhL^1)]** cannot be obtained using the ClickZip principle from **PhL^1** (**Ph{Lu}** is formed exclusively) but it can be obtained by direct complexation from ***1,4-cz*-PhL^1**. However, the reaction is inefficient (required prolonged heating and does not achieve quantitative conversion even when using excess of Lu^{3+}). Nevertheless, ***1,4-cz*-[Lu(PhL^1)]** can be isolated in high purity in low to moderate yield. In contrast to **Ph{Lu}**, the triazole signal in ^{1}H NMR (401.0 MHz) is shifted more downfield (around 8.7 ppm; orange) and the difference in shift at the bridging methylene group is smaller (around 0.3 ppm from each other; green).

compound	$t_{½}$ (6.0 M HCl, 80 °C), h	$t_{½}$ (1.0 M HCl, 80 °C), h	$t_{½}$ (0.1 M HCl, 80 °C), h	$t_{½}$ (0.1 M HCl, 25 °C), h
**Ph{Lu}**	$2.53 \times 10^4 \pm 1.70 \times 10^3$	too stable	too stable	too stable
**Ph{Yb}**	$4.69 \times 10^3 \pm 2.10 \times 10^1$	too stable	too stable	too stable
**Ph{Tm}**	$1.36 \times 10^3 \pm 1.22 \times 10^1$	too stable	too stable	too stable
**Ph{Er}**	$3.43 \times 10^2 \pm 1.22 \times 10^1$	$5.92 \times 10^4 \pm 1.13 \times 10^3$	too stable	too stable
**Ph{Ho}**	$6.82 \times 10^1 \pm 2.73 \times 10^0$	$1.32 \times 10^4 \pm 2.62 \times 10^2$	too stable	too stable
**Ph{Y}**	$4.50 \times 10^1 \pm 3.10 \times 10^{-1}$	$5.94 \times 10^3 \pm 1.36 \times 10^2$	$1.12 \times 10^6 \pm 1.64 \times 10^4$	too stable
**Ph{Dy}**	$1.20 \times 10^1 \pm 1.25 \times 10^{-1}$	$3.50 \times 10^3 \pm 1.19 \times 10^2$	$9.50 \times 10^4 \pm 2.98 \times 10^3$	too stable
**Ph{Tb}**	2.10×10^0	$3.72 \times 10^2 \pm 3.20 \times 10^1$	$3.17 \times 10^4 \pm 3.26 \times 10^3$	too stable
**Ph{Gd}**	1.70×10^{-1}	$3.04 \times 10^1 \pm 4.89 \times 10^{-1}$	$2.47 \times 10^3 \pm 3.72 \times 10^1$	too stable
**Ph{Eu}**	too unstable	$7.30 \times 10^0 \pm 2.63 \times 10^{-1}$	$3.80 \times 10^2 \pm 5.80 \times 10^1$	too stable
**Ph{Sm}**	too unstable	1.46×10^0	$1.11 \times 10^2 \pm 7.46 \times 10^{-1}$	$1.34 \times 10^4 \pm 4.38 \times 10^2$
**Ph{Nd}**	too unstable	too unstable	2.55×10^0	$1.79 \times 10^2 \pm 1.19 \times 10^1$
**Ph{Pr}**	too unstable	too unstable	4.67×10^{-1}	$5.17 \times 10^1 \pm 2.25 \times 10^0$
**Ph{Ce}**	too unstable	too unstable	too unstable	$1.65 \times 10^1 \pm 2.85 \times 10^{-1}$
**Ph{La}**	too unstable	too unstable	too unstable	$2.48 \times 10^0 \pm 2.50 \times 10^{-1}$
**Ph{Ca}**	too unstable	too unstable	too unstable	7.34×10^{-1}
***1,4-cz*-[Lu(PhL¹)]**	too unstable	too unstable	too unstable	$5.07 \times 10^1 \pm 4.78 \times 10^0$
***1,4-cz*-[Gd(PhL¹)]**	too unstable	too unstable	too unstable	$3.20 \times 10^1 \pm 2.13 \times 10^0$
**[Lu(NO₂BnDOTA)]**	too unstable	3.29×10^{-1}	$2.55 \times 10^0 \pm 9.60 \times 10^{-2}$	$1.20 \times 10^3 \pm 1.09 \times 10^2$
**[Gd(NO₂BnDOTA)]**	too unstable	3.67×10^{-1}	$4.87 \times 10^0 \pm 2.92 \times 10^{-1}$	$2.09 \times 10^3 \pm 1.62 \times 10^2$
**[La(NO₂BnDOTA)]**	too unstable	too unstable	2.93×10^{-1}	$1.62 \times 10^1 \pm 1.77 \times 10^{-1}$

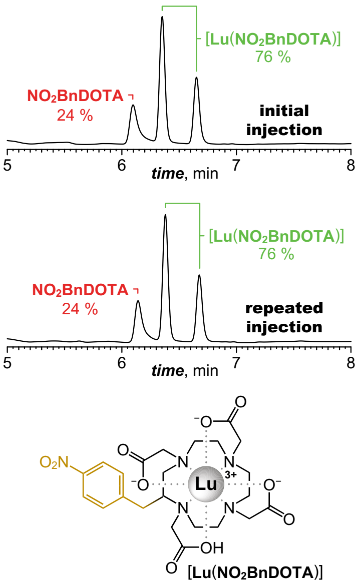

Supplementary Fig. 14. Kinetic inertness half-life comparison. Experimental data of kinetic inertness (Supplementary Fig. 15 to Supplementary Fig. 18) were fitted (excluding dimers ***1,4/1,4-cz*-[Ln(PhL¹)₂]**) by exponential decay function $y = \exp(-x/t)$ using Origin 9 software (y = fraction of intact chelate, x = time, t = decay constant). Kinetic inertness of the analyte was then expressed as half-life $t_{½}$ (in hours) under given conditions that was derived from decay constant ($t_{½} = \ln(2) \times t$). Error of the fit is not given for the case where only single time point (satisfying condition 100% > fraction of intact chelate > 0%) was usable for the fit. The aliquots of **Ph{Ln}** (including **Ph{Y}**) were neutralized with MOPS/NaOH buffer (pH 7.0) prior to injection on HPLC as no re-complexation of leaked Ln^{III} ions into empty cage ***1,5-cz*-PhL¹** could occur (Supplementary Fig. 12). For all other species (**Ph{Ca}**, ***1,4-cz*-[Ln(PhL¹)]** and **[Ln(NO₂BnDOTA)]**), the aliquots for HPLC analysis were diluted with either H_2O (for reaction mixtures in 0.1 M HCl) or partly neutralized by FA/NaOH buffer (pH 3.6, for reaction mixtures in 1.0 M or 6.0 M HCl) to avoid re-complexation. The validity of that approach is demonstrated on example of **[Lu(NO₂BnDOTA)]** in 0.1 M HCl at 80 °C after 1 h, where repeated analysis of the same aliquot resulted in identical ratio of **NO₂BnDOTA**/**[Lu(NO₂BnDOTA)]** = 24:76 (note that this ligand provides two isomeric chelates that are distinguished as two peaks but integrated as one). At neutral pH, the ligand **NO₂BnDOTA** would immediately re-chelate the free metal ions, providing false results for the rate of acid-assisted dechelation being measured.

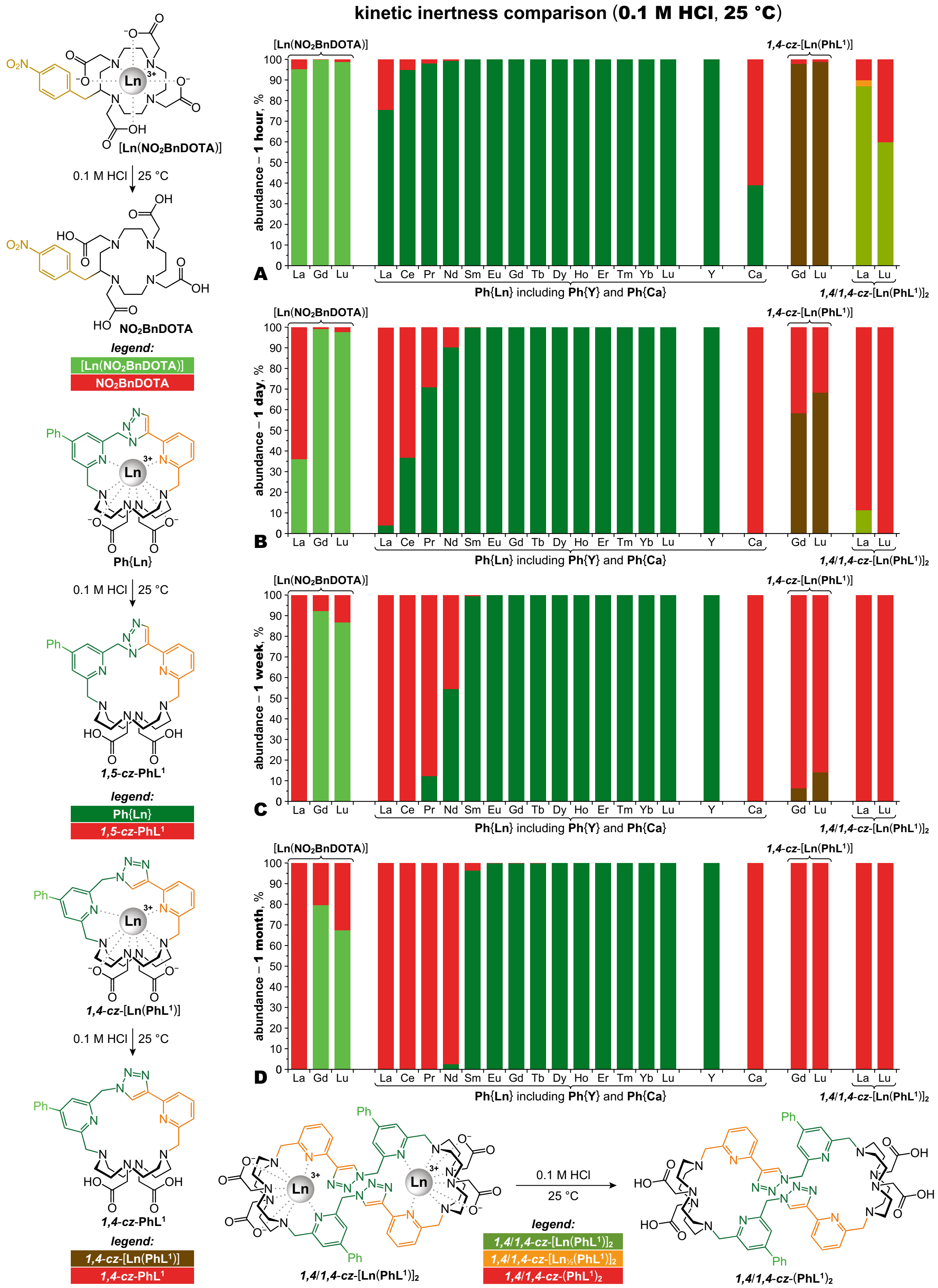

Supplementary Fig. 15. ClickZip kinetic inertness comparison. Stability of **Ph{Ln}** in acidic media (whole series including **Ph{Y}** and **Ph{Ca}**) and its comparison with ***1,4-cz*-[Ln(PhL¹)]** isomer (Ln^{III} = Gd^{III}, Lu^{III}), DOTA-derivative **[Ln(NO₂BnDOTA)]** (Ln^{III} = La^{III}, Gd^{III}, Lu^{III}) and dimers ***1,4/1,4-cz*-[Ln(PhL¹)]₂** (Ln^{III} = La^{III}, Lu^{III}). ***Conditions:*** 0.5 mM analyte in 0.1 M HCl, 25 °C. ***Analysis:*** HPLC with UV detection at λ_{max} of the given chelate (H_2O–MeCN gradient with FA additive). **A.** 1 hour. **B.** 1 day. **C.** 1 week. **D.** 1 month (30 days). Source data available in Supplementary Data 2.

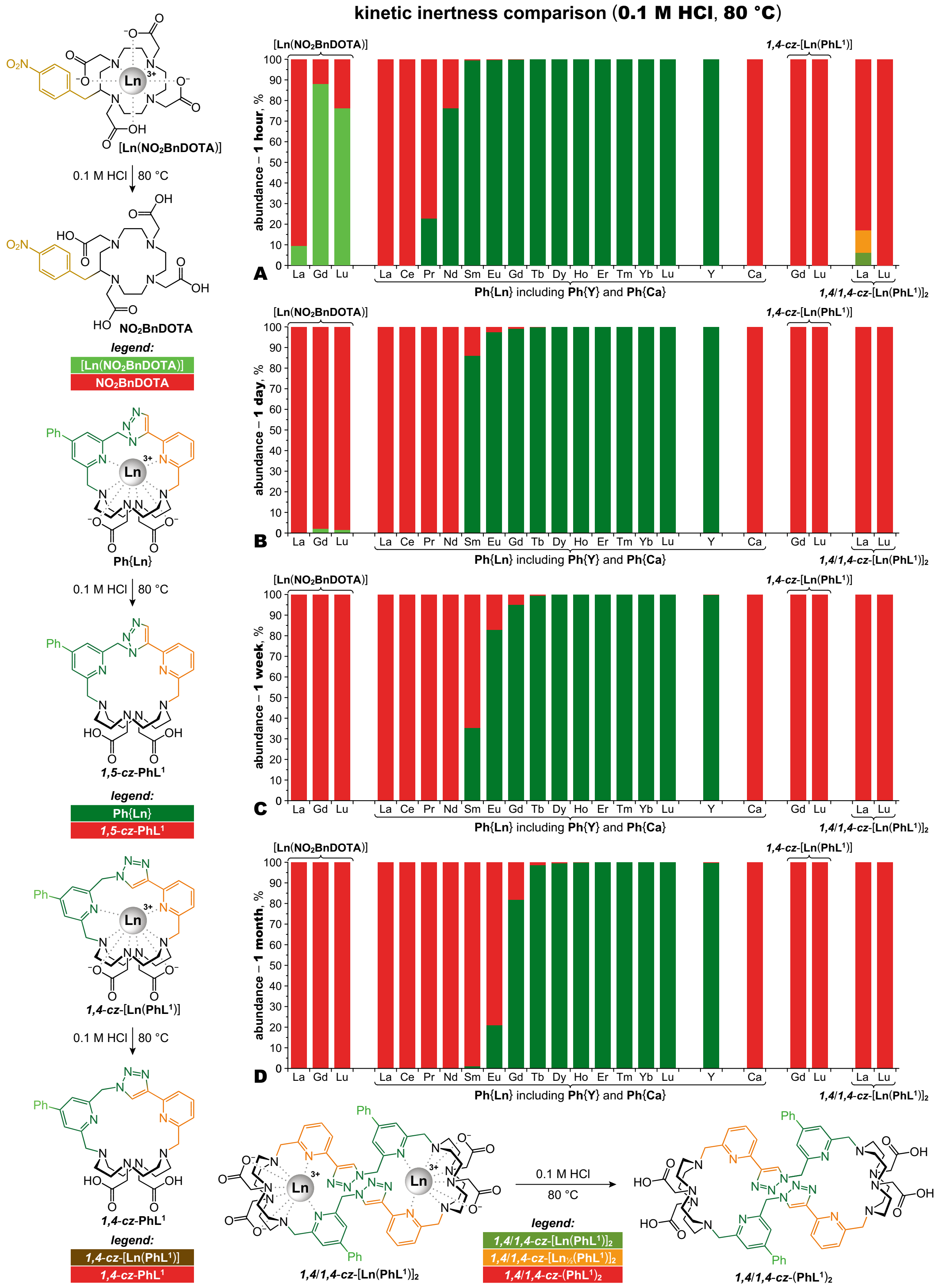

Supplementary Fig. 16. ClickZip kinetic inertness comparison. Stability of **Ph**{**Ln**} in acidic media (whole series including **Ph**{**Y**} and **Ph**{**Ca**}) and its comparison with ***1,4-cz***-[**Ln**(**PhL**^{1})] isomer (Ln^{III} = Gd^{III}, Lu^{III}), DOTA-derivative [**Ln**(**NO**$_2$**BnDOTA**)] (Ln^{III} = La^{III}, Gd^{III}, Lu^{III}) and dimers ***1,4/1,4-cz***-[**Ln**(**PhL**^{1})]$_2$ (Ln^{III} = La^{III}, Lu^{III}). ***Conditions:*** 0.5 mM analyte in 0.1 M HCl, 80 °C. ***Analysis:*** HPLC with UV detection at λ_{max} of the given chelate (H_2O–MeCN gradient with FA additive). **A.** 1 hour. **B.** 1 day. **C.** 1 week. **D.** 1 month (30 days). Source data available in Supplementary Data 2.

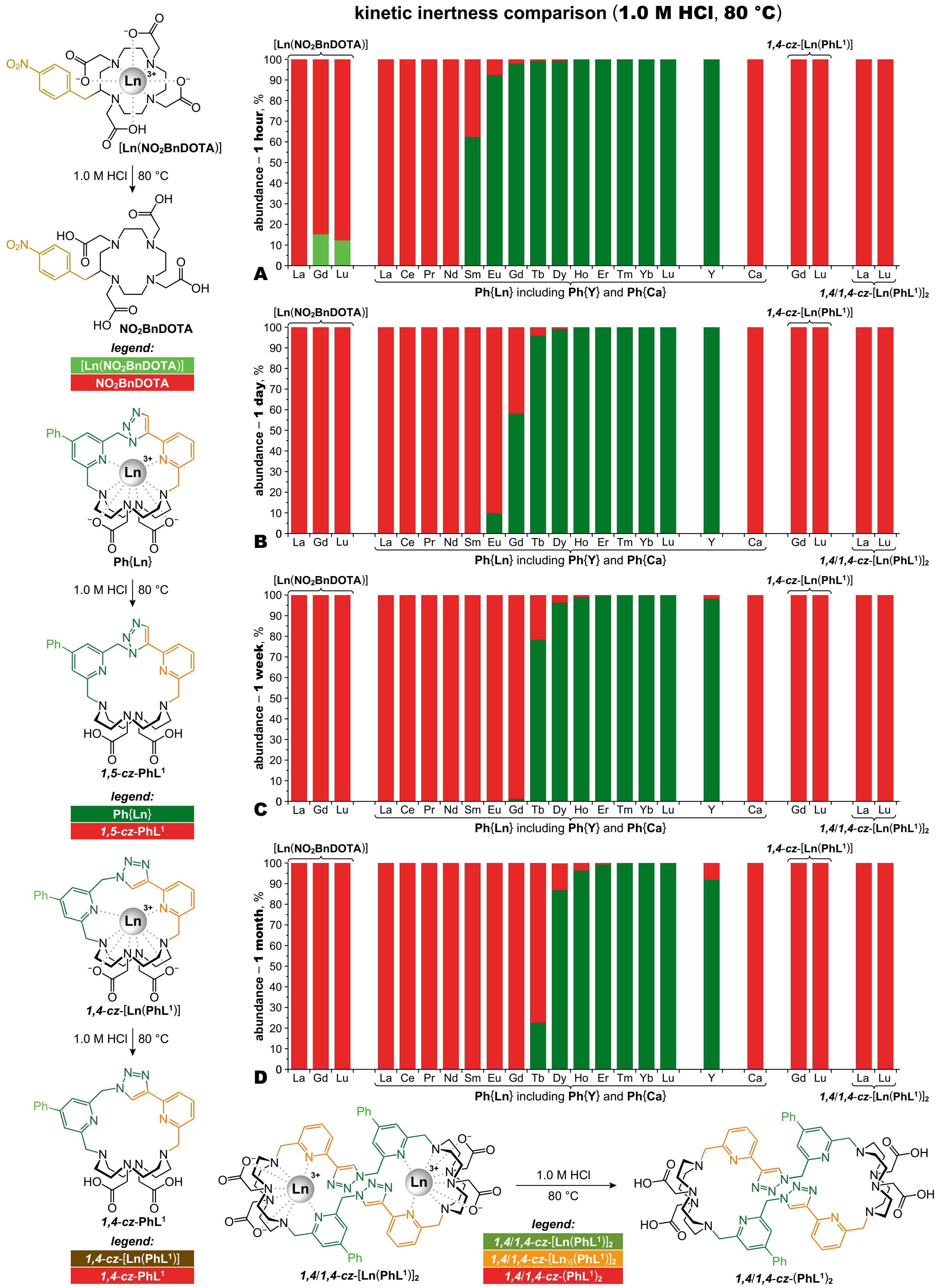

Supplementary Fig. 17. ClickZip kinetic inertness comparison. Stability of **Ph**{**Ln**} in acidic media (whole series including **Ph**{**Y**} and **Ph**{**Ca**}) and its comparison with ***1,4-cz***-[**Ln**(**PhL**[1])] isomer (Ln^{III} = Gd^{III}, Lu^{III}), DOTA-derivative [**Ln**(**NO2BnDOTA**)] (Ln^{III} = La^{III}, Gd^{III}, Lu^{III}) and dimers ***1,4/1,4-cz***-[**Ln**(**PhL**[1])]$_2$ (Ln^{III} = La^{III}, Lu^{III}). ***Conditions:*** 0.5 mM analyte in 1.0 M HCl, 80 °C. ***Analysis:*** HPLC with UV detection at λ_{max} of the given chelate (H_2O–MeCN gradient with FA additive). **A.** 1 hour. **B.** 1 day. **C.** 1 week. **D.** 1 month (30 days). Source data available in Supplementary Data 2.

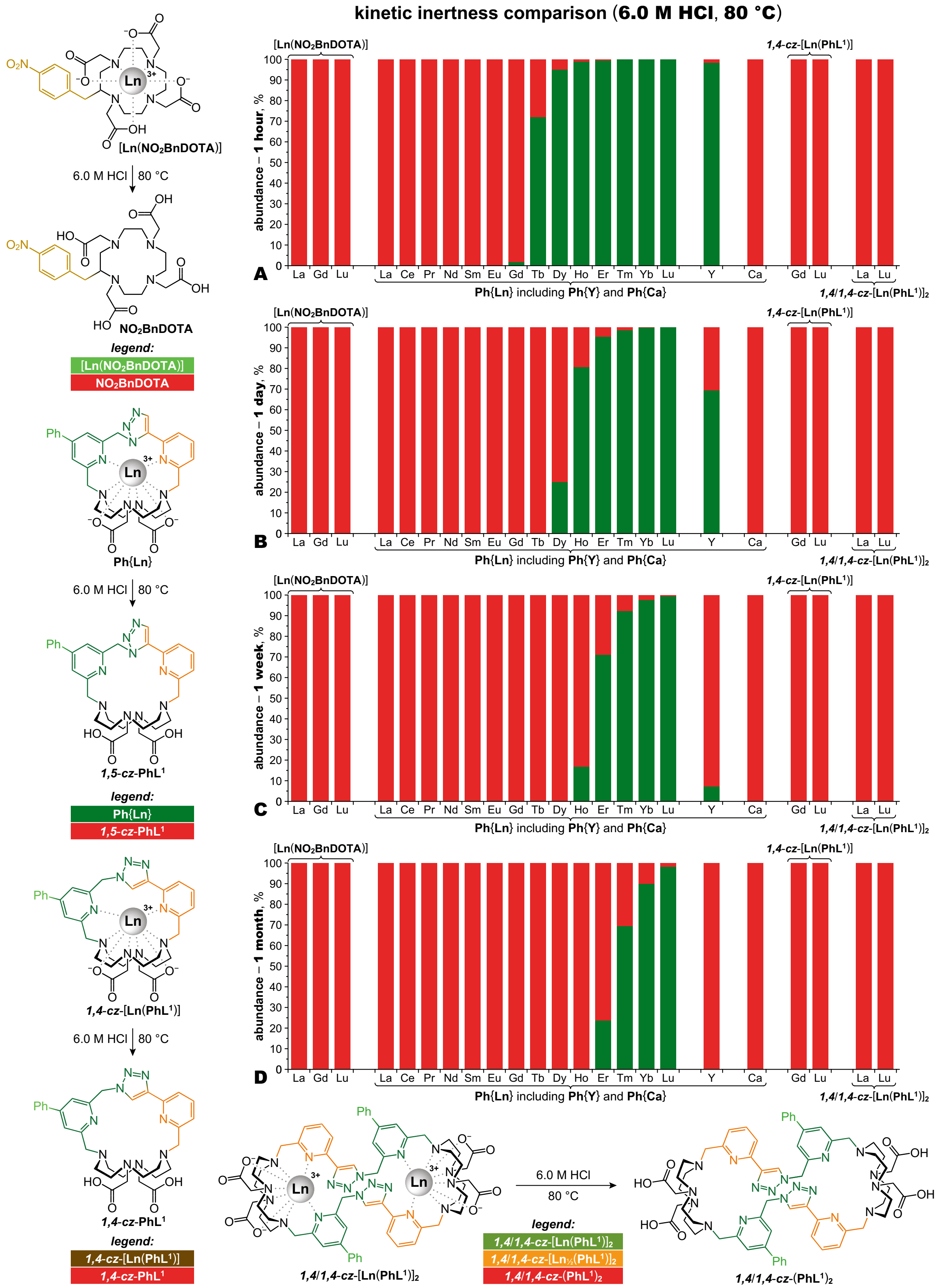

Supplementary Fig. 18. ClickZip kinetic inertness comparison. Stability of **Ph**{**Ln**} in acidic media (whole series including **Ph**{**Y**} and **Ph**{**Ca**}) and its comparison with ***1,4-cz***-[**Ln**(**PhL**[1])] isomer (Ln^{III} = Gd^{III}, Lu^{III}), DOTA-derivative [**Ln**(**NO₂BnDOTA**)] (Ln^{III} = La^{III}, Gd^{III}, Lu^{III}) and dimers ***1,4/1,4-cz***-[**Ln**(**PhL**[1])]$_2$ (Ln^{III} = La^{III}, Lu^{III}). ***Conditions:*** 0.5 mM analyte in 6.0 M HCl, 80 °C. ***Analysis:*** HPLC with UV detection at λ_{max} of the given chelate (H_2O–MeCN gradient with FA additive). **A.** 1 hour. **B.** 1 day. **C.** 1 week. **D.** 1 month (30 days). Source data available in Supplementary Data 2.

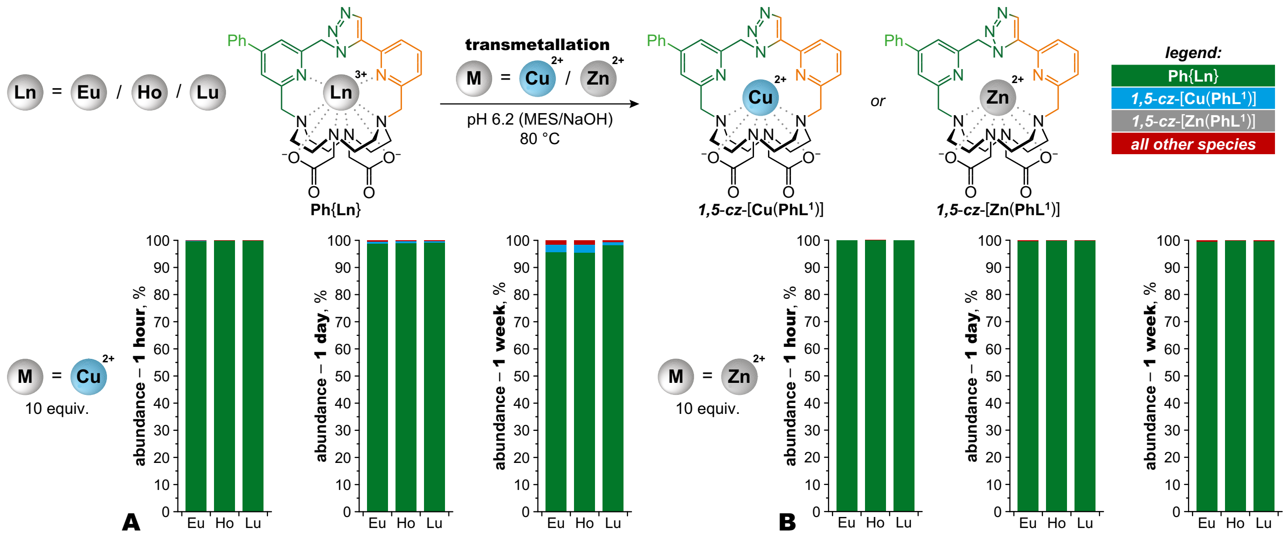

Supplementary Fig. 19. Transmetallation. Stability of **Ph**{**Ln**} (examplified with Ln^{III} = Eu^{III}, Ho^{III}, Lu^{III}) towards transmetallation by divalent metals (M^{II} = Cu^{II}, Zn^{II}). To limit the precipitation of $Cu(OH)_2$ and $Zn(OH)_2$, the pH was lowered from neutral to 6.2 (still a minor precipitation was observed in the case of Cu^{II}). **A.** High resistance to Cu^{II} transmetallation within one week at 80 °C (≤3% ***1,5-cz***-[**Cu**(**PhL**^{1})] observed after 1 week). **B.** Complete resistance to Zn^{II} transmetallation within one week at 80 °C (no ***1,5-cz***-[**Zn**(**PhL**^{1})] observed after 1 week). ***Conditions:*** 0.5 mM **Ph**{**Ln**} derivative (Ln^{III} = Eu^{III}, Ho^{III}, Lu^{III}) and 5.0 mM $M^{II}Cl_2$ salt (M^{II} = Cu^{II} or Zn^{II}) in 100 mM aq. MES/NaOH buffer (pH 6.2) at 80 °C. ***Analysis:*** HPLC with UV detection at λ = 280 nm (H_2O–MeCN gradient with FA additive). Source data available in Supplementary Data 2.

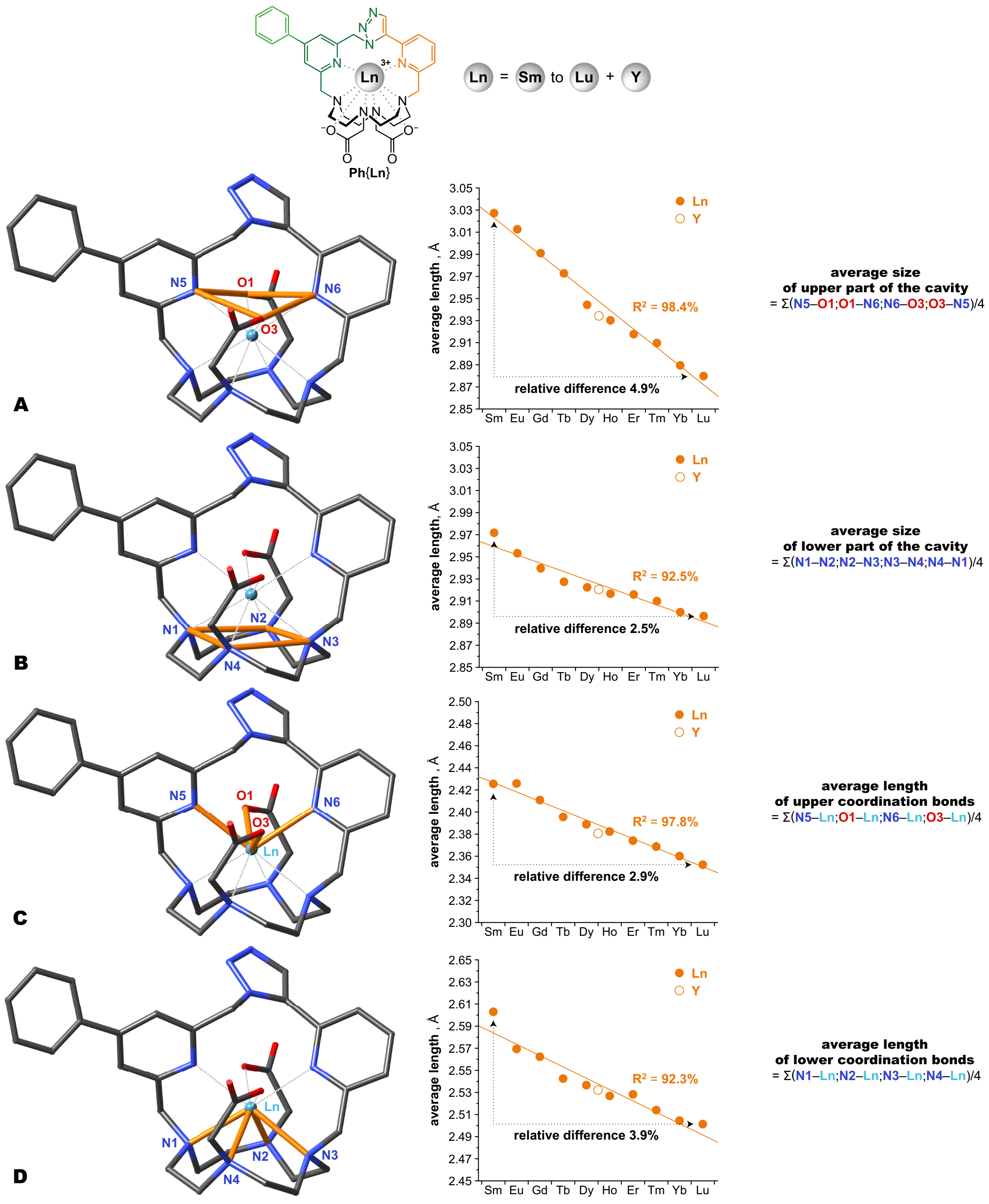

Supplementary Fig. 20. Isostructurality of Ph{Ln} in solid state. The structures of **Ph**{**Sm**} to **Ph**{**Lu**} in the solid state revealed the same coordination environment around the Ln^{III} ion. The size of the cavity in these ClickZip chelates (described by average distance between the donor atoms and the average length of the coordination bonds) gradually shrinks with decreasing ionic radius of the Ln^{III} ion. Nevertheless, the relative difference across the series in any given parameter is very small, indicating a high degree of isostructurality. The homogeneity of the series is further supported by inclusion of the **Ph**{**Y**} solid-state structure (empty orange circle; placed between Dy^{III} and Ho^{III} according to its ionic radius but excluded from the fit). The solid-state structure of **Ph**{**Pr**} was excluded due to the different coordination environment (coordination number 9 with one water molecule directly bound to the Pr^{III} ion). **A.** Average size of the upper part of the cavity. **B.** Average size of the lower part of the cavity. **C.** Average length of the upper coordination bonds. **D.** Average length of the lower coordination bonds. Source data available in Supplementary Data 2.

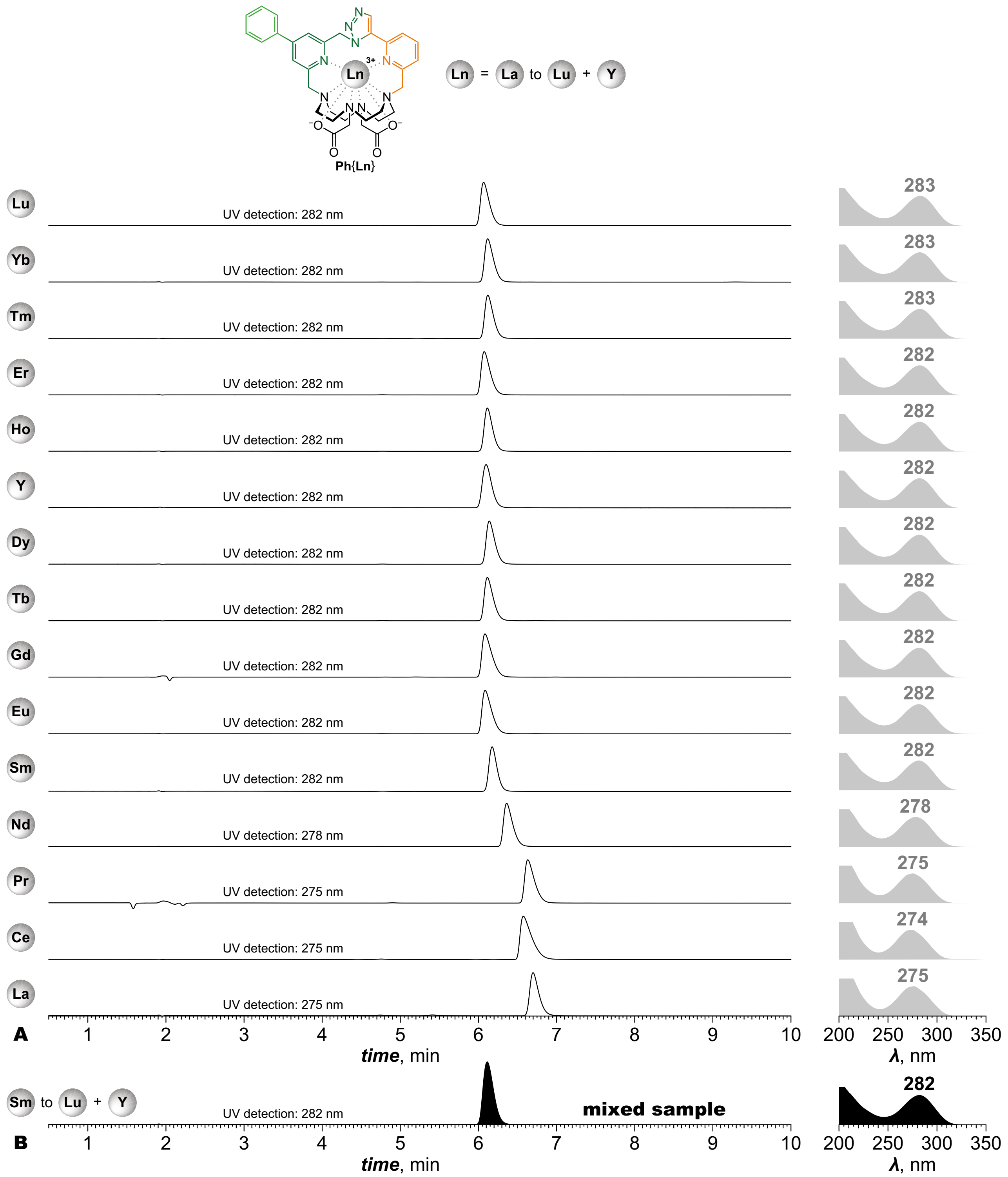

Supplementary Fig. 21. Isostructurality of Ph{Ln} in solution. Comparison of retention times of isolated **Ph**{**La**} to **Ph**{**Lu**} on LC-MS (together with their UV absorption profile). ***Analysis:*** HPLC with UV detection at λ_{max} of the given chelate (H_2O–MeCN gradient with FA additive, 15% to 40% MeCN from 0.5 min to 10 min). **A.** Nearly identical behavior of **Ph**{**Sm**} to **Ph**{**Lu**} (including **Ph**{**Y**} that was placed between Dy^{III} and Ho^{III} according to its ionic radius) indicates high degree of isostructurality within this subset of **Ph**{**Ln**} series despite several orders of magnitude difference in kinetic inertness. Deviation from isostructurality, both in retention time and absorption, are noticeable only for the lanthanides from La^{III} to Nd^{III}, indicating structural change (most likely coordination of water molecule to the Ln^{III} ion). This observation based on solution behavior correlates well with the conclusions obtained from the solid state (Supplementary Fig. 20). **B.** Identical retention time of a mixture of **Ph**{**Sm**} to **Ph**{**Lu**} including **Ph**{**Y**} with UV absorption profile of such mixture.

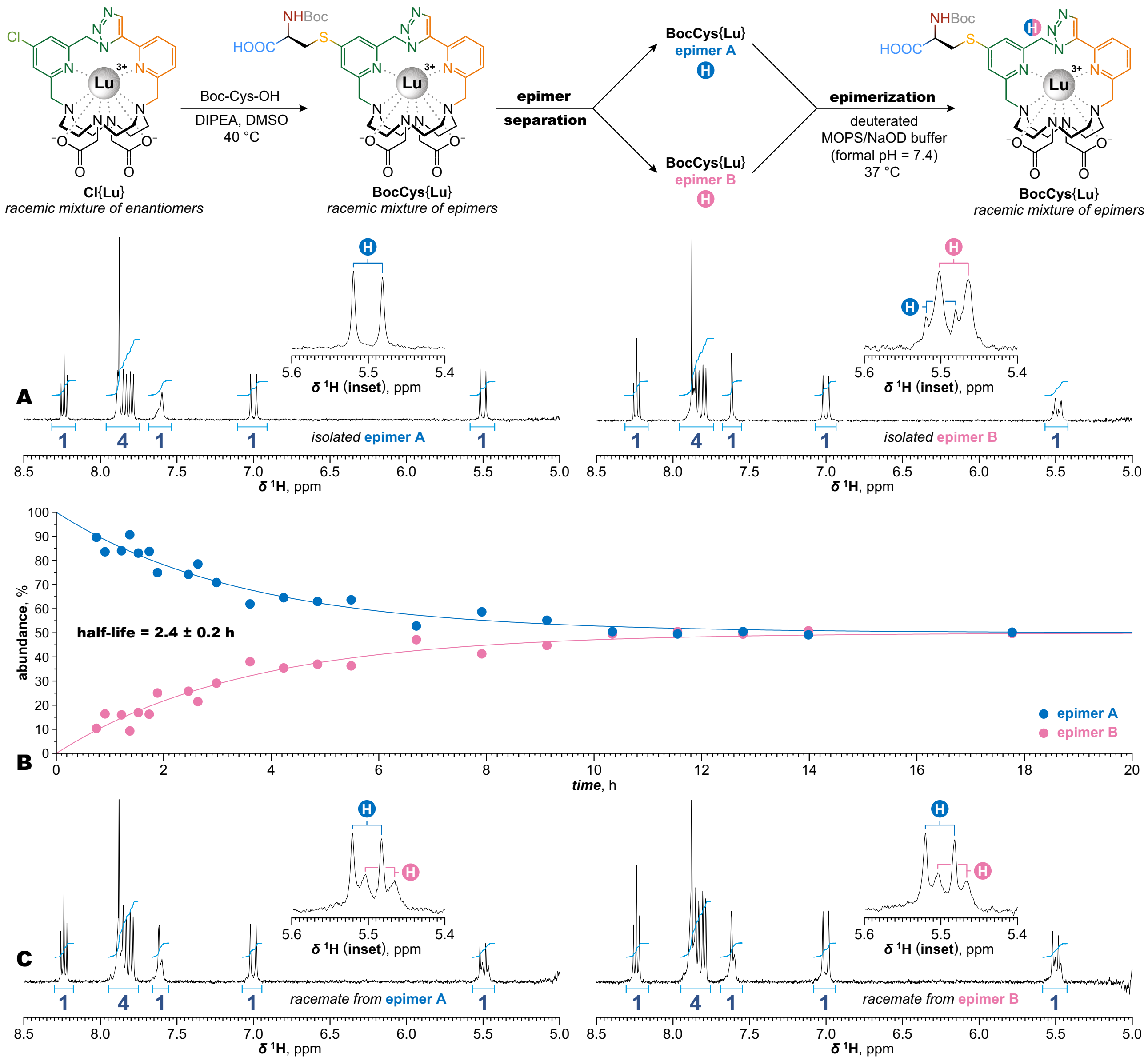

Supplementary Fig. 22. ClickZip chirality inversion (Δδδδδ ↔ Λλλλλ). The reaction of **Cl{Lu}** (racemic mixture of enantiomers due to the inherent chirality of the ClickZip) with Boc-L-Cys-OH yielded racemic mixture of **BocCys{Lu}** epimers, that was possible to resolve partly using preparative HPLC (Supplementary Fig. 97). **A.** ^{1}H NMR (401.0 MHz) of isolated epimer A and B (absolute chirality was not assigned). The purity of obtained epimer A (first eluting on HPLC) was found to be superior to that of epimer B and was therefore used for the epimerization kinetic experiment. **B.** Epimerization from pure epimer A was studied using continuous ^{1}H NMR (MOPS/NaOD buffer in D_2O of formal pH 7.4, 37 °C). Due to the absence of overlap-free signals between the epimers, data were obtained by deconvolution of peak area of one of the hydrogen atoms on bridging CH_2 moiety (between pyridine and triazole ring, 5.5 ppm) from each time point. A monoexponential fit of data provided epimerization half-life of ~2.4 h under chosen condition. No cleavage of Boc group was observed during the whole experiment (previous attempts with non-buffered solutions in D_2O failed due to concomitant cleavage of Boc moiety). **C.** ^{1}H NMR (400.1 MHz) of racemic mixtures from isolated epimers A and B (concurrently incubated under identical conditions on heated magnetic stirrer) after the conclusion of epimerization study. Source data available in Supplementary Data 2.

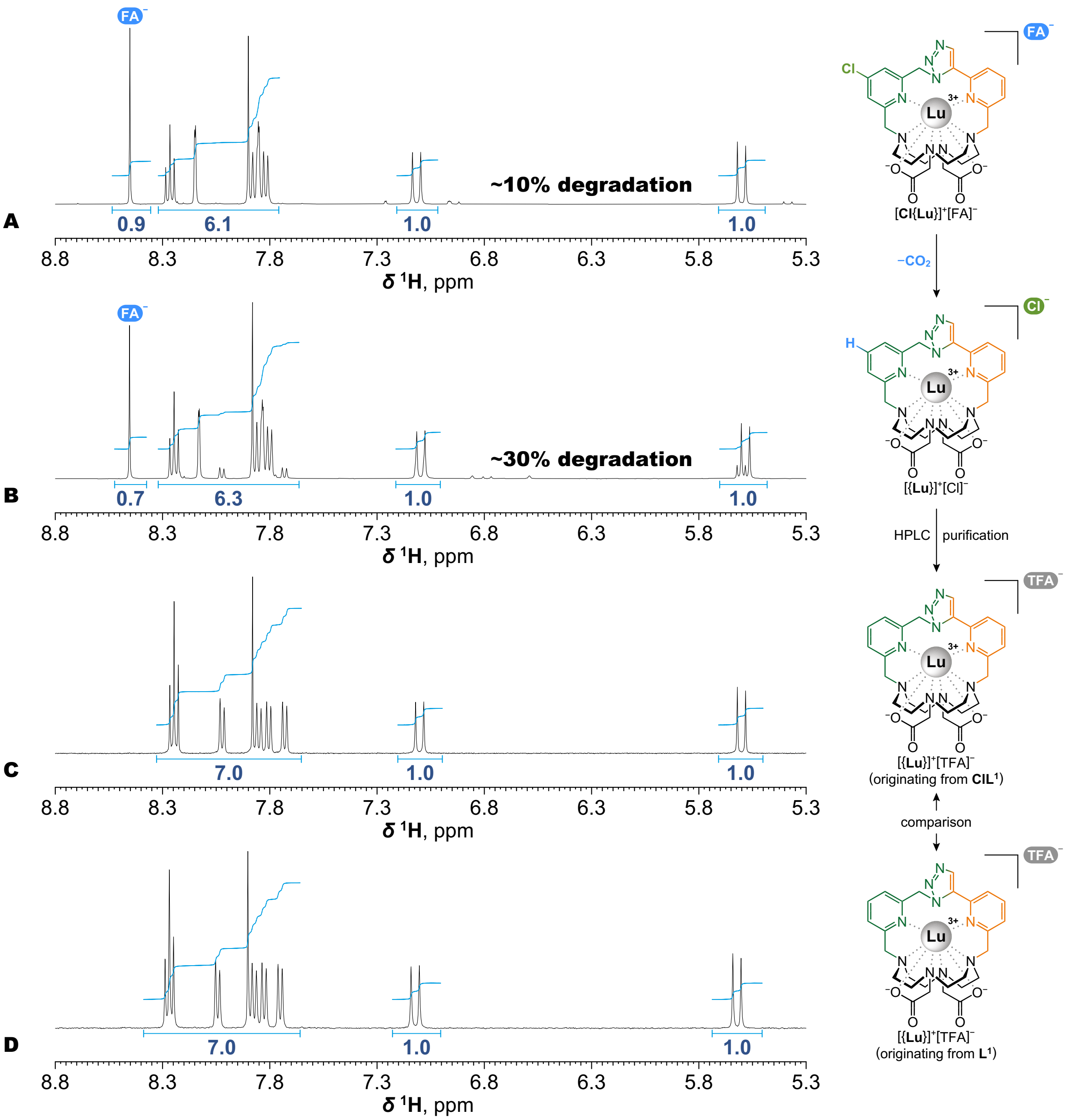

Supplementary Fig. 23. Degradation of Cl{Lu} when isolated as FA salt. The choice of the counterion (FA or TFA) to the positively charged ClickZip chelate was found to be generally irrelevant to the stability of the compound, except in the case of **Cl**{**Lu**} derivative. **A.** ^{1}H NMR (400.1 MHz) of isolated FA salt, where ~10% of the compound already degraded during lyophilization to a chloride salt of {**Lu**} with a release of CO_2 (along with small extent of hydrolysis of Cl to OH). **B.** ^{1}H NMR (401.0 MHz) sample of the isolated solid material after standing few days in a freezer (~30% degradation with the corresponding decrease of FA signal and increase of aromatic signals). Degradation can be slowed by making diluted aqueous solution, nevertheless the reaction could not be entirely prevented. However, when **Cl**{**Lu**} was isolated as TFA salt, no degradation occurred even after 2 years of storage at −18 °C. **C.** ^{1}H NMR (401.0 MHz) sample of the formed {**Lu**} byproduct. **D.** Comparison of ^{1}H NMR (400.1 MHz) from a sample of {**Lu**} formed by click zip from ligand **L**^{1}.

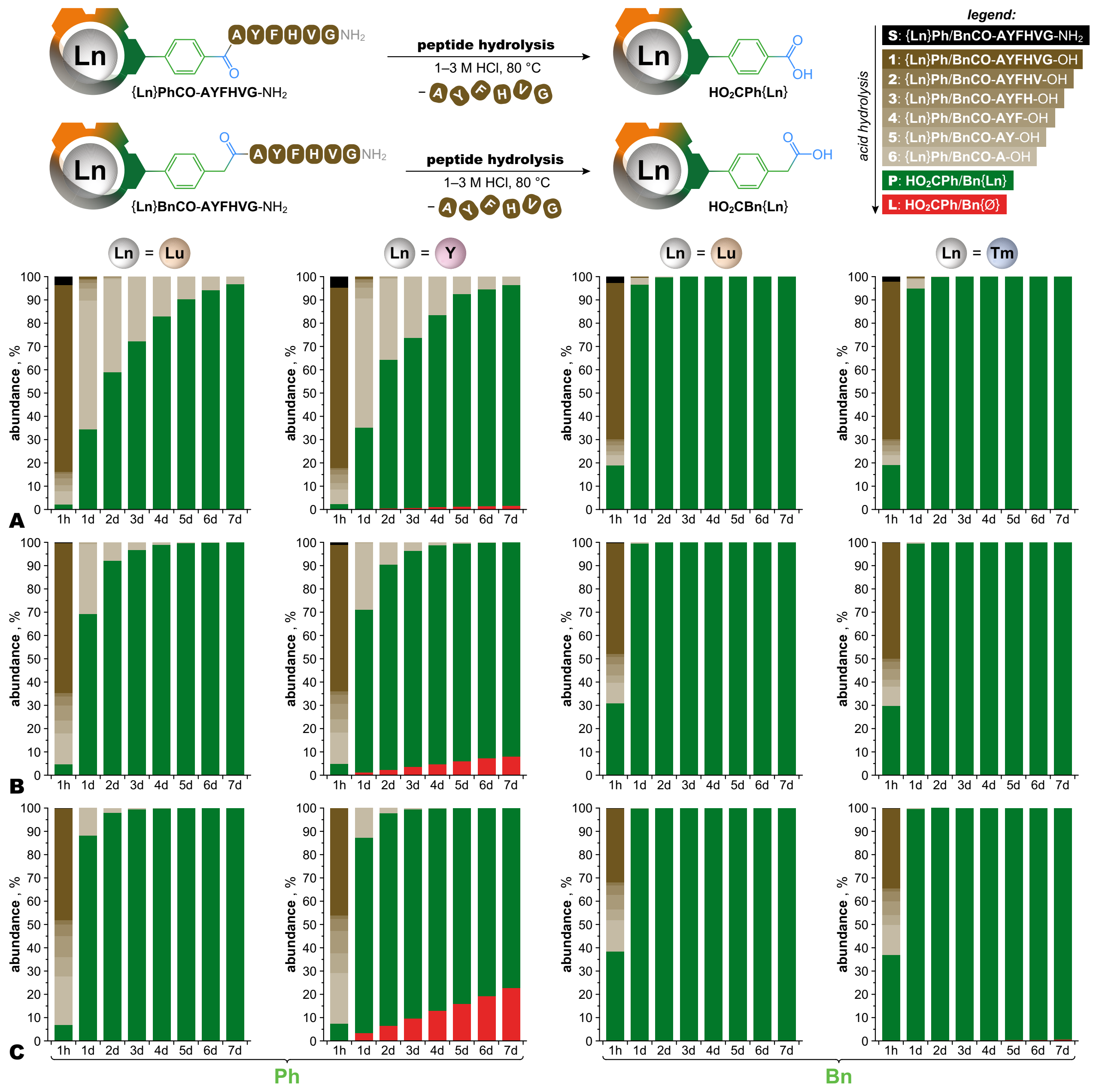

Supplementary Fig. 24. Acid hydrolysis of model hexapeptides with ClickZip tags. Acidic hydrolysis of ClickZip conjugates (chelates of monoisotopic or nearly monoisotopic Tm^{III}, Lu^{III} and Y^{III} were chosen purposedly) with model hexapeptide AYFHVG-NH_2. Cleavage of the amide linkage from the ClickZip chelate to peptide (green bars) is faster for aliphatic (BnCO) compared to aromatic (PhCO) carbonyl. The hydrolysis is expectedly faster with increasing concentration of the acid. None or negligible release of the Ln^{III} ion (red bars) was observed for Lu^{III} and Tm^{III} ClickZip chelates making them fully orthogonal with complete peptide hydrolysis under all examined conditions. In contrast, noticeable release of Y^{III} ions from the chelate in {**Y**}**PhCO-AYFHVG**-NH_2 occurs under the same conditions. These results are consistent with the acid-assisted dechelation data from **Ph**{**Ln**} chelate (Fig 2A). ***Conditions:*** 0.5 mM {**Ln**}**PhCO-AYFHVG**-NH_2 (Ln = Lu^{III}, Y^{III}) or {**Ln**}**BnCO-AYFHVG**-NH_2 (Ln = Lu^{III}, Tm^{III}) in HCl (1–3 M) at 80 °C. ***Analysis:*** HPLC with UV detection at λ = 285 nm (H_2O–MeCN gradient with 0.1% FA additive). All ClickZip-containing intermediates (brown bars) were distinguished and identified due to good UV absorption and MS ionization. **A.** 1 M HCl at 80 °C. **B.** 2 M HCl at 80 °C. **C.** 3 M HCl at 80 °C. Source data available in Supplementary Data 2.

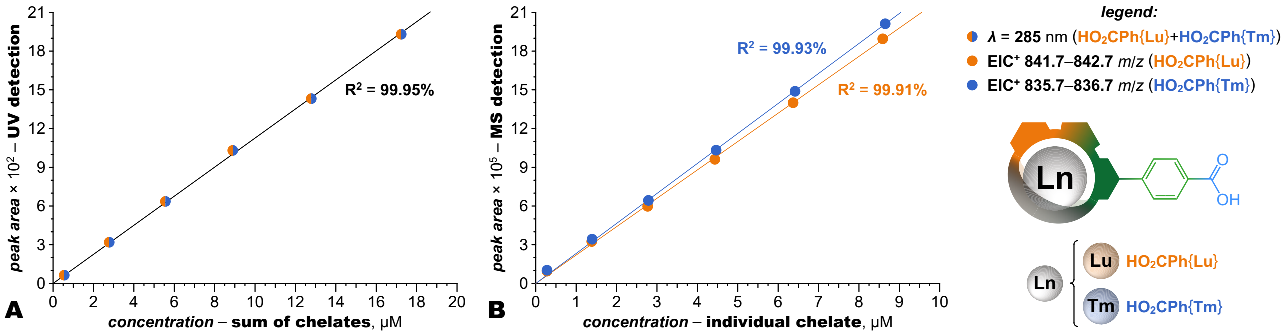

Supplementary Fig. 25. LC-MS (single quadrupole) quantification of HO$_2$CPh{Ln} tags. The detection and quantification is based on a combination of UV absorbance and relative abundance of molecular ion in MS. It takes advantage of the isostructurality of ClickZip chelates that, within the range Sm^{III}–Lu^{III}, leads to their identical retention times and UV absorption profiles (Supplementary Fig. 21). For several ClickZip tags present at the same time, the UV peak provides their collective quantification. The total quantity can be divided into individual tags based on the relative abundance of the corresponding molecular peaks in the MS spectrum. ***Analysis:*** HPLC with combined UV detection at $\lambda = 285$ nm and MS detection (H_2O–MeCN gradient with 0.1% FA additive). **A.** Calibration curve of nearly equimolar mixtures of **HO$_2$CPh{Lu}** and **HO$_2$CPh{Tm}** of various absolute concentration (obtained from aqueous stock solutions of known concentration determined by ICP-OES) showing linear dependence of UV peak area on concentration. **B.** Independent quantification of both **HO$_2$CPh{Lu}** and **HO$_2$CPh{Tm}** from their nearly equimolar mixtures of different absolute concentrations based on the difference of their exact masses (the coincidence of retention time ensures equal ionization for both compounds). This demonstrates that ClickZip tags can be quantified even with a commonplace LC-MS system with a single quadrupole mass spectrometer within the given concentration range (above ~25 μM the relationship becomes non-linear). ***Detection:*** EIC$^+$ 841.7–842.7 *m*/*z* and 835.7–836.7 *m*/*z* using ESI$^+$ ionization. Source data available in Supplementary Data 2.

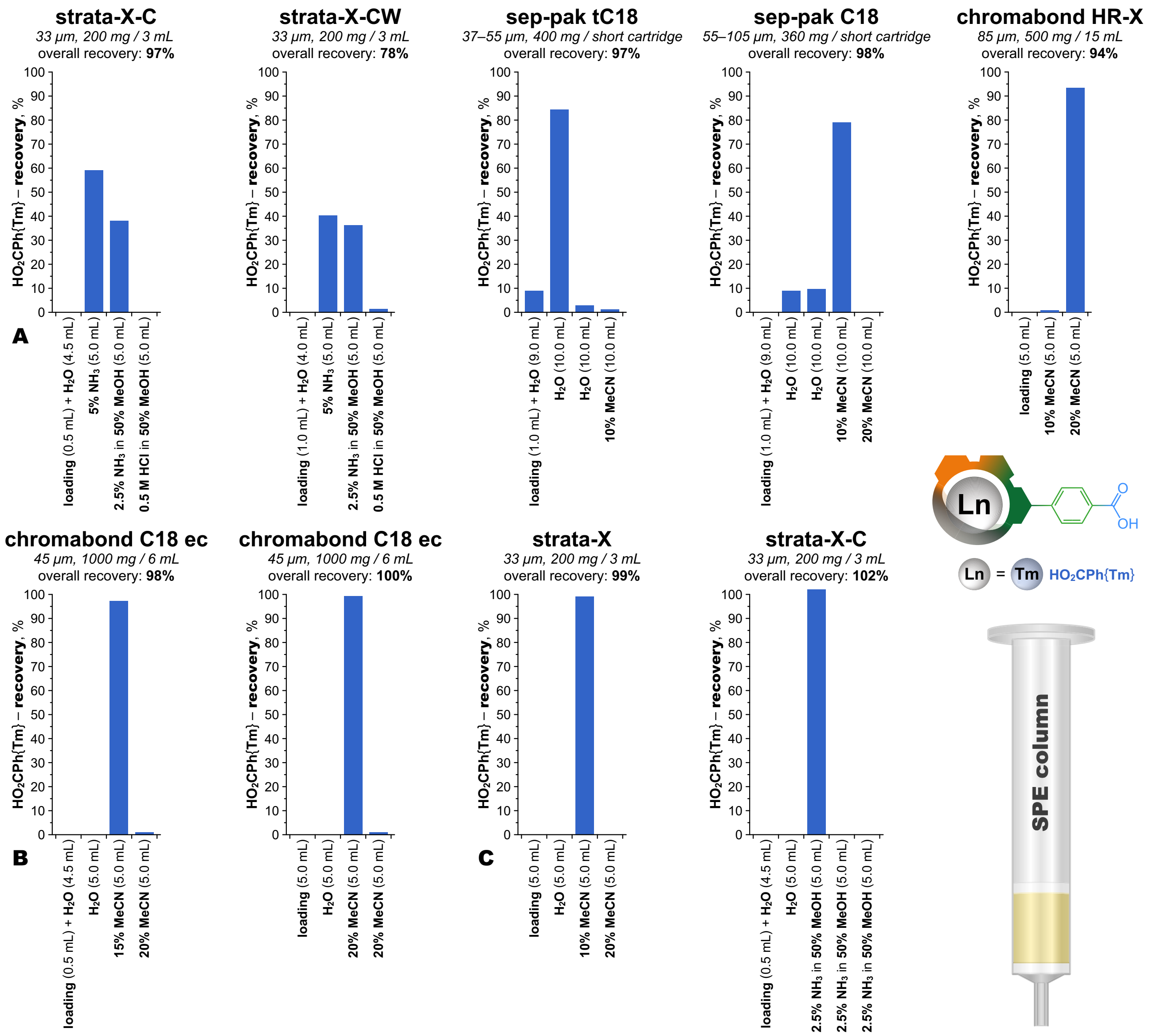

Supplementary Fig. 26. Solid-phase extraction of HO_2CPh\{Ln\} tags. Simulation of **HO_2CPh\{Tm\}** pre-purification from complicated organic matrices using commercial solid-phase extraction (SPE) columns. The amount of analyte in each fraction was compared to the amount that was loaded to quantify the recovery (overall recovery being the sum of all fractions). In some cases, different loading volumes (but with the same total amount of analyte) were also tested. ***Conditions:*** Dry SPE columns were first conditioned with H_2O−MeCN solutions (100%, 80%, 60%, 40%, 20%, 10%, 5% MeCN, 5 mL each) followed by washing with H_2O (10 mL). Total amount of 5 nmol of **HO_2CPh\{Tm\}** in 500 mM MOPS/NaOH buffer (pH 7.0) was then loaded in 0.5 mL, 1.0 mL or 5.0 mL volume (5 nmol of **HO_2CPh\{Tm\}** in unbuffered H_2O was used in the case of strata X-C and strata X-CW). ***Analysis:*** HPLC with UV detection at $\lambda = 285$ nm (H_2O–MeCN gradient with 0.1% FA additive). **A.** Examples of poor performance due to elution of **HO_2CPh\{Tm\}** in multiple fractions and/or its incomplete recovery. **B.** Good performance with nearly complete recovery of **HO_2CPh\{Tm\}** in a single fraction. **C.** Optimal performance on strata-X and strata-X-C with complete recovery of **HO_2CPh\{Tm\}** in a single fraction. Source data available in Supplementary Data 2.

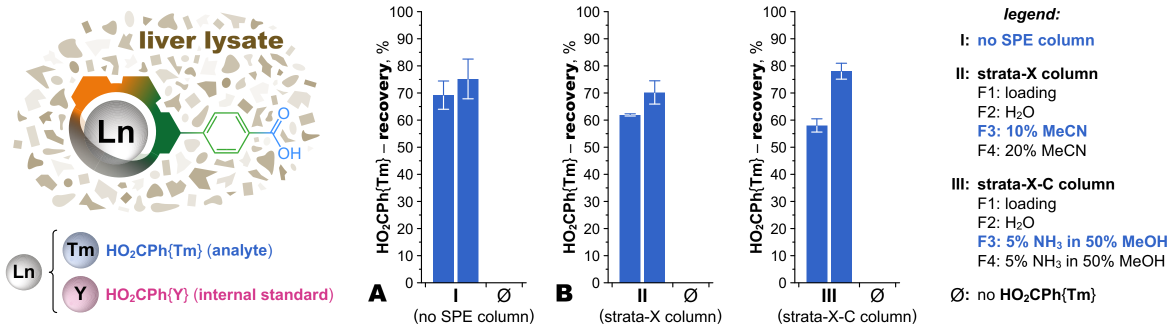

Supplementary Fig. 27. Quantification of HO_2CPh{Ln} using LC-MS/MS. Comparison of **HO_2CPh{Tm}** operation losses for mouse liver lysate (spiked with **HO_2CPh{Tm}** before the HCl hydrolysis) when processed with or without SPE columns (each experiment was performed in duplicates that are displayed as individual bars). Obtained data are plotted as **HO_2CPh{Tm}** recovery against known input amount, standard deviation represents the average of three measurements. The elution scheme for the two tested SPE columns (approach **II** and **III**) is shown on the right: the analysis was performed only on the third fraction (labelled F3, blue). The matrix effect was compensated by using **HO_2CPh{Y}** as an internal standard. Each approach (**I–III**) was accompanied with a single blank experiment (with mouse liver, but without **HO_2CPh{Tm}** tag, labelled Ø). ***Methodology:*** Six blank mouse liver samples (from C57BL/6J mice, ~100 mg in each sample) were suspended in HCl (6 M, 1.0 mL) in a glass vial (4 mL) and spiked with **HO_2CPh{Tm}** (2.0 pmol total, from an aqueous stock solution of known concentration from ICP-OES). The resulting set of solutions was capped with a septum stopper and stirred at 80 °C for 3 days. Samples were then divided into three sets (each in duplicate). Processing of the first pair of samples is identical to the description given in Materials and Methods. Samples from the second and third pairs were processed analogously, but were additionally purified with SPE columns (strata-X and strata-X-C, resulting lysate volume 200 μL). Details for the LC-MS/MS method are given in Supplementary Fig. 28. **A.** The processing of liver lysate (from the original tissue weight of ~100 mg) without the use of SPE columns (approach **I**) and its gradual concentration to a final volume of 400 μL gives an MS signal recovery of **HO_2CPh{Tm}** tag ~70%. **B.** The **HO_2CPh{Tm}** recoveries obtained using the SPE columns (approach **II** and **III**) show that they provide no additional benefit. Thus, a procedure without SPE columns was used to process liver samples from mice injected with prolactin-releasing peptide conjugates (approach **I**). The data are presented as the mean ± SD ($N = 3$ measurements/technical replicates). Source data available in Supplementary Data 2.

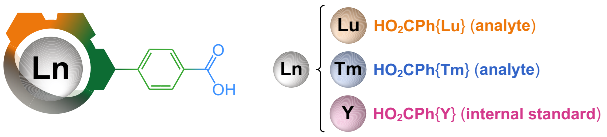

LC-MS/MS

dwell time: 50 msec
entrance potential: 10 V
ion source temperature: 350°C
ion spray voltage: 5.5 kV
curtain gas: 40 psi
GS1/GS2 gas: 50 psi

multiple reaction monitoring (MRM) transitions

identity	*role*	$[M]^+$ *mass*	*declustering potential, V*	*product ions*	*ion purpose*	*collision energy, V*	*cell exit potential, V*
**HO_2CPh\{Lu\}**	analyte	842.2	251	813.9	quantifier	47	34
				786.1	qualifier	49	30
**HO_2CPh\{Tm\}**	analyte	836.3	130	808.1	quantifier	45	36
				780.2	qualifier	47	36
**HO_2CPh\{Y\}**	internal standard	756.2	181	728.2	quantifier	41	34
				700.2	qualifier	43	32

Supplementary Fig. 28. LC-MS/MS parameters for quantification of HO_2CPh\{Ln\} tags. The **HO_2CPh\{Lu\}** and **HO_2CPh\{Tm\}** complexes, along with the corresponding internal standard **HO_2CPh\{Y\}**, were separated and detected using a liquid chromatograph Exion LC AD with tandem mass spectrometer QTrap 6500+ (both *Sciex*), equipped with a separation column Kinetex 1.7 µm F5, 100 × 2.1 mm, as well as a guard column 1.7 µm F5, 2.1 mm (both *Phenomenex*). Mobile phase A: 5 mM AF with 0.1% FA in deionized H_2O. Mobile phase B: 5 mM AF with 0.1% FA in MeOH. The separation was carried out by using the following gradient: 0 – 1.5 min of 10% B, 1.5 – 6 min of 95% B, 6 – 11 min of 95% B, 11 – 11.1 min of 10% B, and 11.1 – 16 min of 10% B with a flow rate of 0.2 mL min^{-1}. Analytes were monitored on LC-MS/MS through their specific multiple reaction monitoring (MRM) transitions by utilizing electrospray ionization in a positive mode (ESI^+). Parameters of the measurements are listed above. Additionally, the injection volume was set to 10 µL, and the samples in the autosampler were maintained at 5 °C.

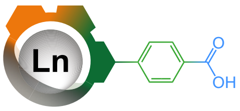

Ln: Lu HO₂CPh{Lu} (analyte); Tm HO₂CPh{Tm} (analyte); Y HO₂CPh{Y} (internal standard)

LC-MS/MS

$$\mathrm{LOD} = \frac{s_{yx}}{b_1} \times t_{1-\alpha;(n-2)} \times \sqrt{1 + \frac{1}{n} + \frac{c_{av}^2}{\sum_n (c_i - c_{av})^2}} \qquad \mathrm{LOQ} = \mathrm{LOD} \times 3$$

A

raw data

c, nM	HO₂CPh{Lu}	HO₂CPh{Y}	***ratio*** (Lu/Y)
1.0	3.10×10^3	2.18×10^6	1.42×10^{-3}
1.0	4.13×10^3	2.33×10^6	1.85×10^{-3}
1.0	3.17×10^3	2.20×10^6	1.44×10^{-3}
2.0	6.84×10^3	2.09×10^6	3.27×10^{-3}
2.0	4.96×10^3	2.14×10^6	2.32×10^{-3}
2.0	6.87×10^3	2.11×10^6	3.26×10^{-3}
5.0	1.68×10^4	2.07×10^6	8.12×10^{-3}
5.0	1.73×10^4	1.96×10^6	8.83×10^{-3}
5.0	1.64×10^4	2.06×10^6	7.96×10^{-3}

parameter	***value***
b_1 (slope)	0.00171
s_{yx} (relative std. deviation)	0.000433
n (number of points)	9
c_{av} (concentration average)	2.67
$t_{1-\alpha;(n-2)}$ (critical t-test value)	1.89
$\sum_n (c_i - c_{av})^2$	26

Ln = Lu HO₂CPh{Lu}

limit of detection (LOD): 0.57 nM

limit of quantification (LOQ): 1.70 nM

B

raw data

c, nM	HO₂CPh{Tm}	HO₂CPh{Y}	***ratio*** (Tm/Y)
1.0	2.14×10^4	2.18×10^6	9.82×10^{-3}
1.0	2.02×10^4	2.33×10^6	9.06×10^{-3}
1.0	1.74×10^4	2.20×10^6	7.91×10^{-3}
2.0	3.90×10^4	2.09×10^6	1.87×10^{-2}
2.0	3.90×10^4	2.14×10^6	1.82×10^{-2}
2.0	3.66×10^4	2.11×10^6	1.73×10^{-2}
5.0	9.22×10^4	2.07×10^6	4.45×10^{-2}
5.0	8.72×10^4	1.96×10^6	4.45×10^{-2}
5.0	9.74×10^4	2.06×10^6	4.73×10^{-2}

parameter	***value***
b_1 (slope)	0.00913
s_{yx} (relative std. deviation)	0.00106
n (number of points)	9
c_{av} (concentration average)	2.67
$t_{1-\alpha;(n-2)}$ (critical t-test value)	1.89
$\sum_n (c_i - c_{av})^2$	26

Ln = Tm HO₂CPh{Tm}

limit of detection (LOD): 0.26 nM

limit of quantification (LOQ): 0.78 nM

Supplementary Fig. 29. LOD and LOQ of HO_2CPh\{Ln\} on LC-MS/MS. Determination of LOD (limit of detection) and LOQ (limit of quantification) of **HO_2CPh\{Lu\}** and **HO_2CPh\{Tm\}** in presence of blank matrix (liver lysate) using **HO_2CPh\{Y\}** as an internal standard. The raw data were obtained by integration of peak area of corresponding quantifier signal (see materials and methods section). More details about the calculations and about other validation parameters (test of linearity, repeatability and accuracy) can be found in Supplementary Data 1 (excel spreadsheet). **A.** LOD and LOQ of **HO_2CPh\{Lu\}** **B.** LOD and LOQ of **HO_2CPh\{Tm\}**.

Y(NO_3)$_3$ (internal standard) Ln { Lu Lu(NO_3)$_3$ (analyte); Yb Yb(NO_3)$_3$ (analyte); Tm Tm(NO_3)$_3$ (analyte) }

ICP-MS

$$\mathrm{LOD} = \frac{s_{yx}}{b_1} \times t_{1-\alpha;(n-2)} \times \sqrt{1 + \frac{1}{n} + \frac{c_{av}^2}{\sum_n (c_i - c_{av})^2}} \qquad \mathrm{LOQ} = \mathrm{LOD} \times 3$$

A

raw data

c, ng × L^{-1}	Lu(NO_3)$_3$, cps	Y(NO_3)$_3$, cps	*ratio* (Lu/Y)
0.0	0.00 × 10^0	3.75 × 10^4	0.00 × 10^0
0.0	0.00 × 10^0	3.92 × 10^4	0.00 × 10^0
0.0	8.00 × 10^0	3.90 × 10^4	2.05 × 10^{-5}
0.0	0.00 × 10^0	3.89 × 10^4	0.00 × 10^0
0.0	0.00 × 10^0	3.82 × 10^4	0.00 × 10^0
0.5	2.80 × 10^1	3.90 × 10^4	7.18 × 10^{-4}
0.5	2.48 × 10^1	3.90 × 10^4	6.36 × 10^{-4}
0.5	3.36 × 10^1	3.90 × 10^4	8.61 × 10^{-4}
0.5	2.88 × 10^1	3.90 × 10^4	7.38 × 10^{-4}
0.5	2.88 × 10^1	3.86 × 10^4	7.46 × 10^{-4}
1.0	4.08 × 10^1	3.74 × 10^4	1.09 × 10^{-3}
1.0	4.88 × 10^1	3.85 × 10^4	1.27 × 10^{-3}
1.0	4.80 × 10^1	3.73 × 10^4	1.29 × 10^{-3}
1.0	5.68 × 10^1	3.93 × 10^4	1.45 × 10^{-3}
1.0	5.44 × 10^1	3.83 × 10^4	1.42 × 10^{-3}

parameter	*value*
b_1 (slope)	0.00130
s_{yx} (relative std. deviation)	0.000101
n (number of points)	15
c_{av} (concentration average)	0.50
$t_{1-\alpha;(n-2)}$ (critical t-test value)	1.77
$\sum_n (c_i - c_{av})^2$	2.52

Ln = Lu Lu(NO_3)$_3$

limit of detection (LOD): 0.15 ng × L^{-1} (**0.86** pM)
limit of quantification (LOQ): 0.45 ng × L^{-1} (**2.57** pM)

B

raw data

c, ng × L^{-1}	Yb(NO_3)$_3$, cps	Y(NO_3)$_3$, cps	*ratio* (Yb/Y)
0.0	3.12 × 10^0	3.75 × 10^4	8.30 × 10^{-5}
0.0	7.11 × 10^{-1}	3.92 × 10^4	1.81 × 10^{-5}
0.0	3.87 × 10^0	3.90 × 10^4	9.94 × 10^{-5}
0.0	−1.08 × 10^{-1}	3.89 × 10^4	−2.78 × 10^{-6}
0.0	3.07 × 10^0	3.82 × 10^4	8.04 × 10^{-5}
0.5	1.35 × 10^1	3.90 × 10^4	3.46 × 10^{-4}
0.5	9.49 × 10^0	3.90 × 10^4	2.43 × 10^{-4}
0.5	1.19 × 10^1	3.90 × 10^4	3.05 × 10^{-4}
0.5	9.49 × 10^0	3.90 × 10^4	2.43 × 10^{-4}
0.5	1.03 × 10^1	3.86 × 10^4	2.68 × 10^{-4}
1.0	1.68 × 10^1	3.74 × 10^4	4.48 × 10^{-4}
1.0	2.31 × 10^1	3.85 × 10^4	6.01 × 10^{-4}
1.0	1.68 × 10^1	3.73 × 10^4	4.49 × 10^{-4}
1.0	1.43 × 10^1	3.93 × 10^4	3.65 × 10^{-4}
1.0	1.67 × 10^1	3.83 × 10^4	4.37 × 10^{-4}

parameter	*value*
b_1 (slope)	0.00040
s_{yx} (relative std. deviation)	0.000060
n (number of points)	15
c_{av} (concentration average)	0.50
$t_{1-\alpha;(n-2)}$ (critical t-test value)	1.77
$\sum_n (c_i - c_{av})^2$	2.52

Ln = Yb Yb(NO_3)$_3$

limit of detection (LOD): 0.29 ng × L^{-1} (**1.68** pM)
limit of quantification (LOQ): 0.86 ng × L^{-1} (**5.03** pM)

C

raw data

c, ng × L^{-1}	Tm(NO_3)$_3$, cps	Y(NO_3)$_3$, cps	*ratio* (Tm/Y)
0.0	8.00 × 10^{-1}	3.75 × 10^4	2.13 × 10^{-5}
0.0	0.00 × 10^0	3.92 × 10^4	0.00 × 10^0
0.0	8.00 × 10^{-1}	3.90 × 10^4	2.05 × 10^{-5}
0.0	0.00 × 10^0	3.89 × 10^4	0.00 × 10^0
0.0	0.00 × 10^0	3.82 × 10^4	0.00 × 10^0
0.5	3.28 × 10^1	3.90 × 10^4	8.41 × 10^{-4}
0.5	3.20 × 10^1	3.90 × 10^4	8.20 × 10^{-4}
0.5	2.48 × 10^1	3.90 × 10^4	6.35 × 10^{-4}
0.5	3.28 × 10^1	3.90 × 10^4	8.40 × 10^{-4}
0.5	2.40 × 10^1	3.86 × 10^4	6.22 × 10^{-4}
1.0	5.28 × 10^1	3.74 × 10^4	1.41 × 10^{-3}
1.0	5.84 × 10^1	3.85 × 10^4	1.52 × 10^{-3}
1.0	5.20 × 10^1	3.73 × 10^4	1.39 × 10^{-3}
1.0	5.44 × 10^1	3.93 × 10^4	1.39 × 10^{-3}
1.0	6.40 × 10^1	3.83 × 10^4	1.67 × 10^{-3}

parameter	*value*
b_1 (slope)	0.00147
s_{yx} (relative std. deviation)	0.000092
n (number of points)	15
c_{av} (concentration average)	0.50
$t_{1-\alpha;(n-2)}$ (critical t-test value)	1.77
$\sum_n (c_i - c_{av})^2$	2.52

Ln = Tm Tm(NO_3)$_3$

limit of detection (LOD): 0.12 ng × L^{-1} (**0.71** pM)
limit of quantification (LOQ): 0.36 ng × L^{-1} (**2.13** pM)

Supplementary Fig. 30. LOD and LOQ of HO_2CPh{Ln} on ICP-MS. Due to the complete atomization of **HO_2CPh{Ln}** in plasma, determination of LOD (limit of detection) and LOQ (limit of quantification) was performed with certified standards Lu(NO_3)$_3$, Yb(NO_3)$_3$ and Tm(NO_3)$_3$ instead (with Y(NO_3)$_3$ as an internal standard). More details about the calculations and about other validation parameters (test of linearity, repeatability and accuracy) can be found in Supplementary Data 1 (excel spreadsheet). **A.** Indirect determination of LOD and LOQ for **HO_2CPh{Lu}** **B.** Indirect determination of LOD and LOQ for **HO_2CPh{Yb}**. **C.** Indirect determination of LOD and LOQ for **HO_2CPh{Tm}**.

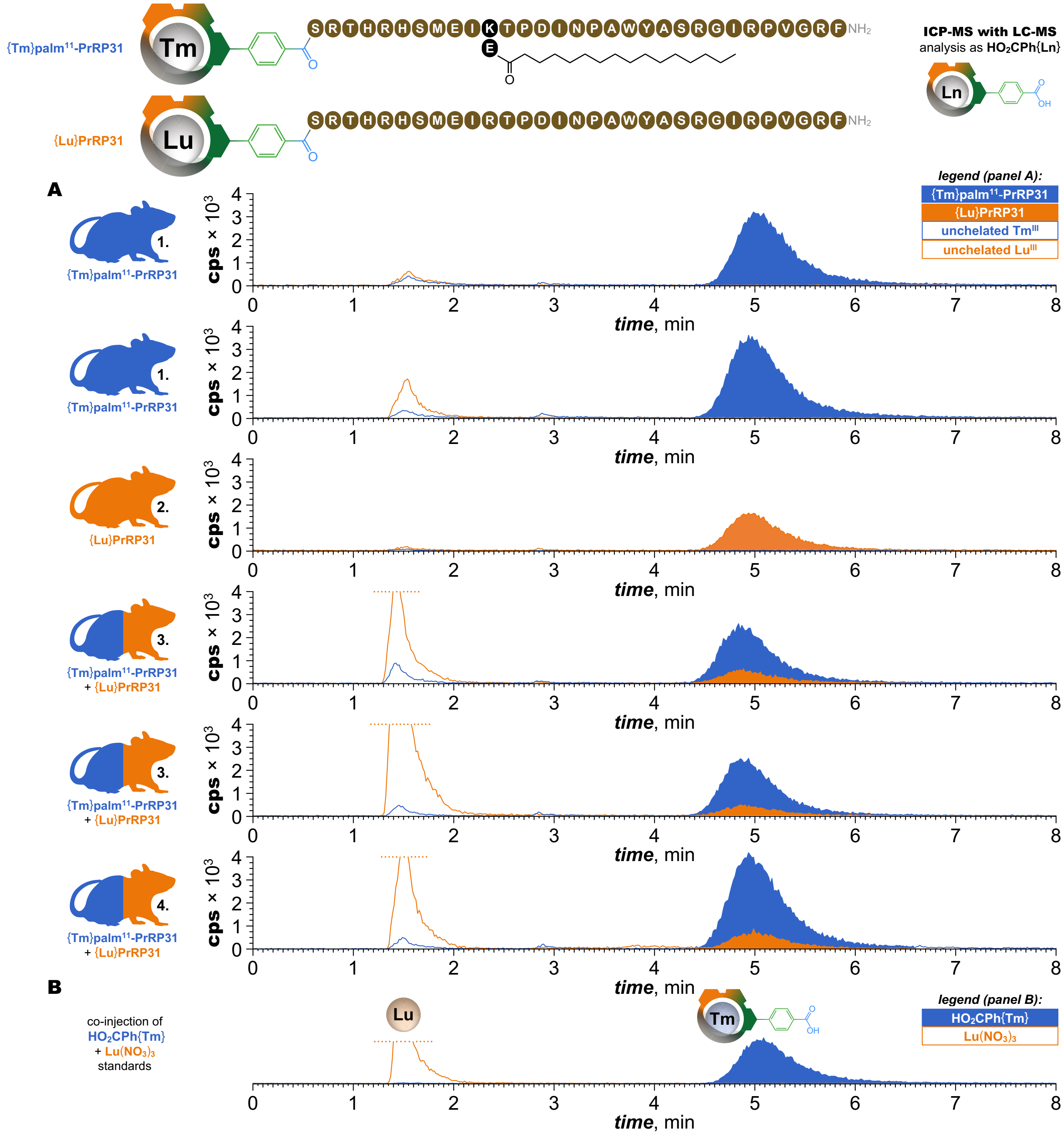

Supplementary Fig. 31. Speciation analysis in mouse liver by LC-ICP-MS. Chromatograms obtained from mouse liver samples hydrolyzed in HCl with LC speciation prior to ICP-MS detection. The chromatograms show overlayed traces of simultaneously detected Tm a Lu, with clear speciation into peaks of **HO_2CPh**{**Ln**} tags (at ~5 min) and unchelated Ln^{III} ions (at ~1.5 min). Concentrations determined from integration of the **HO_2CPh**{**Ln**} peaks were used to quantify uptake of {**Tm**}**palm^{11}-PrRP31** and/or {**Lu**}**PrRP31** in mouse liver (Fig. 8E). ***Conditions:*** isocratic elution using 5% *1*,*2*-hexanediol in H_2O with 1% FA additive (1 mL min^{-1} flow rate). **A.** Chromatograms of individual liver samples accompanied by the injection scheme for a given mouse (two liver samples were taken for mice 1 and 3). Extremely high stability of ClickZip tags allowe d their separation and quantification from presence of non-negligible amounts of unchelated Ln^{III} ions from unexpected enviromental contamination, achieving excellent agreement with LC-MS/MS analysis from Fig. 8C. **B.** Chromatogram of co-injected **HO_2CPh**{**Tm**} and $Lu(NO_3)_3$ standards to determine retention of **HO_2CPh**{**Ln**} and unchelated Ln^{III} ions.

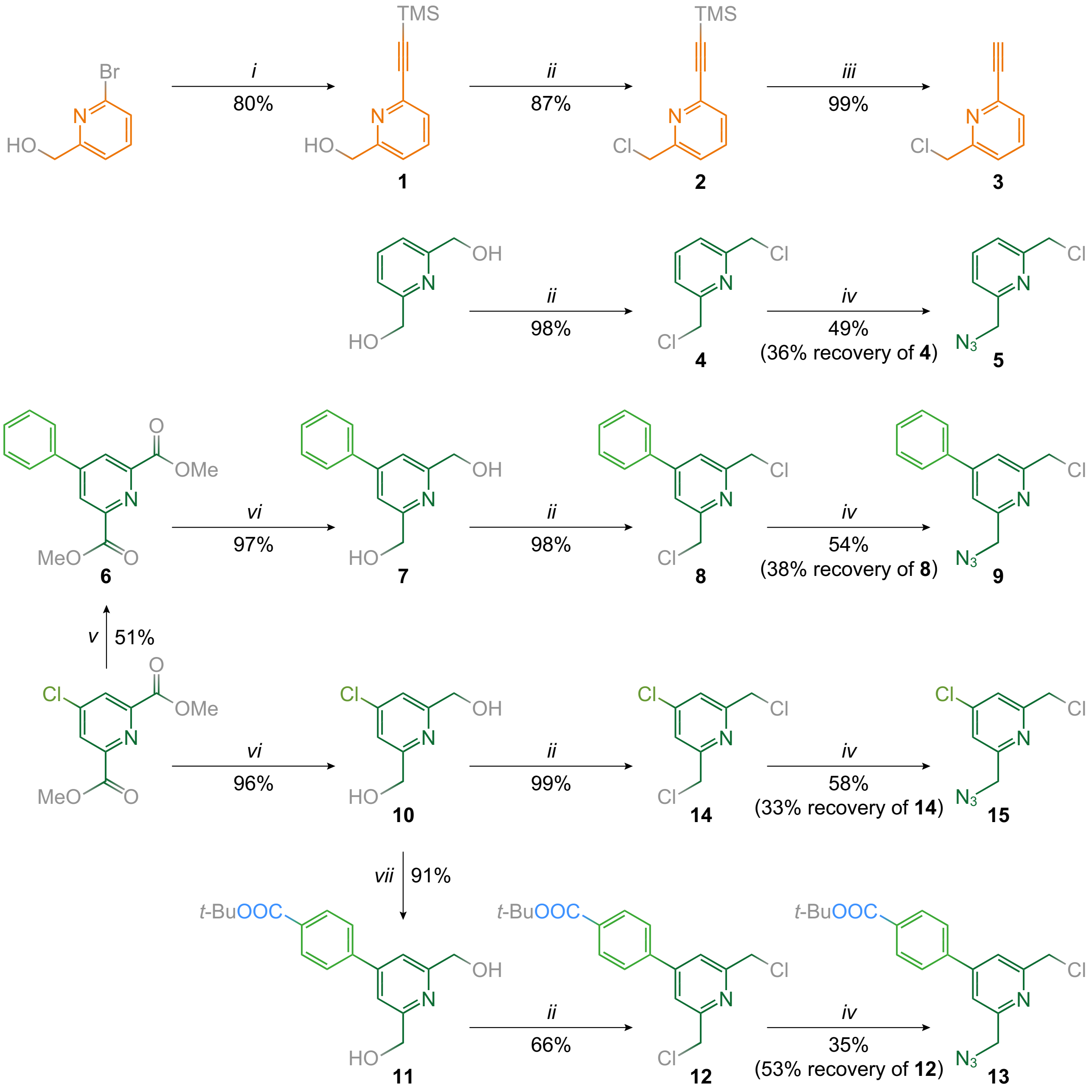

Supplementary Fig. 32. Synthesis of alkyne or azide bearing pyridine intermediates. The pyridine alkylating agents were obtained by short reaction sequences (2−4 steps from readily available and inexpensive precursors) using well estabilished and generally high-yielding chemistry. Lower yields were obtained only in the case of intermediate **6** (although balanced by simple isolation of **6** by precipitating from reaction mixture) and in the last desymmetrization steps of azide bearing pyridines (**5**, **9**, **13** and **15**). There, the inevitable statistical mixture of Cl/Cl, Cl/N_3 and N_3/N_3 species was obtained, but the formation of undesired N_3/N_3 was greatly surpressed using moderate excess of Cl/Cl over NaN_3, which in turn was recovered during workup and reused for a next round of desymmetrization. ***Conditions:*** (*i*) ethynyltrimethylsilane, CuI (cat.), [Pd(PPh_3)$_2$$Cl_2$] (cat.), TEA, THF, RT; (*ii*) $SOCl_2$, DCM, RT; (*iii*) KF, MeCN, H_2O, RT; (*iv*) NaN_3, K_2CO_3, MeCN, 50–80 °C; (*v*) Ph–B(OH)$_2$, XPhos Pd G2 (cat.), Cs_2CO_3, DMF, 80 °C; (*vi*) $NaBH_4$, MeOH, THF, RT; (*vii*) *t*-BuO_2C–*p*-Ph–B(OH)$_2$, XPhos Pd G2 (cat.), K_3PO_4, *1*,*4*-dioxane, H_2O, 80°C. Yields refer to isolated compounds.

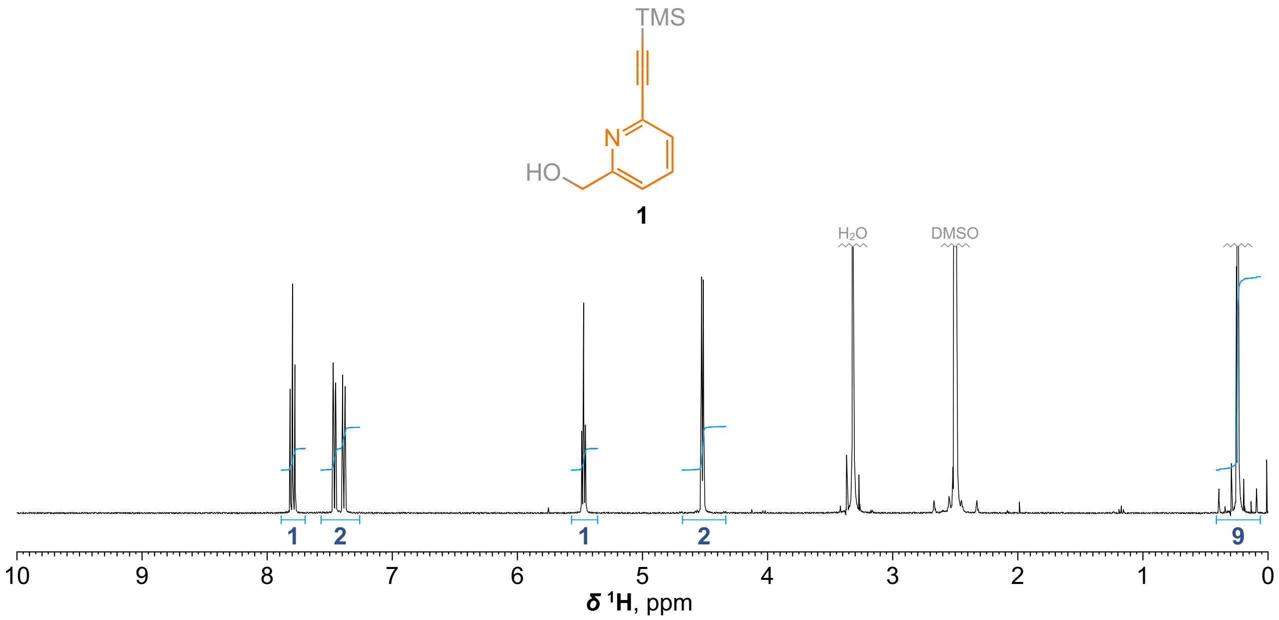

Supplementary Fig. 33. Synthesis and characterization of intermediate 1. Pear-shaped glass flask (50 mL) was charged with (*6*-bromopyridin-*2*-yl)methanol (2.47 g; 13.1 mmol; 1.0 equiv.), CuI (149 mg; 782 µmol; 6.0 mol%), [Pd(PPh_3)$_2$$Cl_2$] (225 mg; 321 µmol; 2.4 mol%) and magnetic stirrer and three times secured with argon. Under constant flow of argon was then added dry THF (25 mL) through septum followed by addition of ethynyltrimethylsilane (2.0 mL; 14.5 mmol; 1.1 equiv.) and TEA (5.5 mL; 39.5 mmol; 3.0 equiv.) after which the mixture changed color to dark brown. The flask was left stirring under septum (but without external argon) for 3 h at RT. The mixture was then transferred to a separatory funnel with EtOAc (150 mL) and H_2O (100 mL). After shaking, the dark colored (and not entirely homogenous) organic layer was separated and the aqueous layer was further extracted with EtOAc (5 × 50 mL). Combined organic layers were filtered through cotton plug and the filtrate was further dried with anhydrous Na_2SO_4, filtered through glass frit (S3) and evaporated to dryness. Resulting dark brown oily residue was purified on flash chromatography (330 g SiO_2, 100% DCM to 10% EtOAc in DCM). Combined fractions with product were evaporated to dryness and further co-evaporated once with DCM. The residue was further dried on high vacuum overnight to give product in the form of free base as dark yellow oil. **Yield:** 2.16 g (80%; 1 step; based on (*6*-bromopyridin-*2*-yl)methanol). **NMR (DMSO-d$_6$):** ^{1}**H** (400.1 MHz) δ_H 0.24 (CH_3, s, 9H); 4.52 (CH_2, d, 2H, $^3J_{HH}$ = 5.8); 5.47 (O*H*, t, 1H, $^3J_{HH}$ = 5.8); 7.39 (C*H*, dd, 1H, $^3J_{HH}$ = 7.7, $^4J_{HH}$ = 1.0); 7.46 (C*H*, dd, 1H, $^3J_{HH}$ = 7.9, $^4J_{HH}$ = 1.0); 7.80 (C*H*, dd, 1H, $^3J_{HH}$ = 7.9, $^3J_{HH}$ = 7.7). ^{13}**C{**^{1}**H}** (100.8 MHz) δ_C −0.3 (CH_3, s); 63.9 (CH_2, s); 93.4 and 104.5 (*C*≡*C*; 2 × s); 120.2 (*C*H, s); 125.4 (*C*H, s); 137.2 (*C*H, s); 140.8 (*C*, s); 162.7 (*C*, s). **ESI-HRMS:** 206.0995 [M+H]$^+$ (theor. [$C_{11}H_{16}N_1O_1Si_1$]$^+$ = 206.0996).

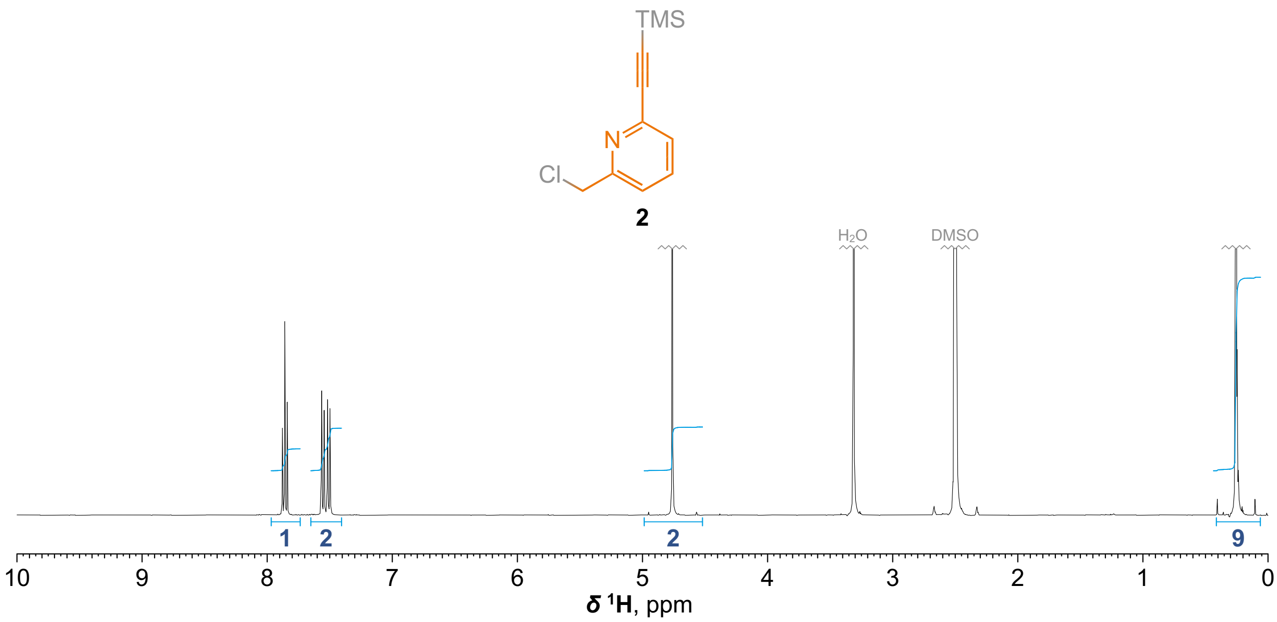

Supplementary Fig. 34. Synthesis and characterization of intermediate 2. In a round-bottom glass flask (100 mL), **1** (2.16 g; 10.5 mmol; 1.0 equiv.) was dissolved in DCM (40 mL). Freshly prepared solution of $SOCl_2$ (1.50 mL; 20.7 mmol; 2.0 equiv.) in DCM (10 mL) was then added and the open flask was stirred for 1 h at RT. Dil. aq. solution of $NaHCO_3$ (50 mL) was then added and resulting biphasic mixture was vigorously stirred for additional 1 h at RT, during which bubbles of gas slowly evolved. Mixture was then transferred to a separatory funnel. After shaking, the bottom phase was separated. Aqueous phase was further extracted with DCM (4 × 50 mL). Combined organic layers were dried with anhydrous Na_2SO_4, filtered through glass frit (S3) and evaporated to dryness. The brown residue (already pure according to ^{1}H NMR) was purified on flash chromatography (220 g SiO_2; 60% P.E. in DCM to 20% P.E. in DCM) to remove the intensively colored impurities. Combined fractions with product were evaporated to dryness and further co-evaporated once with DCM. The residual nearly colorless oil was left in a freezer to solidify. Resulting solid was crushed and further dried on high vacuum overnight to give product in the form of free base as fine white powder. **Yield:** 2.14 g (87%; 1 step; based on **1**). **NMR (DMSO-d$_6$): ^{1}H** (400.1 MHz) δ_H 0.25 (CH_3, s, 9H); 4.76 (CH_2, s, 2H); 7.50 (C*H*, dd, 1H, $^3J_{HH}$ = 7.8, $^4J_{HH}$ = 1.0); 7.55 (C*H*, dd, 1H, $^3J_{HH}$ = 7.9, $^4J_{HH}$ = 1.0); 7.86 (C*H*, dd, 1H, $^3J_{HH}$ = 7.9, $^3J_{HH}$ = 7.8). **^{13}C{^{1}H}** (100.8 MHz) δ_C −0.4 (CH_3, s); 46.3 (CH_2, s); 94.3 and 103.8 (*C*≡*C*; 2 × s); 123.3 (*C*H, s); 126.7 (*C*H, s); 138.1 (*C*H, s); 141.5 (*C*, s); 157.0 (*C*, s). **ESI-HRMS:** 224.0657 [M+H]$^+$ (theor. [$C_{11}H_{15}N_1Cl_1Si_1$]$^+$ = 224.0657).

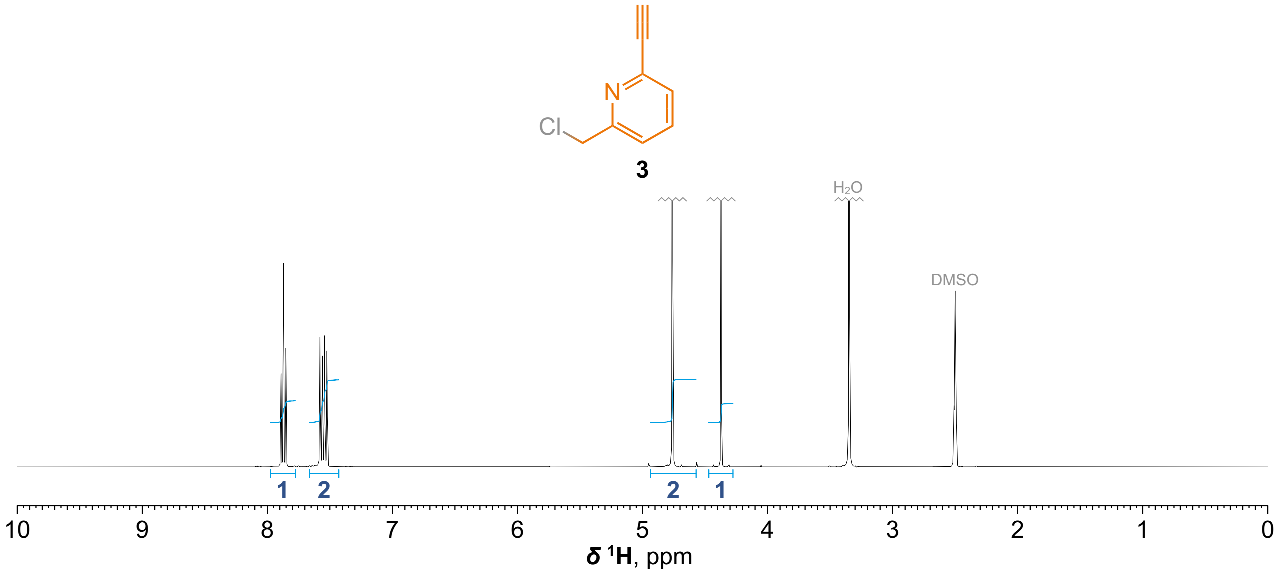

Supplementary Fig. 35. Synthesis and characterization of intermediate 3. In a pear-shaped glass flask (100 mL), **2** (1.05 g; 4.69 mmol; 1.0 equiv.) was dissolved in MeCN (40 mL). Freshly prepared solution of KF (406 mg; 7.0 mmol; 1.5 equiv.) in H_2O (7 mL) was then added and the flask was vigorously stirred for 2 h at RT. The mixture was concentrated to ~10 mL and then diluted with DCM (50 mL) and dil. aq. $NaHCO_3$ (50 mL). The mixture was transferred to a separatory funnel. After shaking, the bottom phase was separated. Aqueous phase was further extracted with DCM (4 × 25 mL). Combined organic layers were dried with anhydrous Na_2SO_4, filtered through glass frit (S3) and evaporated to dryness. The residue was further briefly dried on high vacuum to give product in the form of free base as pale yellow oil. **Yield:** 702 mg (99%; 1 step; based on **2**). **NMR (DMSO-d$_6$):** **^{1}H** (400.1 MHz) δ_H 4.37 (C≡CH, s, 1H); 4.76 (CH_2, s, 2H); 7.53 (CH, dd, 1H, $^3J_{HH}$ = 7.7, $^4J_{HH}$ = 1.0); 7.57 (CH, dd, 1H, $^3J_{HH}$ = 7.8, $^4J_{HH}$ = 1.0); 7.87 (CH, dd, 1H, $^3J_{HH}$ = 7.8, $^3J_{HH}$ = 7.7). **^{13}C{^{1}H}** (100.8 MHz) δ_C 46.3 (CH$_2$, s); 80.6 and 82.6 (C≡CH; 2 × s); 123.4 (CH, s); 126.8 (CH, s); 138.1 (CH, s); 141.3 (C, s); 157.0 (C, s). **ESI-HRMS:** 152.0262 $[M+H]^+$ (theor. $[C_8H_7N_1Cl_1]^+$ = 152.0262).

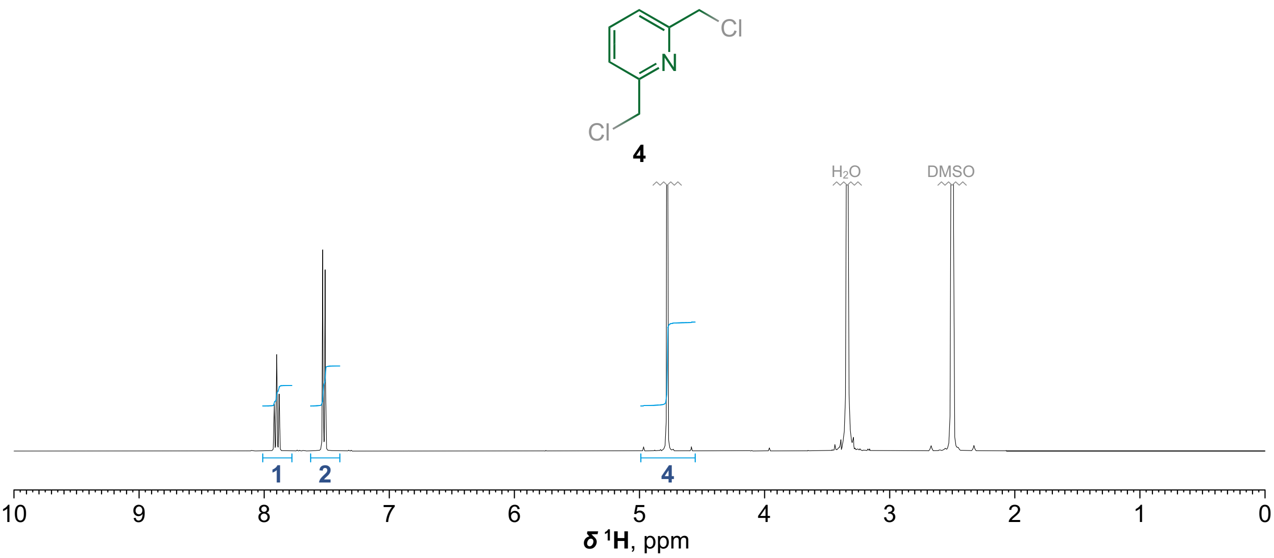

Supplementary Fig. 36. Synthesis and characterization of intermediate 4. Round-bottom glass flask (250 mL) was charged with pyridine-*2*,*6*-diyldimethanol (5.00 g; 35.9 mmol; 1.0 equiv.) and magnetic stirred. The flash was cooled in an ice bath (~5 °C) followed by slow addition of $SOCl_2$ (26 mL; 358 mmol; 10.0 equiv.). After the addition, the mixture was further stirred for 1 h at RT. Mixture was evaporated to dryness followed by addition of DCM (100 mL) to the residue followed by slow addition of dil. aq. $NaHCO_3$ (100 mL). Resulting biphasic mixture was stirred for 1 h at RT, during which bubbles of gas slowly evolved. Mixture was then transferred to a separatory funnel. After shaking, the bottom phase was separated. Aqueous phase was further extracted with DCM (4 × 50 mL). Combined organic layers were dried with anhydrous Na_2SO_4, filtered through glass frit (S3) and evaporated to dryness. The resulting crystalized residue was crushed and further dried on high vacuum overnight to give product in the form of free base as white powder. **Yield:** 6.17 g (98%; 1 step; based on pyridine-*2*,*6*-diyldimethanol). **NMR (DMSO-d$_6$):** ^{1}**H** (400.1 MHz) δ_H 4.78 (CH_2, s, 4H); 7.53 (C*H*, d, 2H, $^3J_{HH}$ = 7.8); 7.91 (C*H*, t, 1H, $^3J_{HH}$ = 7.8). **EI-HRMS:** 174.9947 $[M]^+$ (theor. $[C_7H_7N_1Cl_2]^+$ = 174.9950).

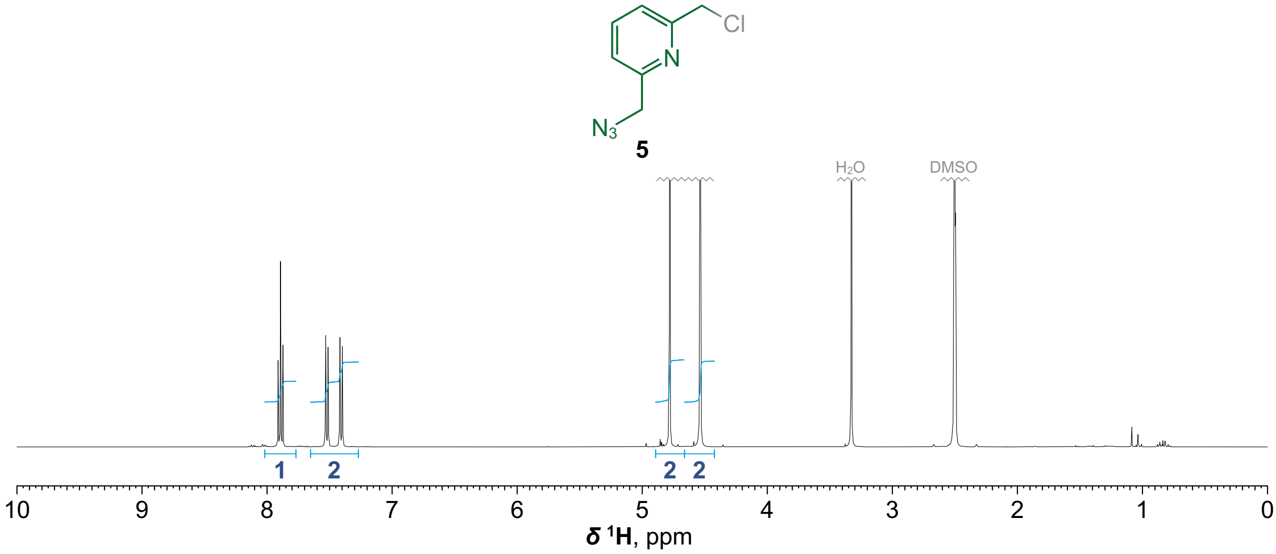

Supplementary Fig. 37. Synthesis and characterization of intermediate 5. In a round-bottom glass flask (250 mL), **4** (6.17 g; 35.0 mmol; 1.2 equiv.) was dissolved in MeCN (80 mL) followed by addition of solid NaN_3 (1.90 g; 29.2 mmol; 1.0 equiv.) and anhydrous K_2CO_3 (4.04 g; 29.2 mmol; 1.0 equiv.) and the resulting suspension was stirred for 5 days at 50 °C. The mixture was filtered on glass frit S3 and the solid residue was washed with MeCN. Filtrate was evaporated to dryness and the orange oily residue was purified by column chromatography (220 g SiO_2; 30% P.E. in DCM to 100% DCM). Fractions with product were combined and evaporated to dryness. The residue was further dried on high vacuum overnight to give product in the form of free base as faint yellow oil. **Yield:** 2.62 g (49%; 1 step; based on NaN_3). **Recovery**: 2.21 g of **4** (36% of the initial amount used). **NMR (DMSO-d$_6$): ^{1}H** (401.0 MHz) δ_H 4.53 (CH_2, s, 2H); 4.78 (CH_2, s, 2H); 7.40 (CH, d, 1H, $^3J_{HH}$ = 7.7, $^4J_{HH}$ = 1.0); 7.52 (CH, d, 1H, $^3J_{HH}$ = 7.8, $^4J_{HH}$ = 1.0); 7.89 (CH, dd, 1H, $^3J_{HH}$ = 7.8, $^3J_{HH}$ = 7.7). **^{13}C{^{1}H}** (100.8 MHz) δ_C 46.6 (CH$_2$, s); 54.2 (CH$_2$, s); 121.9 (CH, s); 122.6 (CH, s); 138.5 (CH, s); 155.7 (C, s); 156.3 (C, s). **ESI-HRMS:** 183.0433 [M+H]$^+$ (theor. $[C_7H_8N_4Cl_1]^+$ = 183.0432).

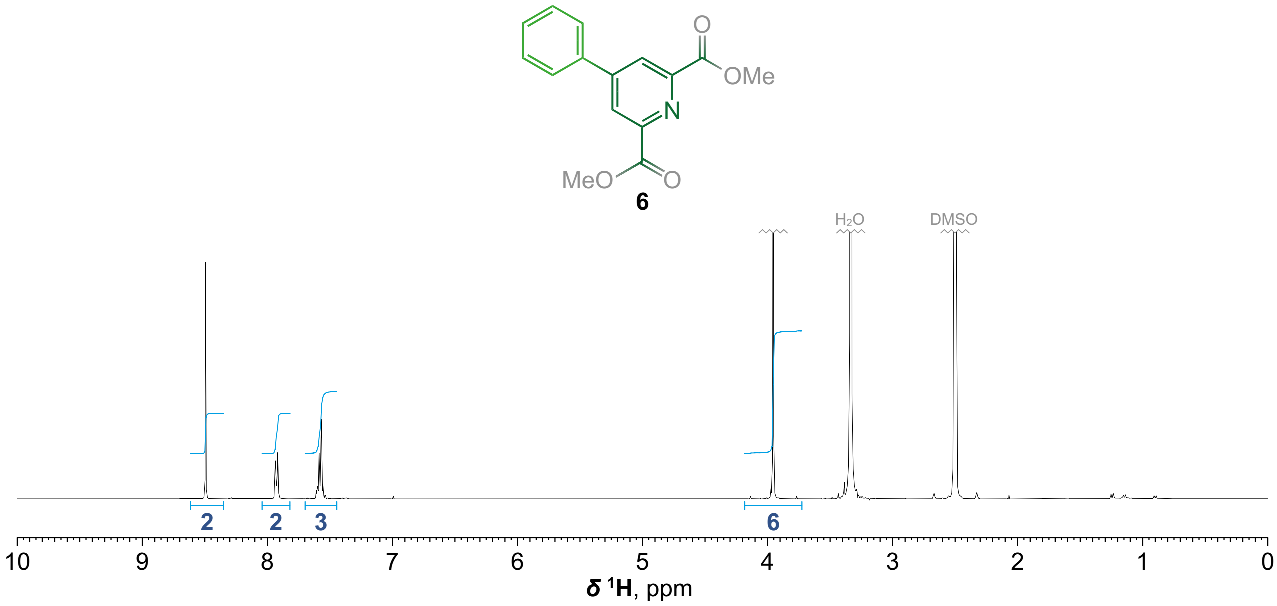

Supplementary Fig. 38. Synthesis and characterization of intermediate 6. Pear-shaped glass flask (250 mL) was charged with dimethyl *4*-chloropyridine-*2*,*6*-dicarboxylate (5.00 g; 21.8 mmol; 1.0 equiv.), phenylboronic acid (3.20 g; 26.2 mmol; 1.2 equiv.) and XPhos Pd G2 (510 mg; 648 µmol; 3.0 mol%) and was then three times secured with argon. Under constant flow of argon was then added dry DMF (110 mL) through septum followed by addition of freshly dried (using heat gun under high vacuum) Cs_2CO_3 (15.6 g; 47.9 mmol; 2.2 equiv.; the flask was briefly opened for addition). The mixture was then stirred for 20 h under septum (but without external argon) at 80 °C. Resulting dark mixture was filtered through glass frit (S3) and the filtrate was poured to a stirred beaker with H_2O (500 mL). Precipitate was collected on glass frit (S2), washed with H_2O and further dried on high vacuum overnight to give product in the form of free base as off white solid. **Yield:** 3.01 g (51%; 1 step; based on dimethyl *4*-chloropyridine-*2*,*6*-dicarboxylate). **NMR (DMSO-d_6):** **^{1}H** (400.1 MHz) δ_H 3.95 (CH_3, s, 6H); 7.45–7.57 (C*H*, m, 3H); 7.82–8.00 (C*H*, m, 2H); 8.48 (C*H*, s, 2H). **^{13}C{^{1}H}** (100.8 MHz) δ_C 52.7 (CH_3, s); 125.0 (*C*H, s); 127.2 (*C*H, s); 129.5 (*C*H, s); 130.2 (*C*H, s); 135.5 (*C*, s); 148.6 (*C*, s); 150.0 (*C*, s); 164.7 (*C*O, s). **ESI-HRMS:** 272.0916 $[M+H]^+$ (theor. $[C_{15}H_{14}N_1O_4]^+$ = 272.0917).

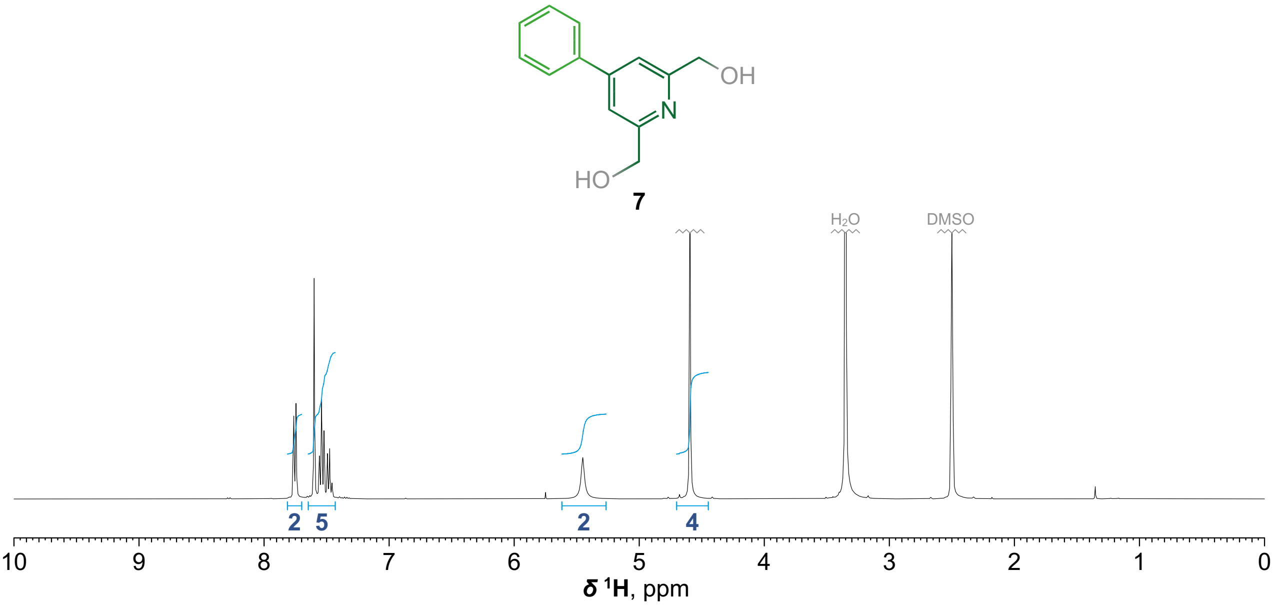

Supplementary Fig. 39. Synthesis and characterization of intermediate 7. In a round-bottom glass flask (500 mL), **6** (2.98 g; 11.0 mmol; 1.0 equiv.) was dissolved in a mixture of MeOH (100 mL) and THF (100 mL). The resulting slightly yellow solution was cooled with an ice bath (~5 °C) followed by portion wise additions (over course of 30 min, with no stopper on the flask) of solid $NaBH_4$ (2.90 g; 76.7 mmol; 9.0 equiv.) during which hydrogen gas evolved intensively and the color of the mixture changed to red and orange. After the addition, the flask was allowed to warm up to RT and was further stirred for 30 min at RT. Resulting faint yellow opalescent solution was filtered through syringe microfilter (PTFE). The filtrate was evaporated to dryness and further co-evaporated with DCM (as a suspension). Residue was dissolved in a mixture of H_2O (300 mL) and DCM (300 mL) and transferred to a separatory funnel. After shaking, the bottom phase was separated. Aqueous phase was further extracted with DCM (8 × 75 mL). Combined organic layers were dried with anhydrous Na_2SO_4, filtered through glass frit (S3) and evaporated to dryness. The resulting crystalized residue was crushed and further dried on high vacuum overnight to give product in the form of free base as nearly colorless solid. **Yield:** 2.29 g (97%; 1 step; based on **6**). **NMR (DMSO-d$_6$):** ^{1}**H** (400.1 MHz) δ_H 4.59 (C*H*$_2$, s, 4H); 5.45 (O*H*, s, 2H); 7.45–7.57 (C*H*, m, 3H); 7.60 (C*H*, s, 2H); 7.68–7.82 (C*H*, m, 2H). ^{13}**C{^{1}H}** (100.8 MHz) δ_C 64.2 (*C*H$_2$, s); 115.7 (*C*H, s); 126.7 (*C*H, s); 129.1 (*C*H, s); 129.3 (*C*H, s); 138.1 (*C*, s); 148.2 (*C*, s); 161.7 (*C*, s). **ESI-HRMS:** 216.1022 $[M+H]^+$ (theor. $[C_{13}H_{14}N_1O_2]^+$ = 216.1019).

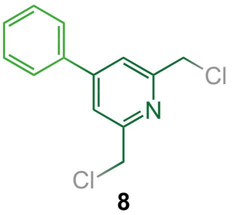

8

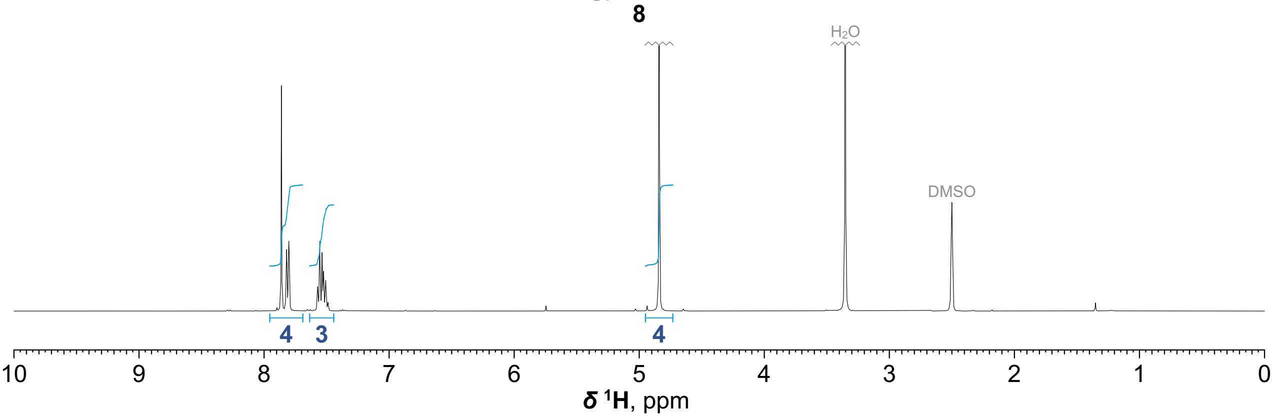

Supplementary Fig. 40. Synthesis and characterization of intermediate 8. In a round-bottom glass flask (500 mL), **7** (2.25 g; 10.5 mmol; 1.0 equiv.) was dissolved (with gentle heating) in DCM (250 mL). Freshly prepared solution of $SOCl_2$ (2.27 mL; 31.3 mmol; 3.0 equiv.) in DCM (10 mL) was then added, after which precipitate started to form. After 1 h of stirring at RT, the resulting clear yellow solution was slowly diluted with dil. aq. solution of $NaHCO_3$ (150 mL) and the resulting biphasic mixture was vigorously stirred for additional 1 h at RT, during which bubbles of gas slowly evolved. Mixture was then transferred to a separatory funnel. After shaking, the bottom phase was separated. Aqueous phase was further extracted with DCM (3 × 100 mL). Combined organic layers were dried with anhydrous Na_2SO_4, filtered through glass frit (S3) and evaporated to dryness. The residue was further dried on high vacuum overnight to give product in the form of free base as faint yellow solid. **Yield:** 2.59 g (98%; 1 step; based on **7**). **NMR (DMSO-d$_6$):** **^{1}H** (400.1 MHz) δ_H 4.88 (C*H*$_2$, s, 4H); 7.47–7.58 (C*H*, m, 3H); 7.77–7.85 (C*H*, m, 2H); 7.86 (C*H*, s, 2H). **^{13}C{^{1}H}** (100.8 MHz) δ_C 46.6 (*C*H$_2$, s); 120.3 (*C*H, s); 126.9 (*C*H, s); 129.3 (*C*H, s); 129.7 (*C*H, s); 136.5 (*C*, s); 149.5 (*C*, s); 157.1 (*C*, s); 157.1 (*C*, s). **ESI-HRMS:** 252.0342 $[M+H]^+$ (theor. $[C_{13}H_{12}N_1Cl_2]^+$ = 252.0341).

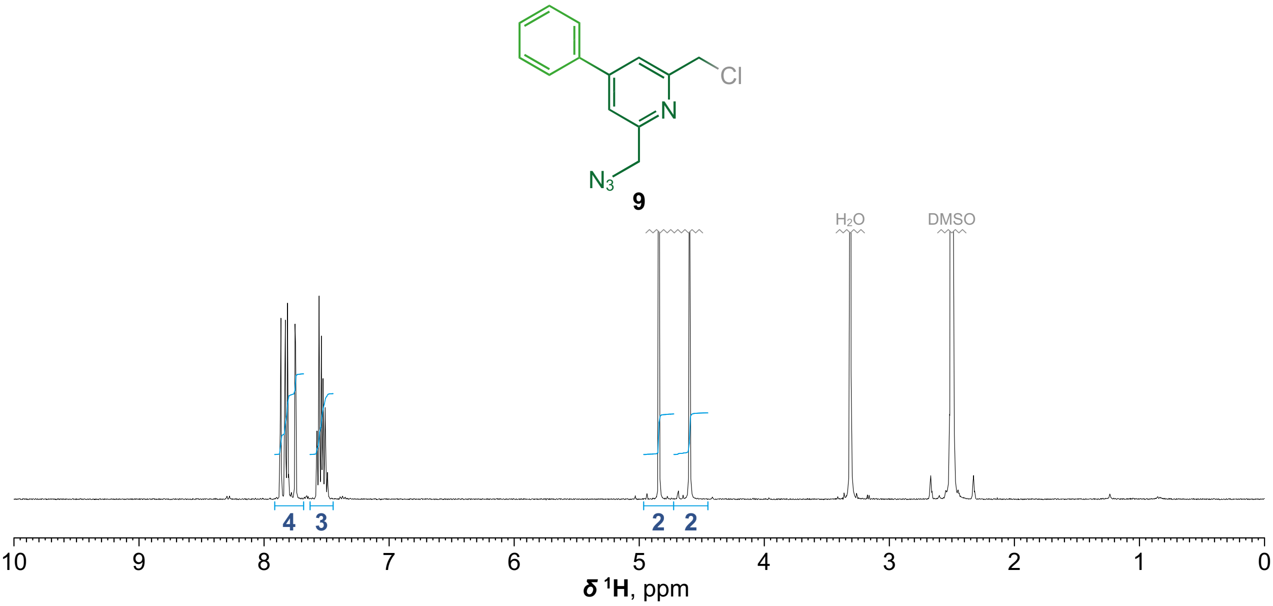

Supplementary Fig. 41. Synthesis and characterization of intermediate 9. In a pear-shaped glass flask (100 mL), **8** (1.60 g; 6.35 mmol; 1.3 equiv.) was dissolved (with gentle heating) in MeCN (60 mL) followed by addition of solid NaN_3 (320 mg; 4.92 mmol; 1.0 equiv.) and dried K_2CO_3 (680 mg; 4.93 mmol; 1.0 equiv.) and the resulting suspension was stirred for 24 h at 80 °C. The mixture was filtered on glass frit S3 and the solid residue was washed with MeCN. Filtrate was evaporated to dryness and the orange oily residue was purified by column chromatography (220 g SiO_2, 15% P.E. in DCM to 100% DCM). Fractions with product were combined and evaporated to dryness. The residue was further dried 1 h on high vacuum to give product in the form of free base as white solid. **Yield:** 691 mg (54%; 1 step; based on NaN_3). **Recovery**: 613 mg of **8** (38% of the initial amount used). **NMR (DMSO-d$_6$):** ^{1}**H** (400.1 MHz) δ_H 4.59 (CH_2, s, 2H); 4.84 (CH_2, s, 2H); 7.46–7.60 (CH, m, 3H); 7.74 (CH, d, 1H, $^4J_{HH}$ = 1.6); 7.77–7.85 (CH, m, 2H); 7.86 (CH, d, 1H, $^4J_{HH}$ = 1.6). ^{13}**C{^{1}H}** (100.8 MHz) δ_C 46.7 (CH$_2$, s); 54.2 (CH$_2$, s); 119.4 (CH, s); 120.0 (CH, s); 126.9 (CH, s); 129.3 (CH, s); 129.7 (CH, s); 136.6 (C, s); 149.3 (C, s); 156.6 (C, s); 157.1 (C, s). **ESI-HRMS:** 259.0747 $[M+H]^+$ (theor. $[C_{13}H_{12}N_4Cl_1]^+$ = 259.0745).

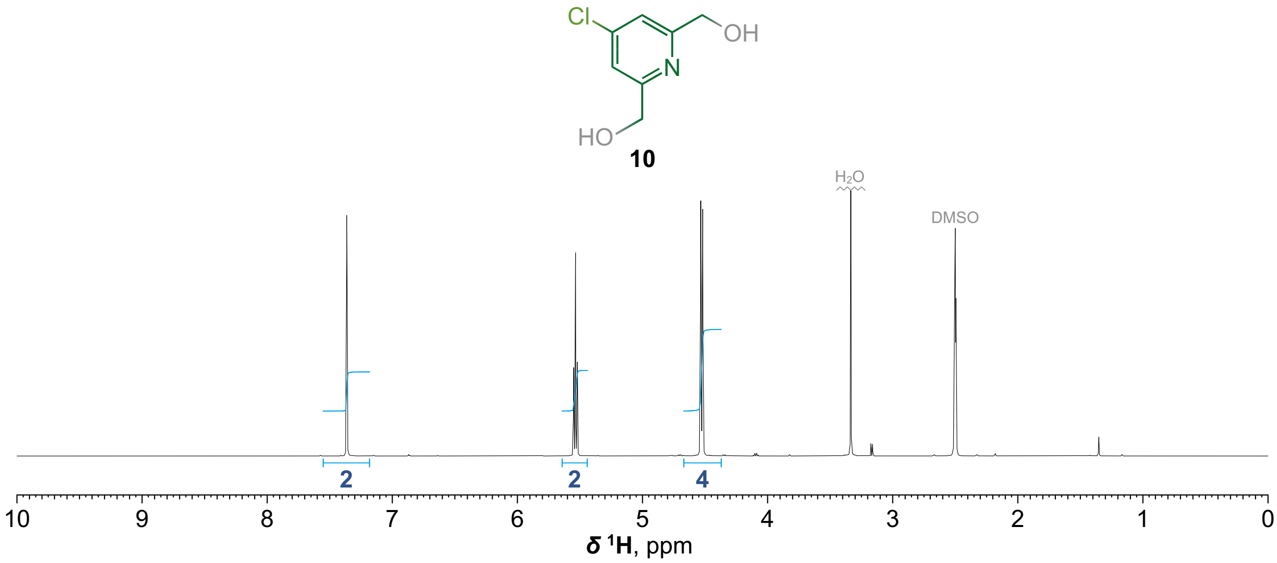

Supplementary Fig. 42. Synthesis and characterization of intermediate 10. In a round-bottom glass flask (1000 mL), dimethyl *4*-chloropyridine-*2*,*6*-dicarboxylate (15.0 g; 65.3 mmol; 1.0 equiv.) was gradually dissolved in a mixture of THF (540 mL) and MeOH (120 mL). The resulting slightly yellow solution was cooled with an ice bath (~5 °C) followed by portion wise additions (over course of 1 h, with no stopper on the flask) of solid $NaBH_4$ (12.4 g; 328 mmol; 5.0 equiv.) during which hydrogen gas evolved intensively and the color of the mixture changed to red, orange and eventually yellow. After the addition, the flask was allowed to warm up to RT and was further for stirred 16 h at RT. Resulting faint yellow opalescent solution was filtered on glass frit (S3), the filtrate was evaporated to dryness and further co-evaporated with DCM (as a suspension). Residue was dissolved in boiling H_2O (~600 mL) and the resulting highly alkaline solution was continuously extracted by DCM overnight. The organic layer (containing partly crystallized product) was evaporated to dryness. The residue was mechanically crushed and further dried on high vacuum to give product in the form of free base as nearly colorless microcrystalline powder. **Yield:** 10.84 g (96%; 1 step; based on dimethyl *4*-chloropyridine-*2*,*6*-dicarboxylate). **NMR (DMSO-d$_6$): ^{1}H** (401.0 MHz) δ_H 4.53 (C*H*$_2$, d, 4H, $^3J_{HH}$ = 6.0); 5.54 (O*H*, t, 2H, $^3J_{HH}$ = 6.0); 7.36 (C*H*, s, 2H). **^{13}C{^{1}H}** (100.8 MHz) δ_C 63.7 (*C*H$_2$, s); 118.0 (*C*H, s); 144.0 (*C*, s); 163.5 (*C*, s). **ESI-HRMS:** 174.0317 $[M+H]^+$ (theor. $[C_7H_9N_1O_2Cl_1]^+$ = 174.0316). **EA** ($C_7H_8N_1O_2Cl_1$, M_R = 173.6): C 48.4 (48.5); H 4.7 (4.5); N 7.7 (8.1); Cl 20.4 (21.2).

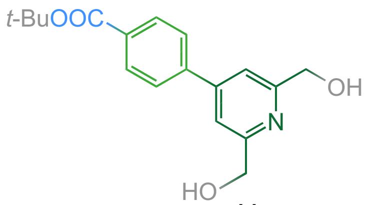

11

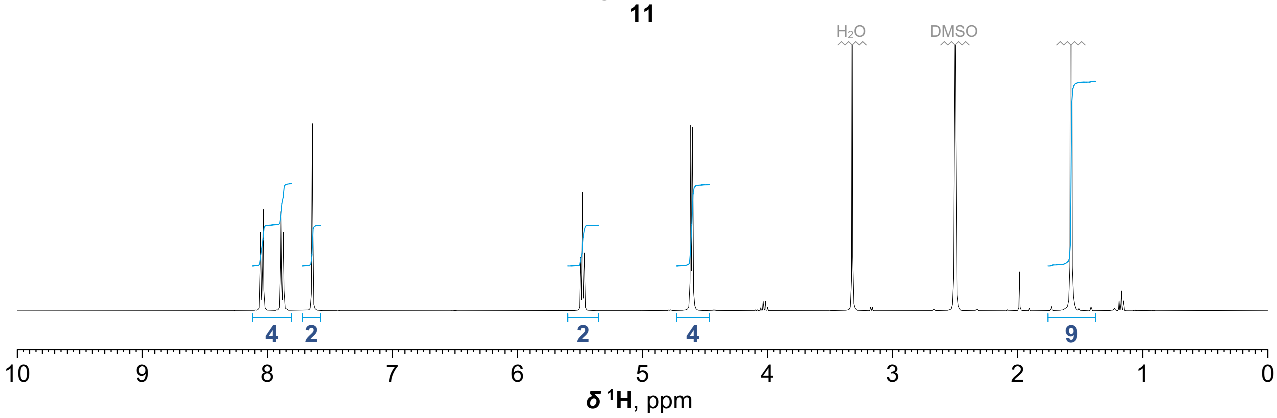

Supplementary Fig. 43. Synthesis and characterization of intermediate 11. Glass vial (40 mL) was charged with **10** (628 mg; 3.62 mmol; 1.0 equiv.), (*4*-(tert-butoxycarbonyl)phenyl)boronic acid (880 mg; 3.96 mmol; 1.1 equiv.) and XPhos Pd G2 (57 mg; 7.2 µmol; 2.0 mol%) and was then three times secured with argon. Under constant flow of argon was then added dry *1,4*-Dioxane (16 mL) through septum followed by addition of freshly prepared (and briefly washed with argon prior addition) solution of $K_3PO_4 \cdot H_2O$ (920 mg; 4.00 mmol; 1.1 equiv.) in H_2O (8 mL). The mixture was then stirred for 16 h under septum (but without external argon) at 80 °C. Resulting dark mixture was transferred to a separatory funnel and diluted with EtOAc (40 mL) and H_2O (50 mL). After shaking, upper layer was separated and aqueous layer was further extracted with EtOAc (5 × 30 mL). Combined organic layers were dried with anhydrous Na_2SO_4, filtered through glass frit (S3) and evaporated to dryness. Residue was purified on flash chromatography (120 g SiO_2, 100% EtOAc to 30% MeOH in EtOAc). Combined fractions with product were evaporated to dryness and further co-evaporated once with DCM. The residue was further dried on high vacuum overnight to give product in the form of free base as off-white powder. **Yield:** 1.04 g (91%; 1 step; based on **10**). **NMR (DMSO-d*6*):** ^{1}**H** (400.1 MHz) δ_H 1.57 (CH_3, s, 9H); 4.61 (CH_2, d, 4H, $^3J_{HH}$ = 5.8); 5.48 (O*H*, t, 2H, $^3J_{HH}$ = 5.8); 7.64 (C*H*, s, 2H); 7.88 (C*H*, d, 2H, $^3J_{HH}$ = 8.3); 8.04 (C*H*, d, 2H, $^3J_{HH}$ = 8.3). ^{13}**C{^{1}H}** (100.8 MHz) δ_C 27.8 (*C*H_3, s); 64.2 (*C*H_2, s); 81.0 (*C*–CH_3, s); 115.8 (*C*H, s); 127.0 (*C*H, s); 129.9 (*C*H, s); 131.6 (*C*, s); 142.2 (*C*, s); 147.0 (*C*, s); 162.0 (*C*, s); 164.6 (*C*O, s). **ESI-HRMS:** 316.1539 $[M+H]^+$ (theor. $[C_{18}H_{22}N_1O_4]^+$ = 316.1543).

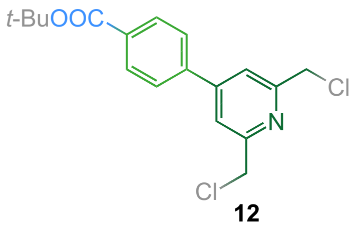

12

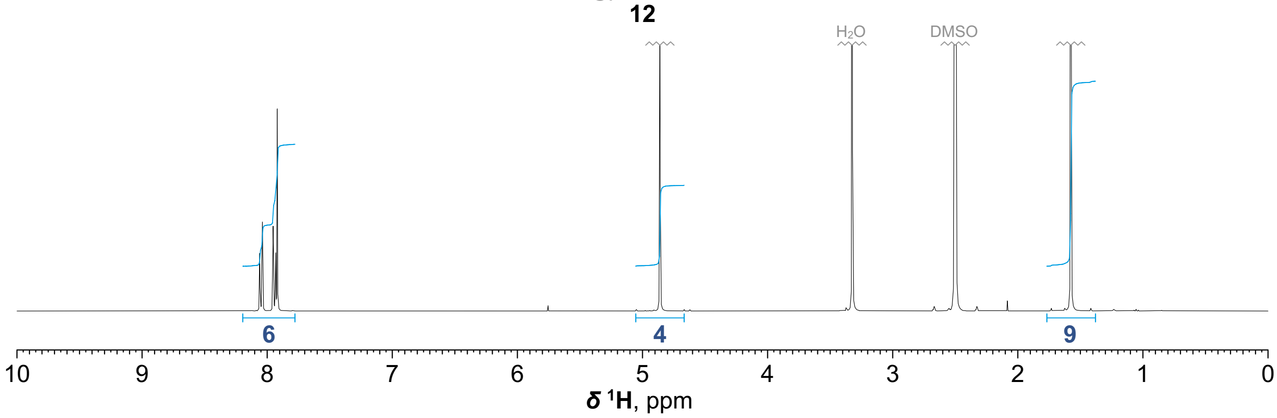

Supplementary Fig. 44. Synthesis and characterization of intermediate 12. In a round-bottom glass flask (250 mL), **11** (1.04 g; 3.30 mmol; 1.0 equiv.) was suspended in DCM (35 mL). Freshly prepared solution of $SOCl_2$ (721 µL; 9.93 mmol; 3.0 equiv.) in DCM (5 mL) was then added. Clear solution was quickly formed and the open flask was stirred for 1 h at RT. Mixture was then quenched by addition of dil. aq. solution of $NaHCO_3$ (60 mL). The resulting biphasic mixture was vigorously stirred for additional 1 h at RT, during which bubbles of gas slowly evolved. Mixture was then transferred to a separatory funnel. After shaking, the bottom phase was separated. Aqueous phase was further extracted with DCM (5 × 30 mL). Combined organic layers were dried with anhydrous Na_2SO_4, filtered through glass frit (S3) and evaporated to dryness. The residue was purified on flash chromatography (120 g SiO_2, 100% DCM to 20% EtOAc in DCM). Combined fractions with product were evaporated to dryness and further co-evaporated once with DCM. The residual faint yellow oil slowly crystallized. The solid was crushed and further dried on high vacuum overnight to give product in the form of free base as off-white powder. **Yield:** 839 mg (66%; 1 step; based on **11**). **NMR (DMSO-d$_6$):** ^{1}**H** (400.1 MHz) δ_H 1.58 (CH_3, s, 9H); 4.86 (CH_2, s, 4H); 7.92 (C*H*, s, 2H); 7.94 (C*H*, d, 2H, $^3J_{HH}$ = 8.7); 8.05 (C*H*, d, 2H, $^3J_{HH}$ = 8.7). ^{13}**C{^{1}H}** (100.8 MHz) δ_C 27.8 (CH_3, s); 46.5 (CH_2, s); 81.2 (*C*–CH_3, s); 120.6 (*C*H, s); 127.2 (*C*H, s); 129.9 (*C*H, s); 132.1 (*C*, s); 140.6 (*C*, s); 148.3 (*C*, s); 157.3 (*C*, s); 164.5 (*C*O, s). **ESI-HRMS:** 352.0866 $[M+H]^+$ (theor. $[C_{18}H_{20}N_1O_2Cl_2]^+$ = 352.0866).

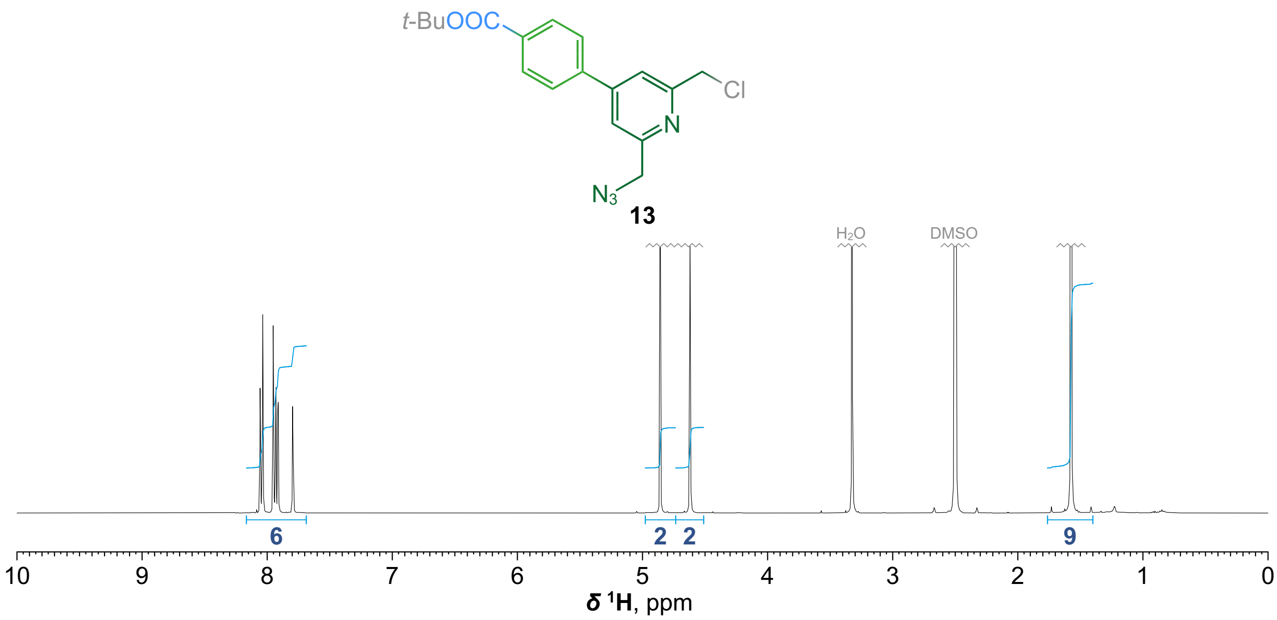

Supplementary Fig. 45. Synthesis and characterization of intermediate 13. In a glass vial (20 mL), **12** (837 mg; 2.38 mmol; 1.3 equiv.) was dissolved in MeCN (18 mL) followed by addition of solid NaN_3 (119 mg; 1.83 mmol; 1.0 equiv.) and dried K_2CO_3 (253 mg; 1.83 mmol; 1.0 equiv.) and the resulting suspension was stirred for 24 h at 80 °C. The mixture was filtered on through syringe microfilter (PTFE) and the solid residue was washed with MeCN. Filtrate was evaporated to dryness and oily residue was purified by column chromatography (SiO_2, 20% Pentane in DCM to 1% EtOAc in DCM). Fractions with product were combined and evaporated to dryness. The resulting nearly colorless oil was further dried on high vacuum overnight (where product slowly crystallized) to give product in the form of free base as colorless solid. **Yield:** 230 mg (35%; 1 step; based on NaN_3). **Recovery**: 442 mg of **12** (53% of the initial amount used). **NMR (DMSO-d*6*):** ^{1}**H** (401.0 MHz) δ_H 1.57 (C*H*$_3$, s, 9H); 4.62 (C*H*$_2$, s, 2H); 4.86 (C*H*$_2$, s, 2H); 7.80 (C*H*, d, 1H, $^4J_{HH} = 1.6$); 7.91 (C*H*, d, 1H, $^4J_{HH} = 1.6$); 7.94 (C*H*, d, 2H, $^3J_{HH} = 8.6$); 8.05 (C*H*, d, 2H, $^3J_{HH} = 8.6$). ^{13}**C{^{1}H}** (100.8 MHz) δ_C 27.8 (*C*H$_3$, s); 46.6 (*C*H$_2$, s); 54.2 (*C*H$_2$, s); 81.2 (*C*–CH$_3$, s); 119.6 (*C*H, s); 120.3 (*C*H, s); 127.2 (*C*H, s); 129.9 (*C*H, s); 132.1 (*C*, s); 140.7 (*C*, s); 148.1 (*C*, s); 156.8 (*C*, s); 157.3 (*C*, s); 164.5 (*C*O, s). **ESI-HRMS:** 359.1270 $[M+H]^+$ (theor. $[C_{18}H_{20}N_4O_2Cl_1]^+$ = 359.1269).

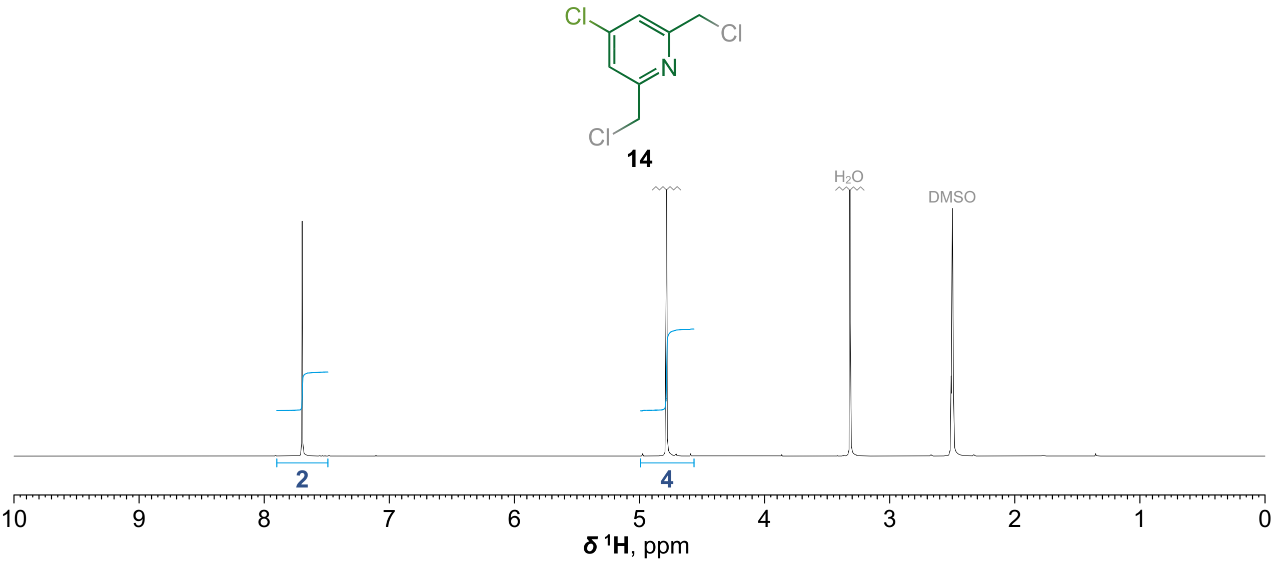

Supplementary Fig. 46. Synthesis and characterization of intermediate 14. In a round-bottom glass flask (100 mL), **10** (1.00 g; 5.76 mmol; 1.0 equiv.) was suspended in DCM (55 mL) followed by addition of $SOCl_2$ (1.25 mL; 17.2 mmol; 3.0 equiv.). The mixture was stirred for 1 h at RT, producing clear solution. Dil. aq. solution of $NaHCO_3$ (50 mL) was then added and resulting biphasic mixture was vigorously stirred for additional 1 h at RT, during which bubbles of gas slowly evolved. Mixture was then transferred to a separatory funnel. After shaking, the bottom phase was separated. Aqueous phase was further extracted with DCM (4 × 50 mL). Combined organic layers were dried with anhydrous Na_2SO_4, filtered through glass frit (S3) and evaporated to dryness. The resulting crystalized residue was crushed and further dried on high vacuum overnight to give product in the form of free base as nearly colorless microcrystalline powder. **Yield:** 1.22 g (99%; 1 step; based on **10**). **NMR (DMSO-d$_6$):** ^{1}**H** (400.1 MHz) δ_H 4.79 (CH_2, s, 4H); 7.70 (*arom.*, s, 2H). ^{13}**C{^{1}H}** (100.8 MHz) δ_C 45.7 (CH$_2$, s); 122.8 (CH, s); 144.4 (C, s); 158.2 (C, s). **ESI-HRMS:** 209.9638 $[M+H]^+$ (theor. $[C_7H_7N_1Cl_3]^+$ = 209.9639).

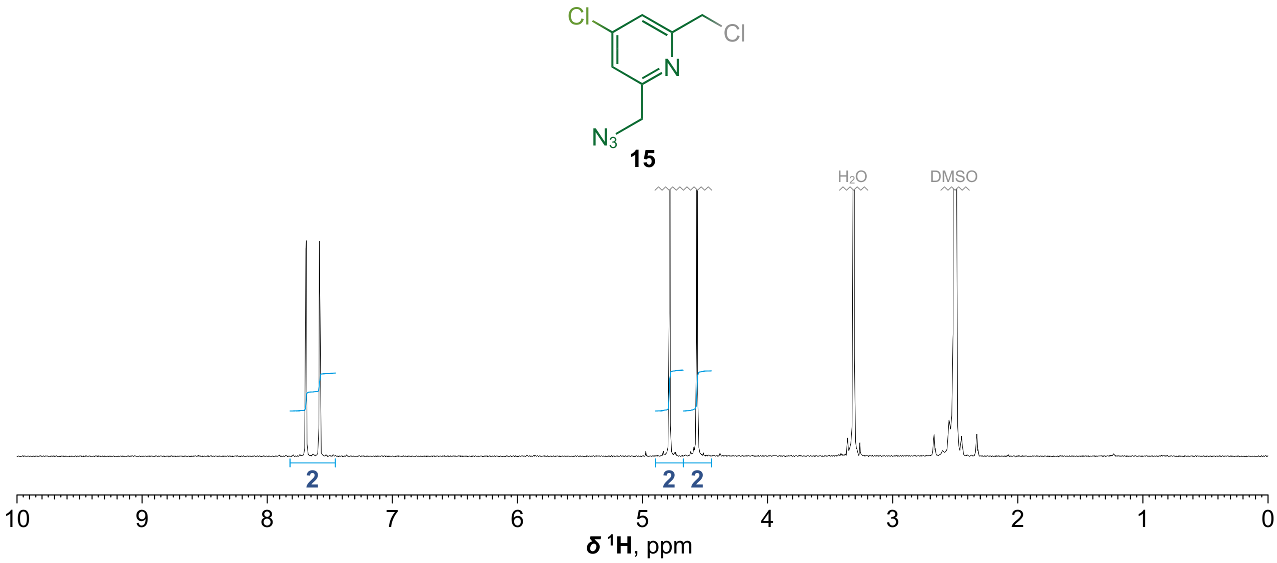

Supplementary Fig. 47. Synthesis and characterization of intermediate 15. In a glass vial (40 mL), **14** (1.65 g; 7.84 mmol; 1.3 equiv.) was dissolved in MeCN (35 mL) followed by addition of solid NaN_3 (392 mg; 6.03 mmol; 1.0 equiv.) and dried K_2CO_3 (833 mg; 6.03 mmol; 1.0 equiv.) and the resulting suspension was stirred for 9 h at 70 °C. The mixture was filtered on through syringe microfilter (PTFE) and the solid residue was washed with MeCN. Filtrate was evaporated to dryness and yellow oily residue was purified by column chromatography (SiO_2, 40% P.E. in DCM to 10% P.E. in DCM). Fractions with product were combined and evaporated to dryness. The residue was further dried on high vacuum overnight to give product in the form of free base as nearly colourless oil, that subsequently crystallized on standing in the freezer. **Yield:** 755 mg (58%; 1 step; based on NaN_3). **Recovery**: 540 mg of **14** (33% of the initial amount used). **NMR (DMSO-d*6*):** ^{1}**H** (401.0 MHz) δ_H 4.56 (C*H*$_2$, s, 2H); 4.78 (C*H*$_2$, s, 2H); 7.58 (C*H*, d, 1H, $^{4}J_{HH}$ = 1.9); 7.69 (C*H*, d, 1H, $^{4}J_{HH}$ = 1.9). ^{13}**C{**^{1}**H}** (100.8 MHz) δ_C 45.8 (*C*H$_2$, s); 53.6 (*C*H$_2$, s); 121.9 (*C*H, s); 122.5 (*C*H, s); 144.4 (*C*, s); 157.9 (*C*, s); 158.2 (*C*, s). **ESI-HRMS:** 217.0040 $[M+H]^+$ (theor. $[C_7H_7N_4Cl_2]^+$ = 217.0042).

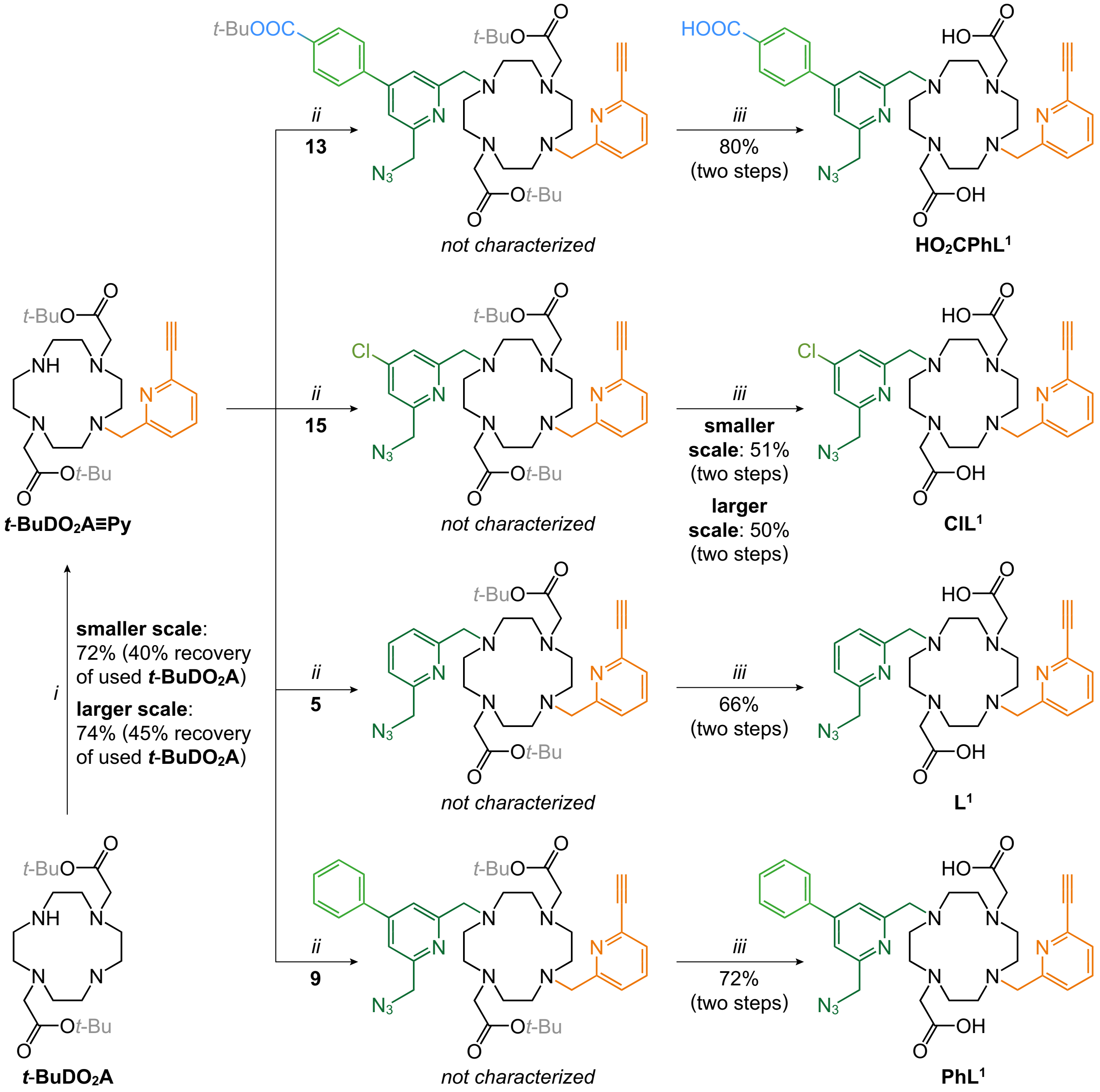

Supplementary Fig. 48. Synthesis of alkyne and azide bearing ligands. The protected ligands (stable enough to be isolated) were obtained by step-wise alkylation of commercially available ***t*-BuDO₂A** (whose excess in respect to the alkylating agent was used in the first desymetrization step to minimize formation of double alkylated byproduct). Final ligands were then obtained after removal of *t*-Bu esters with TFA. ***Conditions:*** (*i*) **3**, K_2CO_3, MeCN, RT; (*ii*) K_2CO_3, MeCN, RT; (*iii*) TFA, RT. Yields refer to isolated compounds.

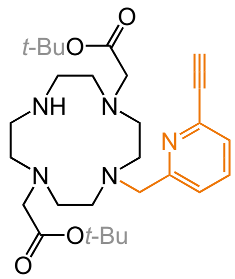

t-BuDO$_2$A≡Py

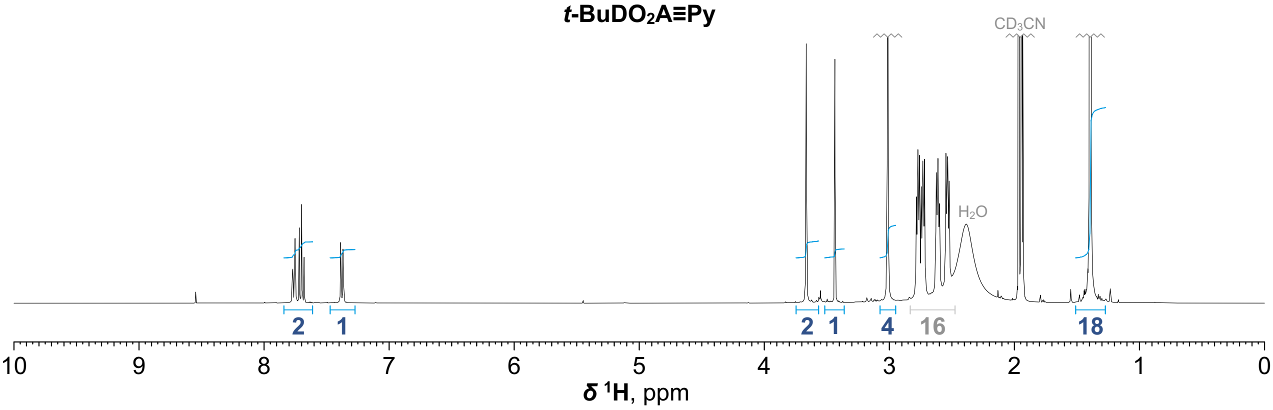

Supplementary Fig. 49. Synthesis and characterization of intermediate *t*-BuDO$_2$A≡Py. In a pear-shaped glass flask (250 mL), ***t*-BuDO$_2$A** (free base; 1.31 g; 3.02 mmol; 2.3 equiv.) was dissolved in MeCN (50 mL) followed by addition of dried K_2CO_3 (228 mg; 1.65 mmol; 1.0 equiv.). To the vigorously stirred reaction mixture, solution of **3** (250 mg; 1.65 mmol; 1.0 equiv.) in MeCN (100 mL) was added dropwise (over the course of 8 h). Resulting mixture was further stirred for 24 h at RT. Mixture was filtered through syringe microfilter (PTFE) and the filtrate was evaporated to dryness. Residue was purified by preparative HPLC (C18; H_2O–MeCN gradient with FA additive). Fractions with product were combined, neutralized with dil. aq. $NaHCO_3$ and evaporated to dryness. Residue was dissolved in DCM (100 mL) and H_2O (100 mL) and transferred to a separatory funnel. After shaking, the bottom phase was separated. Aqueous phase was further extracted with DCM (4 × 50 mL). Combined organic layers were dried with anhydrous Na_2SO_4, filtered through glass frit (S3) and evaporated to dryness. The residue was further dried on high vacuum overnight to give product in the form of free base as yellow oil that solidifed on standing. **Yield:** 614 mg (72%; 1 step; based on **3**). **Recovery**: 522 mg of ***t*-BuDO$_2$A** (40% of the initial amount used). **NMR (CD_3CN): ^{1}H** (401.0 MHz) δ_H 1.39 (CH_3, s, 18H); 2.53 (CH_2–N, m, 4H); 2.61 (CH_2–N, m, 4H); 2.73 (CH_2–N, m, 4H); 2.77 (CH_2–N, m, 4H); 3.01 (CH_2–CO, s, 4H); 3.44 (C≡C*H*, s, 1H); 3.66 (CH_2–arom., s, 2H); 7.38 (C*H*, dd, 1H, $^3J_{HH}$ = 7.5, $^4J_{HH}$ = 1.3); 7.70 (C*H*, dd, 1H, $^3J_{HH}$ = 7.9, $^3J_{HH}$ = 7.5); 7.76 (C*H*, dd, 1H, $^3J_{HH}$ = 7.9, $^4J_{HH}$ = 1.3). **^{13}C{^{1}H}** (100.8 MHz) δ_C 28.4 (*C*H_3, s); 48.2 (*C*H_2, s); 51.8 (*C*H_2, s); 52.6 (*C*H_2, s); 54.9 (*C*H_2, s); 57.6 (*C*H_2–CO, s); 59.3 (*C*H_2–arom., s); 78.0 (C≡*C*H, s); 81.3 (*C*–CH_3, s); 84.0 (*C*≡CH, s); 124.9 (*C*H, s); 126.5 (*C*H, s); 137.8 (*C*H, s); 141.6 (*C*, s); 162.8 (*C*, s); 172.0 (*C*O, s). **ESI-HRMS:** 516.3542 [M+H]$^+$ (theor. $[C_{28}H_{46}N_5O_4]^+$ = 516.3544).

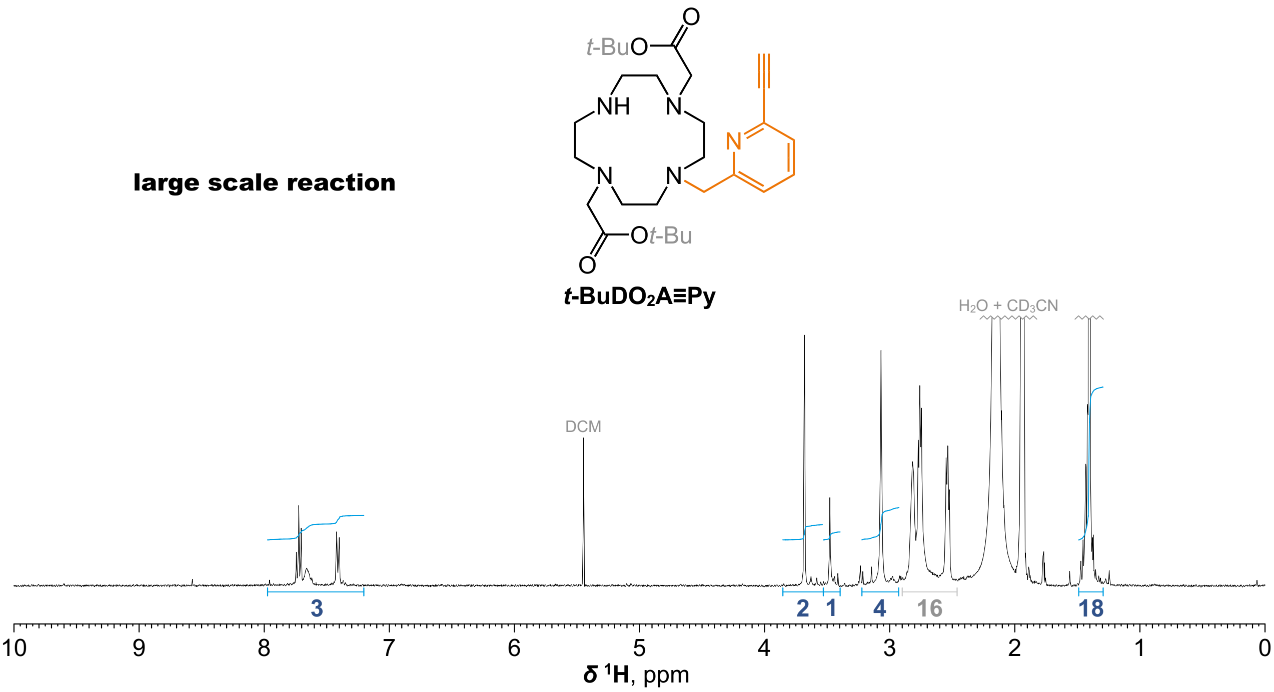

Supplementary Fig. 50. Large scale synthesis of intermediate *t*-BuDO$_2$A≡Py. In a pear-shaped glass flask (500 mL), ***t*-BuDO$_2$A** (free base; 3.48 g; 8.70 mmol; 2.2 equiv.) was dissolved in MeCN (100 mL) followed by addition of dried K_2CO_3 (274 mg; 1.98 mmol; 0.5 equiv.). To the vigorously stirred reaction mixture, solution of **3** (600 mg; 3.96 mmol; 1.0 equiv.) in MeCN (200 mL) was added dropwise (over the course of 4 h). Resulting mixture was further stirred for 24 h at RT. Mixture was filtered through syringe microfilter (PTFE) and the filtrate was evaporated to dryness. Residue was purified by flash chromatography (C18; H_2O–MeCN gradient with FA additive). Fractions with product were combined, neutralized with dil. aq. $NaHCO_3$ and evaporated to dryness. Residue was dissolved in DCM (150 mL) and H_2O (150 mL) and transferred to a separatory funnel. After shaking, the bottom phase was separated. Aqueous phase was further extracted with DCM (4 × 75 mL). Combined organic layers were dried with anhydrous Na_2SO_4, filtered through glass frit (S3) and evaporated to dryness. The residue was further dried on high vacuum overnight to give product in the form of free base as yellow oil. **Yield:** 1.47 mg (74%; 1 step; based on **3**). **Recovery**: 1.56 g of ***t*-BuDO$_2$A** (45% of the initial amount used).

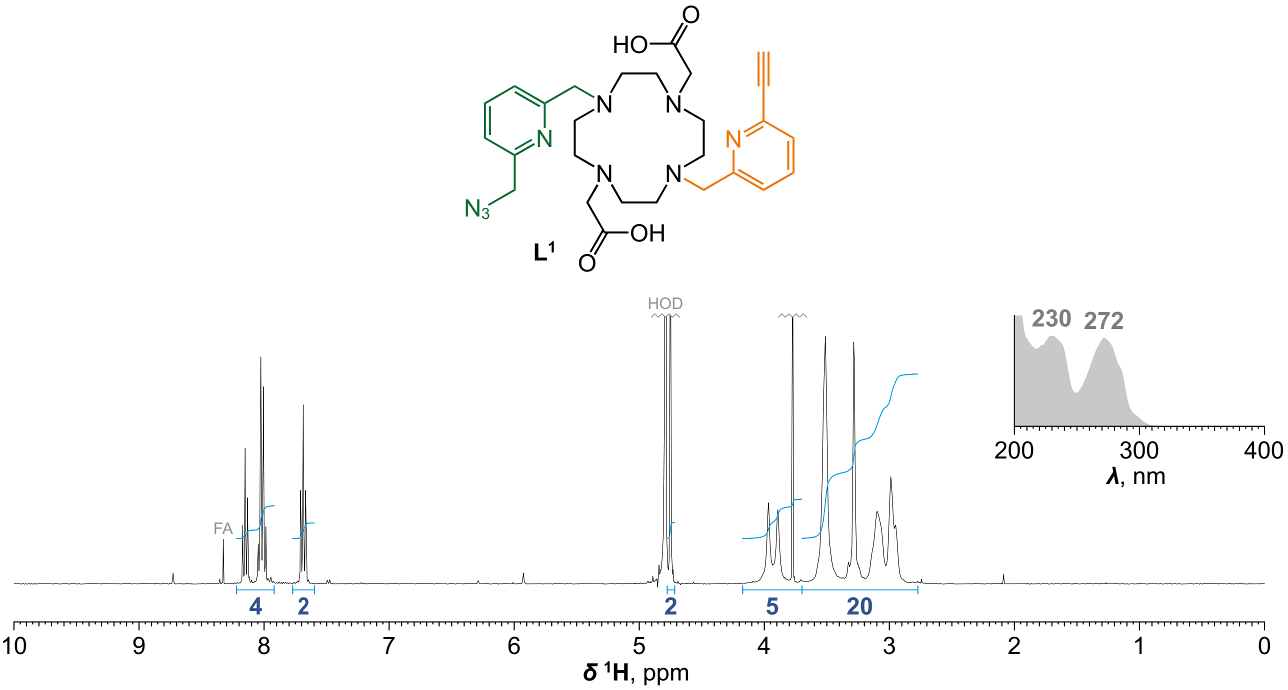

Supplementary Fig. 51. Synthesis and characterization of ligand $\mathbf{L^1}$. In a pear-shaped glass flask (25 mL), ***t*-BuDO$_2$A≡Py** (216 mg; 419 µmol; 1.0 equiv.) was dissolved in MeCN (12 mL) followed by addition of dried K_2CO_3 (174 mg; 1.26 mmol; 3.0 equiv.). Solution of **5** (92 mg; 504 µmol; 1.2 equiv.) in MeCN (3 mL) was then added and the resulting suspension was stirred for 3 d at RT. Solids were filtered off using syringe microfilter (PTFE) and filtrate was evaporated to dryness. Residue was purified by preparative HPLC (C18; H_2O–MeCN gradient with FA additive). Fractions with *tert*-butyl protected product were combined, evaporated to dryness and twice co-evaporated with MeOH to remove most of MeCN. Residue was dissolved in TFA (3 mL) and the resulting clear mixture was stirred for 16 h at RT. Mixture was evaporated to dryness and twice co-evaporated with MeOH to remove most of TFA. Residue was purified by preparative HPLC (C18; H_2O–MeCN gradient with FA additive). Fractions with product were joined and directly lyophilized to give product in nearly zwitterionic form as white fluffy solid. **Yield:** 161 mg (66%; 2 steps; based on ***t*-BuDO$_2$A≡Py**). **NMR (D_2O, pD ~4): ^{1}H** (400.1 MHz) δ_H 2.80–3.60 (C*H*$_2$–N, C*H*$_2$–CO, m, 16+4H); 3.74 (C≡C*H*, s, 1H); 3.86 (C*H*$_2$–arom., bs, 2H); 3.94 (C*H*$_2$–arom., bs, 2H); 4.72 (C*H*$_2$–N_3, s, 2H); 7.63–7.74 (C*H*, m, 2H); 7.95–8.03 (C*H*, m, 3H); 8.12 (C*H*, t, 1H, $^3J_{HH}$ = 7.8). **^{13}C{^{1}H}** (100.8 MHz) δ_C 48.3 (*C*H$_2$–N, s); 48.4 (*C*H$_2$–N, s); 51.4 (*C*H$_2$–N, s); 51.5 (*C*H$_2$–N, s); 54.3 (*C*H$_2$–N_3, s); 56.6 (*C*H$_2$–CO, s); 58.0 (*C*H$_2$–arom., s); 58.9 (*C*H$_2$–arom., s); 80.9 and 82.0 (*C*≡*C*H; 2 × s); 124.5 (*C*H, s); 126.2 (*C*H, s); 126.3 (*C*H, s); 128.5 (*C*H, s); 140.5 (*C*H, s); 141.2 (*C*, s); 142.7 (*C*H, s); 155.0 (*C*, s); 155.5 (*C*, s); 157.2 (*C*, s); 169.2 (*C*O, s). **ESI-HRMS:** 550.2880 [M+H]$^+$ (theor. $[C_{27}H_{36}N_9O_4]^+$ = 550.2885). **UV absorption:** λ_{max} = 230 nm; 272 nm. **EA** ($C_{27}H_{35}N_9O_4 \cdot 0.1FA \cdot 1.6H_2O$, M_R = 583.1): C 55.8 (55.5); H 6.6 (6.1); N 21.6 (21.2).

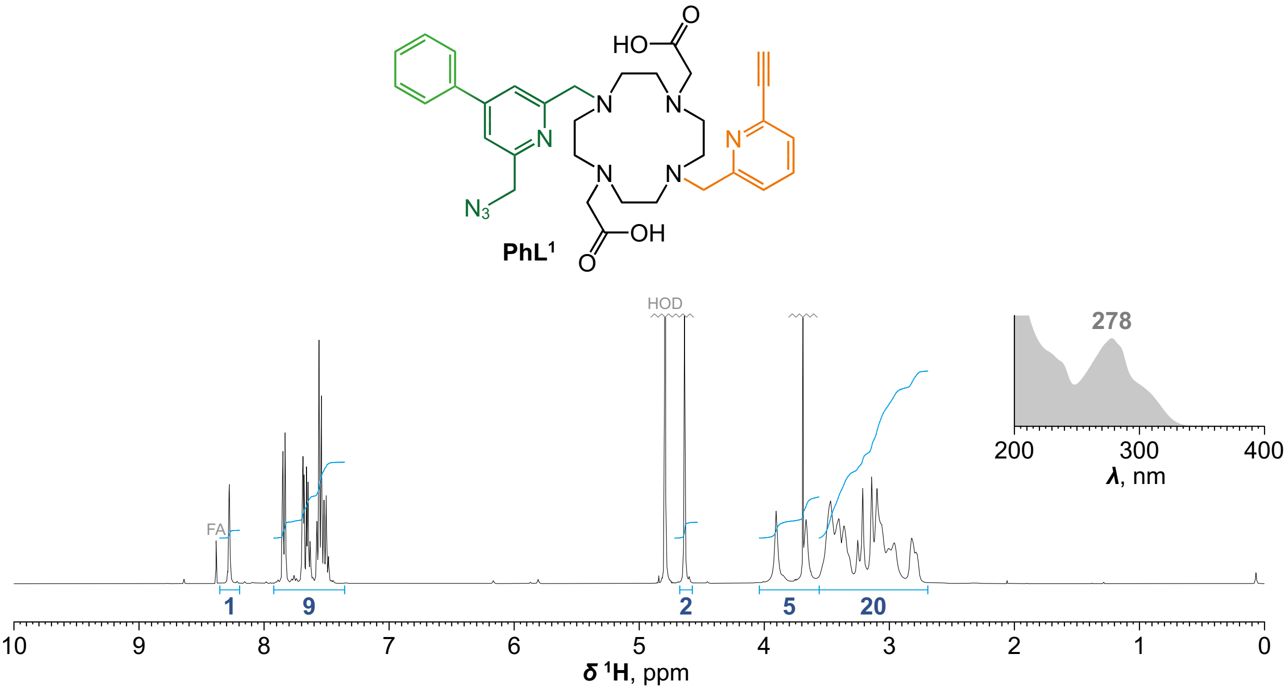

Supplementary Fig. 52. Synthesis and characterization of ligand PhL[1]. In a pear-shaped glass flask (50 mL), ***t*-BuDO$_2$A≡Py** (375 mg; 727 µmol; 1.0 equiv.) was dissolved in dry MeCN (20 mL) followed by addition of dried K_2CO_3 (300 mg; 2.17 mmol; 3.0 equiv.). Solution of **9** (189 mg; 731 µmol; 1.0 equiv.) in dry MeCN (5 mL) was then added and the resulting suspension was stirred for 24 h at RT. Solids were filtered off using syringe microfilter (PTFE) and filtrate was evaporated to dryness. Residue was purified by preparative HPLC (C18; H_2O–MeCN gradient with FA additive). Fractions with *tert*-butyl protected product were combined, evaporated to dryness and twice co-evaporated with MeOH to remove most of MeCN. Residue was dissolved in TFA (3 mL) and the resulting clear mixture was stirred for 16 h at RT. Mixture was evaporated to dryness and twice co-evaporated with MeOH to remove most of TFA. Residue was purified by preparative HPLC (C18; H_2O–MeCN gradient with FA additive). Fractions with product were joined and directly lyophilized to give product in nearly zwitterionic form as white fluffy solid. **Yield:** 342 mg (72%; 2 steps; based on ***t*-BuDO$_2$A≡Py**). **NMR (D_2O, pD ~4):** ^{1}**H** (401.0 MHz) δ_H 2.70–3.59 (CH_2–N, CH_2–CO, m, 16+4H); 3.66 (CH_2–arom., bs, 2H); 3.69 (C≡C*H*, s, 1H); 3.90 (CH_2–arom., bs, 2H); 4.64 (CH_2–N_3, s, 2H); 7.46–7.60 (C*H*, C*H*, m, 3+1H); 7.62 (C*H*, m, 3H); 7.79–7.88 (C*H*, m, 2H); 8.28 (C*H*, d, 1H, $^{4}J_{HH}$ = 1.6). ^{13}**C{^{1}H}** (100.8 MHz) δ_C 48.2 (*C*H_2–N, s); 48.3 (*C*H_2–N, s); 51.2 (*C*H_2–N, s); 51.3 (*C*H_2–N, s); 54.7 (*C*H_2–N_3, s); 56.4 (*C*H_2–CO, s); 57.8 (*C*H_2–arom., s); 59.1 (*C*H_2–arom., s); 80.3 and 82.4 (*C*≡*C*H; 2 × s); 121.9 (*C*H, s); 124.4 (*C*H, s); 125.6 (*C*H, s); 128.2 (*C*H, s); 128.5 (*C*H, s); 130.0 (*C*H, s); 130.8 (*C*H, s); 137.7 (*C*, s); 140.3 (*C*H, s); 141.2 (*C*, s); 153.6 (*C*, s); 155.4 (*C*, bs); 156.2 (*C*, s); 157.6 (*C*, s); 168.7 (*C*O, s). **ESI-HRMS:** 626.3195 $[M+H]^+$ (theor. $[C_{33}H_{40}N_9O_4]^+$ = 626.3198). **UV absorption:** λ_{max} = 278 nm. **EA** ($C_{33}H_{39}N_9O_4 \cdot 0.2FA \cdot 0.9H_2O$, M_R = 651.2): C 61.2 (61.6); H 6.4 (6.1); N 19.4 (19.1).

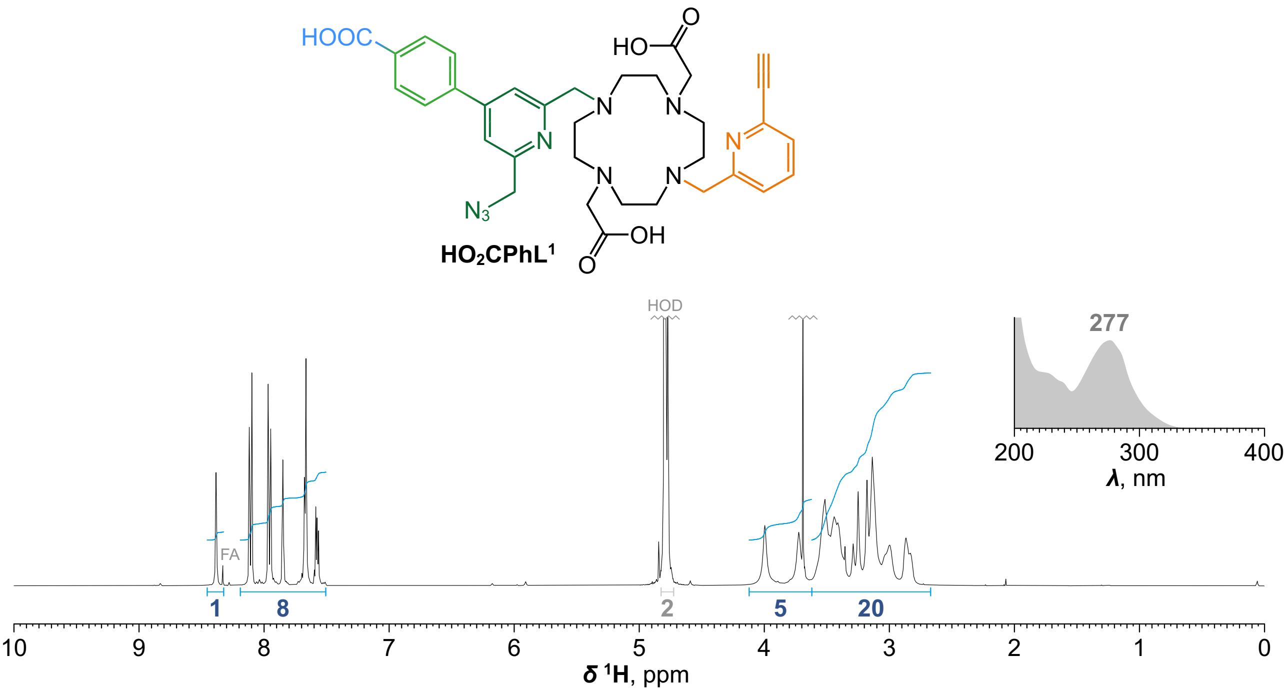

Supplementary Fig. 53. Synthesis and characterization of ligand HO_2CPhL^1. In a glass vial (20 mL), ***t*-BuDO$_2$A≡Py** (90 mg; 163 µmol; 1.1 equiv.) was dissolved in MeCN (6 mL) followed by addition of dried K_2CO_3 (85 mg; 616 µmol; 4.0 equiv.). Solution of **13** (55 mg; 153 µmol; 1.0 equiv.) in MeCN (2 mL) was then added and the resulting suspension was stirred for 18 h at RT. Solids were filtered off using syringe microfilter (PTFE) and filtrate was evaporated to dryness. Residue was purified by preparative HPLC (C18; H_2O–MeCN gradient with FA additive). Fractions with *tert*-butyl protected product were combined, evaporated to dryness and twice co-evaporated with MeOH to remove most of MeCN. Residue was dissolved in TFA (3 mL) and the resulting clear mixture was stirred for 16 h at RT. Mixture was evaporated to dryness and twice co-evaporated with MeOH to remove most of TFA. Residue was purified by preparative HPLC (C18; H_2O–MeCN gradient with FA additive). Fractions with product were joined and directly lyophilized to give product in nearly zwitterionic form as white solid. **Yield:** 87 mg (80%; 2 steps; based on **13**). **NMR (D_2O, pD ~4): ^{1}H** (401.0 MHz) δ_H 2.76–3.64 (CH_2–N, CH_2–CO, m, 16+4H); 3.69 (C≡C*H*, s, 1H); 3.72 (CH_2–arom., bs, 2H); 3.99 (CH_2–arom., bs, 2H); 4.77 (CH_2–N_3, s, 2H); 7.54–7.70 (C*H*, m, 3H); 7.85 (C*H*, d, 1H, $^4J_{HH}$ = 1.6); 7.96 (C*H*, d, 2H, $^3J_{HH}$ = 8.5); 8.11 (C*H*, d, 2H, $^3J_{HH}$ = 8.5); 8.38 (C*H*, d, 1H, $^4J_{HH}$ = 1.6). **ESI-HRMS:** 670.3094 $[M+H]^+$ (theor. $[C_{34}H_{40}N_9O_6]^+$ = 670.3096). **UV absorption:** λ_{max} = 277 nm. **EA** ($C_{34}H_{40}N_9O_6\cdot0.2FA\cdot1.6H_2O$, M_R = 707.8): C 58.0 (58.4); H 6.1 (5.9); N 17.8 (17.5).

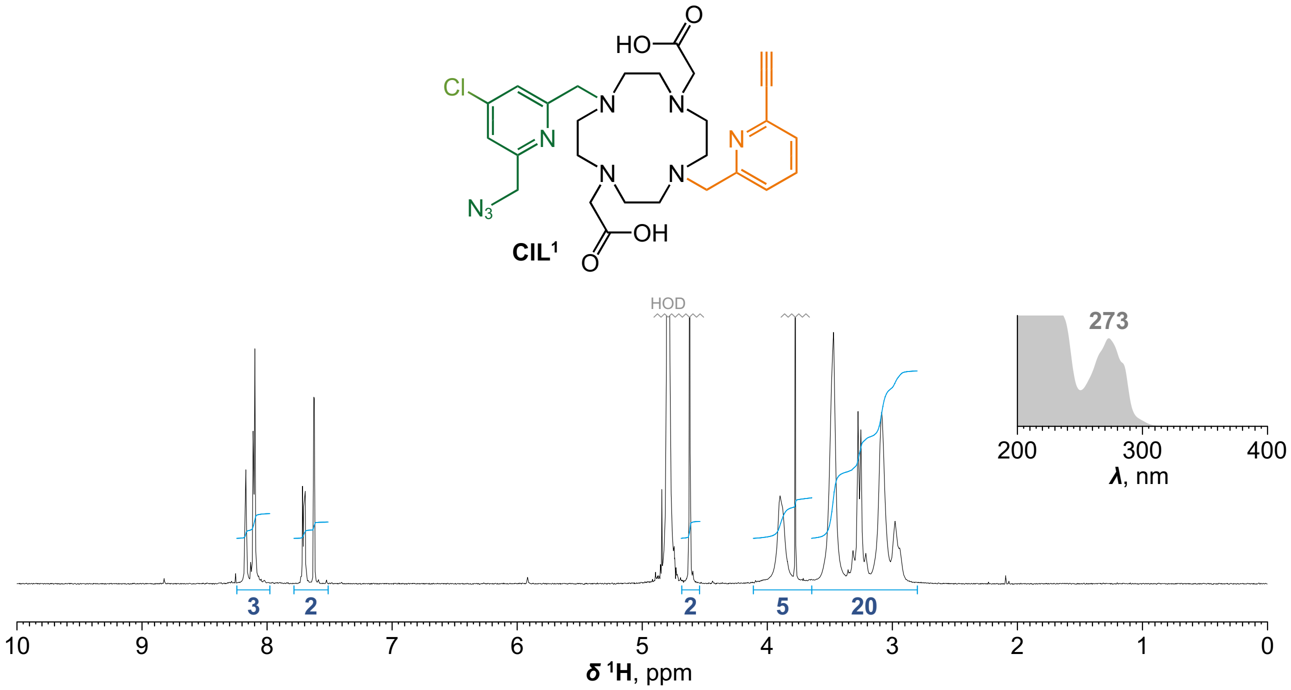

Supplementary Fig. 54. Synthesis and characterization of ligand ClL[1]. In a glass vial (20 mL), ***t*-BuDO$_2$A≡Py** (304 mg; 589 µmol; 1.1 equiv.) was dissolved in MeCN (7 mL) followed by addition of dried K_2CO_3 (305 mg; 2.21 mmol; 4.0 equiv.). Solution of **15** (120 mg; 553 µmol; 1.0 equiv.) in MeCN (3 mL) was then added and the resulting suspension was stirred for 2 d at RT. Solids were filtered off using syringe microfilter (PTFE) and filtrate was evaporated to dryness. Residue was purified by preparative HPLC (C18; H_2O–MeCN gradient with FA additive). Fractions with *tert*-butyl protected product were combined, evaporated to dryness and twice co-evaporated with MeOH to remove most of MeCN. Residue was dissolved in TFA (3 mL) and the resulting clear mixture was stirred for 16 h at RT. Mixture was evaporated to dryness and twice co-evaporated with MeOH to remove most of TFA. Residue was purified by preparative HPLC (C18; H_2O–MeCN gradient with FA additive). Fractions with product were joined and directly lyophilized to give product in the form of trifluoroacetate salt as white fluffy solid. **Yield:** 196 mg (51%; 2 steps; based on ***t*-BuDO$_2$A≡Py**). **NMR (D_2O, pD ~3):** **^{1}H** (401.0 MHz) δ_H 2.86–3.60 (CH_2–N, CH_2–CO, m, 16+4H); 3.77 (C≡C*H*, s, 1H); 3.79–4.06 (CH_2–arom., bm, 4H); 4.62 (CH_2–N_3, s, 2H); 7.62 (C*H*, d, 1H, $^4J_{HH}$ = 1.9); 7.67–7.73 (C*H*, m, 1H); 8.07–8.14 (C*H*, m, 2H); 8.17 (C*H*, d, 1H, $^4J_{HH}$ = 1.9); **ESI-HRMS:** 582.2344 [M−H]$^-$ (theor. $[C_{27}H_{33}N_9O_4Cl_1]^-$ = 582.2350). **UV absorption:** λ_{max} = 273 nm. **EA** ($C_{27}H_{34}N_9O_4Cl_1 \cdot 0.7TFA \cdot 1.7H_2O$, M_R = 694.5): C 49.1 (49.0); H 5.5 (5.4); N 18.2 (18.4); Cl 5.1 (5.3).

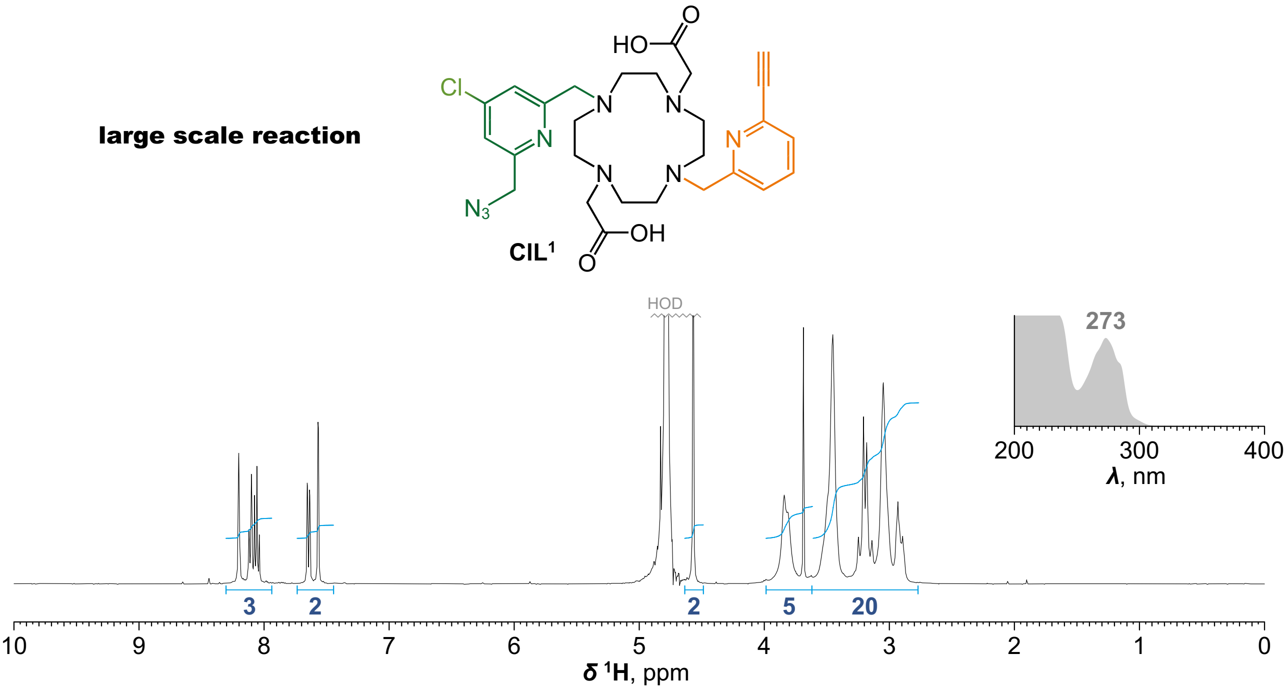

Supplementary Fig. 55. Large scale synthesis of ligand ClL^{1}. In a round-bottom glass flask (500 mL), ***t*-BuDO$_2$A≡Py** (1.47 g; 2.85 mmol; 1.1 equiv.) was dissolved in MeCN (50 mL) followed by addition of dried K_2CO_3 (1.43 g; 2.59 mmol; 4.0 equiv.). Solution of **15** (562 mg; 2.59 mmol; 1.0 equiv.) in MeCN (20 mL) was then added and the resulting suspension was stirred for 3 d at RT. Solids were filtered off using syringe microfilter (PTFE) and filtrate was evaporated to dryness. Residue was purified by flash chromatography (C18; H_2O–MeCN gradient with FA additive). Fractions with *tert*-butyl protected product were combined, evaporated to dryness and twice co-evaporated with MeOH to remove most of MeCN. Residue was dissolved in TFA (20 mL) and the resulting clear mixture was stirred for 12 h at RT. Mixture was evaporated to dryness and twice co-evaporated with MeOH to remove most of TFA. Residue was purified by flash chromatography (C18; H_2O–MeCN gradient with FA additive). Fractions with product were joined and directly lyophilized to give product in the form of trifluoroacetate salt as faint yellow fluffy solid. **Yield:** 905 mg (50%; 2 steps; based on **15**). **EA** ($C_{27}H_{34}N_9O_4Cl_1 \cdot 0.6TFA \cdot 2.4H_2O$, M_R = 695.7): C 48.7 (48.4); H 5.7 (5.4); N 18.1 (18.4); Cl 5.1 (5.3).

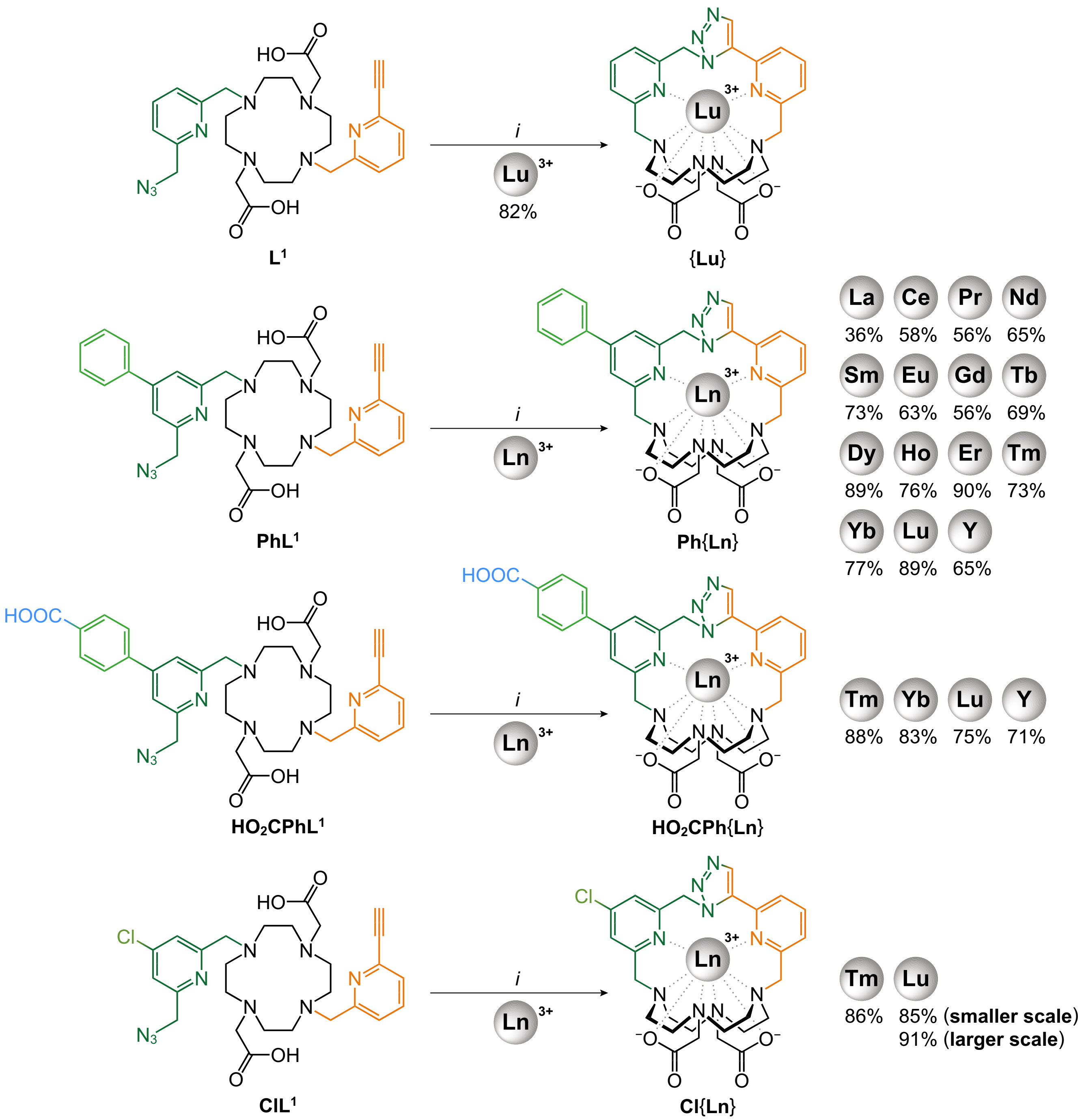

Supplementary Fig. 56. Synthesis of ClickZips. Straightforward synthesis of ClickZips from their corresponding ligand precursors by irreversible lanthanide templated Huisgen cycloadition reaction. No catalyst, additive or organic co-solvent is required. The reaction is entirely regioselective (*1*,*5*:*1*,*4* triazole = 100:0) due to favorable orientation of alkyne and azide group after Ln^{III} coordination, albeit formation of some intermolecular byproducts is inevitable (their abundance increases from Lu to La, but they can be easily removed during HPLC purification). ***Conditions:*** (*i*) MOPS/NaOH buffer (pH 7.0), H_2O, 80 °C. Yields refer to isolated compounds.

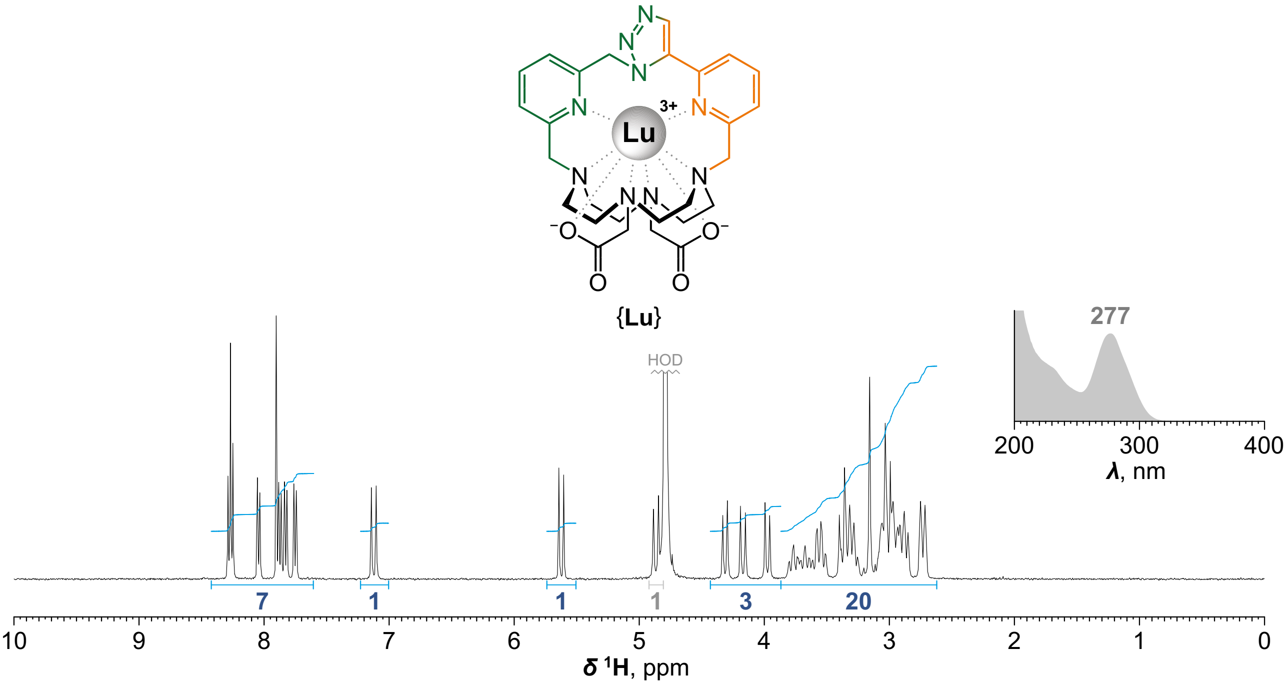

Supplementary Fig. 57. Synthesis and characterization of {Lu}. In a glass vial (20 mL), $\mathbf{L^1}\cdot 0.1FA\cdot 1.6H_2O$ (20.8 mg; 35.7 µmol; 1.0 equiv.) was dissolved in H_2O (17 mL) followed by addition aq. MOPS/NaOH buffer (500 mM; pH 7.0; 1.80 mL; 900 µmol; 25 equiv.) and aq. $LuCl_3$ (100 mM; 400 µL; 40.0 µmol; 1.1 equiv.) and the mixture was stirred for 16 h at 80 °C. Mixture was then filtered through syringe microfilter (RC). The filtrate was concentrated on rotary evaporator and then directly purified by preparative HPLC (C18; H_2O–MeCN gradient with TFA additive). Fractions with product were joined and directly lyophilized to give product in the form of trifluoroacetate salt as white fluffy solid. **Yield:** 26.6 mg (82%; 1 step; based on $\mathbf{L^1}\cdot 0.1FA\cdot 1.6H_2O$). **NMR ($D_2O$, pD ~3): ^{1}H** (400.1 MHz) δ_H 2.64–3.82 (CH_2–N, CH_2–CO, m, 16+4H); 3.95 (CH_2–arom., d, 1H, $^2J_{HH}$ = 14.7); 4.15 (CH_2–arom., d, 1H, $^2J_{HH}$ = 15.8); 4.29 (CH_2–arom., d, 1H, $^2J_{HH}$ = 14.7); 4.85 (CH_2–arom., d, 1H, $^2J_{HH}$ = 15.8); 5.60 (CH_2–N_3, d, 1H, $^2J_{HH}$ = 15.4); 7.10 (CH_2–N_3, d, 1H, $^2J_{HH}$ = 15.4); 7.73 (C*H*, d, 1H, $^3J_{HH}$ = 7.9); 7.80 (C*H*, d, 1H, $^3J_{HH}$ = 7.7); 7.85 (C*H*, d, 1H, $^3J_{HH}$ = 7.9); 7.88 (C*H*–N_3, s, 1H); 8.04 (C*H*, d, 1H, $^3J_{HH}$ = 7.7); 8.22–8.33 (C*H*, m, 2H). **ESI-HRMS:** 722.2060 $[M]^+$ (theor. $[C_{27}H_{33}N_9O_4Lu_1]^+$ = 722.2058). **UV absorption:** λ_{max} = 277 nm. **EA** ($[C_{27}H_{33}N_9O_4Lu_1]^+[TFA]^-\cdot 0.3TFA\cdot 2.4H_2O$, M_R = 913.0): C 38.9 (38.7); H 4.2 (3.8); N 13.8 (13.7); Lu 19.2 (16.5). **X-Ray:** In a glass vial (4 mL), ***1,5-cz*-[Lu(L¹)]**$^+$[TFA]$^-\cdot 0.3TFA\cdot 2.4H_2O$ (1.5 mg; 1.6 µmol; 1.0 equiv.) was dissolved in H_2O (70 µL) followed by addition of aq. $HClO_4$ (1.0 M; 2.0 µL; 2.0 µmol; 1.2 equiv.). The vial was gently heated and vortexed. The vial was left standing inside of a closed larger glass vial (20 mL) filled with *i*-PrOH, yielding single crystals over time.

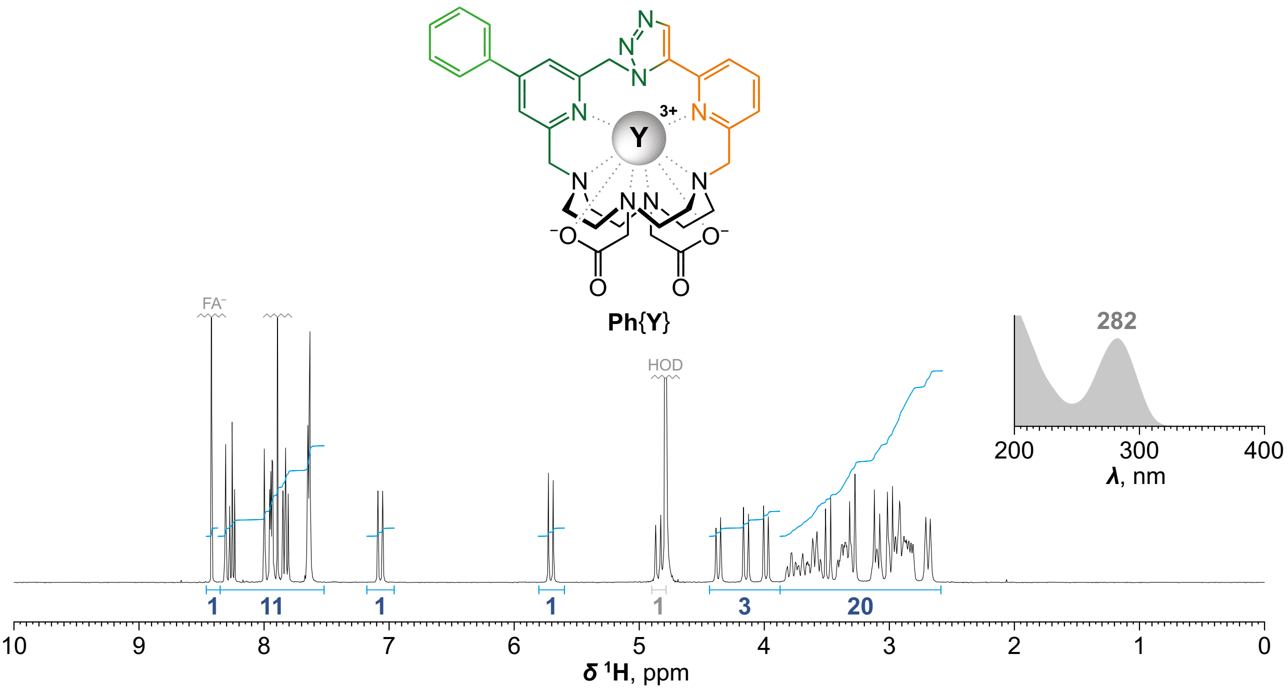

Supplementary Fig. 58. Synthesis and characterization of Ph{Y}. In a glass vial (20 mL), **PhL¹**·0.2FA·0.9H_2O (38 mg; 59 µmol; 1.0 equiv.) was dissolved in H_2O (16 mL) followed by addition aq. MOPS/NaOH buffer (500 mM; pH 7.0; 2.96 mL; 1.48 mmol; 25 equiv.) and aq. YCl_3 (100 mM; 650 µL; 65 µmol; 1.1 equiv.) and the mixture was stirred for 20 h at 80 °C. Mixture was then filtered through syringe microfilter (RC). The filtrate was concentrated on rotary evaporator and then directly purified by preparative HPLC (C18; H_2O–MeCN gradient with FA additive). Fractions with product were joined and directly lyophilized to give product in the form of formate salt as white fluffy solid. **Yield:** 32 mg (65%; 1 step; based on **PhL¹**·0.2FA·0.9H_2O). **NMR (D_2O, pD ~5): ¹H** (401.0 MHz) δ_H 2.58–3.86 (CH_2–N, CH_2–CO, m, 16+4H); 3.99 (CH_2–arom., d, 1H, $^2J_{HH}$ = 14.8); 4.15 (CH_2–arom., d, 1H, $^2J_{HH}$ = 16.2); 4.37 (CH_2–arom., d, 1H, $^2J_{HH}$ = 14.8); 4.85 (CH_2–arom., d, 1H, $^2J_{HH}$ = 16.2); 5.71 (CH_2–N_3, d, 1H, $^2J_{HH}$ = 15.5); 7.07 (CH_2–N_3, d, 1H, $^2J_{HH}$ = 15.5); 7.58–7.68 (C*H*, m, 3H); 7.78–7.86 (C*H*, m, 2H); 7.89 (C*H*–N_3, s, 1H); 7.91–7.97 (C*H*, m, 2H); 7.99 (C*H*, d, 1H, $^4J_{HH}$ = 1.6); 8.26 (C*H*, t, 1H, $^3J_{HH}$ = 7.9); 8.31 (C*H*, d, 1H, $^4J_{HH}$ = 1.6); 8.42 (*FA*⁻, s, 1H). **¹³C{¹H}** (100.8 MHz) δ_C 49.0 (*C*H_2–N, s); 49.2 (*C*H_2–N, s); 51.4 (*C*H_2–N, s); 51.7 (*C*H_2–N, s); 53.0 (*C*H_2–N_3, s); 53.9 (*C*H_2–N, s); 54.1 (*C*H_2–N, s); 54.7 (*C*H_2–N, s); 55.7 (*C*H_2–N, s); 59.5 (*C*H_2–CO, s); 59.6 (*C*H_2–arom., s); 59.8 (*C*H_2–arom., s); 61.4 (*C*H_2–CO, s); 123.9 (*C*H, s); 126.5 (*C*H, s); 127.6 (*C*H, s); 128.4 (*C*H, s); 130.1 (*C*H, s); 130.3 (*C*H, s); 131.8 (*C*H, s); 136.2 (*C*, s); 136.8 (*C*H–N_3, s); 138.2 (*C*H, s); 142.8 (*C*, s); 146.0 (*C*, s); 155.0 (*C*, s); 155.6 (*C*, s); 158.6 (*C*, s); 158.7 (*C*, s); 171.2 (*FA*⁻, s); 177.7 (*C*O, s); 179.0 (*C*O, s). **ESI-HRMS:** 712.2017 $[M]^+$ (theor. $[C_{33}H_{37}N_9O_4Y_1]^+$ = 712.2022). **UV absorption:** λ_{max} = 282 nm. **EA** ($[C_{33}H_{37}N_9O_4Y_1]^+[FA]^-\cdot 4.5H_2O$, M_R = 838.7): C 48.7 (48.6); H 5.6 (5.2); N 15.0 (14.7); Y 10.6 (10.3). **X-Ray:** In a glass vial (4 mL), $[$**Ph{Y}**$]^+[FA]^-\cdot 4.5H_2O$ (1.4 mg; 1.7 µmol; 1.0 equiv.) was dissolved in H_2O (100 µL) followed by addition of aq. $HClO_4$ (1.0 M; 2.0 µL; 2.0 µmol; 1.2 equiv.). The vial was gently heated and vortexed. The vial was left standing inside of a closed larger glass vial (20 mL) filled with *i*-PrOH, yielding single crystals over time.

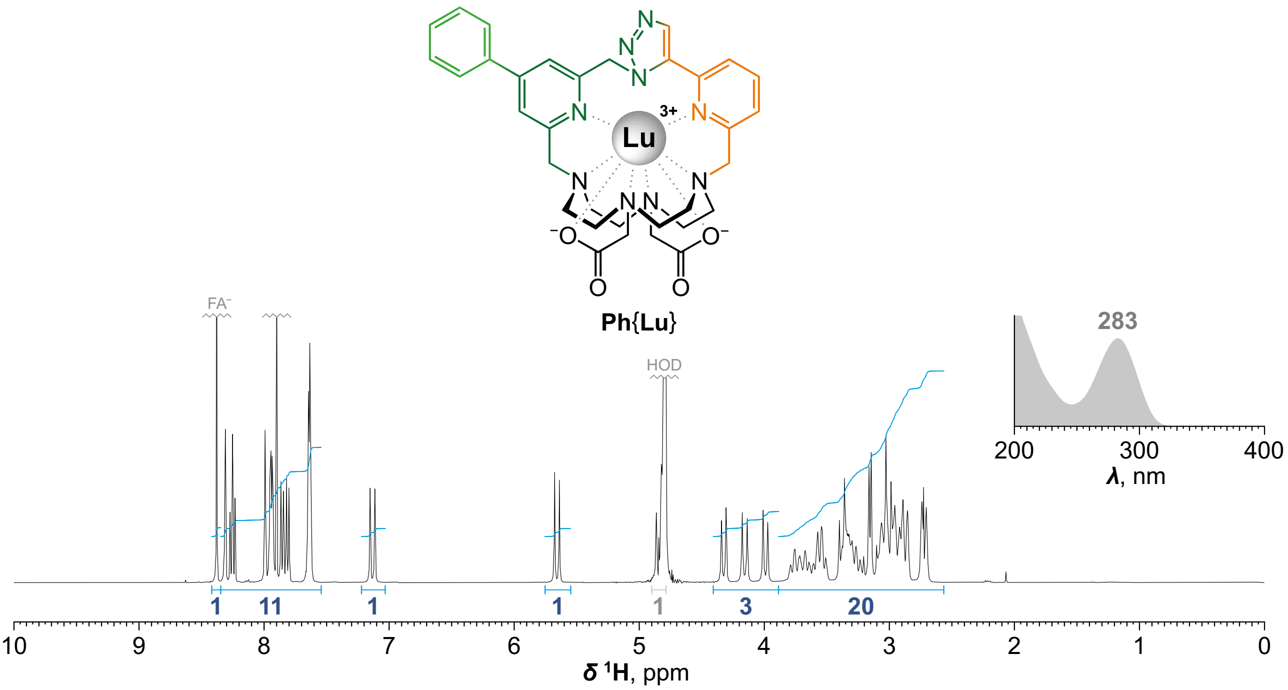

Supplementary Fig. 59. Synthesis and characterization of Ph{Lu}. In a glass vial (20 mL), **PhL[1]**·1.3TFA (30.0 mg; 38.8 µmol; 1.0 equiv.) was dissolved in H_2O (17 mL) followed by addition aq. MOPS/NaOH buffer (500 mM; pH 7.0; 2.30 mL; 1.15 mmol; 30 equiv.) and aq. $LuCl_3$ (100 mM; 500 µL; 50.0 µmol; 1.3 equiv.) and the mixture was stirred for 4 h at 80 °C. Mixture was then filtered through syringe microfilter (RC). The filtrate was concentrated on rotary evaporator and then directly purified by preparative HPLC (C18; H_2O–MeCN gradient with FA additive). Fractions with product were joined and directly lyophilized to give product in the form of formate salt as white fluffy solid. **Yield:** 32.6 mg (89%; 1 step; based on **PhL[1]**·1.3TFA). **NMR (D_2O, pD ~5): ^{1}H** (400.1 MHz) δ_H 2.62–3.83 (CH_2–N, CH_2–CO, m, 16+4H); 3.99 (CH_2–arom., d, 1H, $^2J_{HH}$ = 14.7); 4.16 (CH_2–arom., d, 1H, $^2J_{HH}$ = 15.8); 4.32 (CH_2–arom., d, 1H, $^2J_{HH}$ = 14.7); 4.84 (CH_2–arom., d, 1H, $^2J_{HH}$ = 15.8); 5.66 (CH_2–N_3, d, 1H, $^2J_{HH}$ = 15.3); 7.13 (CH_2–N_3, d, 1H, $^2J_{HH}$ = 15.3); 7.60–7.67 (C*H*, m, 3H); 7.81 (C*H*, dd, 1H, $^3J_{HH}$ = 7.9, $^4J_{HH}$ = 1.1); 7.86 (C*H*, dd, 1H, $^3J_{HH}$ = 7.9, $^4J_{HH}$ = 1.1); 7.90 (C*H*–N_3, s, 1H); 7.91–7.97 (C*H*, m, 2H); 7.99 (C*H*, d, 1H, $^4J_{HH}$ = 1.6); 8.25 (C*H*, t, 1H, $^3J_{HH}$ = 7.9); 8.31 (C*H*, d, 1H, $^4J_{HH}$ = 1.6); 8.38 (*FA*$^-$, s, 1H). **^{13}C{^{1}H}** (100.8 MHz) δ_C 48.9 (CH_2–N, bs); 49.1 (CH_2–N, bs); 51.8 (CH_2–N, bs); 51.9 (CH_2–N, bs); 52.9 (CH_2–N_3, s); 53.8 (CH_2–N, s); 53.9 (CH_2–N, bs); 54.9 (CH_2–N, s); 56.0 (CH_2–N, s); 59.2 (CH_2–CO, s); 59.6 (CH_2–arom., s); 59.7 (CH_2–arom., s); 61.0 (CH_2–CO, s); 123.7 (*C*H, s); 126.5 (*C*H, s); 127.7 (*C*H, s); 128.3 (*C*H, s); 130.3 (*C*H, s); 130.5 (*C*H, s); 131.8 (*C*H, s); 136.0 (*C*, s); 136.4 (*C*H–N_3, s); 138.4 (*C*, s); 142.6 (*C*H, s); 146.4 (*C*, s); 154.7 (*C*, s); 156.0 (*C*, s); 158.7 (*C*, s); 158.8 (*C*, s); 170.0 (*FA*$^-$, s); 177.9 (*C*O, s); 179.4 (*C*O, s). **ESI-HRMS:** 798.2377 $[M]^+$ (theor. $[C_{33}H_{37}N_9O_4Lu_1]^+$ = 798.2371). **UV absorption:** λ_{max} = 283 nm. **EA** ($[C_{33}H_{37}N_9O_4Lu_1]^+[FA]^-\cdot5.5H_2O$, M_R = 942.8): C 43.3 (43.7); H 5.2 (4.6); N 13.4 (13.1); Lu 18.6 (18.0). **X-Ray:** In a glass vial (2 mL), $[$**Ph{Lu}**$]^+[FA]^-\cdot5.5H_2O$ (5.1 mg; 5.4 µmol; 1.0 equiv.) was dissolved in H_2O (230 µL) followed by addition of aq. $HClO_4$ (1.0 M; 6.2 µL; 6.2 µmol; 1.2 equiv.). Immediately formed precipitate was dissolved by gentle heating and vortexing of the vial. Clear solution was allowed to cool to RT and *i*-PrOH was added until cloudiness appeared. Clear solution was again achieved by gentle heating and vortexing of the vial, which was then left standing, yielding single crystals over time.

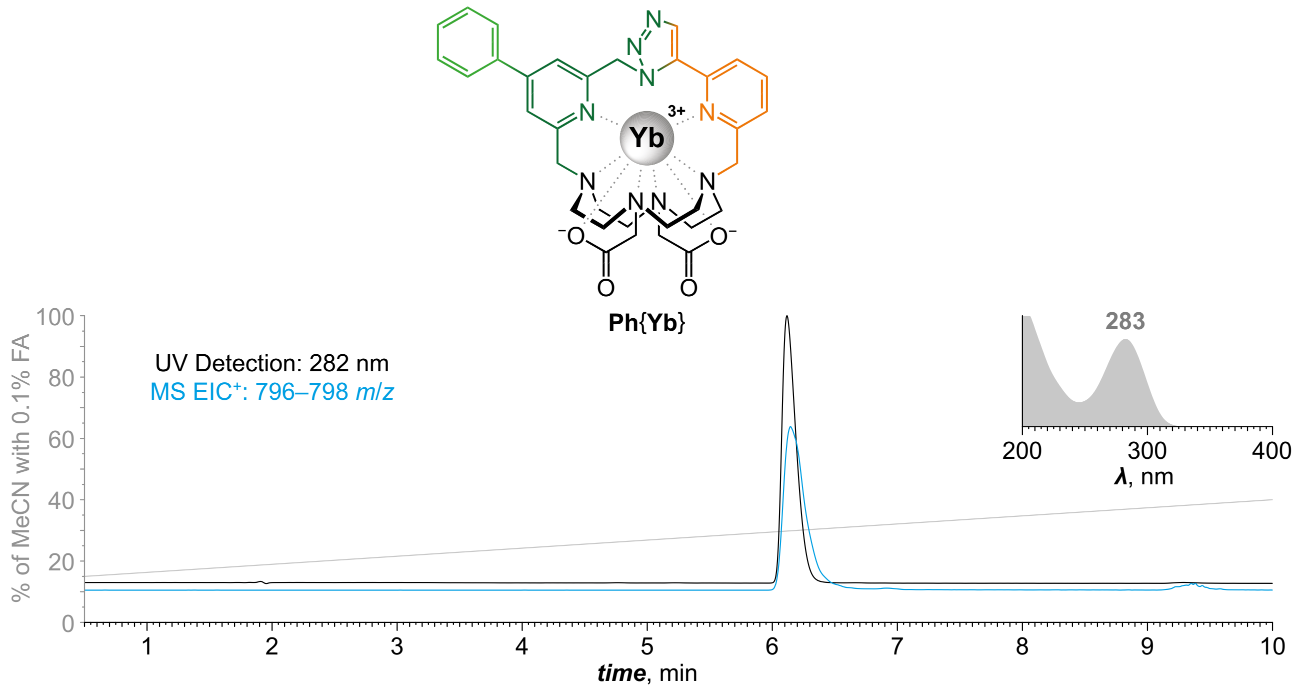

Supplementary Fig. 60. Synthesis and characterization of Ph{Yb}. In a glass vial (20 mL), **PhL**[1]·1.3H_2O (49 mg; 75 µmol; 1.0 equiv.) was dissolved in H_2O (15 mL) followed by addition aq. MOPS/NaOH buffer (500 mM; pH 7.0; 3.75 mL; 1.88 mmol; 25 equiv.) and aq. $YbCl_3$ (100 mM; 825 µL; 83 µmol; 1.1 equiv.) and the mixture was stirred for 20 h at 80 °C. Mixture was then filtered through syringe microfilter (RC). The filtrate was concentrated on rotary evaporator and then directly purified by preparative HPLC (C18; H_2O–MeCN gradient with FA additive). Fractions with product were joined and directly lyophilized to give product in the form of formate salt as white fluffy solid. **Yield:** 53 mg (77%; 1 step; based on **PhL**[1]·1.3H_2O). **ESI-HRMS:** 797.2347 $[M]^+$ (theor. $[C_{33}H_{37}N_9O_4Yb_1]^+$ = 797.2352). **UV absorption:** λ_{max} = 283 nm. **EA** ($[C_{33}H_{37}N_9O_4Yb_1]^+[FA]^-\cdot 4.2H_2O$, M_R = 917.4): C 44.5 (44.1); H 5.1 (5.1); N 13.7 (13.3); Yb 18.4 (18.7). **X-Ray:** In a glass vial (4 mL), ***1,5-cz*-[Ph{Yb}]**$^+[FA]^-\cdot 4.2H_2O$ (1.4 mg; 1.6 µmol; 1.0 equiv.) was dissolved in H_2O (100 µL) followed by addition of aq. $HClO_4$ (1.0 M; 2.0 µL; 2.0 µmol; 1.2 equiv.). The vial was gently heated and vortexed. The vial was left standing inside of a closed larger glass vial (20 mL) filled with *i*-PrOH, yielding single crystals over time.

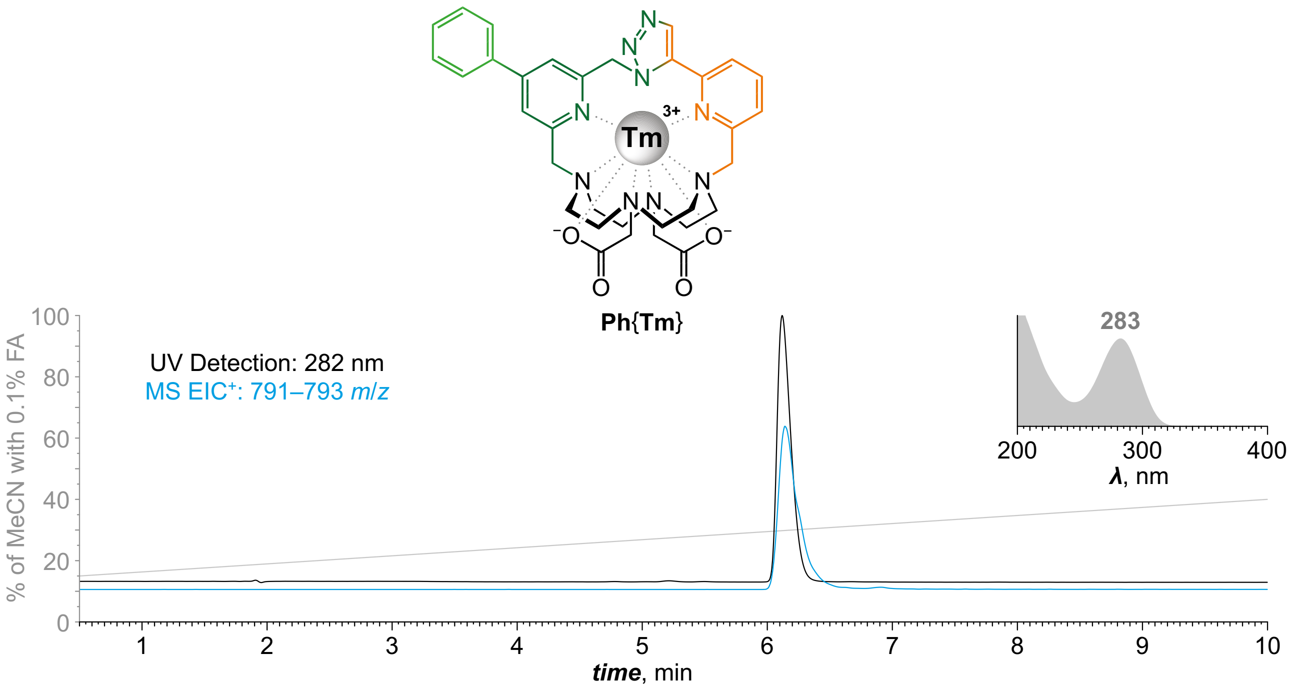

Supplementary Fig. 61. Synthesis and characterization of Ph{Tm}. In a glass vial (20 mL), **PhL[1]**·1.3H_2O (49 mg; 75 µmol; 1.0 equiv.) was dissolved in H_2O (15 mL) followed by addition aq. MOPS/NaOH buffer (500 mM; pH 7.0; 3.75 mL; 1.88 mmol; 25 equiv.) and aq. $TmCl_3$ (100 mM; 825 µL; 83 µmol; 1.1 equiv.) and the mixture was stirred for 20 h at 80 °C. Mixture was then filtered through syringe microfilter (RC). The filtrate was concentrated on rotary evaporator and then directly purified by preparative HPLC (C18; H_2O–MeCN gradient with FA additive). Fractions with product were joined and directly lyophilized to give product in the form of formate salt as white fluffy solid. **Yield:** 51 mg (73%; 1 step; based on **PhL[1]**·1.3H_2O). **ESI-HRMS:** 792.2299 $[M]^+$ (theor. $[C_{33}H_{37}N_9O_4Tm_1]^+$ = 792.2305). **UV absorption:** λ_{max} = 283 nm. **EA** ($[C_{33}H_{37}N_9O_4Tm_1]^+[FA]^-\cdot 4.7H_2O$, M_R = 922.3): C 44.3 (44.4); H 5.2 (5.1); N 13.7 (13.5); Tm 18.3 (18.3). **X-Ray:** In a glass vial (4 mL), $[$**Ph**{**Tm**}$]^+[FA]^-\cdot 4.7H_2O$ (1.5 mg; 1.6 µmol; 1.0 equiv.) was dissolved in H_2O (100 µL) followed by addition of aq. $HClO_4$ (1.0 M; 2.0 µL; 2.0 µmol; 1.2 equiv.). The vial was gently heated and vortexed. The vial was left standing inside of a closed larger glass vial (20 mL) filled with *i*-PrOH, yielding single crystals over time.

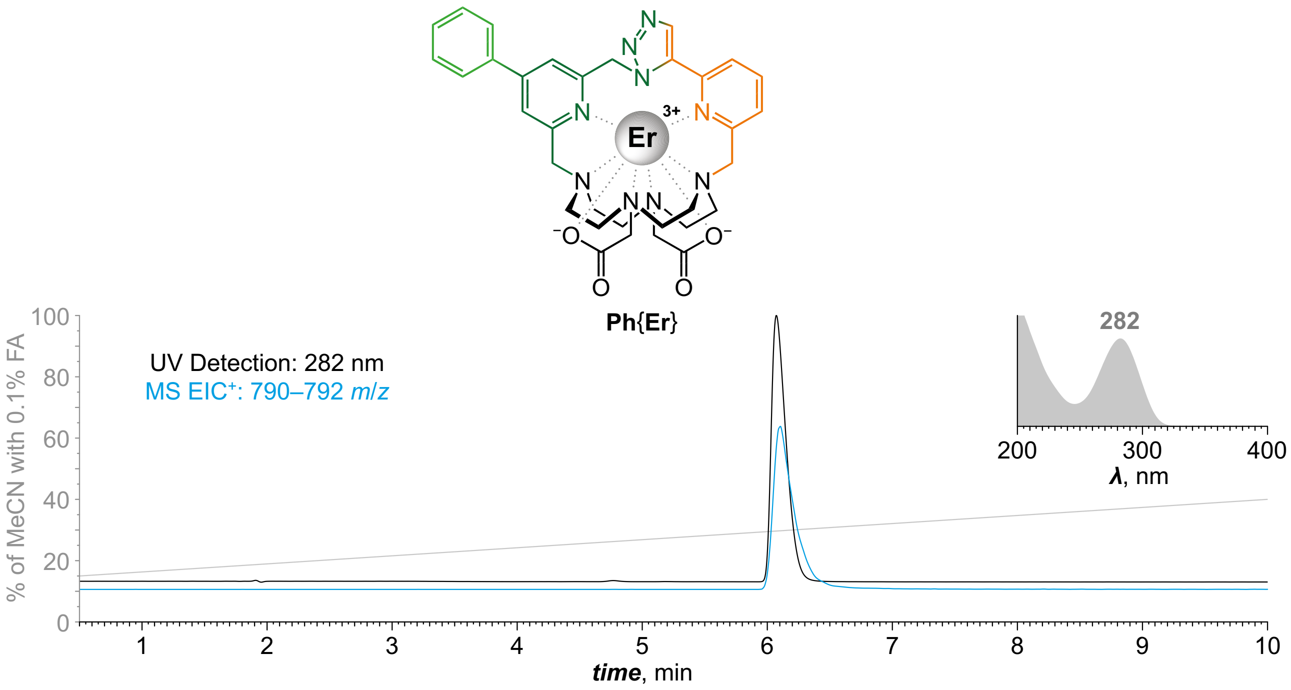

Supplementary Fig. 62. Synthesis and characterization of Ph{Er}. In a glass vial (20 mL), **PhL^{1}**·1.3TFA (20.5 mg; 26.5 µmol; 1.0 equiv.) was dissolved in H_2O (17 mL) followed by addition aq. MOPS/NaOH buffer (500 mM; pH 7.0; 1.84 mL; 920 µmol; 35 equiv.) and aq. $ErCl_3$ (100 mM; 370 µL; 37.0 µmol; 1.4 equiv.) and the mixture was stirred for 22 h at 80 °C. Mixture was then filtered through syringe microfilter (RC). The filtrate was concentrated on rotary evaporator and then directly purified by preparative HPLC (C18; H_2O–MeCN gradient with FA additive). Fractions with product were joined and directly lyophilized to give product in the form of formate salt as white fluffy solid. **Yield:** 22.8 mg (90%; 1 step; based on **PhL^{1}**·1.3TFA). **ESI-HRMS:** 791.2280 $[M]^+$ (theor. $[C_{33}H_{37}N_9O_4Er_1]^+$ = 791.2302). **UV absorption:** λ_{max} = 282 nm. **EA** ($[C_{33}H_{37}N_9O_4Er_1]^+[FA]^-\cdot 6.9H_2O$, M_R = 960.3): C 42.5 (42.4); H 5.4 (4.9); N 13.1 (12.7); Er 17.4 (16.8). **X-Ray:** In a glass vial (4 mL), [**Ph**{**Er**}]$^+$[FA]$^-$·6.9H_2O (1.6 mg; 1.7 µmol; 1.0 equiv.) was dissolved in H_2O (100 µL) followed by addition of aq. $HClO_4$ (1.0 M; 2.0 µL; 2.0 µmol; 1.2 equiv.). The vial was gently heated and vortexed. The vial was left standing inside of a closed larger glass vial (20 mL) filled with *i*-PrOH, yielding single crystals over time.

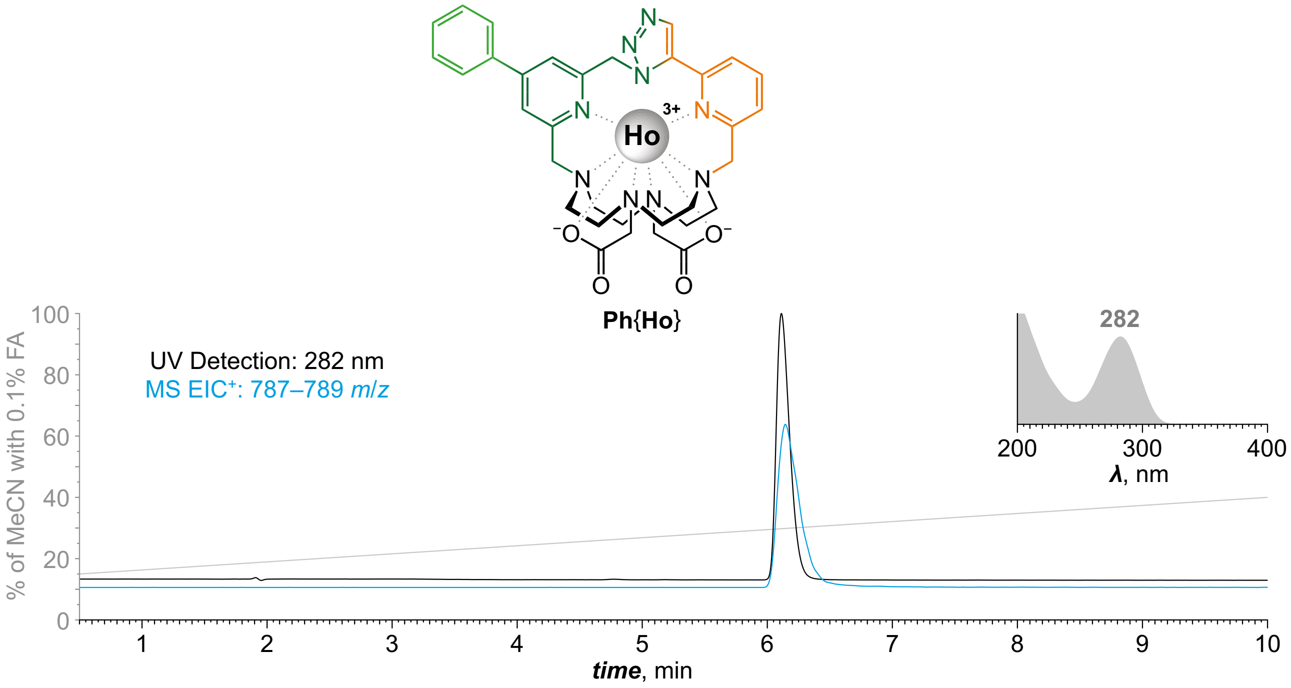

Supplementary Fig. 63. Synthesis and characterization of Ph{Ho}. In a glass vial (20 mL), **PhL[1]**·0.2FA·0.9H_2O (22 mg; 34 µmol; 1.0 equiv.) was dissolved in H_2O (17 mL) followed by addition aq. MOPS/NaOH buffer (500 mM; pH 7.0; 1.72 mL; 860 µmol; 25 equiv.) and aq. $HoCl_3$ (100 mM; 375 µL; 38 µmol; 1.1 equiv.) and the mixture was stirred for 20 h at 80 °C. Mixture was then filtered through syringe microfilter (RC). The filtrate was concentrated on rotary evaporator and then directly purified by preparative HPLC (C18; H_2O–MeCN gradient with FA additive). Fractions with product were joined and directly lyophilized to give product in the form of formate salt as white fluffy solid. **Yield:** 24 mg (76%; 1 step; based on **PhL[1]**·0.2FA·0.9H_2O). **ESI-HRMS:** 788.2265 $[M]^+$ (theor. $[C_{33}H_{37}N_9O_4Ho_1]^+$ = 788.2266). **UV absorption:** λ_{max} = 282 nm. **EA** ($[C_{33}H_{37}N_9O_4Ho_1]^+[FA]^-\cdot 5.0H_2O$, M_R = 923.7): C 44.2 (44.2); H 5.2 (4.8); N 13.6 (13.3); Ho 17.9 (17.3). **X-Ray:** In a glass vial (4 mL), [**Ph**{**Ho**}]$^+$[FA]$^-\cdot$5.0H_2O (1.5 mg; 1.6 µmol; 1.0 equiv.) was dissolved in H_2O (100 µL) followed by addition of aq. $HClO_4$ (1.0 M; 2.0 µL; 2.0 µmol; 1.2 equiv.). The vial was gently heated and vortexed. The vial was left standing inside of a closed larger glass vial (20 mL) filled with *i*-PrOH, yielding single crystals over time.

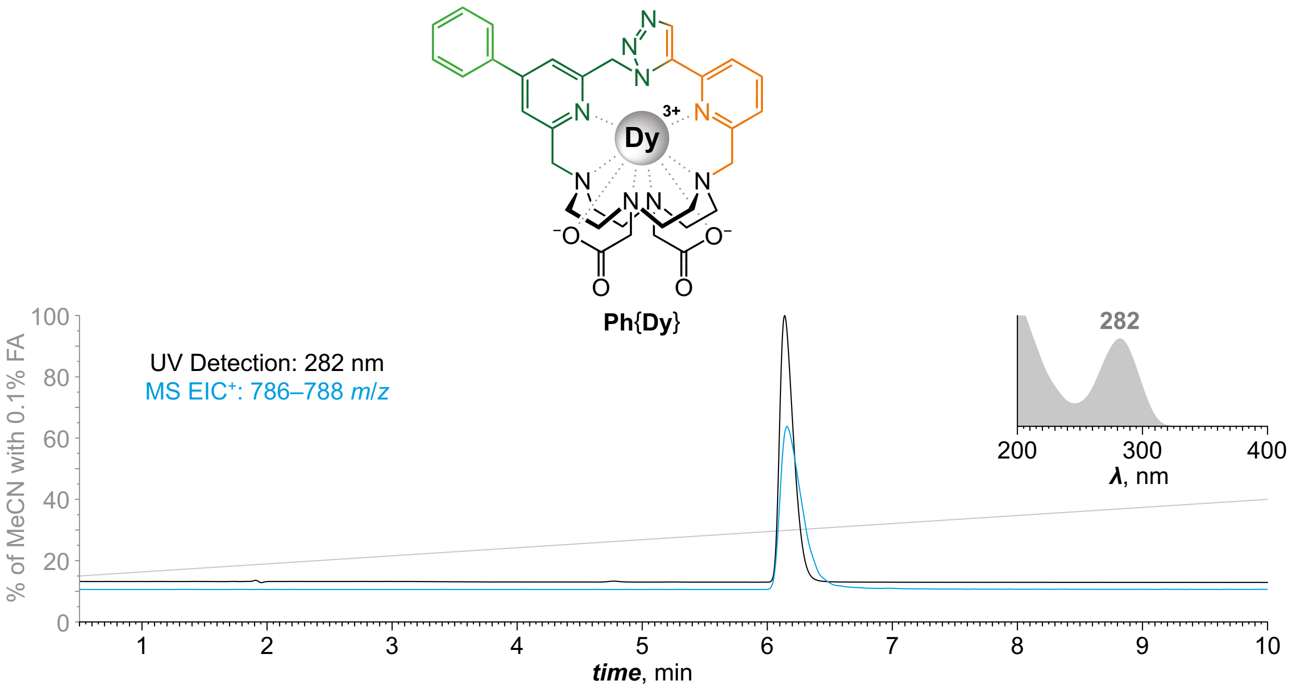

Supplementary Fig. 64. Synthesis and characterization of Ph{Dy}. In a glass vial (20 mL), **PhL[1]**·1.3TFA (20.0 mg; 25.8 µmol; 1.0 equiv.) was dissolved in H_2O (17 mL) followed by addition aq. MOPS/NaOH buffer (500 mM; pH 7.0; 1.84 mL; 920 µmol; 36 equiv.) and aq. $DyCl_3$ (100 mM; 370 µL; 37.0 µmol; 1.4 equiv.) and the mixture was stirred for 46 h at 80 °C. Mixture was then filtered through syringe microfilter (RC). The filtrate was concentrated on rotary evaporator and then directly purified by preparative HPLC (C18; H_2O–MeCN gradient with FA additive). Fractions with product were joined and directly lyophilized to give product in the form of formate salt as white fluffy solid. **Yield:** 22.2 mg (89%; 1 step; based on **PhL[1]**·1.3TFA). **ESI-HRMS:** 787.2248 $[M]^+$ (theor. $[C_{33}H_{37}N_9O_4Dy_1]^+ = 787.2255$). **UV absorption:** $\lambda_{max} = 282$ nm. **EA** ($[C_{33}H_{37}N_9O_4Dy_1]^+[FA]^-\cdot 7.7H_2O$, $M_R = 970.0$): C 42.1 (42.5); H 5.5 (5.0); N 13.0 (12.6); Dy 16.8 (16.1). **X-Ray:** In a glass vial (4 mL), $[$**Ph{Dy}**$]^+[FA]^-\cdot 7.7H_2O$ (1.6 mg; 1.6 µmol; 1.0 equiv.) was dissolved in H_2O (100 µL) followed by addition of aq. $HClO_4$ (1.0 M; 2.0 µL; 2.0 µmol; 1.2 equiv.). The vial was gently heated and vortexed. The vial was left standing inside of a closed larger glass vial (20 mL) filled with *i*-PrOH, yielding single crystals over time.

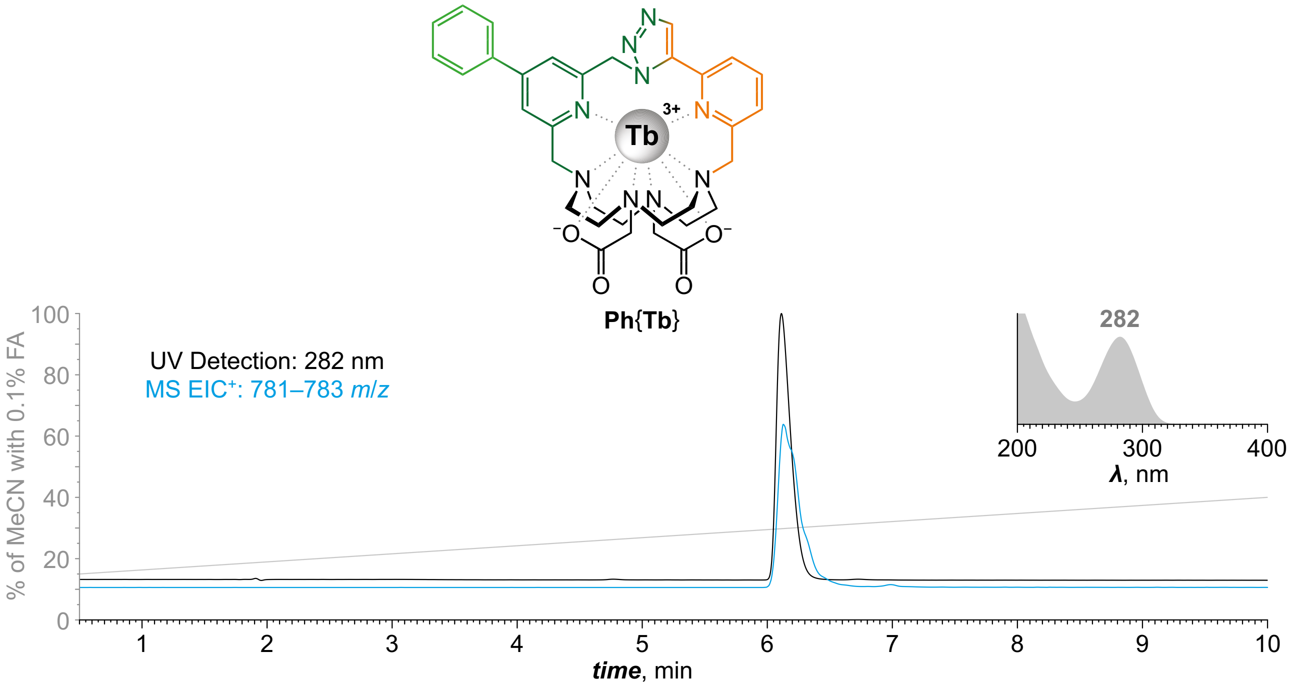

Supplementary Fig. 65. Synthesis and characterization of Ph{Tb}. In a glass vial (20 mL), **PhL[1]**·1.3H_2O (50 mg; 77 µmol; 1.0 equiv.) was dissolved in H_2O (15 mL) followed by addition aq. MOPS/NaOH buffer (500 mM; pH 7.0; 3.75 mL; 1.88 mmol; 24 equiv.) and aq. $TbCl_3$ (100 mM; 825 µL; 83 µmol; 1.1 equiv.) and the mixture was stirred for 3 d at 80 °C. Mixture was then filtered through syringe microfilter (RC). The filtrate was concentrated on rotary evaporator and then directly purified by preparative HPLC (C18; H_2O–MeCN gradient with FA additive). Fractions with product were joined and directly lyophilized to give product in the form of formate salt as white fluffy solid. **Yield:** 49 mg (69%; 1 step; based on **PhL[1]**·1.3H_2O). **ESI-HRMS:** 782.2211 $[M]^+$ (theor. $[C_{33}H_{37}N_9O_4Tb_1]^+$ = 781.2217). **UV absorption:** λ_{max} = 282 nm. **EA** ($[C_{33}H_{37}N_9O_4Tb_1]^+[FA]^-$·4.9$H_2O$, M_R = 915.9): C 44.6 (44.7); H 5.3 (5.1); N 13.8 (13.6); Tb 17.4 (17.1). **X-Ray:** In a glass vial (4 mL), $[$**Ph**{**Tb**}$]^+[FA]^-$·4.9H_2O (1.5 mg; 1.6 µmol; 1.0 equiv.) was dissolved in H_2O (100 µL) followed by addition of aq. $HClO_4$ (1.0 M; 2.0 µL; 2.0 µmol; 1.2 equiv.). The vial was gently heated and vortexed. The vial was left standing inside of a closed larger glass vial (20 mL) filled with *i*-PrOH, yielding single crystals over time.

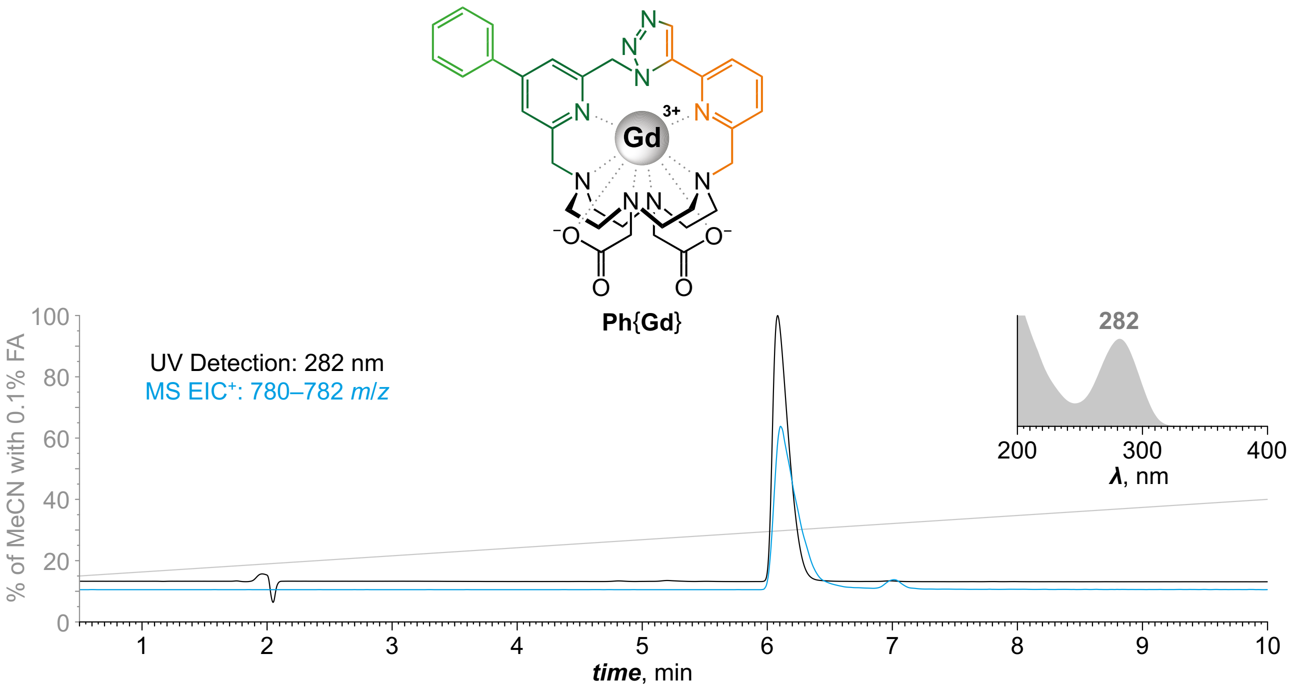

Supplementary Fig. 66. Synthesis and characterization of Ph{Gd}. In a glass vial (40 mL), **PhL^{1}**·2.2H_2O (75 mg; 113 µmol; 1.0 equiv.) was dissolved in H_2O (32 mL) followed by addition aq. MOPS/NaOH buffer (500 mM; pH 7.0; 4.60 mL; 2.30 mmol; 20 equiv.) and aq. $GdCl_3$ (100 mM; 1.20 mL; 120 µmol; 1.1 equiv.) and the mixture was stirred for 2 d at 80 °C. Mixture was then filtered through syringe microfilter (RC). The filtrate was concentrated on rotary evaporator and then directly purified by preparative HPLC (C18; H_2O–MeCN gradient with FA additive). Fractions with product were joined and directly lyophilized to give product in the form of formate salt as white fluffy solid. **Yield:** 58 mg (56%; 1 step; based on **PhL^{1}**·2.2H_2O). **ESI-HRMS:** 781.2218 $[M]^+$ (theor. $[C_{33}H_{37}N_9O_4Gd_1]^+ = 781.2204$). **UV absorption:** $\lambda_{max} = 282$ nm. **EA** ($[C_{33}H_{37}N_9O_4Gd_1]^+[FA]^-\cdot 5.3H_2O$, $M_R = 921.5$): C 44.3 (44.7); H 5.3 (4.7); N 13.7 (13.4); Gd 17.1 (16.2). **X-Ray:** In a glass vial (2 mL), [**Ph**{**Gd**}]$^+$[FA]$^-$·5.3H_2O (3.0 mg; 3.3 µmol; 1.0 equiv.) was dissolved in H_2O (128 µL) followed by addition of aq. $HClO_4$ (1.0 M; 6.6 µL; 6.6 µmol; 2.0 equiv.). Immediately formed precipitate was dissolved by gentle heating and vortexing of the vial. Clear solution was allowed to cool to RT and *i*-PrOH was added until cloudiness appeared. The vial was left standing inside of a closed larger glass vial (20 mL) filled with *i*-PrOH, yielding single crystals over time.

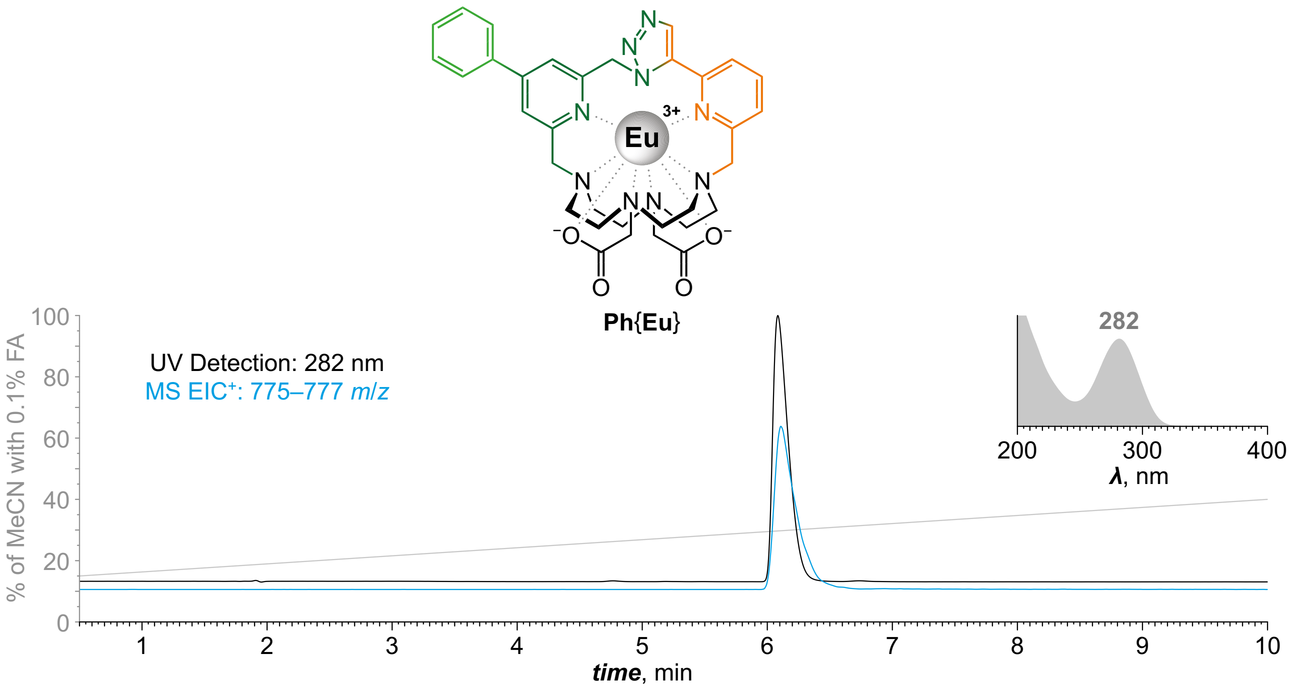

Supplementary Fig. 67. Synthesis and characterization of Ph{Eu}. In a glass vial (20 mL), **PhL¹**·1.3H_2O (50 mg; 77 µmol; 1.0 equiv.) was dissolved in H_2O (15 mL) followed by addition aq. MOPS/NaOH buffer (500 mM; pH 7.0; 3.75 mL; 1.88 mmol; 24 equiv.) and aq. $EuCl_3$ (100 mM; 825 µL; 83 µmol; 1.1 equiv.) and the mixture was stirred for 3 d at 80 °C. Mixture was then filtered through syringe microfilter (RC). The filtrate was concentrated on rotary evaporator and then directly purified by preparative HPLC (C18; H_2O–MeCN gradient with FA additive). Fractions with product were joined and directly lyophilized to give product in the form of formate salt as white fluffy solid. **Yield:** 44 mg (63%; 1 step; based on **PhL¹**·1.3H_2O). **ESI-HRMS:** 776.2171 $[M]^+$ (theor. $[C_{33}H_{37}N_9O_4Eu_1]^+$ = 776.2175). **UV absorption:** λ_{max} = 282 nm. **EA** ($[C_{33}H_{37}N_9O_4Eu_1]^+[FA]^-$·4.6$H_2O$, M_R = 903.6): C 45.2 (45.3); H 5.3 (5.1); N 14.0 (13.7); Eu 16.8 (16.5). **X-Ray:** In a glass vial (4 mL), $[$**Ph{Eu}**$]^+[FA]^-$·4.6H_2O (1.6 mg; 1.8 µmol; 1.0 equiv.) was dissolved in H_2O (75 µL) followed by addition of aq. $HClO_4$ (1.0 M; 2.0 µL; 2.0 µmol; 1.1 equiv.). The vial was gently heated and vortexed. The vial was left standing inside of a closed larger glass vial (20 mL) filled with *i*-PrOH, yielding single crystals over time.

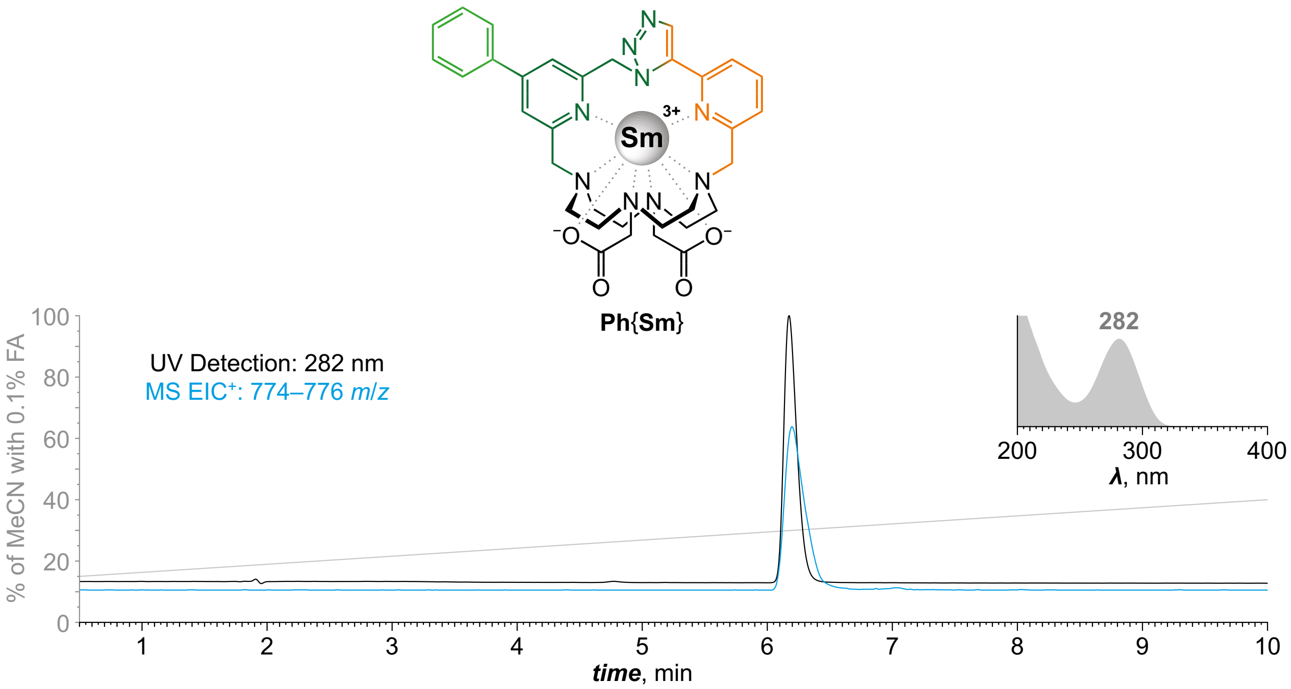

Supplementary Fig. 68. Synthesis and characterization of Ph{Sm}. In a glass vial (20 mL), **PhL¹**·1.3TFA (25.6 mg; 33.1 µmol; 1.0 equiv.) was dissolved in H_2O (17 mL) followed by addition aq. MOPS/NaOH buffer (500 mM; pH 7.0; 2.30 mL; 1.15 mmol; 35 equiv.) and aq. $SmCl_3$ (100 mM; 460 µL; 46.0 µmol; 1.4 equiv.) and the mixture was stirred for 12 d at 80 °C. Mixture was then filtered through syringe microfilter (RC). The filtrate was concentrated on rotary evaporator and then directly purified by preparative HPLC (C18; H_2O–MeCN gradient with FA additive). Fractions with product were joined and directly lyophilized to give product in the form of formate salt as white fluffy solid. **Yield:** 22.3 mg (73%; 1 step; based on **PhL¹**·1.3TFA). **ESI-HRMS:** 775.2158 $[M]^+$ (theor. $[C_{33}H_{37}N_9O_4Sm_1]^+$ = 775.2160). **UV absorption:** λ_{max} = 282 nm. **EA** ($[C_{33}H_{37}N_9O_4Sm_1]^+[FA]^-\cdot5.8H_2O$, M_R = 923.6): C 44.2 (44.7); H 5.4 (4.9); N 13.6 (13.3); Nd 16.3 (15.8). **X-Ray:** In a glass vial (4 mL), [**Ph**{**Sm**}]$^+$[FA]$^-\cdot5.8H_2O$ (1.6 mg; 1.7 µmol; 1.0 equiv.) was dissolved in H_2O (75 µL) followed by addition of aq. $HClO_4$ (1.0 M; 2.0 µL; 2.0 µmol; 1.2 equiv.). The vial was gently heated and vortexed. The vial was left standing inside of a closed larger glass vial (20 mL) filled with *i*-PrOH, yielding single crystals over time.

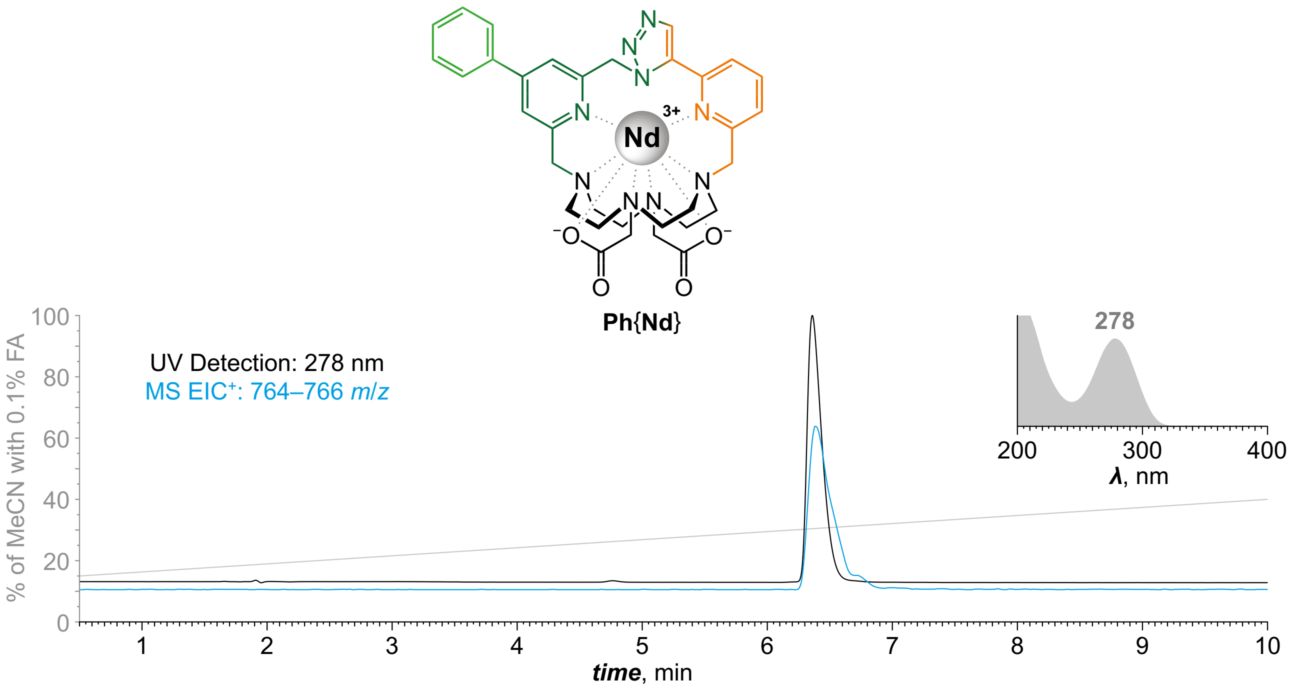

Supplementary Fig. 69. Synthesis and characterization of Ph{Nd}. In a glass vial (20 mL), **PhL[1]**·1.3TFA (25.7 mg; 33.2 µmol; 1.0 equiv.) was dissolved in H_2O (17 mL) followed by addition aq. MOPS/NaOH buffer (500 mM; pH 7.0; 2.30 mL; 1.15 mmol; 35 equiv.) and aq. $NdCl_3$ (100 mM; 460 µL; 46.0 µmol; 1.4 equiv.) and the mixture was stirred for 12 d at 80 °C. Mixture was then filtered through syringe microfilter (RC). The filtrate was concentrated on rotary evaporator and then directly purified by preparative HPLC (C18; H_2O–MeCN gradient with FA additive). Fractions with product were joined and directly lyophilized to give product in the form of formate salt as white fluffy solid. **Yield:** 19.8 mg (65%; 1 step; based on **PhL[1]**·1.3TFA). **ESI-HRMS:** 765.2039 $[M]^+$ (theor. $[C_{33}H_{37}N_9O_4Nd_1]^+$ = 765.2040). **UV absorption:** λ_{max} = 278 nm. **EA** ($[C_{33}H_{37}N_9O_4Nd_1]^+[FA]^-\cdot 5.6H_2O$, M_R = 913.9): C 44.7 (45.0); H 5.4 (4.6); N 13.8 (13.4); Nd 15.8 (15.2).

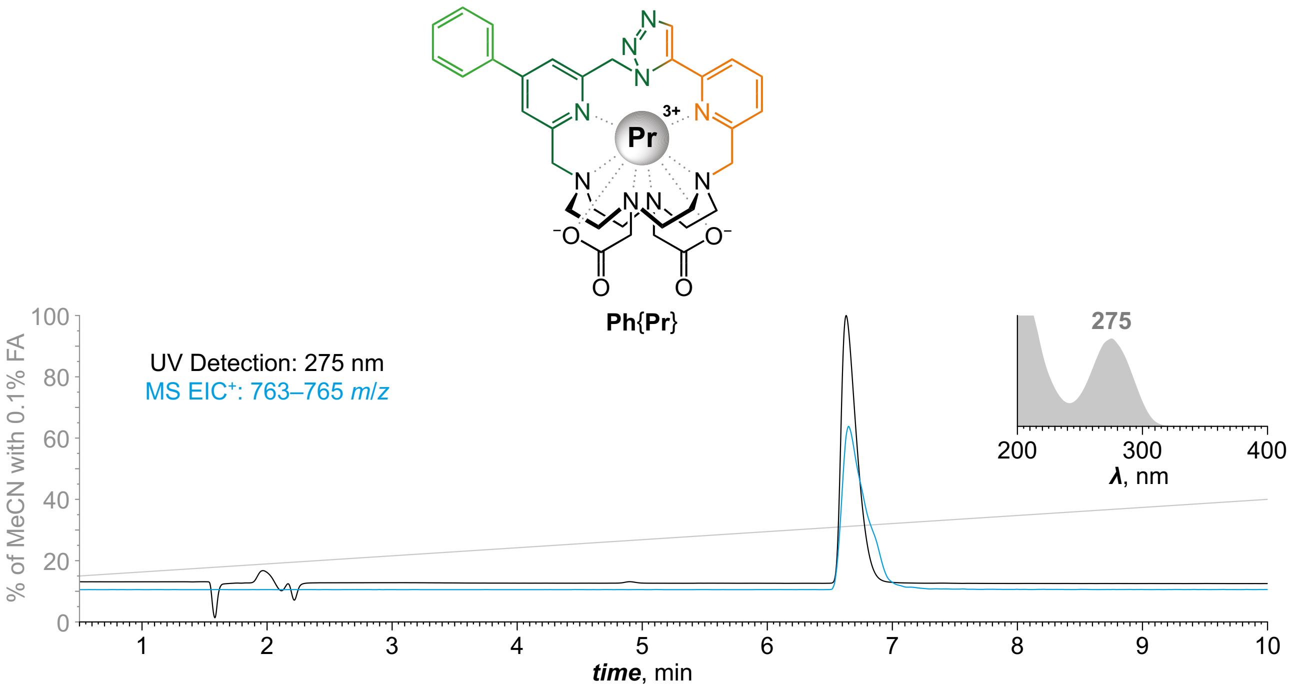

Supplementary Fig. 70. Synthesis and characterization of Ph{Pr}. In a glass vial (20 mL), **PhL**$^{\mathbf{1}}$·1.3TFA (26.4 mg; 34.1 µmol; 1.0 equiv.) was dissolved in H_2O (17 mL) followed by addition aq. MOPS/NaOH buffer (500 mM; pH 7.0; 2.40 mL; 1.20 mmol; 35 equiv.) and aq. $PrCl_3$ (100 mM; 500 µL; 50.0 µmol; 1.5 equiv.) and the mixture was stirred for 14 d at 80 °C. Mixture was then filtered through syringe microfilter (RC). The filtrate was concentrated on rotary evaporator and then directly purified by preparative HPLC (C18; H_2O–MeCN gradient with FA additive). Fractions with product were joined and directly lyophilized to give product in the form of formate salt as white fluffy solid. **Yield:** 17.7 mg (56%; 1 step; based on **PhL**$^{\mathbf{1}}$·1.3TFA). **ESI-HRMS:** 764.2035 $[M]^+$ (theor. $[C_{33}H_{37}N_9O_4Pr_1]^+$ = 764.2040). **UV absorption:** λ_{max} = 275 nm. **EA** ($[C_{33}H_{37}N_9O_4Pr_1]^+[FA]^-\cdot 6.4H_2O$, M_R = 924.9): C 44.2 (44.7); H 5.5 (5.0); N 13.6 (13.1); Pr 15.2 (14.8). **X-Ray:** In a glass vial (4 mL), [**Ph**{**Pr**}]$^+$[FA]$^-$·6.4H_2O (1.0 mg; 1.1 µmol; 1.0 equiv.) was dissolved in H_2O (40 µL) followed by addition of aq. $HClO_4$ (1.0 M; 1.1 µL; 1.1 µmol; 1.0 equiv.). The vial was briefly vortexed. The vial was left standing inside of a closed larger glass vial (20 mL) filled with *i*-PrOH, yielding single crystals over time.

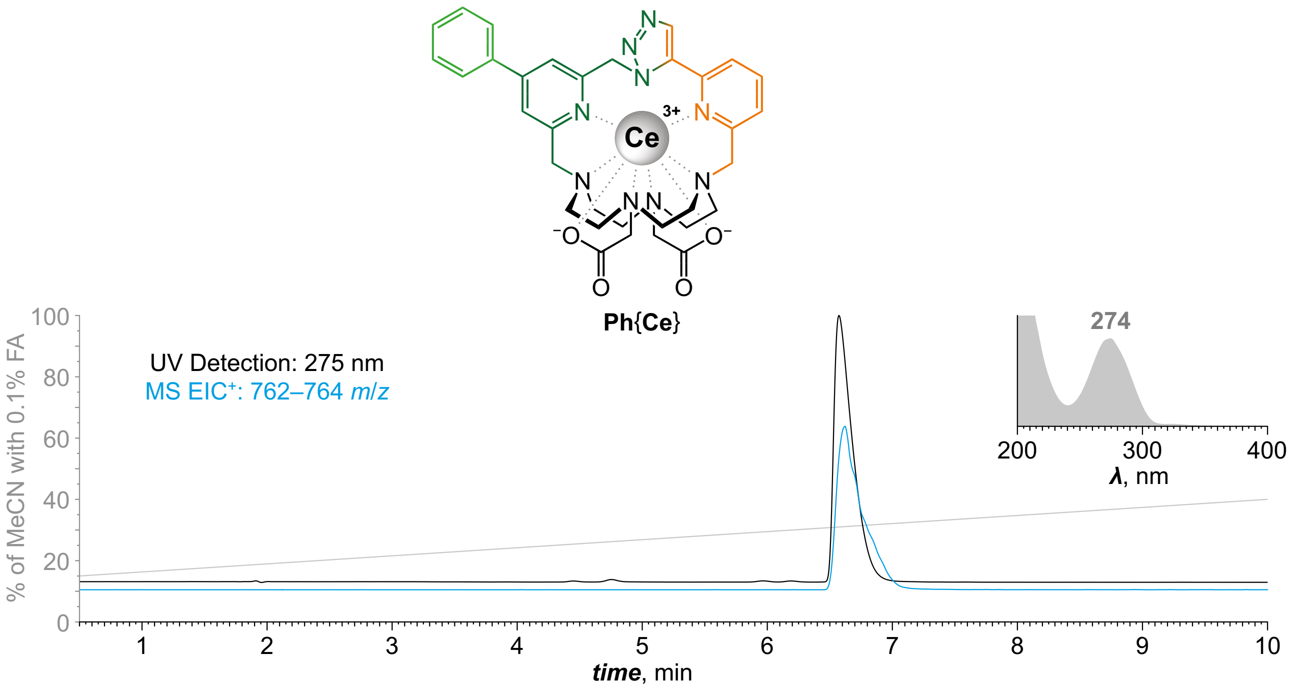

Supplementary Fig. 71. Synthesis and characterization of Ph{Ce}. In a glass vial (20 mL), **PhL^{1}**·1.3TFA (30.5 mg; 39.4 µmol; 1.0 equiv.) was dissolved in H_2O (17 mL) followed by addition aq. MOPS/NaOH buffer (500 mM; pH 7.0; 2.30 mL; 1.15 mmol; 29 equiv.) and aq. $CeCl_3$ (100 mM; 545 µL; 54.5 µmol; 1.4 equiv.) and the mixture was stirred for 14 d at 80 °C. Mixture was then filtered through syringe microfilter (RC). The filtrate was concentrated on rotary evaporator and then directly purified by preparative HPLC (C18; H_2O–MeCN gradient with FA additive). Fractions with product were joined and directly lyophilized to give product in the form of formate salt as white fluffy solid. **Yield:** 21.2 mg (58%; 1 step; based on **PhL^{1}**·1.3TFA). **ESI-HRMS:** 763.2012 $[M]^+$ (theor. $[C_{33}H_{37}N_9O_4Ce_1]^+$ = 763.2017). **UV absorption:** λ_{max} = 274 nm. **EA** ($[C_{33}H_{37}N_9O_4Ce_1]^+[FA]^-\cdot 6.5H_2O$, M_R = 925.9): C 44.1 (44.7); H 5.6 (5.1); N 13.6 (13.1); Ce 15.1 (14.5).

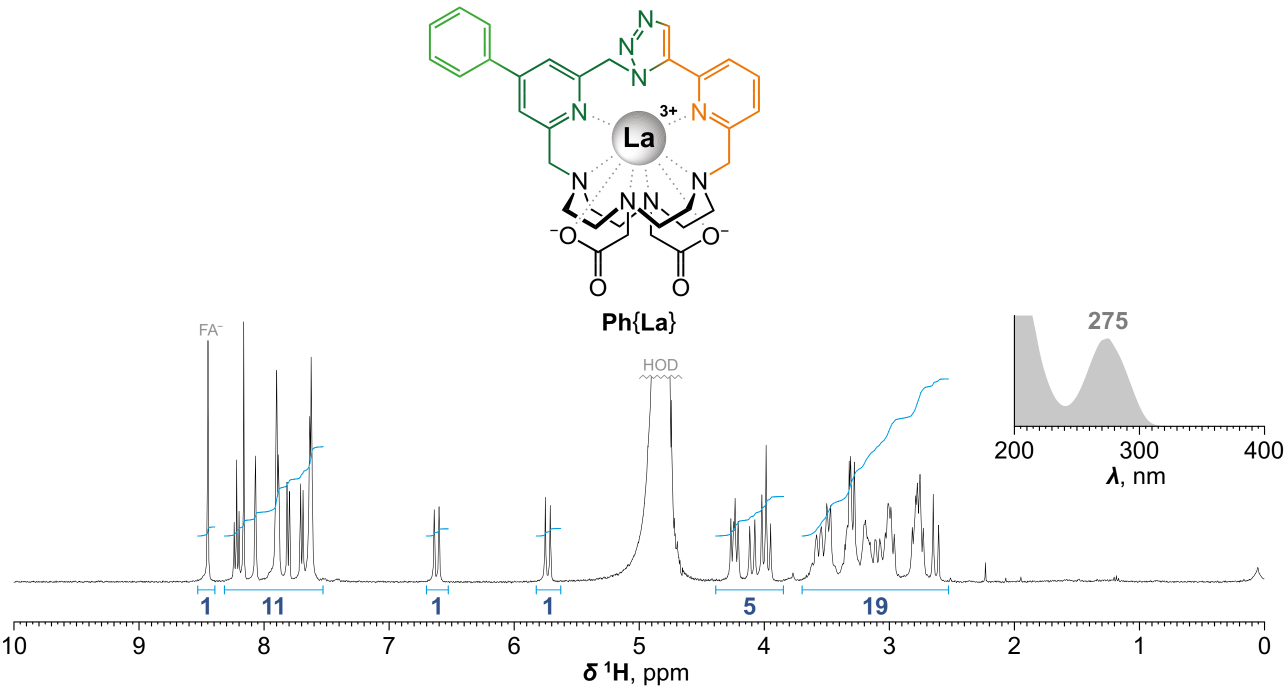

Supplementary Fig. 72. Synthesis and characterization of Ph{La}. In a glass vial (20 mL), **PhL¹**·0.2FA·0.9H_2O (30 mg; 46 µmol; 1.0 equiv.) was dissolved in H_2O (17 mL) followed by addition aq. MOPS/NaOH buffer (500 mM; pH 7.0; 2.30 mL; 1.15 mmol; 25 equiv.) and aq. $LaCl_3$ (100 mM; 500 µL; 50 µmol; 1.1 equiv.) and the mixture was stirred for 14 d at 80 °C. Mixture was then filtered through syringe microfilter (RC). The filtrate was concentrated on rotary evaporator and then directly purified by preparative HPLC (C18; H_2O–MeCN gradient with FA additive). Fractions with product were joined and directly lyophilized to give product in the form of formate salt as white fluffy solid. **Yield:** 15 mg (36%; 1 step; based on **PhL¹**·0.2FA·0.9H_2O). **NMR (D_2O, pD ~5): ¹H** (401.0 MHz) δ_H 2.54–3.63 (CH_2–N, CH_2–CO, m, 16+3H); 3.96 (CH_2–arom., d, 1H, $^2J_{HH}$ = 14.4); 4.00 (CH_2–arom., d, 1H, $^2J_{HH}$ = 14.0); 4.09 (CH_2–CO, 1H, d, $^2J_{HH}$ = 16.6); 4.23 (CH_2–arom., d, 1H, $^2J_{HH}$ = 14.4); 4.25 (CH_2–arom., d, 1H, $^2J_{HH}$ = 14.0); 5.72 (CH_2–N_3, d, 1H, $^2J_{HH}$ = 15.8); 6.62 (CH_2–N_3, d, 1H, $^2J_{HH}$ = 15.8); 7.58–7.66 (C*H*, m, 3H); 7.70 (C*H*, dd, 1H, $^3J_{HH}$ = 7.9, $^4J_{HH}$ = 1.0); 7.81 (C*H*, dd, 1H, $^3J_{HH}$ = 7.9, $^4J_{HH}$ = 1.0); 7.83–7.95 (C*H*, m, 3H); 8.06 (C*H*, d, 1H, $^4J_{HH}$ = 1.7); 8.16 (C*H*–N_3, s, 1H); 8.22 (C*H*, t, 1H, $^3J_{HH}$ = 7.9); 8.42 (*FA*⁻, s, 1H). **¹³C{¹H}** (100.8 MHz) δ_C 53.5 (CH_2–N_3, s); 56.3 (CH_2–N, s); 58.7 (CH_2–N, s); 58.9 (CH_2–N, s); 59.2 (CH_2–N, s); 59.3 (CH_2–N, s); 60.0 (CH_2–N, s); 61.9 (CH_2–N, s); 62.5 (CH_2–CO, s); 63.5 (CH_2–N, s); 65.1 (CH_2–CO, s); 65.2 (CH_2–arom., s); 66.6 (CH_2–arom., s); 124.1 (*C*H, s); 124.6 (*C*H, s); 127.1 (*C*H, s); 127.9 (*C*H, s); 128.1 (*C*H, s); 130.2 (*C*H, s); 131.5 (*C*H, s); 136.1 (*C*H, s); 136.2 (*C*, s); 136.8 (*C*H–N_3, s); 142.0 (*C*H, s); 144.9 (*C*, s); 153.6 (*C*, s); 157.0 (*C*, s); 157.5 (*C*, s); 159.0 (*C*, s); 171.0 (*FA*⁻, s); 179.0 (*C*O, s); 180.4 (*C*O, s). **ESI-HRMS:** 762.2024 $[M]^+$ (theor. $[C_{33}H_{37}N_9O_4La_1]^+$ = 762.2027). **UV absorption:** λ_{max} = 275 nm. **EA** ($[C_{33}H_{37}N_9O_4La_1]^+[FA]^-\cdot 4.8H_2O$, M_R = 894.1): C 45.7 (46.1); H 5.4 (4.7); N 14.1 (13.6); La 15.5 (15.0).

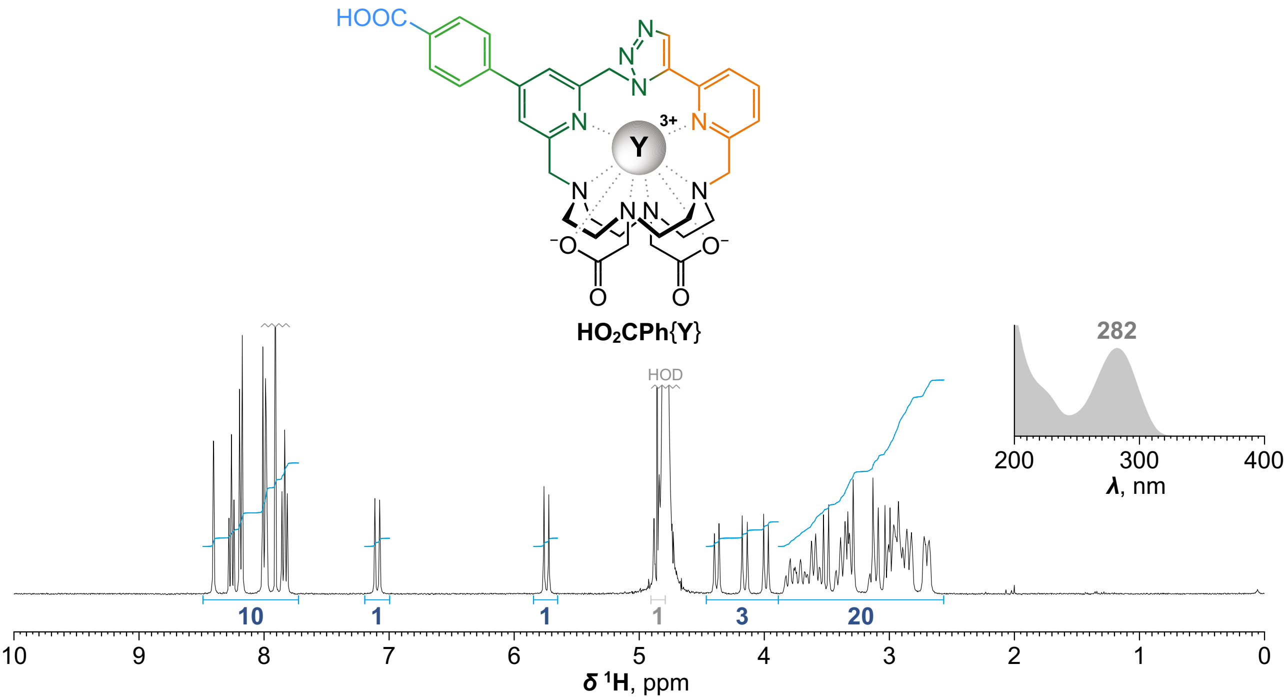

Supplementary Fig. 73. Synthesis and characterization of $HO_2CPh\{Y\}$. In a glass vial (20 mL), **HO_2CPhL^1**$\cdot$0.1FA$\cdot$2.9H_2O (13 mg; 18 µmol; 1.0 equiv.) was dissolved in H_2O (18 mL) followed by addition aq. MOPS/NaOH buffer (500 mM; pH 7.0; 1.20 mL; 600 µmol; 31 equiv.) and aq. YCl_3 (100 mM; 197 µL; 20 µmol; 1.1 equiv.) and the mixture was stirred for 22 h at 80 °C. Mixture was then filtered through syringe microfilter (RC). The filtrate was concentrated on rotary evaporator and then directly purified by preparative HPLC (C18; H_2O–MeCN gradient with TFA additive). Fractions with product were joined and directly lyophilized to give product in the form of trifluoroacetate salt as white fluffy solid. **Yield:** 13 mg (71%; 1step; based on **HO_2CPhL^1**$\cdot$0.1FA$\cdot$2.9H_2O). **NMR (D_2O, pD ~2): ^{1}H** (401.0 MHz) δ_H 2.60–3.88 (CH_2–N, CH_2–CO, m, 16+4H); 3.98 (CH_2–arom., d, 1H, $^2J_{HH}$ = 14.9); 4.16 (CH_2–arom., d, 1H, $^2J_{HH}$ = 16.3); 4.38 (CH_2–arom., d, 1H, $^2J_{HH}$ = 14.9); 4.86 (CH_2–arom., d, 1H, $^2J_{HH}$ = 16.3); 5.94 (CH_2–N_3, d, 1H, $^2J_{HH}$ = 15.5); 7.09 (CH_2–N_3, d, 1H, $^2J_{HH}$ = 15.5); 7.79–7.87 (C*H*, m, 2H); 7.91 (C*H*–N_3, s, 1H); 7.98 (C*H*, d, 1H, $^4J_{HH}$ = 1.8); 8.00 (C*H*, d, 2H, $^3J_{HH}$ = 8.5); 8.02 (C*H*, d, 2H, $^3J_{HH}$ = 8.5); 8.26 (C*H*, t, 1H, $^3J_{HH}$ = 7.9); 8.40 (C*H*, d, 1H, $^4J_{HH}$ = 1.8). **ESI-HRMS:** 756.1925 $[M]^+$ (theor. $[C_{34}H_{37}N_9O_6Y_1]^+$ = 756.1920). **UV absorption:** λ_{max} = 282 nm. **EA** ($[C_{34}H_{37}N_9O_6Y_1]^+[TFA]^-\cdot$0.6TFA$\cdot$4.8$H_2O$, M_R = 1024.5): C 43.6 (43.9); H 4.6 (4.2); N 12.3 (12.0); Y 8.7 (7.9); F 8.9 (8.5).

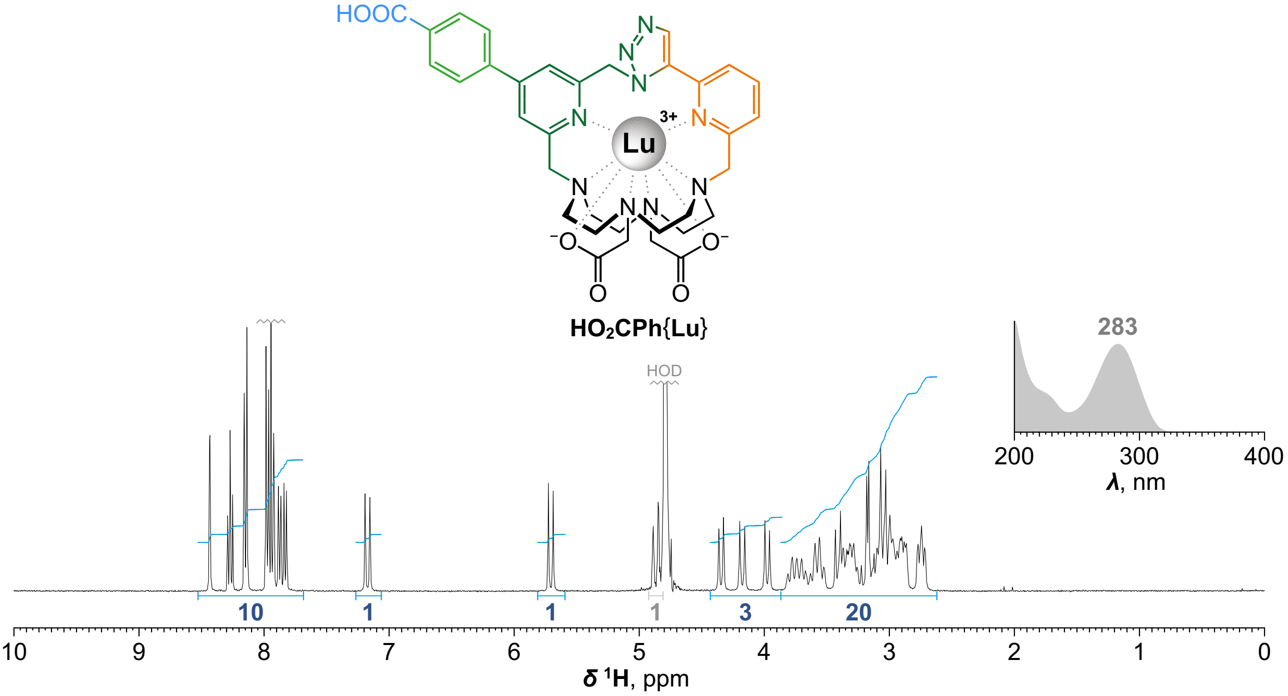

Supplementary Fig. 74. Synthesis and characterization of $HO_2CPh\{Lu\}$. In a glass vial (20 mL), **HO_2CPhL^1**$\cdot 0.2FA\cdot 1.6H_2O$ (85 mg; 120 µmol; 1.0 equiv.) was dissolved in H_2O (12 mL) followed by addition aq. MOPS/NaOH buffer (500 mM; pH 7.0; 6.00 mL; 3.00 mmol; 25 equiv.) and aq. $LuCl_3$ (100 mM; 1.3 mL; 130 µmol; 1.1 equiv.) and the mixture was stirred for 16 h at 80 °C. Mixture was then filtered through syringe microfilter (RC). The filtrate was concentrated on rotary evaporator and then directly purified by preparative HPLC (C18; H_2O–MeCN gradient with TFA additive). Fractions with product were joined and directly lyophilized to give product in the form of trifluoroacetate salt as white fluffy solid. **Yield:** 105 mg (75%; 1step; based on **HO_2CPhL^1**$\cdot 0.2FA\cdot 1.6H_2O$). **NMR (D_2O, pD ~2): ^{1}H** (400.1 MHz) δ_H 2.64–3.86 (CH_2–N, CH_2–CO, m, 16+4H); 3.98 (CH_2–arom., d, 1H, $^2J_{HH}$ = 14.7); 4.18 (CH_2–arom., d, 1H, $^2J_{HH}$ = 15.9); 4.34 (CH_2–arom., d, 1H, $^2J_{HH}$ = 14.7); 4.87 (CH_2–arom., d, 1H, $^2J_{HH}$ = 15.9); 5.71 (CH_2–N_3, d, 1H, $^2J_{HH}$ = 15.3); 7.17 (CH_2–N_3, d, 1H, $^2J_{HH}$ = 15.3); 7.83 (C*H*, dd, 1H, $^3J_{HH}$ = 7.9, $^4J_{HH}$ = 1.1); 7.88 (C*H*, dd, 1H, $^3J_{HH}$ = 7.9, $^4J_{HH}$ = 1.1); 7.92 (C*H*, d, 1H, $^4J_{HH}$ = 1.7); 7.94 (C*H*–N_3, s, 1H); 7.97 (C*H*, d, 2H, $^3J_{HH}$ = 8.5); 8.15 (C*H*, d, 2H, $^3J_{HH}$ = 8.5); 8.27 (C*H*, t, 1H, $^3J_{HH}$ = 7.9); 8.43 (C*H*, d, 1H, $^4J_{HH}$ = 1.7). **ESI-HRMS:** 842.2259 $[M]^+$ (theor. $[C_{34}H_{37}N_9O_6Lu_1]^+$ = 842.2269). **UV absorption:** λ_{max} = 283 nm. **EA** ($[C_{34}H_{40}N_{10}O_4Lu_1]^+[TFA]^-\cdot 1.4TFA\cdot 3.2H_2O$, M_R = 1172.9): C 39.7 (40.0); H 3.9 (3.5); N 10.7 (10.3); Lu 14.9 (14.9); F 11.7 (11.8). **X-Ray**: In a glass vial (4 mL), [**$HO_2CPh\{Lu\}$**]$^+[TFA]^-\cdot 1.4TFA\cdot 3.2H_2O$ (1.8 mg; 1.5 µmol; 1.0 equiv.) was dissolved in H_2O (120 µL) followed by addition of aq. $HClO_4$ (1.0 M; 2.5 µL; 2.5 µmol; 1.6 equiv.). The vial was gently heated and vortexed. The vial was left standing inside of a closed larger glass vial (20 mL) filled with *i*-PrOH, yielding microcrystalline material. Mother liquor was discarded and the solid residue was re-dissolved in H_2O (2 mL) with the help of heat, vortexing and ultrasound. The solution was filtered through syringe microfilter (RC) and left standing inside of a closed larger glass vial (20 mL) filled with *i*-PrOH, yielding single crystals over time.

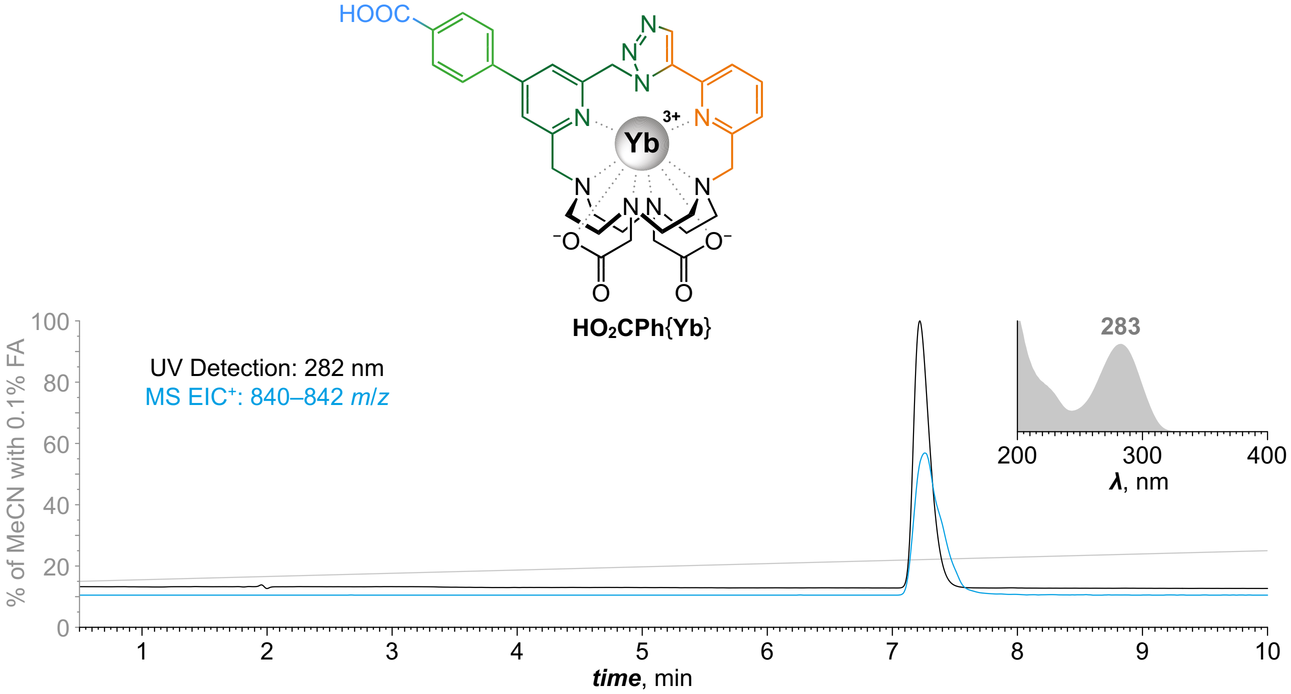

Supplementary Fig. 75. Synthesis and characterization of HO$_2$CPh{Yb}. In a glass vial (20 mL), **HO$_2$CPhL^1**·0.1FA.3.2H$_2$O (59.0 mg; 83 µmol; 1.0 equiv.) was dissolved in H_2O (25 mL) followed by addition aq. MOPS/NaOH buffer (500 mM; pH 7.0; 5.0 mL; 2.50 mmol; 30 equiv.) and aq. $YbCl_3$ (100 mM; 1.00 mL; 100 µmol; 1.2 equiv.) and the mixture was stirred for 16 h at 80 °C. Mixture was then filtered through syringe microfilter (RC). The filtrate was concentrated on rotary evaporator and then directly purified by preparative HPLC (C18; H_2O–MeCN gradient with TFA additive). Fractions with product were joined and directly lyophilized to give product in the form of trifluoroacetate salt as white fluffy solid. **Yield:** 85.2 mg (83%; 1step; based on **HO$_2$CPhL^1**·0.1FA.3.2H$_2$O). **ESI-HRMS:** 841.2256 $[M]^+$ (theor. $[C_{34}H_{37}N_9O_6Yb_1]^+$ = 841.2250). **UV absorption:** λ_{max} = 283 nm. **EA** ($[C_{34}H_{40}N_{10}O_4Yb_1]^+[TFA]^-$·2.1TFA·2.0H$_2$O, M_R = 1229.2): C 39.3 (39.1); H 3.5 (3.1); N 10.3 (9.9); F 14.4 (14.0).

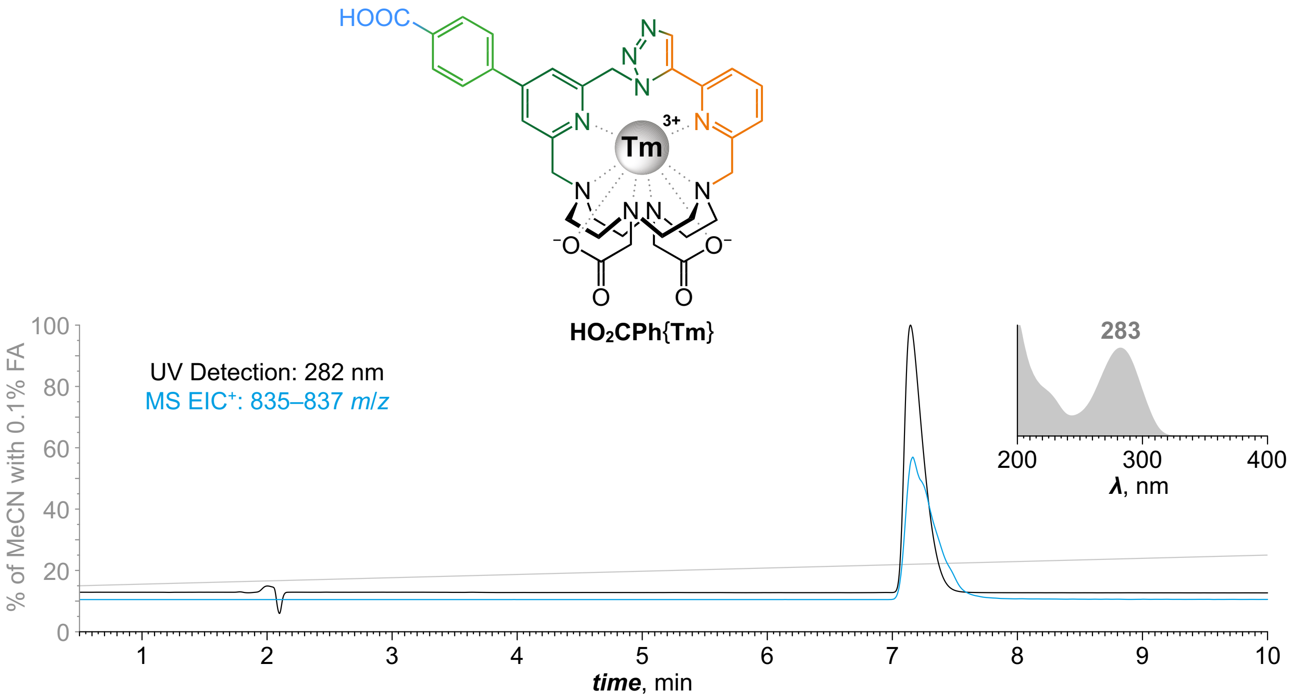

Supplementary Fig. 76. Synthesis and characterization of $HO_2CPh\{Tm\}$. In a glass vial (20 mL), **HO_2CPhL^1**·0.1FA.3.2H_2O (48.6 mg; 66.4 µmol; 1.0 equiv.) was dissolved in H_2O (15 mL) followed by addition aq. MOPS/NaOH buffer (500 mM; pH 7.0; 3.4 mL; 1.70 mmol; 26 equiv.) and aq. $TmCl_3$ (100 mM; 800 µL; 80.0 µmol; 1.2 equiv.) and the mixture was stirred for 8 h at 80 °C. Mixture was then filtered through syringe microfilter (RC). The filtrate was concentrated on rotary evaporator and then directly purified by preparative HPLC (C18; H_2O–MeCN gradient with TFA additive). Fractions with product were joined and directly lyophilized to give product in the form of trifluoroacetate salt as white fluffy solid. **Yield:** 65.2 mg (88%; 1 step; based on **HO_2CPhL^1**·0.1FA.3.2H_2O). **ESI-HRMS:** 836.2206 $[M]^+$ (theor. $[C_{34}H_{37}N_9O_6Tm_1]^+$ = 836.2203). **UV absorption:** λ_{max} = 283 nm. **EA** ($[C_{34}H_{40}N_{10}O_4Tm_1]^+[TFA]^-$·1.1TFA·2.6$H_2O$, M_R = 1121.9): C 40.9 (40.9); H 3.9 (3.8); N 11.2 (11.2); Tm 15.1 (15.2); F 10.7 (10.7).

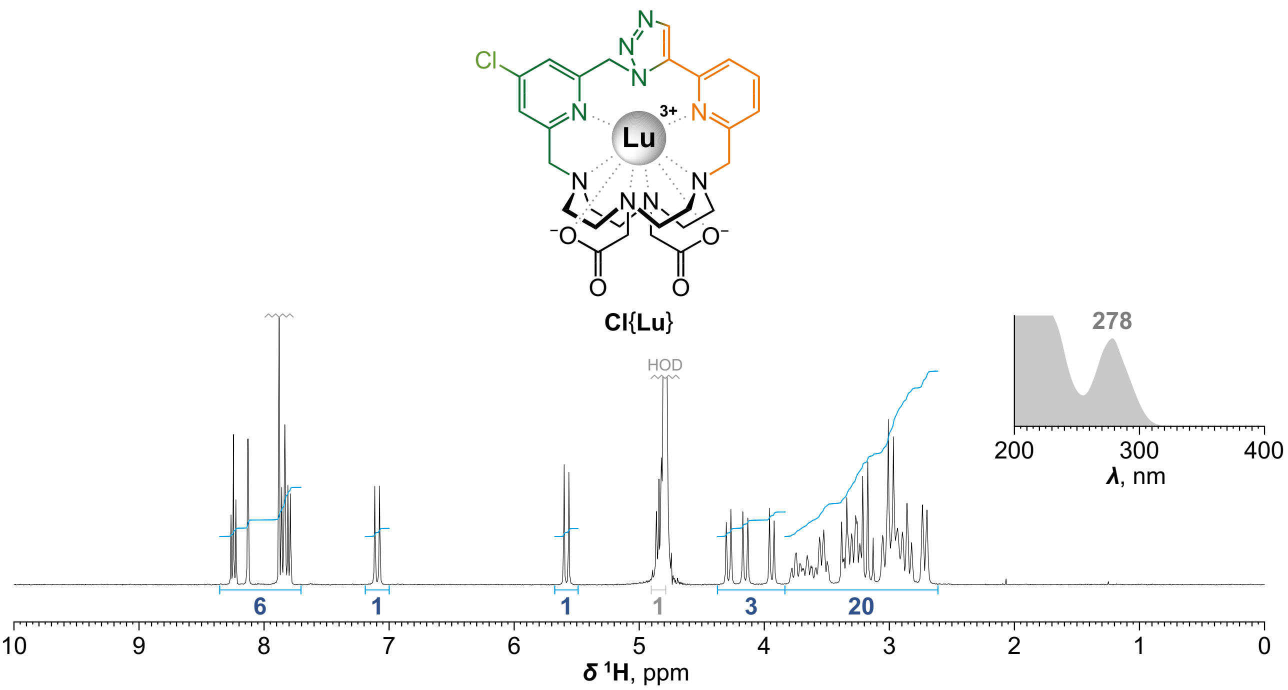

Supplementary Fig. 77. Synthesis and characterization of Cl{Lu}. In a glass vial (40 mL), **ClL**[1]$\cdot$0.7TFA$\cdot$1.7H_2O (190 mg; 274 μmol; 1.0 equiv.) was dissolved in H_2O (21 mL) followed by addition aq. MOPS/NaOH buffer (500 mM; pH 7.0; 15.4 mL; 7.70 mmol; 28 equiv.) and aq. $LuCl_3$ (100 mM; 3.30 mL; 330 μmol; 1.2 equiv.) and the mixture was stirred for 16 h at 80 °C. Mixture was then filtered through syringe microfilter (RC). The filtrate was concentrated on rotary evaporator and then directly purified by preparative HPLC (C18; H_2O–MeCN gradient with TFA additive). Fractions with product were joined and directly lyophilized to give product in the form of trifluoroacetate salt as white fluffy solid. **Yield:** 224 mg (85%; 1 step; based on **ClL**[1]$\cdot$0.7TFA$\cdot$1.7H_2O). **NMR (D_2O, pD ~3):** ^{1}**H** (401.0 MHz) δ_H 2.61–3.84 (CH_2–N, CH_2–CO, m, 16+4H); 3.94 (CH_2–arom., d, 1H, $^{2}J_{HH}$ = 14.9); 4.15 (CH_2–arom., d, 1H, $^{2}J_{HH}$ = 15.9); 4.29 (CH_2–arom., d, 1H, $^{2}J_{HH}$ = 14.9); 4.84 (CH_2–arom., d, 1H, $^{2}J_{HH}$ = 15.9); 5.58 (CH_2–N_3, d, 1H, $^{2}J_{HH}$ = 15.3); 7.10 (CH_2–N_3, d, 1H, $^{2}J_{HH}$ = 15.3); 7.80 (C*H*, dd, 1H, $^{3}J_{HH}$ = 7.9, $^{4}J_{HH}$ = 1.2); 7.83 (C*H*, d, 1H, $^{4}J_{HH}$ = 1.9); 7.85 (C*H*, dd, 1H, $^{3}J_{HH}$ = 7.9, $^{4}J_{HH}$ = 1.2); 7.88 (C*H*–N_3, s, 1H); 8.13 (C*H*, d, 1H, $^{4}J_{HH}$ = 1.9); 8.24 (C*H*, t, 1H, $^{3}J_{HH}$ = 7.9). **ESI-HRMS:** 756.1664 $[M]^+$ (theor. $[C_{27}H_{32}N_9O_4Cl_1Lu_1]^+$ = 756.1668). **UV absorption:** λ_{max} = 278 nm. **EA** ($[C_{27}H_{32}N_9O_4Cl_1Lu_1]^+[TFA]^-\cdot5.1H_2O$, M_R = 961.9): C 36.2 (36.4); H 4.4 (4.0); N 13.1 (12.9); Cl 3.7 (3.7); F 5.9 (6.4); Lu 18.2 (16.8). **X-Ray**: In a glass vial (4 mL), [**Cl**{**Lu**}]$^+$[TFA]$^-\cdot$5.1H_2O (1.6 mg; 1.7 μmol; 1.0 equiv.) was dissolved in H_2O (75 μL) followed by addition of aq. $HClO_4$ (1.0 M; 2.0 μL; 2.0 μmol; 1.2 equiv.). The vial was gently heated and vortexed. The vial was left standing inside of a closed larger glass vial (20 mL) filled with *i*-PrOH, yielding single crystals over time.

large scale reaction

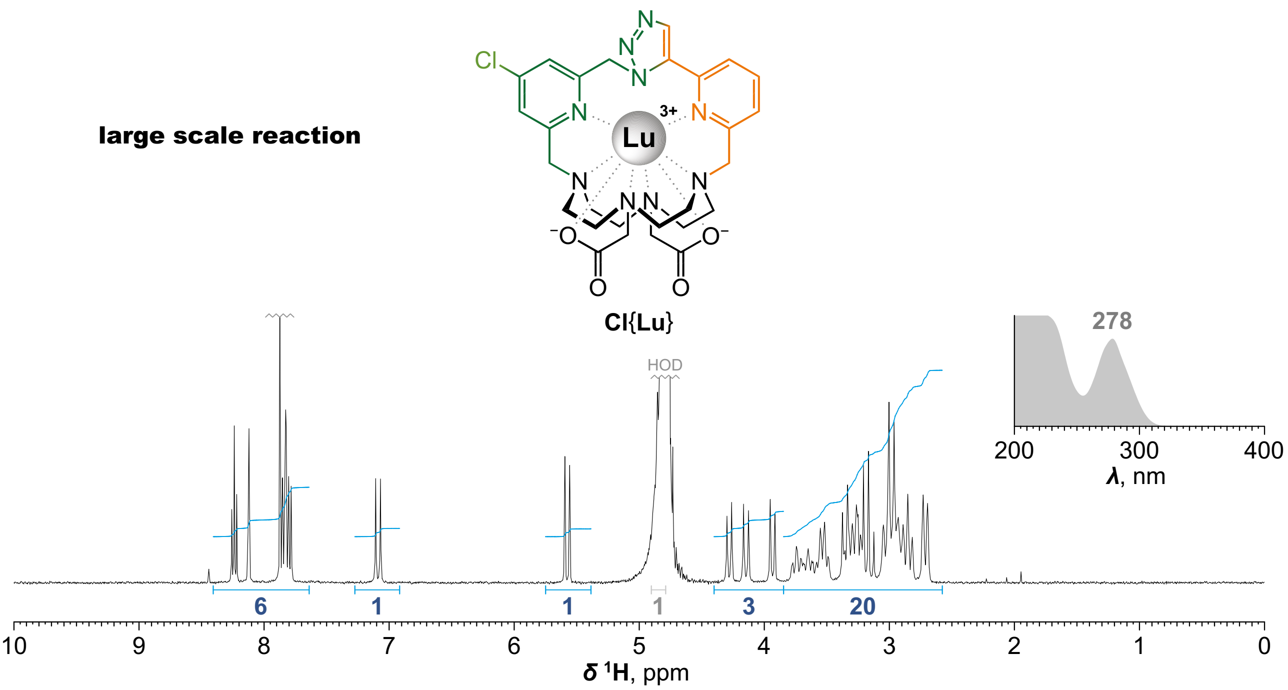

Supplementary Fig. 78. Large scale synthesis of Cl{Lu}. In a round-bottom glass flask (500 mL), **ClL¹**·0.6TFA·2.4H_2O (905 mg; 1.34 mmol; 1.0 equiv.) was dissolved in H_2O (300 mL) followed by addition aq. MOPS/NaOH buffer (500 mM; pH 7.0; 80 mL; 40 mmol; 30 equiv.) and aq. $LuCl_3$ (100 mM; 15 mL; 1.5 mmol; 1.1 equiv.) and the mixture was stirred for 12 h at 80 °C. Mixture was then concentrated on rotary evaporator and then directly purified by flash chromatography (C18; H_2O–MeCN gradient with TFA additive). Fractions with product were joined and directly lyophilized to give product in the form of trifluoroacetate salt as white fluffy solid. **Yield:** 1.17 g (91%; 1 step; based on **ClL¹**·0.6TFA·2.4H_2O). **EA** ($[C_{27}H_{32}N_9O_4Cl_1Lu_1]^+[TFA]^-$·0.3TFA·4.4$H_2O$, M_R = 983.5): C 36.2 (36.3); H 4.2 (4.1); N 12.8 (12.8); Cl 3.6 (3.5); F 7.5 (7.4).

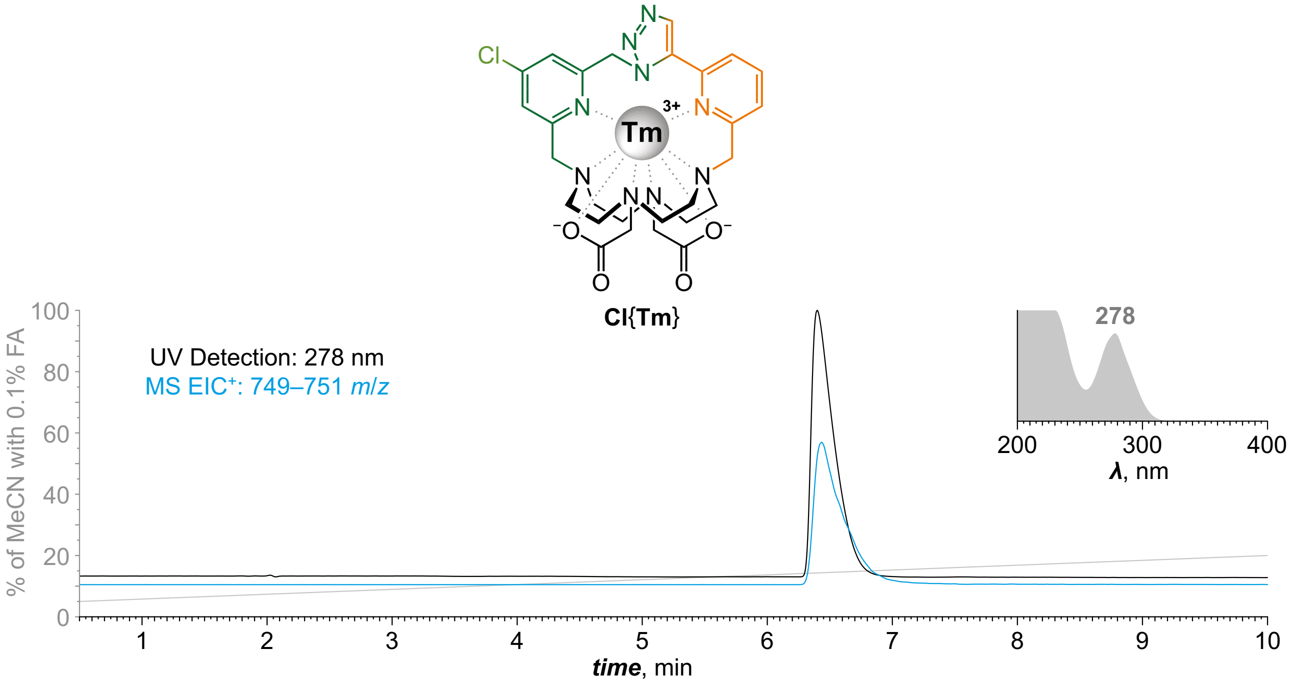

Supplementary Fig. 79. Synthesis and characterization of Cl{Tm}. In a glass vial (20 mL), **ClL¹**·0.6TFA·1.0H_2O (56.5 mg; 84.3 µmol; 1.0 equiv.) was dissolved in H_2O (15 mL) followed by addition aq. MOPS/NaOH buffer (500 mM; pH 7.0; 4.0 mL; 2.00 mmol; 24 equiv.). and aq. $TmCl_3$ (100 mM; 1.00 mL; 100 µmol; 1.2 equiv.) and the mixture was stirred for 6 h at 80 °C. Mixture was then filtered through syringe microfilter (RC). The filtrate was concentrated on rotary evaporator and then directly purified by preparative HPLC (C18; H_2O–MeCN gradient with TFA additive). Fractions with product were joined and directly lyophilized to give product in the form of trifluoroacetate salt as white fluffy solid. **Yield:** 68.0 mg (86%, 1 step; based on **ClL¹**·0.6TFA·1.0H_2O). **ESI-HRMS:** 750.1608 $[M]^+$ (theor. $[C_{27}H_{32}N_9O_4Cl_1Tm_1]^+$ = 750.1602). **UV absorption:** λ_{max} = 278 nm. **EA** ($[C_{27}H_{32}N_9O_4Cl_1Tm_1]^+[TFA]^-$·0.2TFA·2.6$H_2O$, M_R = 933.6): C 37.8 (37.7); H 4.0 (3.8); N 13.5 (13.4); Cl 3.8 (3.9); F 7.3 (7.3); Tm 18.1 (17.8).

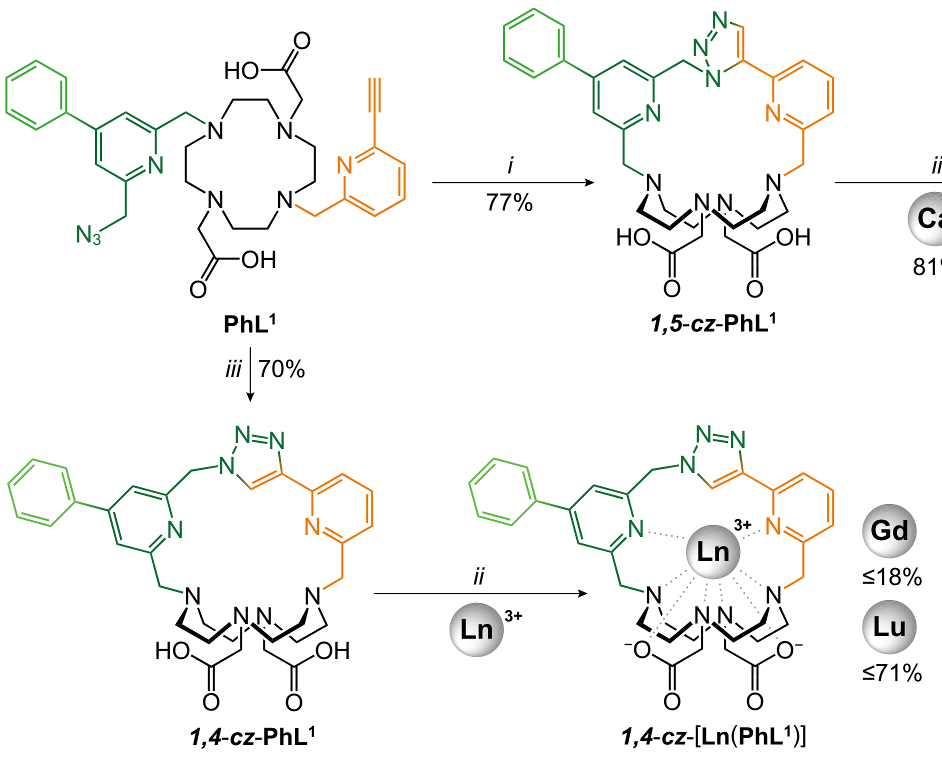

Supplementary Fig. 80. Empty cages and their direct complexation. Both isomers of empty cages can be obtained by simple heating of **PhL¹** in aqueous environment: ***1,5-cz*-PhL¹** is the major product at acidic conditions (or alternatively using Na^I ions template at alkaline conditions), while ***1,4-cz*-PhL¹** isomer is formed predominantely at alkaline pH in the absence of templating cations. The reaction rate is strongly dependent on pH (slowest in the 5–7 pH range, which is typically suitable for metal ion complexation), suggesting variety in distance between the azide and alkyne moiety as a function of intramolecular hydrogen bonding at given pH. The cavity of ***1,5-cz*-PhL¹** is tight enought to completely prevent direct complexation of Ln^{III} ions (0% conversion even after 6 months at 80 °C), therefore leaving ClickZip as the only option to access **Ph{Lu}**. On contrary, direct complexation of Ca^{II} ions is viable. While complexation of Ln^{3+} ions with slightly larger ***1,4-cz*-PhL¹** is possible, the reaction is very inefficient (requires prolonged heating and does not proceeds quantitatively even with large excess of Ln^{III} ions). Empty cages (example with ***1,5-cz*-ClL¹**) can also be obtained as minor by-products during synthesis of corresponding ligands. ***Conditions:*** (*i*) aq. citric acid (pH 2.4), 80 °C; (*ii*) MOPS/NaOH buffer (pH 7.0), 80 °C; (*iii*) borate/Cs_2CO_3 buffer (pH 9.0), 80 °C. Yields refer to isolated compounds.

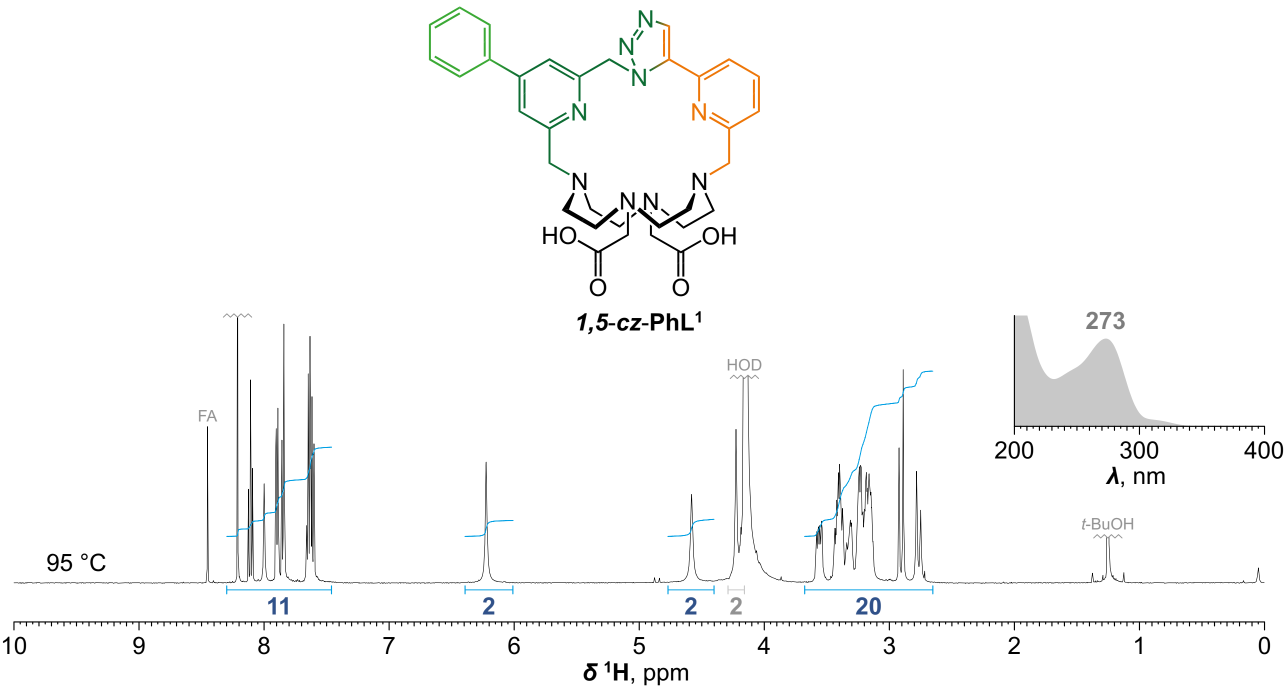

Supplementary Fig. 81. Synthesis and characterization of *1,5-cz*-PhL^1. In a glass vial (40 mL), **PhL^1**·0.2FA·0.9H_2O (130 mg; 200 µmol; 1.0 equiv.) was dissolved in H_2O (20 mL) followed by addition aq. citric acid (100 mM; 20 mL; 2.0 mmol; 10 equiv.) to reach pH 2.4. Resulting mixture was then stirred for 2 d at 80 °C. Mixture was then filtered through syringe microfilter (RC). The filtrate was concentrated on rotary evaporator and then directly purified by preparative HPLC (C18; H_2O–MeCN gradient with FA additive). Fractions with product were joined and directly lyophilized to give product in the form of formate salt as white fluffy solid. **Yield:** 109 mg (77%; 1 step; based on **PhL^1**·0.2FA·0.9H_2O). **NMR (D_2O, pD ~5, 95 °C): ^{1}H** (500.0 MHz) δ_H 2.69–3.63 (CH_2–N, CH_2–CO, m, 16+4H); 4.22 (CH_2–arom., s, 2H); 4.58 (CH_2–arom., s, 2H); 6.22 (CH_2–N_3, s, 2H); 7.61 (C*H*, dd, 1H, $^3J_{HH}$ = 7.9, $^4J_{HH}$ = 1.0); 7.60–7.67 (C*H*, m, 3H); 7.83 (C*H*, s, 1H); 7.85 (C*H*, dd, 1H, $^3J_{HH}$ = 7.9, $^4J_{HH}$ = 1.0); 7.86–7.92 (C*H*, m, 2H); 8.00 (C*H*, s, 1H); 8.11 (C*H*, t, 1H, $^3J_{HH}$ = 7.9); 8.21 (C*H*–N_3, s, 1H). **^{13}C{^{1}H}** (125.7 MHz) δ_C 48.5 (*C*H_2–N, s); 49.9 (*C*H_2–N, s); 50.2 (*C*H_2–N, s); 50.4 (*C*H_2–N, s); 53.4 (*C*H_2–N_3, s); 55.6 (*C*H_2–CO, s); 57.8 (*C*H_2–arom., s); 59.2 (*C*H_2–arom., s); 122.6 (*C*H, s); 124.2 (*C*H, s); 125.4 (*C*H, s); 125.9 (*C*H, s); 127.8 (*C*H, s); 129.9 (*C*H, s); 130.7 (*C*H, s); 134.5 (*C*H–N_3, s); 137.0 (*C*, s); 138.0 (*C*, s); 139.9 (*C*, s); 147.1 (*C*H, s); 152.3 (*C*, bs); 153.6 (*C*, bs); 153.8 (*C*, bs); 156.8 (*C*, s); 172.6 (*C*O, s). **ESI-HRMS:** 626.3200 [M+H]$^+$ (theor. $[C_{33}H_{40}N_9O_4]^+$ = 626.3198). **UV absorption:** λ_{max} = 273 nm. **EA** ($C_{33}H_{39}N_9O_4$·0.9FA·2.0H_2O, M_R = 703.2): C 57.9 (58.3); H 6.4 (5.9); N 17.9 (17.5). **X-Ray**: In a glass vial (2 mL), ***1,5-cz*-PhL^1**·1.1FA·1.6H_2O (2.0 mg; 2.8 µmol; 1.0 equiv.) was dissolved in H_2O (300 µL) followed by addition of aq. $HClO_4$ (1.0 M; 6.0 µL; 6.0 µmol; 2.1 equiv.). Immediately formed precipitate was dissolved by gentle heating and vortexing of the vial. Clear solution was left standing, yielding single crystals over time.

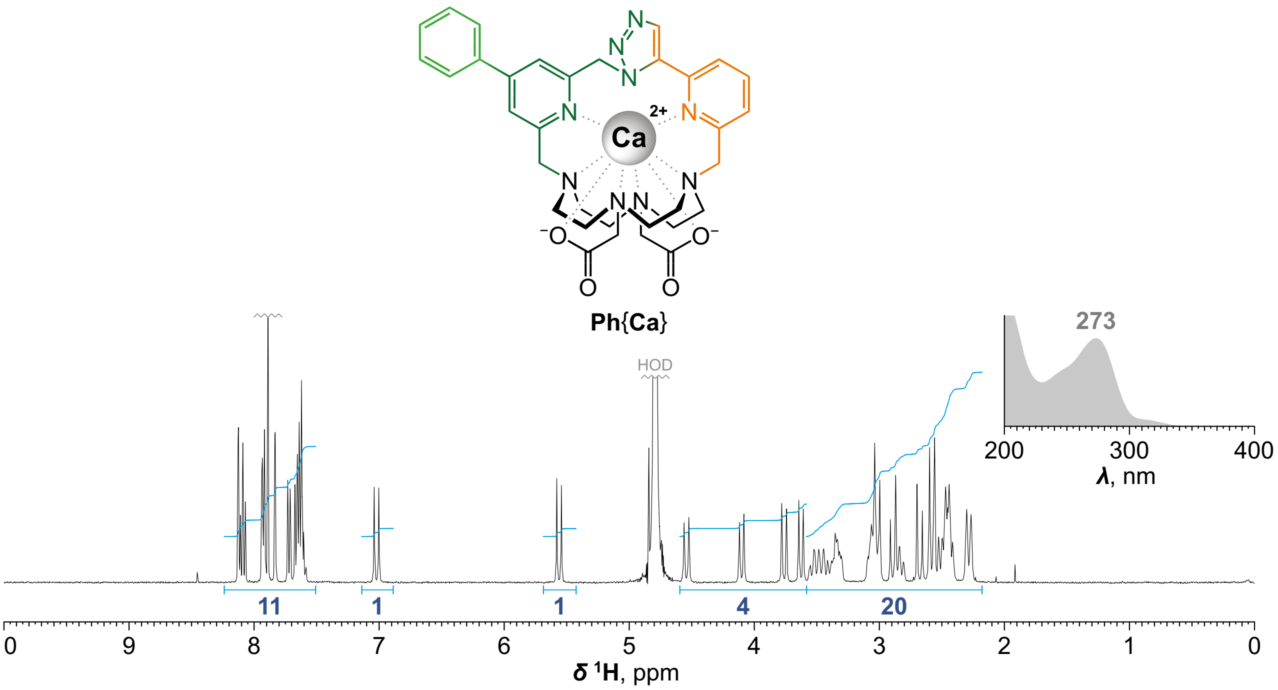

Supplementary Fig. 82. Synthesis and characterization of Ph{Ca}. In a glass vial (20 mL), ***1,5-cz*-PhL**[1]·1.1FA·1.6H_2O (8.2 mg; 11.7 µmol; 1.0 equiv.) was dissolved in H_2O (2 mL) followed by addition aq. MOPS/NaOH buffer (500 mM; pH 7.0; 1.35 mL; 675 µmol; 58 equiv.) and aq. $Ca(NO_3)_2$ (100 mM; 200 µL; 20 µmol; 1.7 equiv.) and the mixture was stirred for 24 h at 80 °C. Mixture was then directly purified by preparative HPLC (C18; H_2O–MeCN gradient with no additive). Fractions with product were joined and directly lyophilized to give product as white fluffy solid. **Yield:** 7.4 mg (81%; 1 step; based on ***1,5-cz*-PhL**[1]·1.1FA·1.6H_2O). **NMR (D_2O, pD ~6):** ^{1}**H** (401.0 MHz) δ_H 2.20–3.58 (C*H*$_2$–N, C*H*$_2$–CO, m, 16+4H); 3.62 (C*H*$_2$–arom., d, 1H, $^2J_{HH}$ = 14.2); 3.76 (C*H*$_2$–arom., d, 1H, $^2J_{HH}$ = 15.3); 4.09 (C*H*$_2$–arom., d, 1H, $^2J_{HH}$ = 14.2); 4.54 (C*H*$_2$–arom., d, 1H, $^2J_{HH}$ = 15.3); 5.56 (C*H*$_2$–N$_3$, d, 1H, $^2J_{HH}$ = 14.9); 7.02 (C*H*$_2$–N$_3$, d, 1H, $^2J_{HH}$ = 14.9); 7.57–7.65 (C*H*, m, 3H); 7.66 (C*H*, d, 1H, $^3J_{HH}$ = 7.9); 7.72 (C*H*, d, 1H, $^3J_{HH}$ = 7.9); 7.83 (C*H*, d, 1H, $^4J_{HH}$ = 1.6); 7.89 (C*H*–N$_3$, s, 1H); 7.90–7.95 (C*H*, m, 2H); 8.09 (C*H*, t, 1H, $^3J_{HH}$ = 7.9); 8.12 (C*H*, d, 1H, $^4J_{HH}$ = 1.6). ^{13}**C{**^{1}**H}** (100.8 MHz) δ_C 48.4 (*C*H$_2$–N, s); 48.5 (*C*H$_2$–N, s); 50.6 (*C*H$_2$–N, s); 50.7 (*C*H$_2$–N, s); 52.6 (*C*H$_2$–N, s); 52.7 (*C*H$_2$–N, s); 53.0 (*C*H$_2$–N, s); 53.3 (*C*H$_2$–N$_3$, s); 53.6 (*C*H$_2$–N, s); 57.9 (*C*H$_2$–CO, s); 59.1 (*C*H$_2$–arom., s); 59.2 (*C*H$_2$–arom., s); 59.3 (*C*H$_2$–CO, s); 123.3 (*C*H, s); 124.7 (*C*H, s); 127.0 (*C*H, s); 128.1 (*C*H, s); 128.2 (*C*H, s); 130.1 (*C*H, s); 130.9 (*C*H, s); 136.7 (*C*H–N$_3$, s); 137.3 (*C*, s); 139.4 (*C*, s); 140.9 (*C*H, s); 146.7 (*C*, s); 153.1 (*C*, s); 155.5 (*C*, s); 159.3 (*C*, s); 159.5 (*C*, s); 178.4 (*C*O, s); 179.7 (*C*O, s). **ESI-HRMS:** 664.2663 $[M+H]^+$ (theor. $[C_{33}H_{38}N_9O_4Ca_1]^+$ = 664.2667). **UV absorption:** λ_{max} = 273 nm. **EA** ($[C_{33}H_{37}N_9O_4Ca_1]$·6.7H_2O, M_R = 784.5): C 50.5 (50.5); H 6.5 (6.0); N 16.1 (15.6); Ca 5.1 (4.8). **X-Ray:** In a glass vial (2 mL), **Ph{Ca}**·6.7H_2O (1.5 mg; ~2 µmol) was mixed with H_2O (50 µL). The mixture was briefly gently heated and vortexed to produce clear colorless solution. The vial was left standing inside of empty closed larger glass vial (20 mL), yielding single crystals over time.

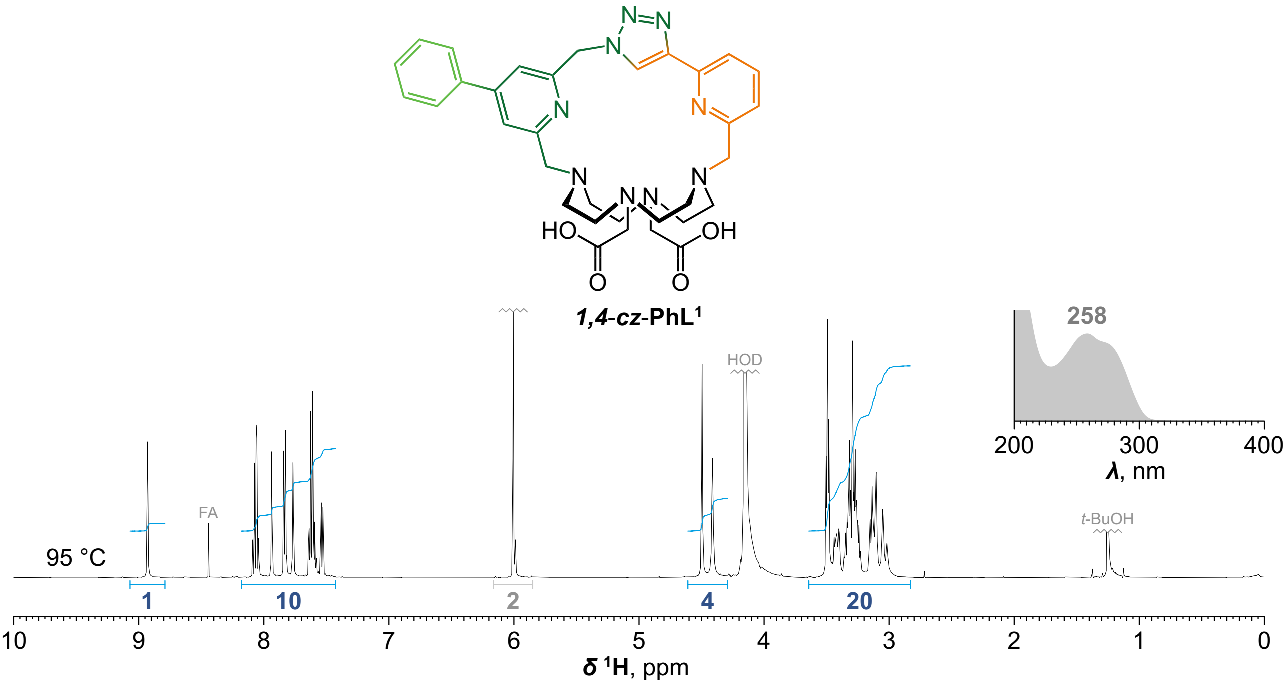

Supplementary Fig. 83. Synthesis and characterization of *1,4-cz*-PhL^1. In a glass vial (40 mL), **PhL^1**·1.3TFA (78.0 mg; 103 µmol; 1.0 equiv.) was dissolved in H_2O (34 mL) followed by addition aq. borate/Cs_2CO_3 buffer (200 mM; pH 9.0; 6.0 mL; 1.2 mmol; ~12 equiv.). Resulting mixture was then stirred for 6 d at 80 °C. Mixture was then filtered through syringe microfilter (RC). The filtrate was concentrated on rotary evaporator and then directly purified by preparative HPLC (C18; H_2O–MeCN gradient with FA additive). Fractions with product were joined and directly lyophilized to give product in the form of formate salt as white fluffy solid. **Yield:** 49.3 mg (70%; 1 step; based on **PhL^1**·1.3TFA). **NMR (D_2O, pD ~5, 95 °C): ^{1}H** (500.0 MHz) δ_H 2.93–3.56 (CH_2–N, CH_2–CO, m, 16+4H); 4.41 (CH_2–arom., s, 2H); 4.49 (CH_2–arom., s, 2H); 6.00 (CH_2–N_3, s, 2H, partially deuterated at 95 °C); 7.54 (C*H*, dd, 1H, $^3J_{HH}$ = 7.0, $^4J_{HH}$ = 1.7); 7.56–7.66 (C*H*, m, 3H); 7.77 (C*H*, d, 1H, $^4J_{HH}$ = 1.5); 7.79–7.87 (C*H*, m, 2H); 7.94 (C*H*, d, 1H, $^4J_{HH}$ = 1.5); 8.02–8.10 (C*H*, m, 2H); 8.93 (C*H*–N_3, s, 1H). **^{13}C{^{1}H}** (125.7 MHz) δ_C 50.0 (*C*H_2–N, s); 50.7 (*C*H_2–N, bs); 52.0 (*C*H_2–N, s); 52.3 (*C*H_2–N, s); 54.8 (*C*H_2–N_3, s); 56.6 (*C*H_2–CO, s); 59.9 (*C*H_2–arom., s); 60.2 (*C*H_2–arom., bs); 121.2 (*C*H, s); 121.7 (*C*H, s); 123.3 (*C*H, s); 124.5 (*C*H, s); 127.0 (*C*H–N_3, s); 127.7 (*C*H, s); 129.9 (*C*H, s); 130.5 (*C*H, s); 137.1 (*C*, s); 140.2 (*C*H, s); 148.0 (*C*, s); 150.3 (*C*, s); 151.7 (*C*, s); 152.5 (*C*, bs); 154.9 (*C*, s); 155.0 (*C*, s); 174.4 (*C*O, s). **ESI-HRMS:** 626.3199 [M+H]$^+$ (theor. $[C_{33}H_{40}N_9O_4]^+$ = 626.3198). **UV absorption:** λ_{max} = 258 nm. **EA** ($C_{33}H_{39}N_9O_4$·0.6FA·2.3H_2O, M_R = 696.8): C 58.1 (57.9); H 6.5 (6.2); N 18.1 (18.2). **X-Ray**: In a glass vial (2 mL), ***1,4-cz*-PhL^1**·0.6FA·2.3H_2O (1.5 mg; 2.2 µmol; 1.0 equiv.) was dissolved in H_2O (500 µL) followed by addition of aq. $HClO_4$ (1.0 M; 4.5 µL; 4.5 µmol; 2.1 equiv.). Immediately formed precipitate was dissolved by gentle heating and vortexing of the vial. Clear solution was left standing, yielding single crystals over time.

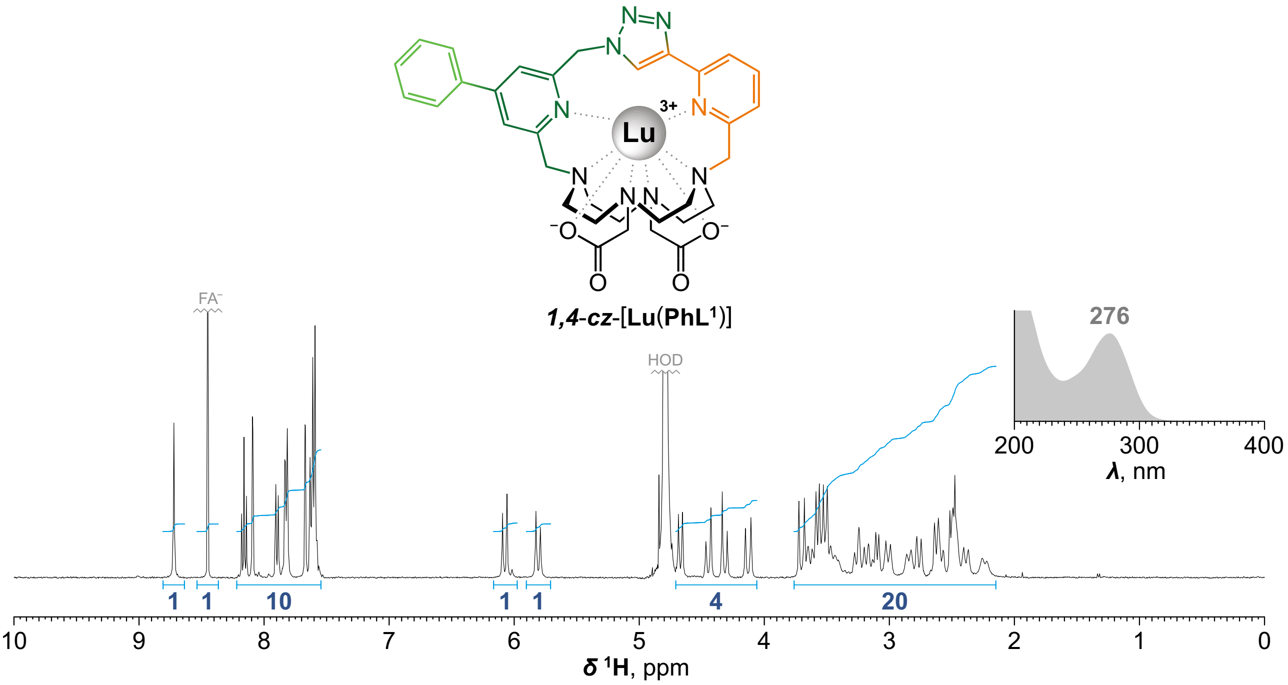

Supplementary Fig. 84. Synthesis and characterization of *1,4-cz*-[Lu(PhL[1])]. In a glass vial (20 mL), ***1,4-cz*-PhL[1]**·0.6FA·2.3H_2O (10.0 mg; 14.4 μmol; 1.0 equiv.) was dissolved in H_2O (2 mL) followed by addition of aq. MOPS/NaOH buffer (500 mM; pH 7.0; 750 μL; 375 μmol; 26 equiv.) and aq. $LuCl_3$ (100 mM; 180 μL; 18.0 μmol; 1.3 equiv.). The resulting suspension was stirred for 20 d at 80 °C. Then, the mixture was diluted with additional H_2O (16 mL) followed by another portion of aq. MOPS/NaOH buffer (500 mM; pH 7.0; 750 μL; 375 μmol; 26 equiv.) and aq. $LuCl_3$ (100 mM; 180 μL; 18.0 μmol; 1.3 equiv.). The resulting suspension was further stirred for 10 d at 80 °C. Mixture was the filtered through syringe microfilter (RC). The filtrate was evaporated to dryness and then resuspended in H_2O (3.6 mL). Mixture was once more filtered through syringe microfilter (RC) and the filtrate was purified by preparative HPLC (C18; H_2O–MeCN gradient with FA additive). Fractions with product were joined and directly lyophilized to give product as white fluffy solid. **Yield:** 8.6 mg (≤71% assuming [**M**]$^+$[FA]$^-$·$x$$H_2O$; M_R = 843.2; 1 step; based on ***1,4-cz*-PhL[1]**·0.6FA·2.3H_2O). **NMR (D_2O, pD ~6): ^{1}H** (401.0 MHz) δ_H 2.13–3.76 (CH_2–N, CH_2–CO, CH_2–arom., m, 16+3+1H); 4.13 (CH_2–CO, d, 1H, $^2J_{HH}$ = 17.7); 4.32 (CH_2–arom., d, 1H, $^2J_{HH}$ = 15.9); 4.45 (CH_2–arom., d, 1H, $^2J_{HH}$ = 15.9); 4.67 (CH_2–arom., d, 1H, $^2J_{HH}$ = 12.7); 5.81 (CH_2– N_3, d, 1H, $^2J_{HH}$ = 14.3); 6.08 (CH_2–N_3, d, 1H, $^2J_{HH}$ = 14.3); 7.56–7.64 (CH, m, 4H); 7.67 (CH, d, 1H, $^4J_{HH}$ = 1.5); 7.79–7.85 (CH, m, 2H); 7.90 (CH, dd, 1H, $^3J_{HH}$ = 7.8, $^4J_{HH}$ = 1.2); 8.09 (CH, d, 1H, $^4J_{HH}$ = 1.5); 8.16 (CH, t, 1H, $^3J_{HH}$ = 7.8); 8.45 (*FA*$^-$, s, 1H); 8.72 (CH–N_3, s, 1H). **ESI-HRMS:** 798.2376 [M]$^+$ (theor. [$C_{33}H_{37}N_9O_4Lu_1$]$^+$ = 798.2371). **UV absorption:** λ_{max} = 276 nm. **X-Ray:** In a glass vial (4 mL), ***1,4-cz*-[Lu(PhL[1])]**$^+$[FA]$^-$·$x$$H_2O$ (1.6 mg; ≤1.9 μmol; 1.0 equiv.) was dissolved in H_2O (250 μL) followed by addition of aq. $HClO_4$ (1.0 M; 2.0 μL; 2.0 μmol; ≥1.0 equiv.). The vial was vortexed and the resulting mixture was filtered through syringe microfilter (RC) to another glass vial (4 mL). The vial was left standing inside of a closed larger glass vial (20 mL) filled with *i*-PrOH, yielding single crystals over time.

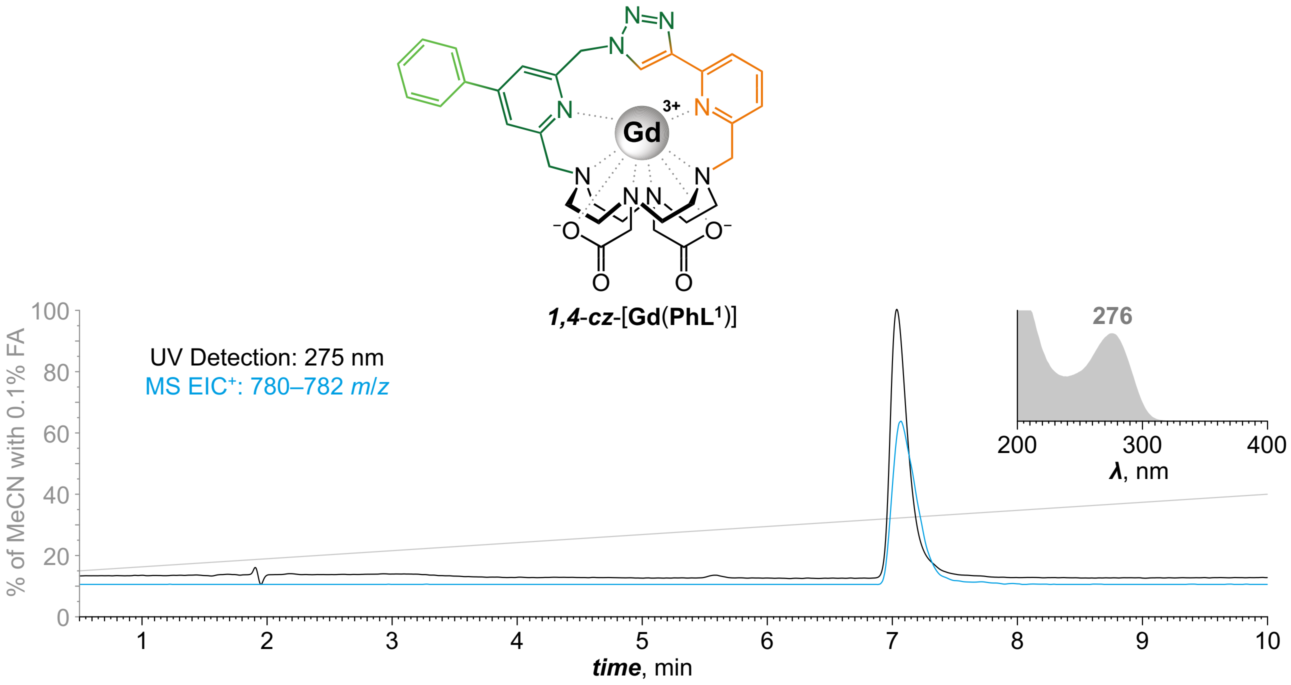

Supplementary Fig. 85. Synthesis and characterization of *1,4-cz*-[Gd(PhL¹)]. In a glass vial (20 mL), ***1,4-cz*-PhL¹**·0.6FA·2.3H_2O (10.0 mg; 14.4 µmol; 1.0 equiv.) was dissolved in H_2O (2 mL) followed by addition of aq. MOPS/NaOH buffer (500 mM; pH 7.0; 750 µL; 375 µmol; 26 equiv.) and aq. $GdCl_3$ (100 mM; 180 µL; 18.0 µmol; 1.3 equiv.). The resulting suspension was stirred for 20 d at 80 °C. Then, the mixture was diluted with additional H_2O (16 mL) followed by another portion of aq. MOPS/NaOH buffer (500 mM; pH 7.0; 750 µL; 375 µmol; 26 equiv.) and aq. $GdCl_3$ (100 mM; 180 µL; 18.0 µmol; 1.3 equiv.). The resulting suspension was further stirred for 10 d at 80 °C. Mixture was the filtered through syringe microfilter (RC). The filtrate was evaporated to dryness and then resuspended in H_2O (3.6 mL). Mixture was once more filtered through syringe microfilter (RC) and the filtrate was purified by preparative HPLC (C18; H_2O–MeCN gradient with FA additive). Fractions with product were joined and directly lyophilized to give product as white fluffy solid. **Yield:** 2.1 mg (≤18% assuming $[\mathbf{M}]^+[\mathrm{FA}]^-\cdot x\mathrm{H_2O}$; M_R = 826.0; 1 step; based on ***1,4-cz*-PhL¹**·0.6FA·2.3H_2O). **ESI-HRMS:** 781.2205 $[M]^+$ (theor. $[C_{33}H_{37}N_9O_4Gd_1]^+$ = 781.2204). **UV absorption:** λ_{max} = 276 nm.

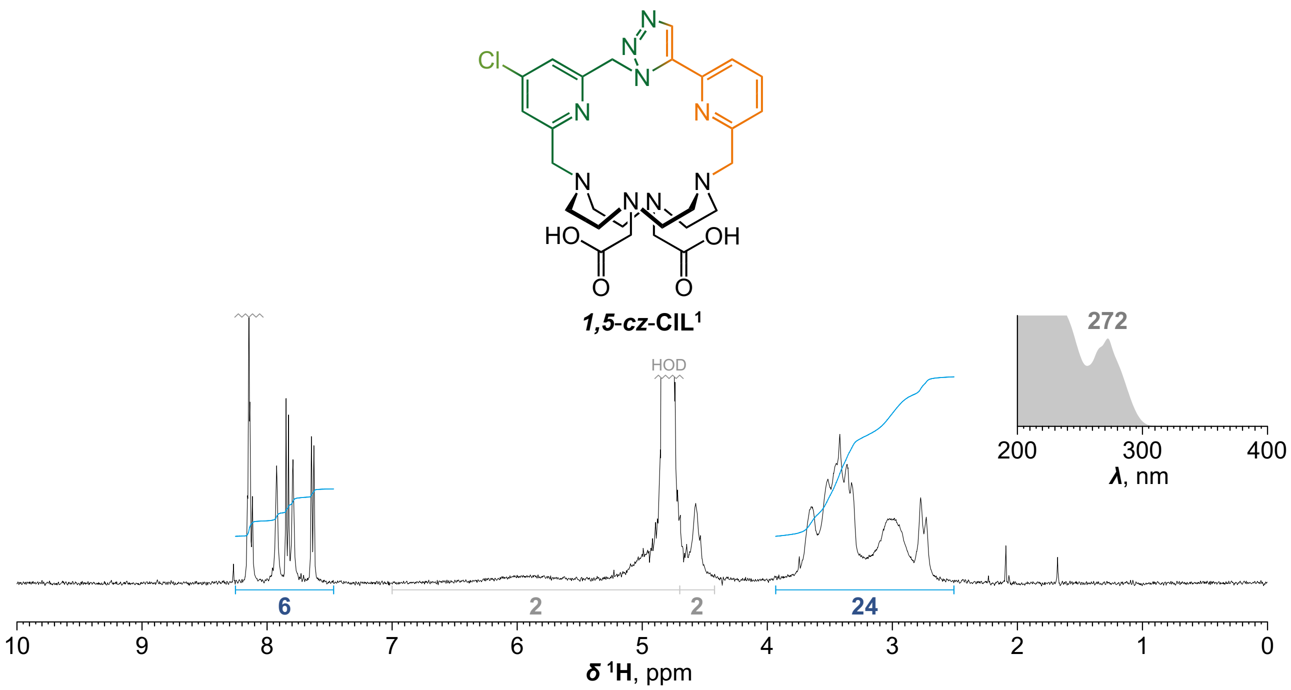

Supplementary Fig. 86. Characterization of *1,5-cz*-ClL[1]. Obtained during large scale synthesis of **ClL[1]** in the form of mixed trifluoroacetate/formate salt as faint yellow solid. **Yield:** 66.6 mg (3%; 1 step; based on **15**). **NMR (D_2O, pD ~3):** ^{1}**H** (401.0 MHz) δ_H 2.48–3.94 (CH_2–N, CH_2–CO, bm, 16+4H); 4.57 (CH_2–arom., bs, 2H); 5.00 (CH_2–arom., bm, 2H); ~4.7–7.0 (CH_2–N_3, bm, 2H); 7.64 (C*H*, dd, 1H, $^3J_{HH}$ = 7.9, $^4J_{HH}$ = 1.0); 7.79 (C*H*, bs, 1H); 7.84 (C*H*, dd, 1H, $^3J_{HH}$ = 7.9, $^4J_{HH}$ = 1.0); 7.92 (C*H*, bs, 1H); 8.14 (C*H*, t, 1H, $^3J_{HH}$ = 7.9); 8.15 (C*H*–N_3, s, 1H). **ESI-HRMS:** 582.2344 $[M-H]^-$ (theor. $[C_{27}H_{33}N_9O_4Cl_1]^-$ = 582.2350). **UV absorption:** λ_{max} = 272 nm. **EA** ($C_{27}H_{34}N_9O_4Cl_1\cdot1.8TFA\cdot3.3H_2O$, M_R = 848.7): C 43.3 (43.3); H 5.0 (4.5); N 14.9 (14.5); Cl 4.2 (4.2); F 12.1 (12.0).

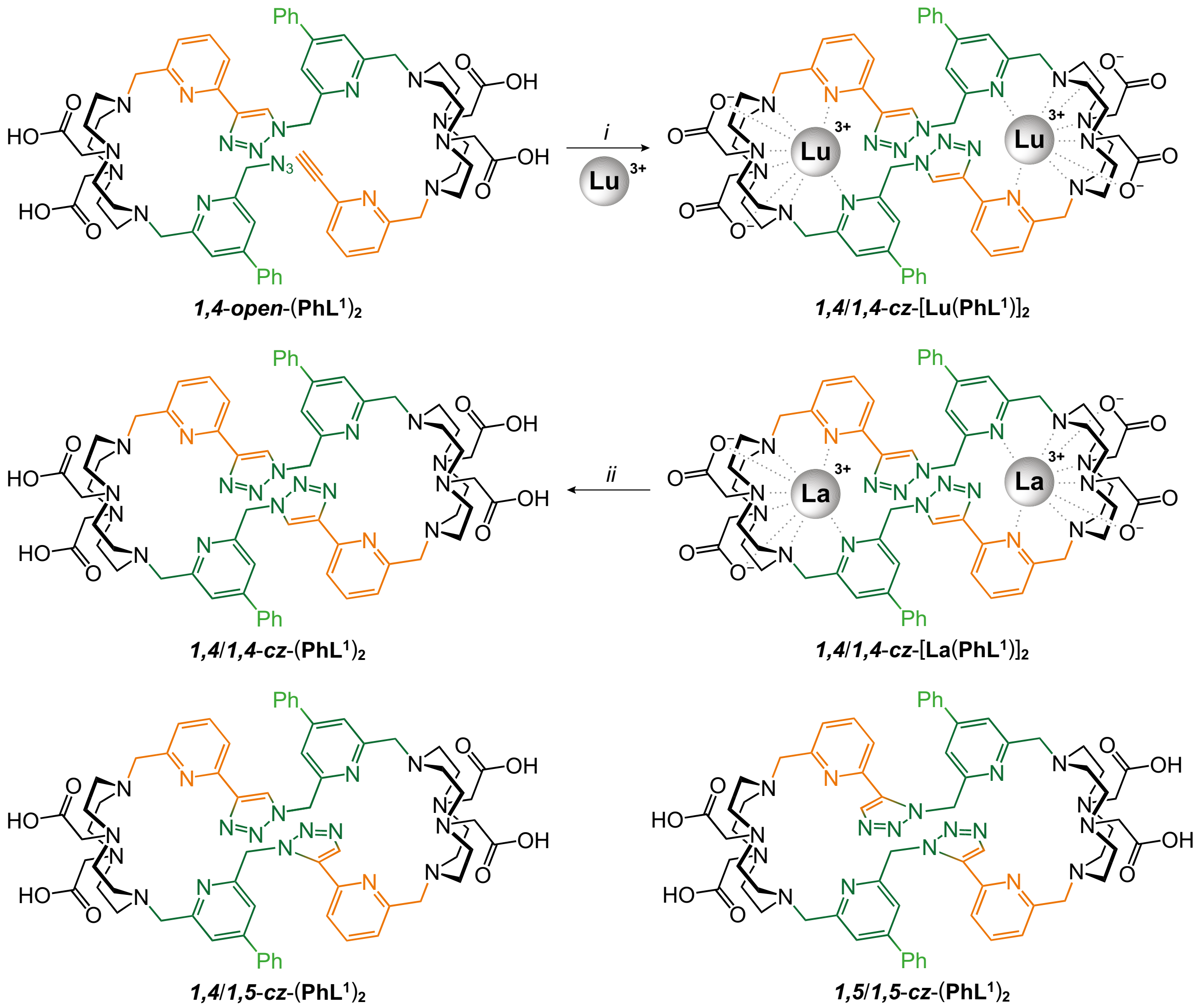

Supplementary Fig. 87. ClickZip dimer byproducts. The minor impurity (typically ≤5%) of isolated **PhL**[1] was identified as ***1,4-open***-(**PhL**[1])$_2$. Its formation was observed during lyophilization of each batch of **PhL**[1] (regardless of purity after preparative HPLC). Upon reaction with with Lu^{3+} ions (pH 7, 80 °C), it cleanly gives symmetrical ***1,4/1,4-cz***-[**Lu**(**PhL**[1])]$_2$ dimer. The analogous ***1,4/1,4-cz***-[**La**(**PhL**[1])]$_2$ was isolated as significant byproduct (20−30%) during synthesis of ***1,4-cz***-[**La**(**PhL**[1])]. Its mild acid hydrolysis (0.1 M HCl, RT) leads to ***1,4/1,4-cz***-(**PhL**[1])$_2$ (same in the case of ***1,4/1,4-cz***-[**Lu**(**PhL**[1])]$_2$). The other empty dimers (symmetrical ***1,5/1,5-cz***-(**PhL**[1])$_2$ and mixed ***1,4/1,5-cz***-(**PhL**[1])$_2$) were isolated as minor byproducts during synthesis of ***1,4-cz***-(**PhL**[1]) at alkaline pH (***1,4/1,4-cz***-(**PhL**[1])$_2$ was also formed, albeit in very low quantitites and its isolation from such reaction mixture was not successful). Since both ***1,4/1,4-cz***-[**La**(**PhL**[1])]$_2$ and ***1,4/1,4-cz***-[**Lu**(**PhL**[1])]$_2$ were found to fully dechelate already in diluted acid, the dimers were not (despite their intriguing structure) further extensively studied. ***Conditions:*** (*i*) MOPS/NaOH buffer, H_2O, 80 °C; (*ii*) HCl, H_2O, RT.

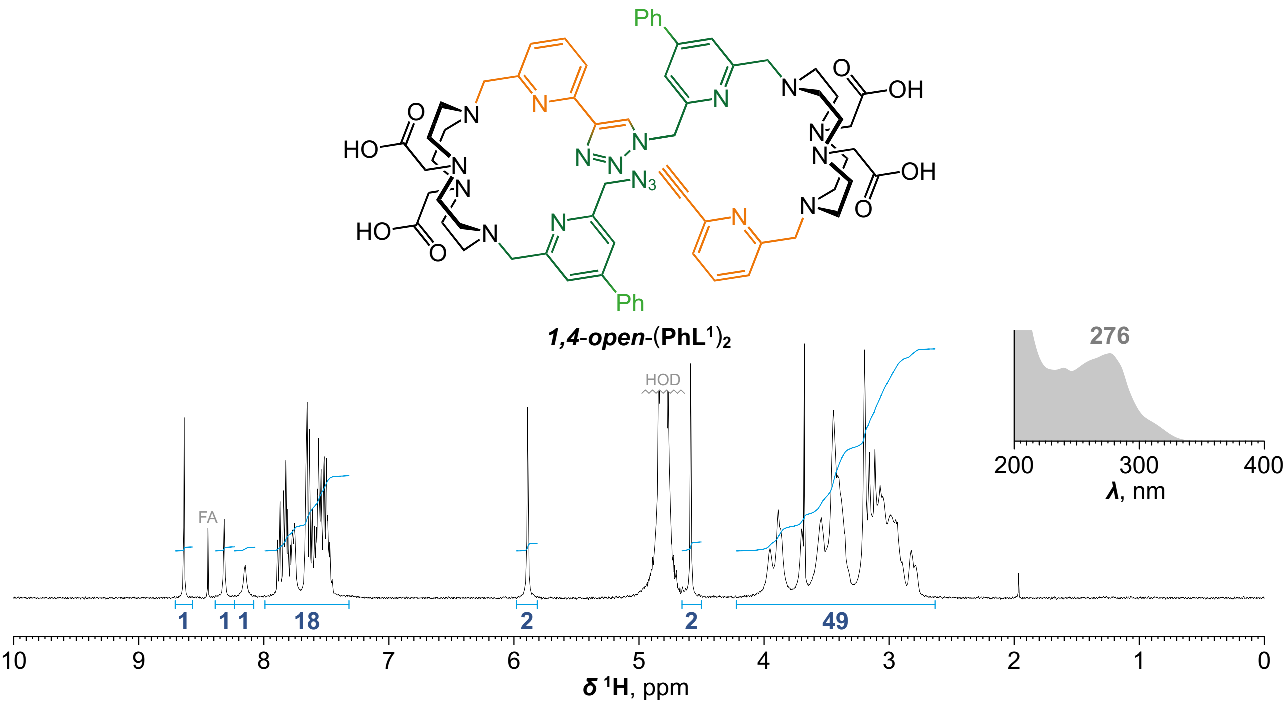

Supplementary Fig. 88. Characterization of byproduct *1,4-open*-(PhL^1)$_2$. Obtained during synthesis of **PhL^1** in the form of formate salt as white solid. **Yield:** 8 mg. **NMR (D_2O): ^{1}H** (401.0 MHz) δ_H 2.61–3.65 (CH_2–N, CH_2–CO, m, 32+8H); 3.68 (C≡C*H*, s, 1H); 3.70 (CH_2–arom., bs, 2H); 3.75–4.21 (CH_2–arom., m, 6H); 4.59 (CH_2–N_3., s, 2H); 5.89 (CH_2–N_3, s, 2H); 7.32–7.98 (C*H*, m, 18H); 8.15 (C*H*, bs, 1H); 8.32 (C*H*, s, 1H); 8.64 (C*H*–N_3, s, 1H). **ESI-HRMS:** 1251.6313 [M+H]$^+$ (theor. $[C_{66}H_{79}N_{18}O_8]^+$ = 1251.6323). **UV absorption:** λ_{max} = 276 nm.

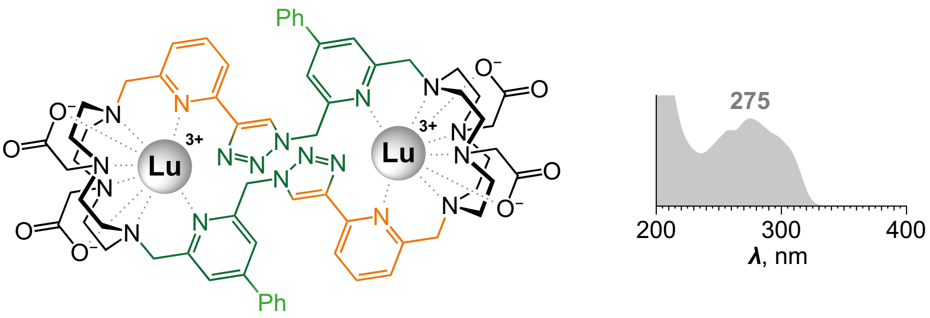

***1,4*/*1,4-cz*-[Lu(PhL¹)]₂**

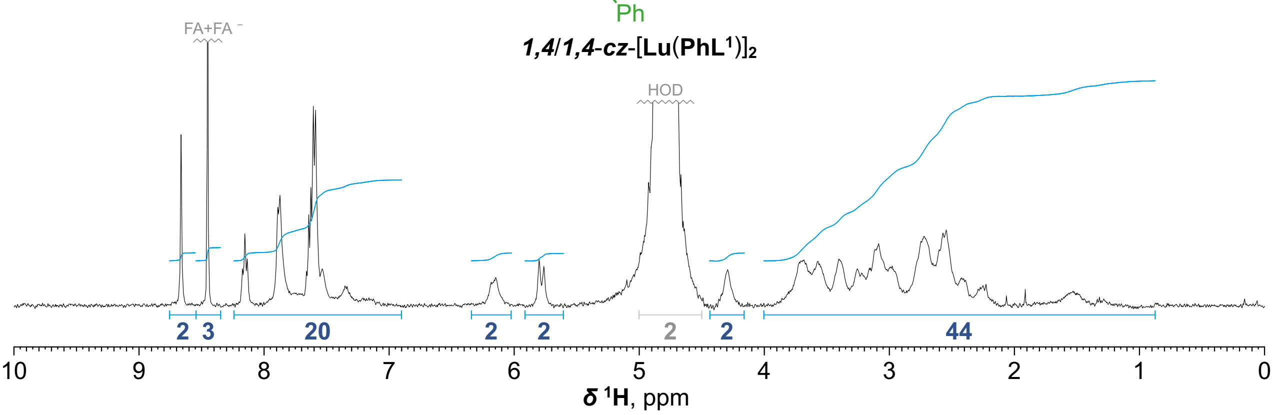

Supplementary Fig. 89. Synthesis and characterization of *1,4/1,4-cz*-[Lu(PhL¹)]. In a glass vial (20 mL), ***1,4-open*-(PhL¹)₂**·0.2FA·*x*H_2O (5.0 mg; ~4 µmol; 1.0 equiv.) was dissolved in H_2O (14 mL) followed by addition aq. MOPS/NaOH buffer (500 mM; pH 7.0; 800 µL; 400 mmol; ~100 equiv.) and aq. $LuCl_3$ (100 mM; 88 µL; 8.8 µmol; ~2.2 equiv.) and the mixture was stirred for 1 h at 80 °C. Mixture was then filtered through syringe microfilter (RC). The filtrate was concentrated on rotary evaporator and then directly purified by preparative HPLC (C18; H_2O–MeCN gradient with FA additive). Fractions with product were joined and directly lyophilized to give product in the form of formate salt as white fluffy solid. **Yield:** 4 mg (~60%; 1 step; based on ***1,4-open*-(PhL¹)₂**·0.2FA·*x*H_2O). **NMR (D_2O, borderline solubility):** **¹H** (401.0 MHz) δ_H 0.87–4.00 (CH_2–N, CH_2–CO, CH_2–arom, m, 32+8+4H); 4.29 (CH_2–arom., bs, 2H); ~4.5–5.0 (CH_2–arom., 2H, obscured by HOD signal); 5.78 (CH_2–N_3, bd, 2H, $^2J_{HH}$ = 15); 6.16 (CH_2–N_3, bs, 2H); 6.93–8.23 (C*H,* m, 20H); 8.45 (*FA*⁻, s); 8.66 (C*H*–N_3, s, 2H). **ESI-HRMS:** 798.2368 $[M]^{2+}$ (theor. $[C_{66}H_{74}N_{18}O_8Lu_2]^{2+}$ = 798.2371). **UV absorption:** λ_{max} = 275 nm.

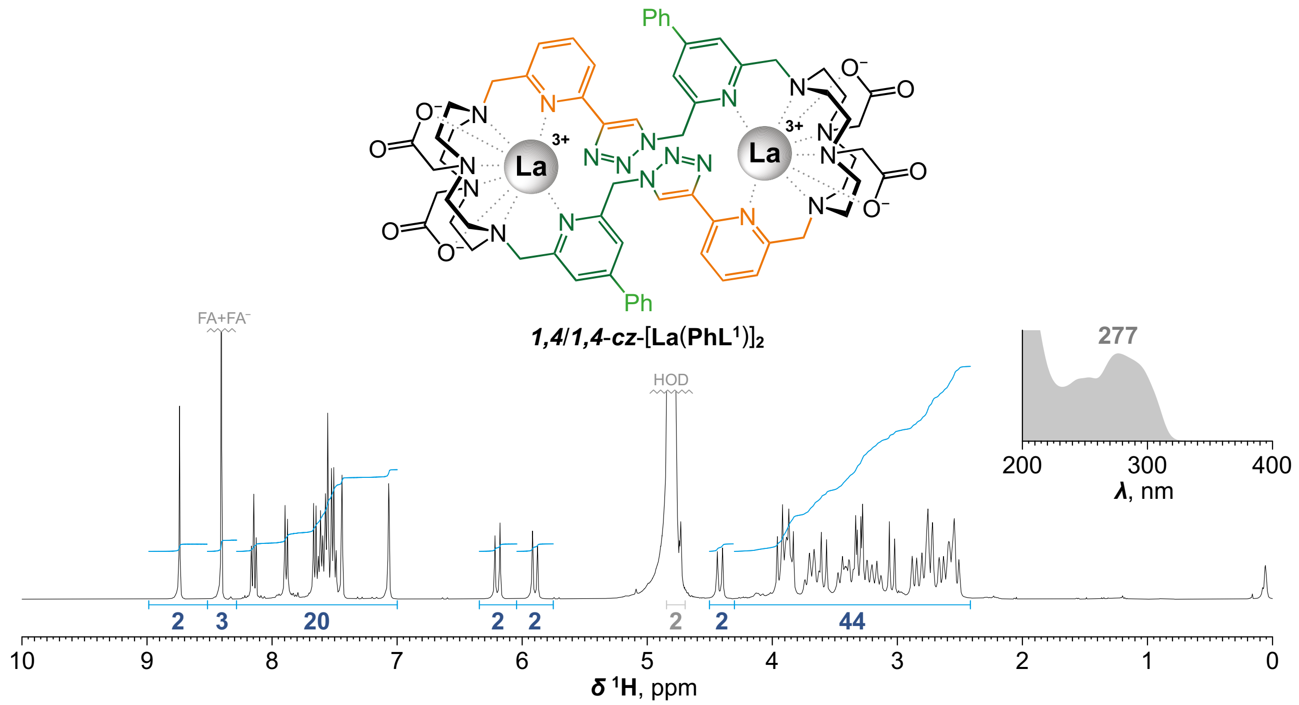

Supplementary Fig. 90. Characterization of byproduct *1,4*/*1,4*-*cz*-[La(PhL[1])]$_2$. Obtained during synthesis of **Ph**{**La**} in the form of formate salt as white fluffy solid. **Yield:** 6 mg. **NMR (D_2O, pD ~5):** **^{1}H** (401.0 MHz) δ_H 2.42–4.27 (CH_2–N, CH_2–CO, CH_2–arom, m, 32+8+4H); 4.42 (CH_2–arom., d, 2H, $^2J_{HH}$ = 17); 4.75 (CH_2–arom., d, 2H, $^2J_{HH}$ = 16.7); 5.90 (CH_2–N_3, d, 2H, $^2J_{HH}$ = 16.3); 6.20 (CH_2–N_3, d, 2H, $^2J_{HH}$ = 16.3); 7.07 (C*H*, d, 2H, $^4J_{HH}$ = 1.7); 7.44 (C*H*, d, 2H, $^4J_{HH}$ = 1.7); 7.47–7.63 (C*H*, m, 10H); 7.66 (C*H*, d, 2H, $^3J_{HH}$ = 7.9); 7.89 (C*H*, d, 2H, $^3J_{HH}$ = 7.9); 8.15 (C*H*, t, 2H, $^3J_{HH}$ = 7.9); 8.41 (*FA*$^-$, s); 8.74 (C*H*–N_3, s, 2H). **^{13}C{^{1}H}** (100.8 MHz) δ_C 49.3 (CH_2–N, s); 49.4 (CH_2–N, s); 51.0 (CH_2–N, s); 51.6 (CH_2–N, s); 52.5 (CH_2–N, s); 53.9 (CH_2–N, s); 54.0 (CH_2–N, s); 54.3 (CH_2–N, s); 55.6 (CH_2–N_3, s); 59.2 (CH_2–arom., s); 59.8 (CH_2–CO, s); 61.2 (CH_2–arom., s); 61.9 (CH_2–CO, s); 120.7 (*C*H, s); 121.2 (*C*H, s); 122.2 (*C*H, s); 125.4 (*C*H, s); 126.7 (*C*H–N_3, s); 128.2 (*C*H, s); 129.6 (*C*H, s); 131.6 (*C*H, s); 135.2 (*C*, s); 143.0 (*C*H, s); 145.5 (*C*, s); 148.9 (*C*, s); 150.2 (*C*, s); 157.2 (*C*, s); 157.3 (*C*, s); 158.9 (*C*, s); 170.8 (*FA*$^-$, s); 177.9 (*C*O, s); 180.5 (*C*O, s). **ESI-HRMS:** 762.2025 $[M]^{2+}$ (theor. $[C_{66}H_{74}N_{18}O_8La_2]^{2+}$ = 762.2027). **UV absorption:** λ_{max} = 277 nm.

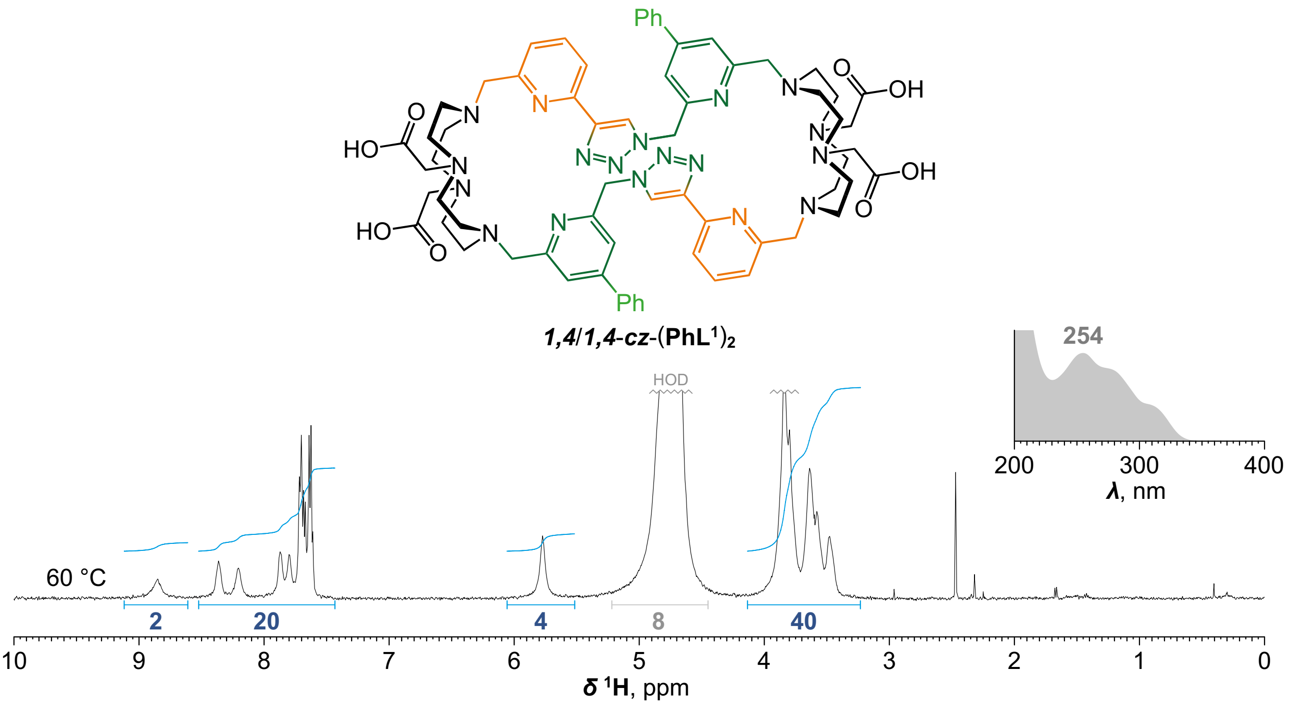

Supplementary Fig. 91. Characterization of byproduct *1,4/1,4-cz*-(PhL^1)$_2$. In a glass vial (4 mL), ***1,4/1,4-cz*-[La(PhL^1)]$_2$**$^+$2[FA]$^-$·xH$_2$O (1.2 mg; <0.7 µmol; 1.0 equiv.) was dissolved in 0.1M aq. HCl (500 µL) and the mixture was stirred for 16 h at RT. Mixture was then diluted with MeOH (500 µL) and TFA (100 µL) and then directly purified by preparative HPLC (C18; H$_2$O–MeCN gradient with TFA additive). Fractions with product were joined and directly lyophilized to give product in the form of trifluoroacetate salt as white fluffy solid. **Yield:** <1 mg. **NMR (D$_2$O, acidified with 1 µL TFA, 60 °C):** ^{1}**H** (500.0 MHz) δ_H 3.22–4.12 (C*H*$_2$–N, C*H*$_2$–CO, bm, 32+8H); ~4.4–5.2 (C*H*$_2$–arom, obscured by HOD signal, bm, 8H); 5.77 (C*H*$_2$–N$_3$, s, 4H); 7.42–8.54 (C*H*, 20H); 8.86 (C*H*–N$_3$, bs, 2H). **ESI-HRMS:** 1251.6300 [M+H]$^+$ (theor. [$C_{66}H_{79}N_{18}O_8$]$^+$ = 1251.6323). **UV absorption:** λ_{max} = 254 nm.

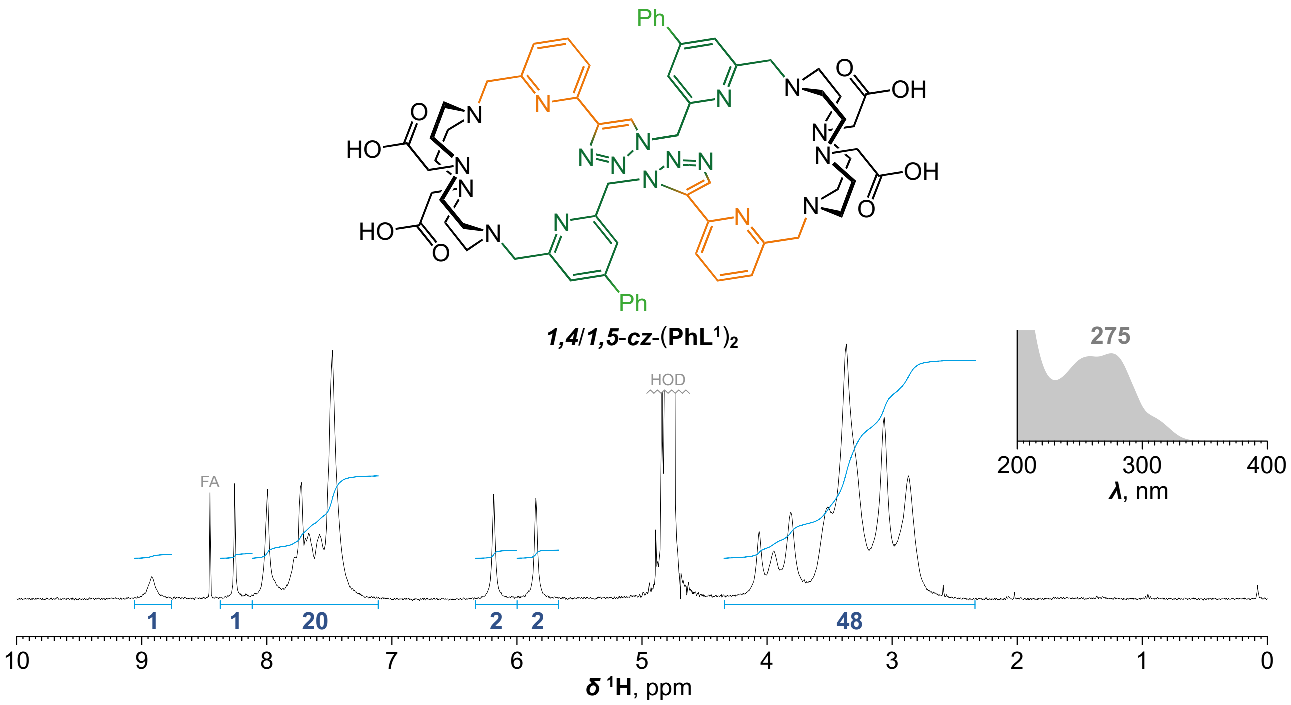

Supplementary Fig. 92. Characterization of byproduct *1,4*/*1,5*-*cz*-(PhL[1])$_2$. Obtained during synthesis of ***1,4*-*cz*-PhL[1]** in the form of formate salt as white solid. **Yield:** 4 mg. **NMR (D_2O): ^{1}H** (401.0 MHz) δ_H 2.33–4.35 (CH_2–N, CH_2–CO, CH_2–arom, bm, 32+8+8H); 5.85 (CH_2–N_3, s, 2H); 6.19 (CH_2–N_3, s, 2H); 7.10–8.11 (C*H*, m, 20H); 8.26 (C*H*–N_3, s, 1H); 8.92 (C*H*–N_3, s, 1H). **ESI-MS** (LC-MS)**:** 626.5 $[M+2H]^{2+}$ (theor. $[C_{66}H_{80}N_{18}O_8]^{2+}$ = 626.3); 418.1 $[M+3H]^{3+}$ (theor. $[C_{66}H_{81}N_{18}O_8]^{3+}$ = 417.9). **UV absorption:** λ_{max} = 275 nm.

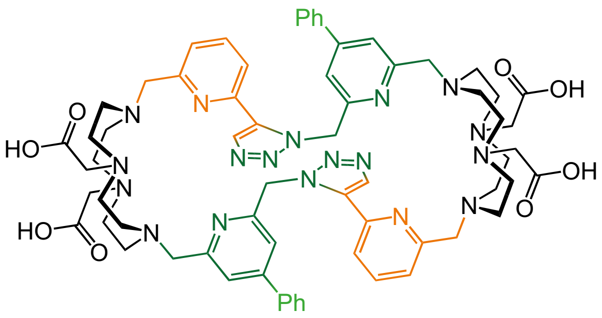

***1,5*/*1,5-cz*-(PhL¹)₂**

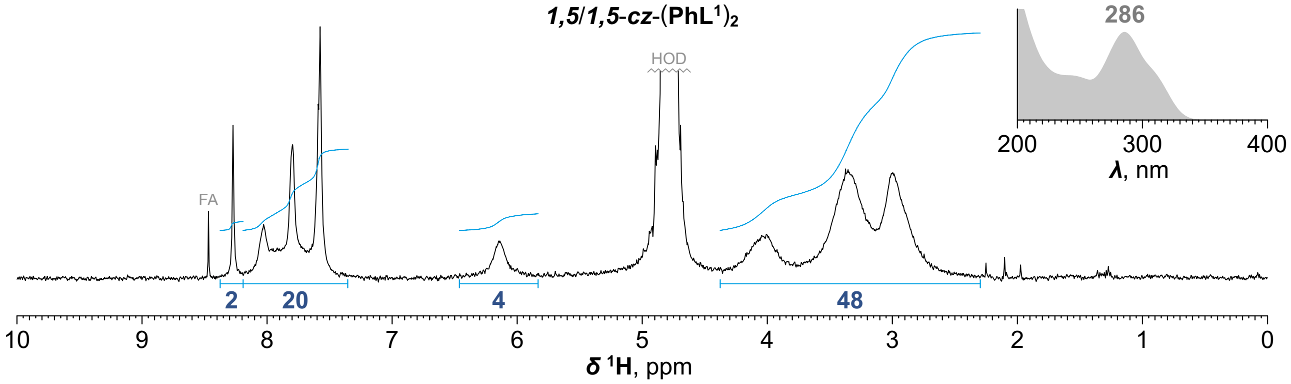

Supplementary Fig. 93. Characterization of byproduct *1,5*/*1,5-cz*-(PhL[1])$_2$. Obtained during synthesis of ***1,4-cz*-PhL[1]** in the form of formate salt as white solid. **Yield:** 3 mg. **NMR (D_2O):** **^{1}H** (401.0 MHz) δ_H 2.29–4.38 (C*H*$_2$–N, C*H*$_2$–CO, C*H*$_2$–arom, bm, 32+8+8H); 6.14 (C*H*$_2$–N$_3$, bs, 4H); 7.33–8.17 (C*H*, m, 20H); 8.27 (C*H*–N$_3$, s, 1H). **ESI-MS** (LC-MS)**:** 626.6 $[M+2H]^{2+}$ (theor. $[C_{66}H_{80}N_{18}O_8]^{2+}$ = 626.3); 418.0 $[M+3H]^{3+}$ (theor. $[C_{66}H_{81}N_{18}O_8]^{3+}$ = 417.9). **UV absorption:** λ_{max} = 286 nm.

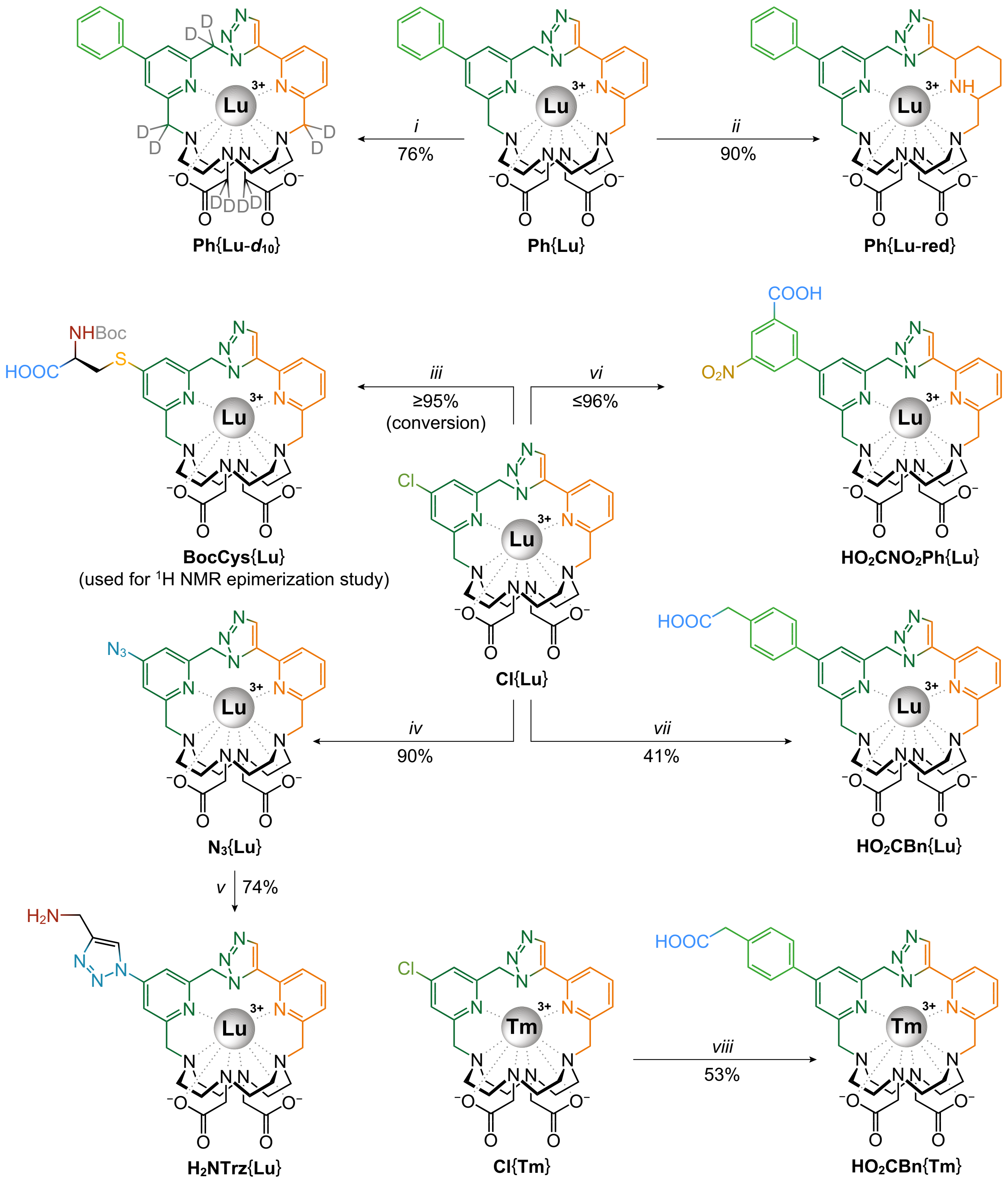

Supplementary Fig. 94. Post ClickZip synthesis examples. The Ln^{III} ion is so well encapsulated in ClickZip that broad range of chemical reactions can be performed on its surface. No decomplexation was observed during any of these transformations. ***Conditions:*** (*i*) DBU, D_2O, 80 °C; (*ii*) $NaBH_4$, MeOH, RT; (*iii*) *N*-Boc-cysteine, DIPEA, DMSO, RT; (*iv*) NaN_3, DMSO, 80 °C; (*v*) propargyl amine, $CuSO_4$ (cat.), sodium ascorbate, MES/NaOH buffer (pH = 5.2), H_2O, RT; (*vi*) *3*-borono-*5*-nitrobenzoic acid, XPhos Pd G2 (cat.), K_3PO_4, DMF, H_2O, 80 °C; (*vii*) *4*-(carboxymethyl)phenylboronic acid pinacol ester, XPhos Pd G2 (cat.), K_3PO_4, DMF, H_2O, 80 °C; (*viii*) *2*-(*4*-boronophenyl)acetic acid, XPhos Pd G2 (cat.), K_3PO_4, DMF, H_2O, 80 °C. Yields refer to isolated compounds (combined yield of two isolated isomers of **Ph{Lu-red}**) except for **BocCys{Lu}** where conversion is given instead.

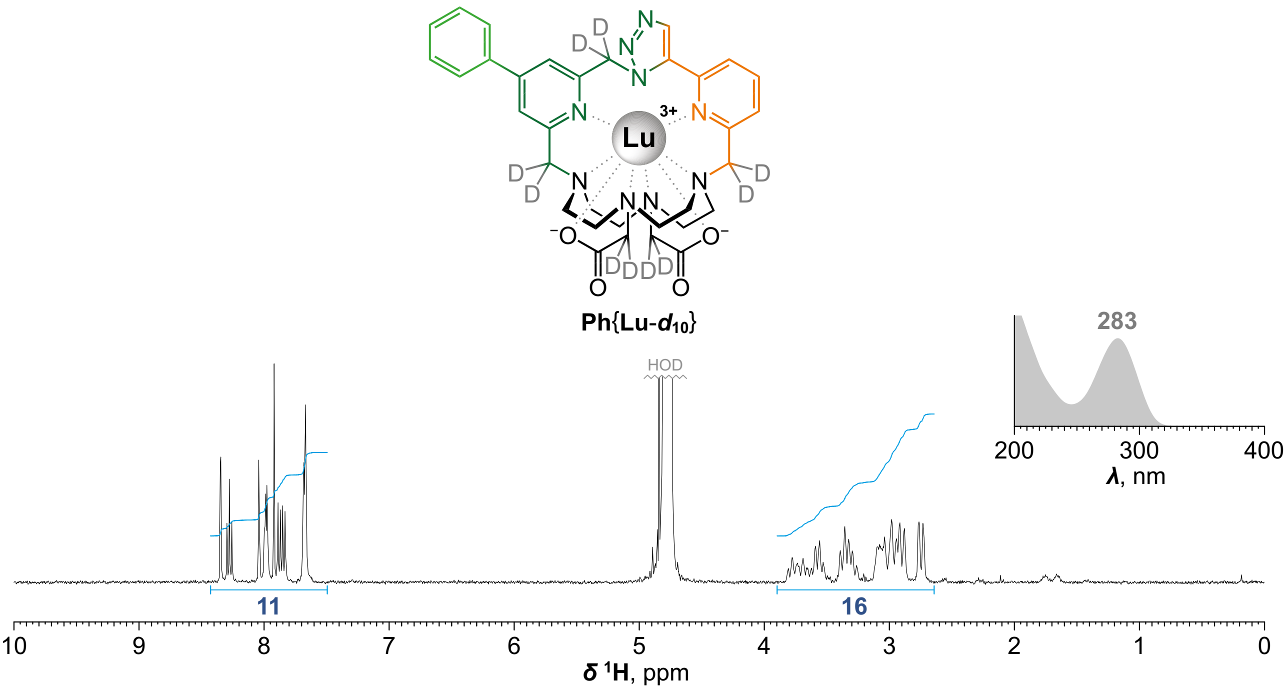

Supplementary Fig. 95. Synthesis and characterization of Ph{Lu-d_{10}}. In a glass vial (4 mL), [**Ph**{**Lu**}]$^+$[FA]$^-$·5.5H_2O (9.0 mg; 9.5 µmol; 1.0 equiv.) was dissolved in D_2O (500 µL) and evaporated to dryness. Residue was re-dissolved in D_2O (500 µL) followed by addition of neat DBU (29 µL; 194 µmol; 20 equiv.) and the resulting solution was stirred for 2 d at 80 °C. Mixture was then evaporated to dryness. Residue was re-dissolved in D_2O (500 µL) followed by addition of another portion of neat DBU (15 µL; 100 µmol; ~10 equiv.) and the resulting solution was further stirred for 16 h at 80 °C. The resulting mixture (no degradation products were observed) was then quenched by addition of FA (75 µL; 2.92 mmol; ~300 equiv.) and directly purified by preparative HPLC (C18; H_2O–MeCN gradient with FA additive). Fractions with product were joined and directly lyophilized to give crude product in the form of formate salt as white fluffy solid (^{1}H NMR analysis found ~1 equiv. of DBU or its byproducts apart from otherwise pure product), which was further purified by preparative HPLC (C18; H_2O–MeCN gradient with TFA additive). Fractions with product were joined and directly lyophilized to give product in the form of trifluoroacetate salt as white fluffy solid. **Yield:** 7.6 mg (76%; 1 step; based on [**Ph**{**Lu**}]$^+$[FA]$^-$·5.5H_2O). **NMR (D_2O, pD ~4): ^{1}H** (400.1 MHz) δ_H 2.64–3.83 (C*H*$_2$–N, m, 16H); 7.55–7.63 (C*H*, m, 3H); 7.84 (C*H*, dd, 1H, $^3J_{HH}$ = 7.9, $^4J_{HH}$ = 1.2); 7.88 (C*H*, dd, 1H, $^3J_{HH}$ = 7.9, $^4J_{HH}$ = 1.2); 7.92 (C*H*–N_3, s, 1H); 7.90–8.00 (C*H*, m, 2H); 8.04 (C*H*, d, 1H, $^4J_{HH}$ = 1.7); 8.28 (C*H*, t, 1H, $^3J_{HH}$ = 7.9); 8.35 (C*H*, d, 1H, $^4J_{HH}$ = 1.7). **ESI-HRMS:** 808.3004 [M]$^+$ (theor. [$C_{33}H_{27}D_{10}N_9O_4Lu_1$]$^+$ = 808.2998). **UV absorption:** λ_{max} = 283 nm. **EA** ([$C_{33}H_{27}D_{10}N_9O_4Lu_1$]$^+$[TFA]$^-$·0.4TFA·4.8$H_2O$, M_R = 1053.8; H was excluded from the fitting): C 40.8 (41.2); N 12.0 (11.6); F 7.6 (7.4).

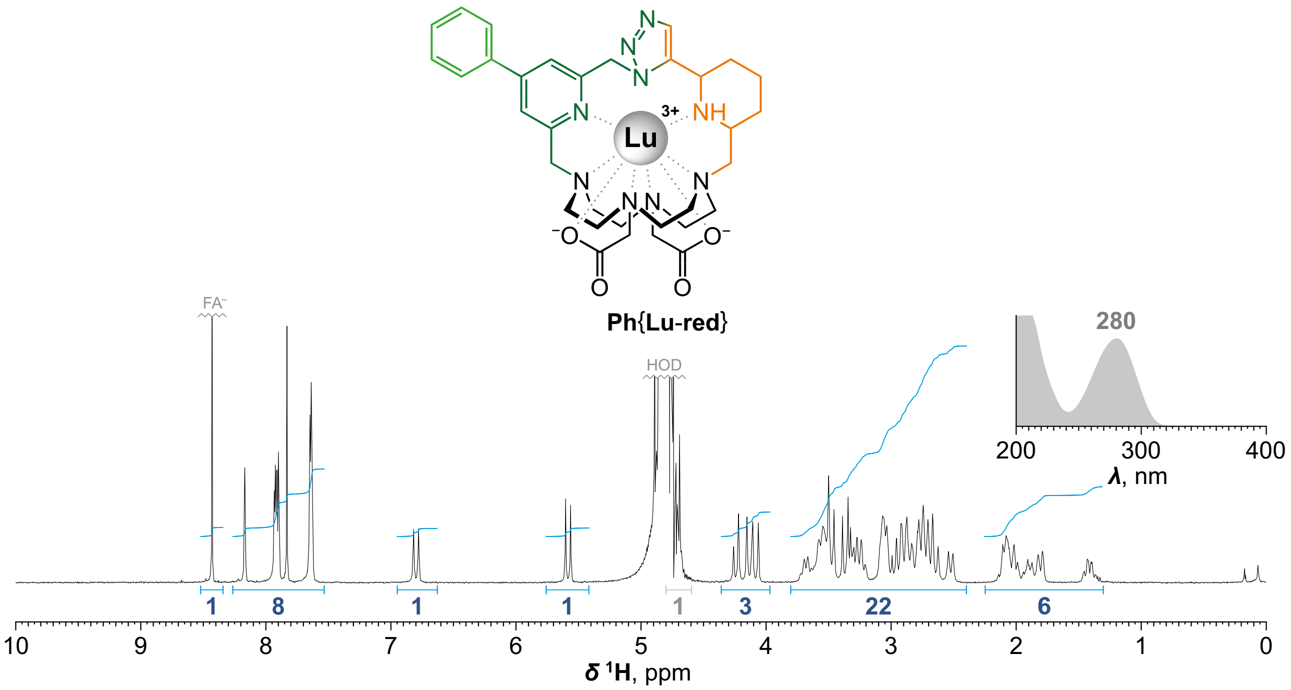

Supplementary Fig. 96. Synthesis and characterization of Ph{Lu-red}. In a glass vial (20 mL), [**Ph**{**Lu**}]$^+$[FA]$^-$·5.5H_2O (12.3 mg; 13.1 µmol; 1.0 equiv.) was dissolved in MeOH (1.3 mL) followed by addition of solid $NaBH_4$ (49.4 mg; 1.31 mmol; 100 equiv.). The resulting open vial was stirred for 1 h at RT. Then additions of $NaBH_4$ (49.4 mg; 1.31 mmol; 100 equiv.) and MeOH (650 µL) were periodically added every 1 h until all starting material was consumed (10 additions in total; analyzed by LC-MS). The mixture (containing two isomers of **Ph**{**Lu-red**} in ~4:1 ratio) was then evaporated to dryness and the residue was re-dissolved in H_2O (3 mL). The resulting solution was then neutralized with neat FA (540 µL; 14.3 mmol; ~1100 equiv.). The resulting suspension was further diluted with MeOH (3 mL) and H_2O (12 mL) and filtered through syringe microfilter (RC). Filtrate was directly purified by preparative HPLC (C18; H_2O–MeCN gradient with FA additive). Fractions with products were joined and directly lyophilized to give products in the form of formate salts as white fluffy solids. **Yield:** 8.9 mg of major isomer + 2.6 mg of minor isomer (90%; 1 step; combined yield; based on [**Ph**{**Lu**}]$^+$[FA]$^-$·5.5H_2O). **NMR (D_2O, major isomer, pD ~6): ^{1}H** (400.1 MHz) δ_H 1.28–2.24 (C*H*$_2$, m, 6H); 2.39–3.78 (C*H*$_2$–N, C*H*$_2$–CO, C*H*$_2$, C*H*, m, 16+4+1+1H); 4.08 (C*H*$_2$, d, 1H, $^2J_{HH}$ = 17.4); 4.13 (C*H*$_2$–arom., d, 1H, $^2J_{HH}$ = 15.7); 4.24 (C*H*$_2$–arom., d, 1H, $^2J_{HH}$ = 15.7); ~4.65–4.75 (C*H*, obscured by HOD signal, m, 1H); 5.58 (C*H*$_2$–N_3, d, 1H, $^2J_{HH}$ = 16.1); 6.80 (C*H*$_2$–N_3, d, 1H, $^2J_{HH}$ = 16.1); 7.59–7.68 (C*H*, m, 3H); 7.83 (C*H*–N_3, s, 1H); 7.90 (C*H*, d, 1H, $^4J_{HH}$ = 1.8); 7.90–7.96 (C*H*, m, 2H); 8.17 (C*H*, d, 1H, $^4J_{HH}$ = 1.8); 8.43 (*FA*$^-$, s, 1H). **^{13}C{^{1}H}** (100.8 MHz) δ_C 22.8 (*C*H$_2$, s); 27.0 (*C*H$_2$, s); 29.5 (*C*H$_2$, s); 50.1 (*C*H, s); 51.4 (*C*H$_2$–N_3, s); 52.1 (*C*H$_2$–N, s); 53.4 (*C*H$_2$–N, s); 54.4 (*C*H$_2$–N, s); 54.5 (*C*H, s); 55.4 (*C*H$_2$–N, s); 55.6 (*C*H$_2$–N, s); 56.0 (*C*H$_2$–N, s); 56.1 (*C*H$_2$–N, s); 56.8 (*C*H$_2$–N, s); 56.0 (*C*H$_2$, s); 64.7 (*C*H$_2$–CO, s); 64.8 (*C*H$_2$–arom., s); 65.4 (*C*H$_2$, s); 119.8 (*C*H, s); 123.7 (*C*H, s); 126.9 (*C*H, s); 129.0 (*C*H, s); 130.5 (*C*H, s); 131.6 (*C*H–N_3, s); 135.7 (*C*, s); 138.9 (*C*, s); 153.1 (*C*, s); 155.9 (*C*, s); 159.0 (*C*, s); 170.5 (*FA*$^-$, s); 178.8 (*C*O, s); 179.5 (*C*O, s). **ESI-HRMS:** 804.2841 [M]$^+$ (theor. [$C_{33}H_{43}N_9O_4Lu_1$]$^+$ = 804.2840). **UV absorption (both isomers):** λ_{max} = 280 nm. **EA (major isomer,** [$C_{33}H_{43}N_9O_4Lu_1$]$^+$[FA]$^-$·6.9H_2O, M_R = 974.0): C 41.9 (41.5); H 6.0 (5.6); N 12.9 (12.8). **EA (minor isomer,** [$C_{33}H_{43}N_9O_4Lu_1$]$^+$[FA]$^-$·7.5H_2O, M_R = 984.8): C 41.5 (40.8); H 6.0 (5.4); N 12.8 (12.2).

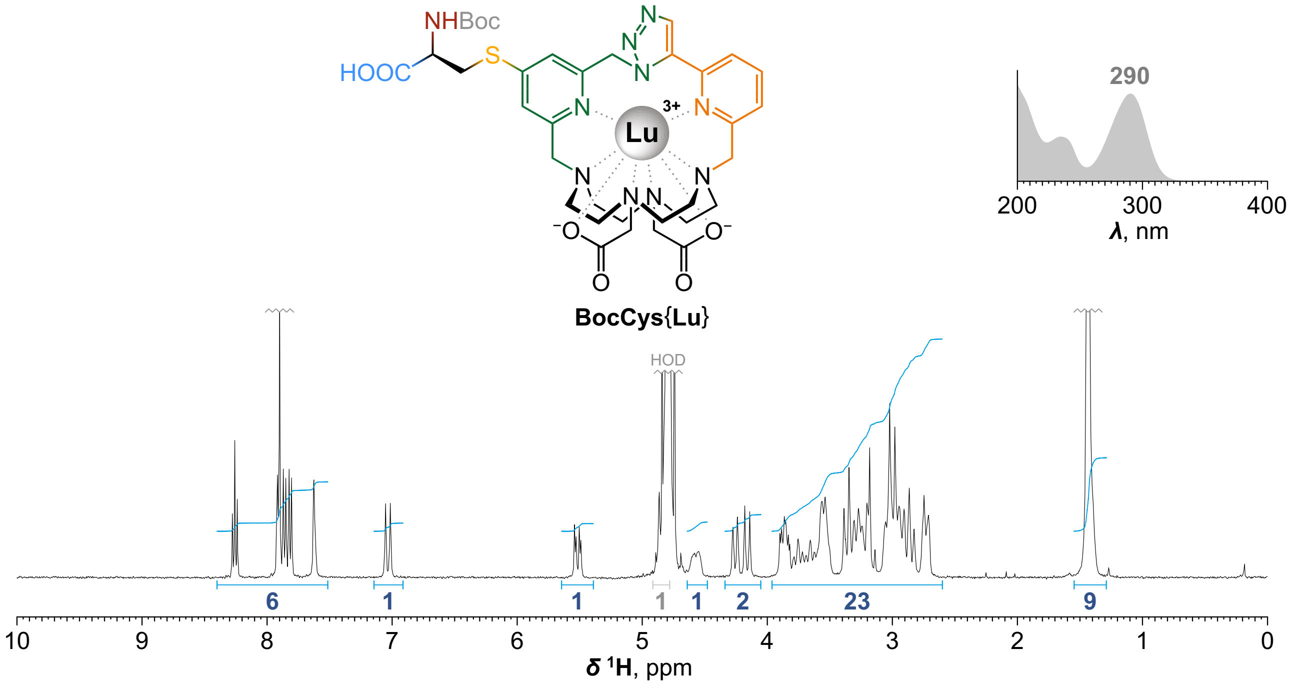

Supplementary Fig. 97. Synthesis of BocCys{Lu} and its epimer separation. In a glass vial (4 mL), [**Cl**{**Lu**}]$^+$[TFA]$^-$·5.1H_2O (4.0 mg; 4.2 µmol; 1.0 equiv.) was dissolved in DMSO (160 µL) followed by addition of *N*-Boc-cysteine (200 mM in DMSO; 31 µL; 6.2 µmol; 1.5 equiv.) and DIPEA (7.3 µL; 42 µmol; 10 equiv.) and the resulting solution was stirred for 16 h at 40 °C. Mixture (containing racemic mixture of **BocCys**{**Lu**} epimers) was then diluted with DMSO (0.5 mL) and filtered through syringe microfilter (RC). Filtrate was then directly purified by preparative HPLC (C18; H_2O–MeCN gradient with TFA additive). Fractions with epimer A (front part of the first eluting peak) and B (latter part of the second eluting peak) were quickly freezed and directly lyophilized. The resulting solids were dissolved in 500 µL of deuterated 50 mM MOPS/NaOD buffer (formal pH 7.4; obtained from MOPS/NaOH buffer of such pH by multiple evaporation/D_2O resupply cycles). Epimer A (obtained in slightly higher epimeric purity than epimer B) was used for continuous ^{1}H NMR epimerization study at 37 °C (Supplementary Fig. 22) while epimer B was incubated concurrently under identical conditions on heated magnetic stirrer. **Conversion:** ≥95% (based on **Cl**{**Lu**}). **NMR (racemix mixture of epimers from smaller scale parallel synthesis, D_2O, pD ~3): ^{1}H** (400.1 MHz) δ_H 1.28–1.54 (C*H*$_3$, m, 9H); 2.63–3.95 (C*H*$_2$–N, C*H*$_2$–CO, C*H*$_2$–S, C*H*$_2$–arom., m, 16+4+2+1H); 4.16 (C*H*$_2$–arom., d, 1H, $^2J_{HH}$ = 15.8); 4.26 (C*H*$_2$–arom., d, 1H, $^2J_{HH}$ = 14.7); 4.48–4.63 (NH–C*H*–COOH, m, 1H); 4.84 (C*H*$_2$–arom., d, 1H, $^2J_{HH}$ = 15.8); 5.45–5.59 (C*H*$_2$–N_3, m, 1H); 7.03 (C*H*$_2$–N_3, d, 1H, $^2J_{HH}$ = 15.2); 7.59–7.65 (C*H*, m, 1H); 7.81 (C*H*, dd, 1H, $^3J_{HH}$ = 7.9, $^4J_{HH}$ = 1.1); 7.86 (C*H*, dd, 1H, $^3J_{HH}$ = 7.9, $^4J_{HH}$ = 1.1); 7.90 (C*H*–N_3, s, 1H); 7.90–7.95 (C*H*, m, 1H); 8.26 (C*H*, t, 1H, $^3J_{HH}$ = 7.9). **ESI-HRMS:** 941.2614 [M]$^+$ (theor. [$C_{35}H_{46}N_{10}O_8S_1Lu_1$]$^+$ = 941.2623). **UV absorption:** λ_{max} = 290 nm.

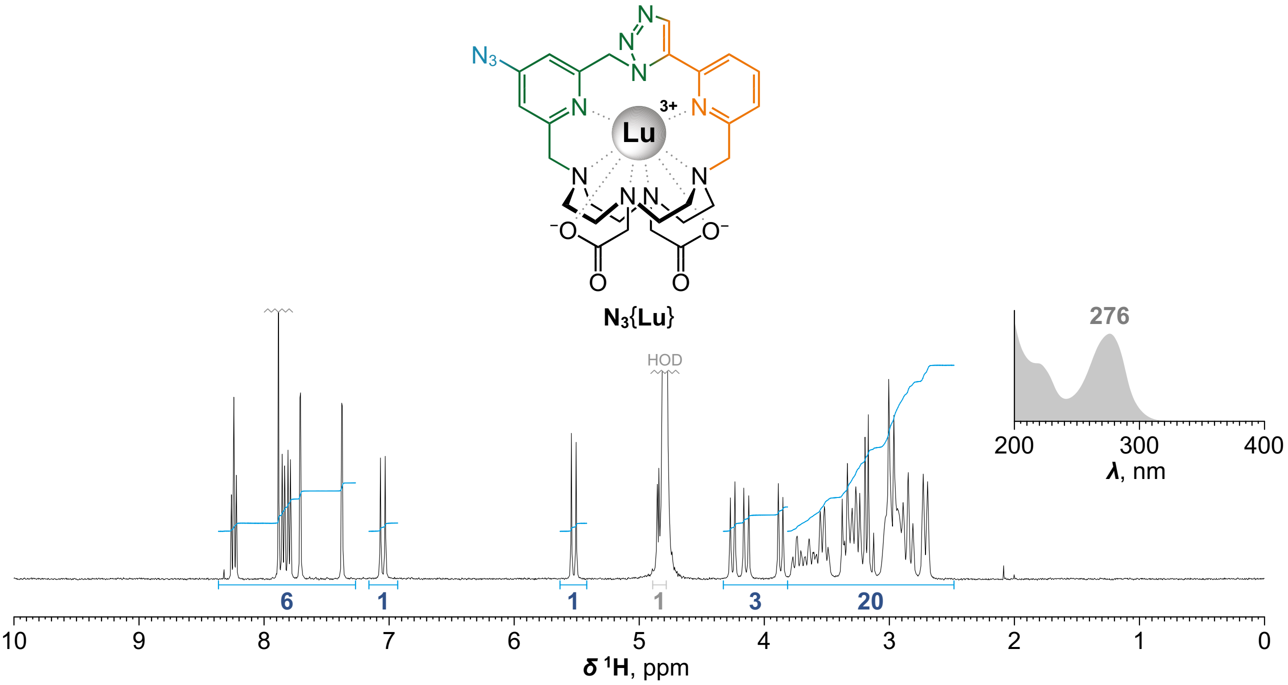

Supplementary Fig. 98. Synthesis and characterization of N_3{Lu}. In a glass vial (4 mL), [**Cl**{**Lu**}]$^+$[TFA]$^-$·5.1H_2O (20.0 mg; 20.8 µmol; 1.0 equiv.) and NaN_3 (13.5 mg; 208 µmol; 10 equiv.) were dissolved in DMSO (415 µL) and the mixture was stirred for 30 min at 80 °C. Mixture was diluted H_2O (3 mL) and directly purified by preparative HPLC (C18; H_2O–MeCN gradient with TFA additive). Fractions with product were joined and directly lyophilized to give product in the form of trifluoroacetate salt as white fluffy solid. **Yield:** 18.6 mg (90%; 1 step; based on [**Ph**{**Lu**}]$^+$[TFA]$^-$·5.1H_2O). **NMR (D_2O, pD ~4):** **^{1}H** (401.0 MHz) δ_H 2.62–3.81 (CH_2–N, CH_2–CO, m, 16+4H); 3.87 (CH_2–arom., d, 1H, $^2J_{HH}$ = 14.8); 4.14 (CH_2–arom., d, 1H, $^2J_{HH}$ = 15.8); 4.25 (CH_2–arom., d, 1H, $^2J_{HH}$ = 14.8); 4.83 (CH_2–arom., d, 1H, $^2J_{HH}$ = 15.8); 5.52 (CH_2–N_3, d, 1H, $^2J_{HH}$ = 15.3); 7.05 (CH_2–N_3, d, 1H, $^2J_{HH}$ = 15.3); 7.38 (C*H*, d, 1H, $^4J_{HH}$ = 2.1); 7.71 (C*H*, d, 1H, $^4J_{HH}$ = 2.1); 7.80 (C*H*, dd, 1H, $^3J_{HH}$ = 7.9, $^4J_{HH}$ = 1.3); 7.84 (C*H*, dd, 1H, $^3J_{HH}$ = 7.9, $^4J_{HH}$ = 1.3); 7.88 (C*H*–N_3, s, 1H); 8.24 (C*H*, t, 1H, $^3J_{HH}$ = 7.9). **ESI-HRMS:** 763.2075 [M]$^+$ (theor. [$C_{27}H_{32}N_{12}O_4Lu_1$]$^+$ = 763.2072). **UV absorption:** λ_{max} = 276 nm. **EA** ([$C_{27}H_{32}N_{12}O_4Lu_1$]$^+$[TFA]$^-$·0.2TFA·5.2$H_2O$, M_R = 993.0): C 35.6 (35.6); H 4.3 (4.0); N 16.9 (16.6); F 6.9 (6.8).

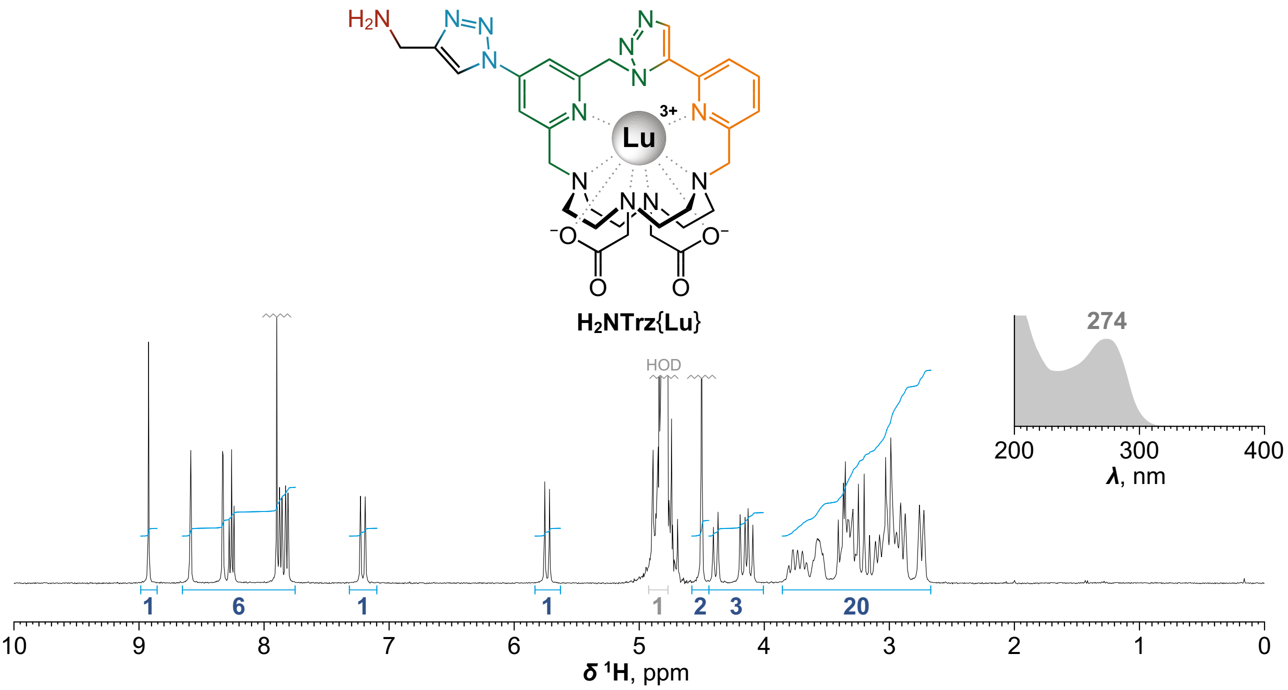

Supplementary Fig. 99. Synthesis and characterization of NH_2Trz{Lu}. In a glass vial (4 mL), [N_3{**Lu**}]$^+$[TFA]$^-$·0.2TFA·5.2H_2O (13.0 mg; 13.1 µmol; 1.0 equiv.) was dissolved in aq. MES/NaOH buffer (500 mM; pH 5.2; 2.62 mL; 1.31 mmol; 100 equiv.) followed by addition of propargylamine (8.3 µL; 131 µmol; 10 equiv.) and aq. solution of $CuSO_4$ (100 mM; 66 µL; 6.6 µmol; 50 mol%). Argon was briefly bubbled through the solution followed by addition of freshly prepared aq. solution of sodium ascorbate (200 mM; 66 µL; 13.2 µmol; 1.0 equiv.) and the mixture was stirred for 24 h at RT. The mixture was then directly purified by preparative HPLC (C18; H_2O–MeCN gradient with TFA additive). Fractions with product were joined and directly lyophilized to give product in the form of trifluoroacetate salt as white fluffy solid. **Yield:** 11.5 mg (74%; 1 step; based on [N_3{**Lu**}]$^+$[TFA]$^-$·0.2TFA·5.2H_2O). **NMR (D_2O, pD ~2):** **^{1}H** (400.1 MHz) δ_H 2.66–3.86 (CH_2–N, CH_2–CO, m, 16+4H); 4.11 (CH_2–arom., d, 1H, $^2J_{HH}$ = 15.1); 4.17 (CH_2–arom., d, 1H, $^2J_{HH}$ = 16.0); 4.39 (CH_2–arom., d, 1H, $^2J_{HH}$ = 15.1); 4.50 (CH_2–NH_2, s, 2H); 4.87 (CH_2–arom., d, 1H, $^2J_{HH}$ = 16.0); 5.74 (CH_2–N_3, d, 1H, $^2J_{HH}$ = 15.3); 7.21 (CH_2–N_3, d, 1H, $^2J_{HH}$ = 15.3); 7.82 (C*H*, dd, 1H, $^3J_{HH}$ = 7.9, $^4J_{HH}$ = 1.1); 7.87 (C*H*, dd, 1H, $^3J_{HH}$ = 7.9, $^4J_{HH}$ = 1.1); 7.90 (C*H*–N_3, s, 1H); 8.26 (C*H*, t, 1H, $^3J_{HH}$ = 7.9); 8.33 (C*H*, d, 1H, $^4J_{HH}$ = 2.0); 8.59 (C*H*, d, 1H, $^4J_{HH}$ = 2.0); 8.92 (C*H*–N_3, s, 1H). **ESI-HRMS:** 818.2501 [M]$^+$ (theor. [$C_{30}H_{37}N_{13}O_4Lu$]$^+$ = 818.2494). **UV absorption:** λ_{max} = 274 nm. **EA** ([$C_{30}H_{37}N_{13}O_4Lu_1$]$^+$[TFA]$^-$·1.5TFA·4.6$H_2O$, M_R = 1185.5): C 35.5 (35.4); H 4.1 (3.7); N 15.4 (15.0); F 12.0 (11.7).

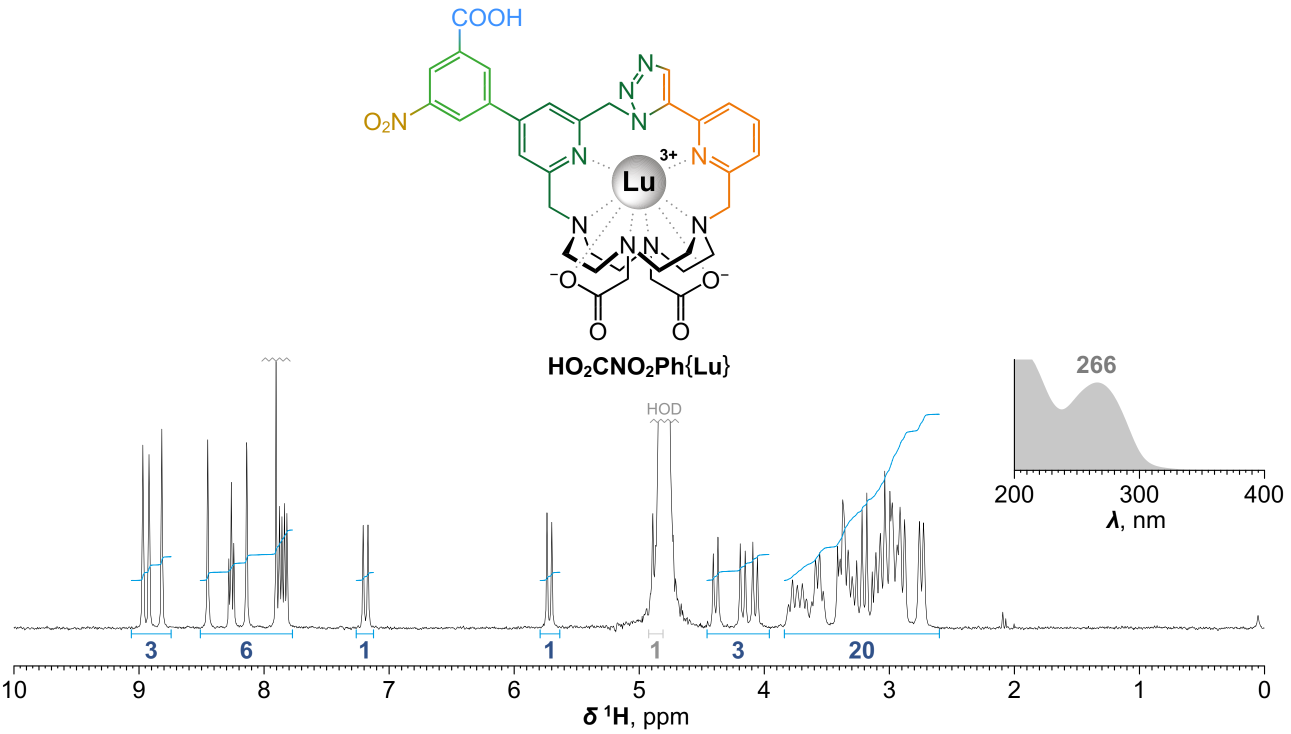

Supplementary Fig. 100. Synthesis and characterization of HO_2CNO_2Ph\{Lu\}. Glass vial (4 mL) was charged with [**Cl**\{**Lu**\}]$^+$[TFA]$^-$·5.1H_2O (10.0 mg; 10.4 µmol; 1.0 equiv.), *3*-borono-*5*-nitrobenzoic acid (4.4 mg; 21 µmol; 2.0 equiv.) and XPhos Pd G2 (0.5 mg; ~0.6 µmol; 6 mol%) and was then three times secured with argon. Under constant flow of argon was then added dry DMF (420 µL) through septum followed by addition of freshly prepared (and briefly washed with argon prior addition) aq. solution of $K_3PO_4 \cdot H_2O$ (326 mM; 160 µL; 52 µmol; 5.0 equiv.). The mixture was then stirred for 16 h under septum (but without external argon) at 80 °C. Resulting heterogenous mixture was diluted with H_2O (2 mL) and the resulting slightly opalescent pale-yellow solution was filtered through syringe microfilter (RC). Filtrate was then directly purified by preparative HPLC (C18; H_2O–MeCN gradient with TFA additive). Fractions with product were joined and directly lyophilized to give product as white fluffy solid. **Yield:** 10 mg (≤96% assuming [**M**]$^+$[TFA]$^-$·$x$$H_2O$; M_R = 1000.7; 1 step; based on [**Cl**\{**Lu**\}]$^+$[TFA]$^-$·5.1H_2O). **NMR (D_2O, pD ~3): ^{1}H** (401.0 MHz) δ_H 2.66–3.84 (CH_2–N, CH_2–CO, m, 16+4H); 4.07 (CH_2–arom., d, 1H, $^2J_{HH}$ = 14.8); 4.17 (CH_2–arom., d, 1H, $^2J_{HH}$ = 15.9); 4.39 (CH_2–arom., d, 1H, $^2J_{HH}$ = 14.8); 4.87 (CH_2–arom., d, 1H, $^2J_{HH}$ = 15.9); 5.72 (CH_2–N_3, d, 1H, $^2J_{HH}$ = 15.4); 7.19 (CH_2–N_3, d, 1H, $^2J_{HH}$ = 15.4); 7.83 (C*H*, dd, 1H, $^3J_{HH}$ = 7.9, $^4J_{HH}$ = 1.2); 7.87 (C*H*, dd, 1H, $^3J_{HH}$ = 7.9, $^4J_{HH}$ = 1.2); 7.90 (C*H*–N_3, s, 1H); 8.14 (C*H*, d, 1H, $^4J_{HH}$ = 1.6); 8.26 (C*H*, t, 1H, $^3J_{HH}$ = 7.9); 8.45 (C*H*, d, 1H, $^4J_{HH}$ = 1.6); 8.82 (C*H*, dd, 1H, $^4J_{HH}$ = 1.8, $^4J_{HH}$ = 1.4); 8.92 (C*H*, dd, 1H, $^4J_{HH}$ = 2.2, $^4J_{HH}$ = 1.4); 8.97 (C*H*, dd, 1H, $^4J_{HH}$ = 2.2, $^4J_{HH}$ = 1.8). **ESI-HRMS:** 887.2121 [M]$^+$ (theor. $[C_{34}H_{36}N_{10}O_8Lu_1]^+$ = 887.2120). **UV absorption:** λ_{max} = 266 nm.

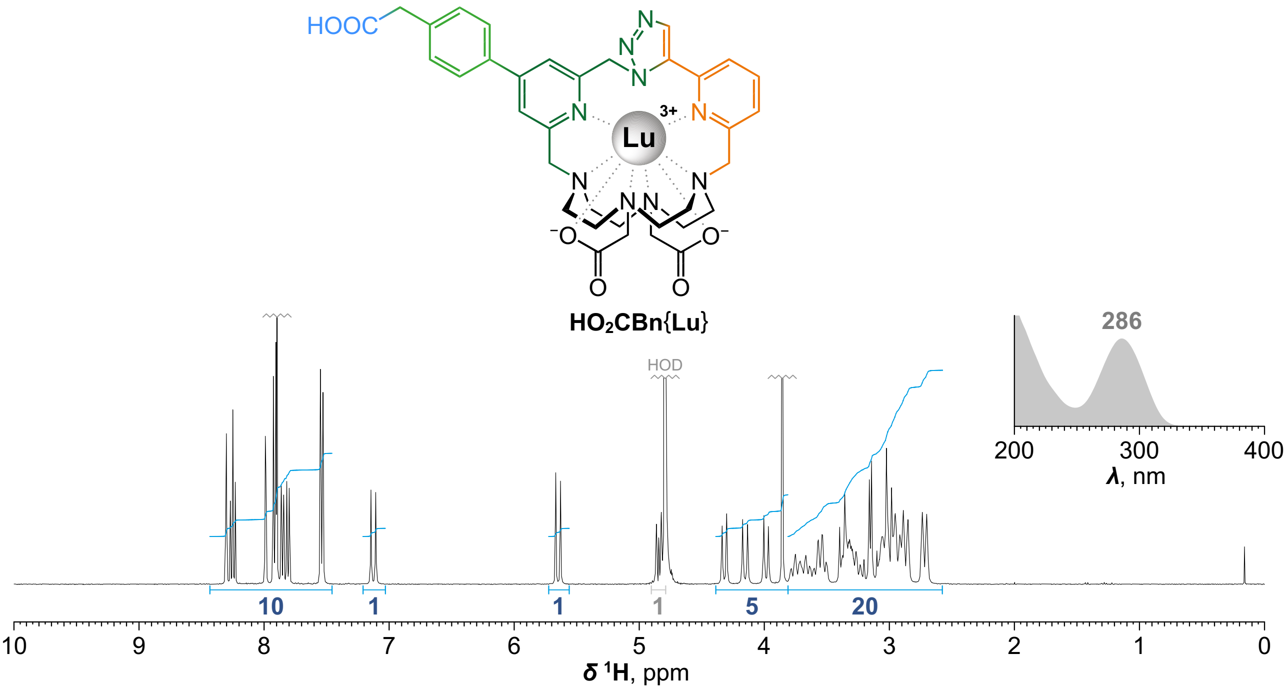

Supplementary Fig. 101. Synthesis and characterization of HO_2CBn{Lu}. Glass vial (4 mL) was charged with [**Cl**{**Lu**}]$^+$[TFA]$^-$·5.1H_2O (15.0 mg; 15.6 µmol; 1.0 equiv.), *4*-(carboxymethyl) phenylboronic acid pinacol ester (8.0 mg; 30.5 µmol; 2.0 equiv.) and XPhos Pd G2 (0.7 mg; ~0.9 µmol; 6 mol%) and was then three times secured with argon. Under constant flow of argon was then added dry DMF (600 µL) through septum followed by addition of freshly prepared (and briefly washed with argon prior addition) aq. solution of K_3PO_4·H_2O (391 mM; 200 µL; 78 µmol; 5.0 equiv.). The mixture was then stirred for 16 h under septum (but without external argon) at 80 °C. Resulting heterogenous mixture was diluted with H_2O (2 mL) and the resulting slightly opalescent pale-yellow solution was filtered through syringe microfilter (RC). Filtrate was then directly purified by preparative HPLC (C18; H_2O–MeCN gradient with TFA additive). Fractions with product were joined and directly lyophilized to give product as white fluffy solid. **Yield:** 7.2 mg (41%; 1 step; based on [**Cl**{**Lu**}]$^+$[TFA]$^-$·5.1H_2O). **NMR (D_2O, pD ~3): ^{1}H** (401.0 MHz) δ_H 2.60–3.82 (CH_2–N, CH_2–CO, m, 16+4H); 3.86 (OC–CH_2–arom., s, 2H); 3.99 (CH_2–arom., d, 1H, $^2J_{HH}$ = 14.7); 4.15 (CH_2–arom., d, 1H, $^2J_{HH}$ = 15.9); 4.32 (CH_2–arom., d, 1H, $^2J_{HH}$ = 14.6); 4.84 (CH_2–arom., d, 1H, $^2J_{HH}$ = 15.9); 5.65 (CH_2–N_3, d, 1H, $^2J_{HH}$ = 15.3); 7.13 (CH_2–N_3, d, 1H, $^2J_{HH}$ = 15.3); 7.54 (C*H*, d, 2H, $^3J_{HH}$ = 8.3); 7.81 (C*H*, dd, 1H, $^3J_{HH}$ = 7.9, $^4J_{HH}$ = 1.2); 7.85 (C*H*, dd, 1H, $^3J_{HH}$ = 7.9, $^4J_{HH}$ = 1.2); 7.90 (C*H*–N_3, s, 1H); 7.92 (C*H*, d, 2H, $^3J_{HH}$ = 8.3); 7.99 (C*H*, d, 1H, $^4J_{HH}$ = 1.6); 8.25 (C*H*, t, 1H, $^3J_{HH}$ = 7.9); 8.30 (C*H*, d, 1H, $^4J_{HH}$ = 1.6). **ESI-HRMS:** 856.2418 [M]$^+$ (theor. [$C_{35}H_{39}N_9O_6Lu_1$]$^+$ = 856.2426). **UV absorption:** λ_{max} = 286 nm. **EA** ([$C_{35}H_{39}N_9O_6Cl_1Lu_1$]$^+$[TFA]$^-$·0.7TFA·4.6$H_2O$, M_R = 1132.4): C 40.7 (40.6); H 4.4 (4.3); N 11.1 (11.3).

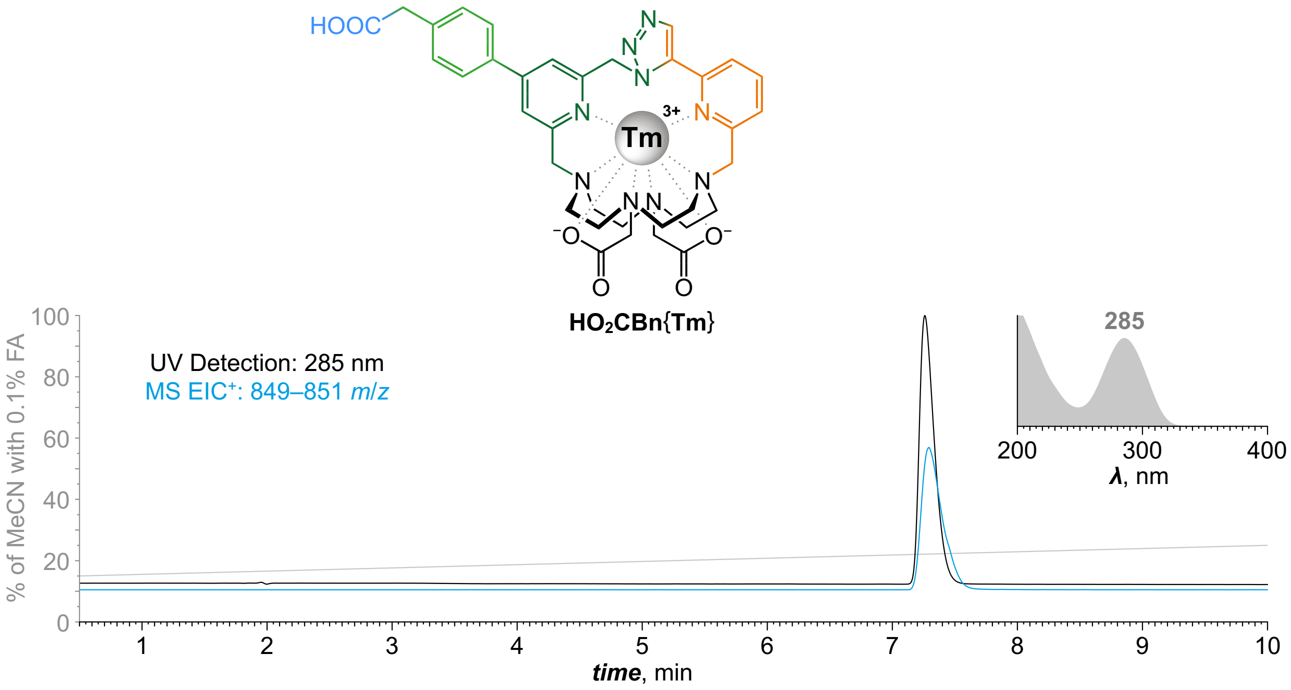

Supplementary Fig. 102. Synthesis and characterization of $HO_2CBn\{Tm\}$. Glass vial (4 mL) was charged with [**Cl**{**Tm**}]$^+$[TFA]$^-$·0.2TFA·2.6H_2O (48.0 mg; 51.4 µmol; 1.0 equiv.), *2*-(*4*-boronophenyl) acetic acid (10.0 mg; 55.6 µmol; 1.1 equiv.) and XPhos Pd G2 (2.0 mg; 2.5 µmol; 5 mol%) and was then three times secured with argon. Under constant flow of argon was then added dry *1*,*4*-dioxane (1.8 mL) through septum followed by addition of freshly prepared (and briefly washed with argon prior addition) aq. solution of $K_3PO_4 \cdot H_2O$ (427 mM; 600 µL; 256 µmol; 5.0 equiv.). The mixture was then stirred for 16 h under septum (but without external argon) at 60 °C. Resulting heterogenous mixture was evaporated to dryness. Residue was re-dissolved in H2O (3 mL), filtered through syringe microfilter (RC) and purified by preparative HPLC (C18; H_2O–MeCN gradient with TFA additive). Fractions with product were joined and directly lyophilized to give product as white fluffy solid. **Yield:** 29.2 mg (53%; 1 step; based on [**Cl**{**Tm**}]$^+$[TFA]$^-$·0.2TFA·2.6H_2O). **ESI-HRMS:** 850.2362 [M]$^+$ (theor. $[C_{35}H_{39}N_9O_6Tm_1]^+$ = 850.2360). **UV absorption:** λ_{max} = 285 nm. **EA** ($[C_{35}H_{39}N_9O_6Cl_1Tm_1]^+[TFA]^-\cdot 0.5TFA \cdot 2.5H_2O$, M_R = 1065.7): C 42.8 (42.6); H 4.2 (3.9); N 11.8 (11.6); F 8.0 (8.0). **X-Ray**: In a glass vial (4 mL), **HO_2CBn**{**Tm**}$^+$[TFA]$^-$·0.5TFA·2.5H_2O (1.0 mg; 0.9 µmol; 1.0 equiv.) was dissolved in H_2O (75 µL) followed by addition of aq. $HClO_4$ (1.0 M; 1.0 µL; 1.0 µmol; 1.1 equiv.). The vial was gently heated and vortexed. The vial was left standing inside of a closed larger glass vial (20 mL) filled with *i*-PrOH, yielding single crystals over time.

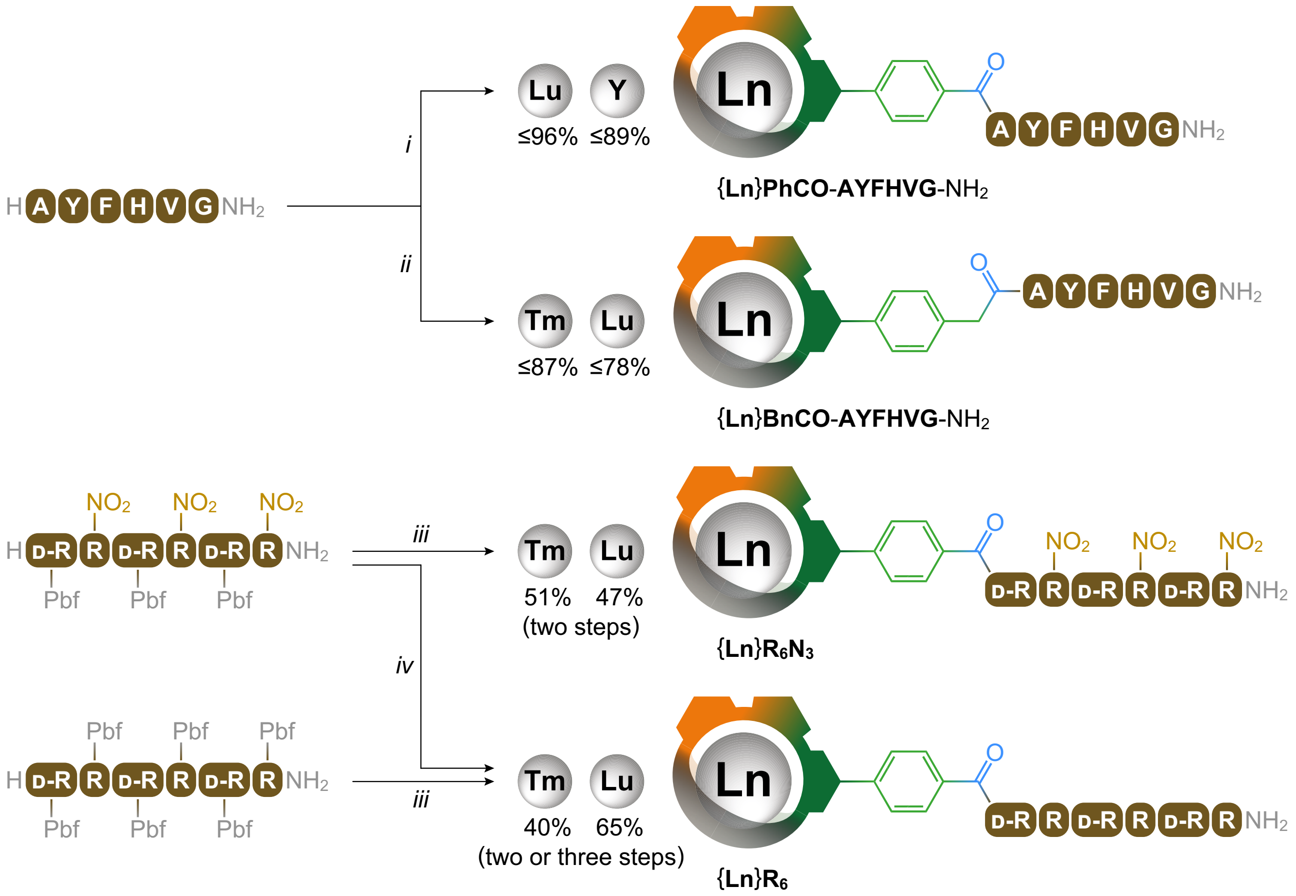

Supplementary Fig. 103. ClickZip hexapeptide conjugates. ClickZips conjugation to peptides was achived using common amide coupling procedure in solution (i.e. no SPPS). While conjugates with H-AYFHVG-NH_2 hexapeptide were synthesized as models for their acid hydrolysis study, the hexaarganine conjugates were designed as cell-penetrating peptides (CPP) with ClickZip tags. No decomplexation was observed during any of these transformations and also, no reduction of pyridine ring was observed during catalytic hydrogenation (as oposed to reduction using $NaBH_4$/MeOH system in the case of **Ph{Lu-red}**; see above). ***Conditions:*** (*i*) **HCO_2CPh{Ln}**, PyAOP, DIPEA, DMSO, RT; (*ii*) **HCO_2CBn{Ln}**, PyAOP, DIPEA, DMSO, RT; (*iii*) **HCO_2CBn{Ln}**, PyAOP, NMM, DMF, DMSO, RT *followed by* TFA, RT; (*iv*) **HCO_2CPh{Ln}**, PyAOP, NMM, DMF, DMSO, RT *followed by* TFA, RT *followed by* H_2, Pd@C, H_2O, RT. Yields refer to isolated compounds.

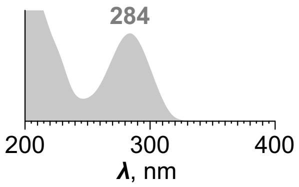

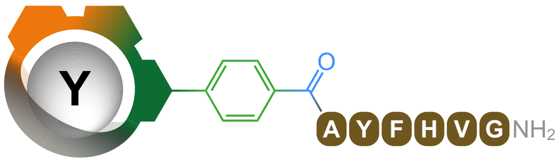

{Y}PhCO-AYFHVG-NH$_2$

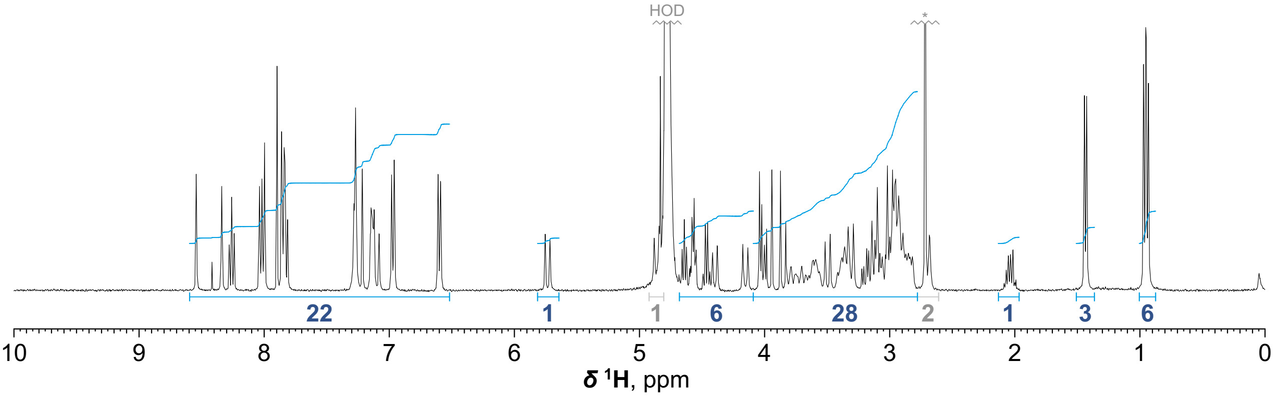

Supplementary Fig. 104. Synthesis and characterization of {Y}PhCO-AYFHVG-NH$_2$. In a glass vial (4 mL), [**HCO$_2$CPh{Y}**]$^+$[TFA]$^-$·0.6TFA·4.8H$_2$O (3.0 mg; 2.9 µmol; 1.0 equiv.) and H-YFHVG-NH$_2$·1.0TFA·xH$_2$O peptide (3.4 mg; ~4.2 µmol; ~1.4 equiv.) were dissolved in DMSO (1 mL) followed by addition of DIPEA (5.0 µL; 29 µmol; ~10 equiv.). Freshly prepared solution of PyOAP (100 mM in DMSO; 52 µL; 5.2 µmol; 1.8 equiv.) was then added and the resulting yellow solution was stirred for 20 min at RT. Mixture was then directly purified by preparative HPLC (C18; H$_2$O–MeCN gradient with TFA additive). Fractions with product were joined and directly lyophilized to give product as white fluffy solid. **Yield:** 4.0 mg (≤89% assuming [**M**]$^+$[TFA]$^-$·xH$_2$O; M_R = 1543; 1 step; based on [**HCO$_2$CPh{Y}**]$^+$[TFA]$^-$·0.6TFA·4.8H$_2$O). **NMR (D$_2$O, pD ~5; asterisk denotes impurity, presumably residual DMSO):** **^{1}H** (401.0 MHz) δ_H 0.93 (^{V}CH_3, d, 3H, $^3J_{HH}$ = 6.9); 0.95 (^{V}CH_3, d, 3H, $^3J_{HH}$ = 6.8); 1.42 (^{A}CH_3, d, 3H, $^3J_{HH}$ = 7.2); 2.03 (^{V}CH–(CH$_3$)$_2$, qqd, 1H, $^3J_{HH}$ = 6.8, $^3J_{HH}$ = 6.7, $^3J_{HH}$ = 6.6); 2.62–3.81 (CH_2–N, CH_2–CO, ^{H}CH_2, ^{F}CH_2, ^{Y}CH_2, m, 16+4+2+2+2H); 3.84 (^{G}CH_2, d, 1H, $^2J_{HH}$ = 17.1); 3.95 (^{G}CH_2, d, 1H, $^2J_{HH}$ = 17.1); 4.01 (CH_2–arom., d, 1H, $^2J_{HH}$ = 14.9); 4.02 (^{V}CH, d, 1H, $^3J_{HH}$ = 6.8); 4.14 (CH_2–arom., d, 1H, $^2J_{HH}$ = 16.2); 4.39 (CH_2–arom., d, 1H, $^2J_{HH}$ = 14.9); 4.45 (^{A}CH, q, 1H, $^3J_{HH}$ = 7.2); 4.52–4.67 (^{H}CH, ^{F}CH, ^{Y}CH, m, 1+1+1H); 4.85 (CH_2–arom., d, 1H, $^2J_{HH}$ = 16.2); 5.72 (CH_2–N$_3$, d, 1H, $^2J_{HH}$ = 15.5); 6.59 (^{Y}CH, d, 2H, $^3J_{HH}$ = 8.0); 6.96 (^{Y}CH, d, 2H, $^3J_{HH}$ = 8.0); 7.09 (CH_2–N$_3$, d, 1H, $^2J_{HH}$ = 15.5); 7.10–7.16 (^{F}CH, m, 2H); 7.21 (^{H}CH, s, 1H); 7.24–7.30 (^{F}CH, m, 3H); 7.78–7.86 (CH, m, 4H); 7.89 (CH–N$_3$, s, 1H); 8.00 (CH, d, 2H, $^3J_{HH}$ = 8.3); 8.03 (CH, d, 1H, $^4J_{HH}$ = 1.6); 8.25 (CH, t, 1H, $^3J_{HH}$ = 7.9); 8.33 (CH, d, 1H, $^4J_{HH}$ = 1.5); 8.53 (^{H}CH, t, 1H, $^4J_{HH}$ = 1.5). **ESI-HRMS:** 1429.5264 [M]$^+$ (theor. [C$_{68}$H$_{80}$N$_{18}$O$_{12}$Y$_1$]$^+$ = 1429.5256). **UV absorption:** λ_{max} = 284 nm.

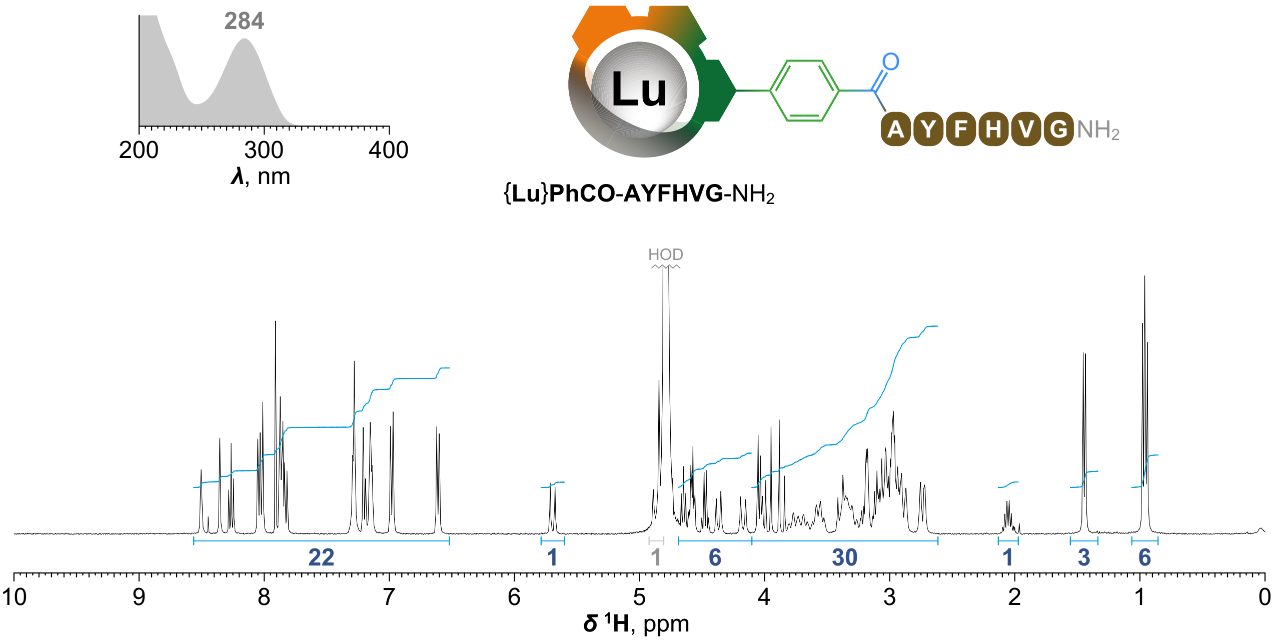

Supplementary Fig. 105. Synthesis and characterization of {Lu}BnCO-AYFHVG-NH$_2$. In a glass vial (4 mL), [**HCO$_2$CPh{Lu}**]$^+$[TFA]$^-$·1.4TFA·3.2H$_2$O (3.3 mg; 2.8 µmol; 1.0 equiv.) and H-YFHVG-NH$_2$·1.0TFA·xH$_2$O (3.4 mg; ~4.2 µmol; ~1.5 equiv.) were dissolved in DMSO (1 mL) followed by addition of DIPEA (5.0 µL; 29 µmol; ~10 equiv.). Freshly prepared solution of PyOAP (100 mM in DMSO; 52 µL; 5.2 µmol; 1.9 equiv.) was then added and the resulting yellow solution was stirred for 20 min at RT. Mixture was then directly purified by preparative HPLC (C18; H$_2$O–MeCN gradient with TFA additive). Fractions with product were joined and directly lyophilized to give product as white fluffy solid. **Yield:** 4.4 mg (≤96% assuming [**M**]$^+$[TFA]$^-$·xH$_2$O; M_R = 1630; 1 step; based on [**HCO$_2$CPh{Lu}**]$^+$[TFA]$^-$·1.4TFA·3.2H$_2$O). **NMR (D$_2$O, pD ~5): ^{1}H** (401.0 MHz) δ_H 0.93 (^{V}CH_3, d, 3H, $^3J_{HH}$ = 6.7); 0.95 (^{V}CH_3, d, 3H, $^3J_{HH}$ = 6.8); 1.42 (^{A}CH_3, d, 3H, $^3J_{HH}$ = 7.2); 2.03 (^{V}CH–(CH$_3$)$_2$, qqd, 1H, $^3J_{HH}$ = 6.8, $^3J_{HH}$ = 6.7, $^3J_{HH}$ = 6.6); 2.62–3.81 (CH_2–N, CH_2–CO, ^{H}CH_2, ^{F}CH_2, ^{Y}CH_2, m, 16+4+2+2+2H); 3.84 (^{G}CH_2, d, 1H, $^2J_{HH}$ = 17.1); 3.95 (^{G}CH_2, d, 1H, $^2J_{HH}$ = 17.1); 4.01 (CH_2–arom., d, 1H, $^2J_{HH}$ = 14.7); 4.02 (^{V}CH, d, 1H, $^3J_{HH}$ = 6.6); 4.15 (CH_2–arom., d, 1H, $^2J_{HH}$ = 15.9); 4.35 (CH_2–arom., d, 1H, $^2J_{HH}$ = 14.7); 4.45 (^{A}CH, q, 1H, $^3J_{HH}$ = 7.2); 4.52–4.66 (^{H}CH, ^{F}CH, ^{Y}CH, m, 1+1+1H); 4.85 (CH_2–arom., d, 1H, $^2J_{HH}$ = 15.9); 5.67 (CH_2–N$_3$, d, 1H, $^2J_{HH}$ = 15.3); 6.59 (^{Y}CH, d, 2H, $^3J_{HH}$ = 8.3); 6.96 (^{Y}CH, d, 2H, $^3J_{HH}$ = 8.3); 7.08–7.18 (CH_2–N$_3$, ^{F}CH, m, 1+2H); 7.19 (^{H}CH, s, 1H); 7.23–7.30 (^{F}CH, m, 3H); 7.81 (CH, dd, 1H, $^3J_{HH}$ = 7.9; $^4J_{HH}$ = 1.2); 7.82–7.87 (CH, m, 3H); 7.89 (CH–N$_3$, s, 1H); 8.00 (CH, d, 2H, $^3J_{HH}$ = 8.1); 8.03 (CH, d, 1H, $^4J_{HH}$ = 1.6); 8.24 (CH, t, 1H, $^3J_{HH}$ = 7.9); 8.33 (CH, d, 1H, $^4J_{HH}$ = 1.6); 8.48 (^{H}CH, bs, 1H). **ESI-HRMS:** 1515.5593 [M]$^+$ (theor. [C$_{68}$H$_{80}$N$_{18}$O$_{12}$Lu$_1$]$^+$ = 1515.5605). **UV absorption:** λ_{max} = 284 nm.

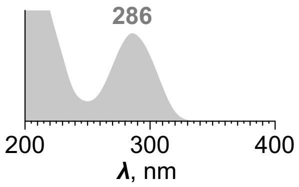

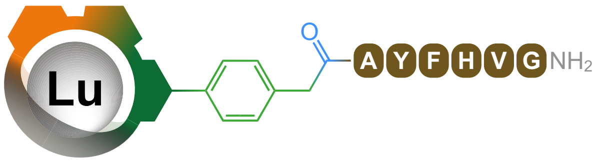

{Lu}BnCO-AYFHVG-NH2

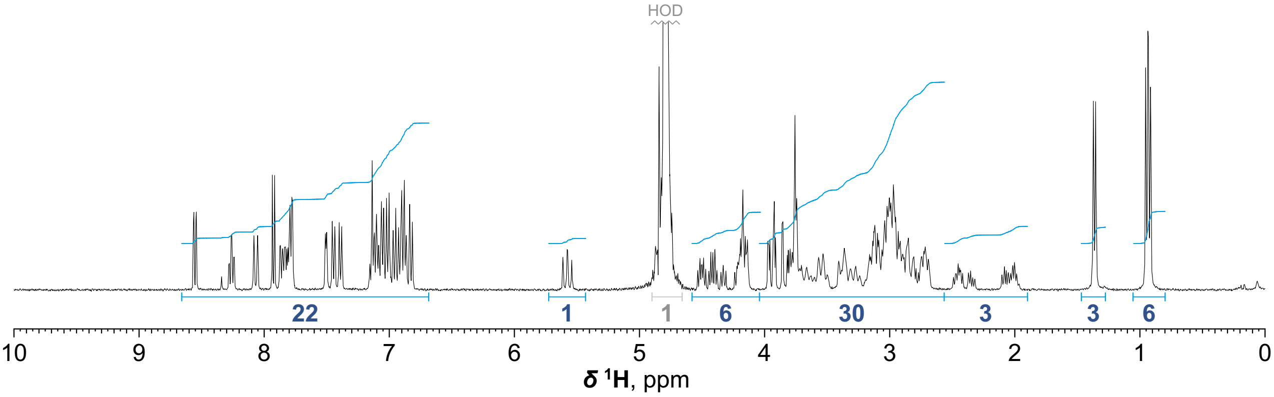

Supplementary Fig. 106. Synthesis and characterization of {Lu}BnCO-AYFHVG-NH$_2$. In a glass vial (4 mL), [**HCO$_2$CBn{Lu}**]$^+$[TFA]$^-$·0.7TFA·4.6H$_2$O (1.6 mg; 1.4 µmol; 1.0 equiv.) and H-YFHVG-NH$_2$·1.0TFA·xH$_2$O (1.6 mg; ~2.0 µmol; ~1.4 equiv.) were dissolved in DMSO (500 µL) followed by addition of DIPEA (2.3 µL; 13 µmol; 9.4 equiv.). Freshly prepared solution of PyOAP (100 mM in DMSO; 25 µL; 2.5 µmol; 1.8 equiv.) was then added and the resulting yellow solution was stirred for 40 min at RT. Mixture was then directly purified by preparative HPLC (C18; H$_2$O–MeCN gradient with TFA additive). Fractions with product were joined and directly lyophilized to give product as white fluffy solid. **Yield:** 1.8 mg (≤78% assuming [**M**]$^+$[TFA]$^-$·xH$_2$O; M_R = 1644; 1 step; based on [**HCO$_2$CBn{Lu}**]$^+$[TFA]$^-$·0.7TFA·4.6H$_2$O). **NMR (D$_2$O, pD ~4; assignment was complicated by two distinguishable diastereomeric set of most of the signals): ^{1}H** (401.0 MHz) δ_H 0.91 (^{V}C*H*$_3$, d, 3H, $^3J_{HH}$ = 6.8); 0.93 (^{V}C*H*$_3$, d, 3H, $^3J_{HH}$ = 6.7); 1.36 (^{A}C*H*$_3$, d, 3H, $^3J_{HH}$ = 7.2); 1.90–4.21 (^{V}C*H*–(CH$_3$)$_2$, C*H*$_2$–N, C*H*$_2$–CO, ^{H}C*H*$_2$, ^{F}C*H*$_2$, ^{Y}C*H*$_2$, ^{G}C*H*$_2$, C*H*$_2$–arom., ^{V}C*H*, ^{A}C*H*, m, 1+16+4+2+2+2+2+5+1+1H); 4.21–4.56 (^{H}C*H*, ^{F}C*H*, ^{Y}C*H*, m, 1+1+1H); 4.81–4.90 (C*H*$_2$–arom., m, 1H); 5.52–5.62 (C*H*$_2$–N$_3$, m, 1H); 6.77–7.18 (^{Y}C*H*, C*H*$_2$–N$_3$, ^{F}C*H*, ^{H}C*H*, ^{F}C*H*, m, 4+1+2+1+3H); 7.34–8.10 (C*H*, C*H*–N$_3$, 8+1H); 8.21–8.30 (C*H*, m, 1H); 8.52–8.58 (^{H}C*H*, m, 1H). **ESI-HRMS:** 765.2919 [M+H]$^{2+}$ (theor. [C$_{69}$H$_{83}$N$_{18}$O$_{12}$Lu$_1$]$^{2+}$ = 765.2917). **UV absorption:** λ_{max} = 286 nm.

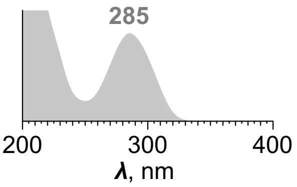

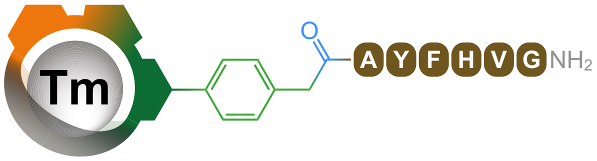

{Tm}BnCO-AYFHVG-NH$_2$

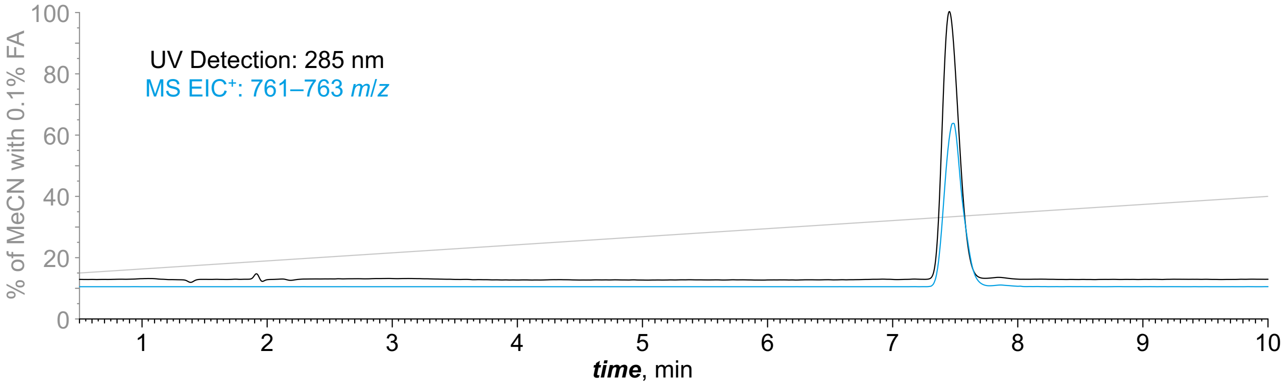

Supplementary Fig. 107. Synthesis and characterization of {Tm}BnCO-AYFHVG-NH$_2$. In a glass vial (4 mL), [**HCO$_2$CBn{Tm}**]$^+$[TFA]$^-$·0.5TFA·2.5H$_2$O (1.5 mg; 1.4 µmol; 1.0 equiv.) and H-YFHVG-NH$_2$·1.0TFA·xH$_2$O (1.6 mg; ~2.0 µmol; ~1.4 equiv.) were dissolved in DMSO (500 µL) followed by addition of DIPEA (2.2 µL; 13 µmol; 9.0 equiv.). Freshly prepared solution of PyOAP (100 mM in DMSO; 24 µL; 2.4 µmol; 1.7 equiv.) was then added and the resulting yellow solution was stirred for 40 min at RT. Mixture was then directly purified by preparative HPLC (C18; H$_2$O–MeCN gradient with TFA additive). Fractions with product were joined and directly lyophilized to give product as white fluffy solid. **Yield:** 2.0 mg (≤87% assuming [**M**]$^+$[TFA]$^-$·xH$_2$O; M_R = 1638; 1 step; based on [**HCO$_2$CBn{Tm}**]$^+$[TFA]$^-$·0.2TFA·7.0H$_2$O). **ESI-HRMS:** 762.2885 [M+H]$^{2+}$ (theor. [$C_{69}H_{83}N_{18}O_{12}Tm_1$]$^{2+}$ = 762.2887). **UV absorption:** λ_{max} = 285 nm.

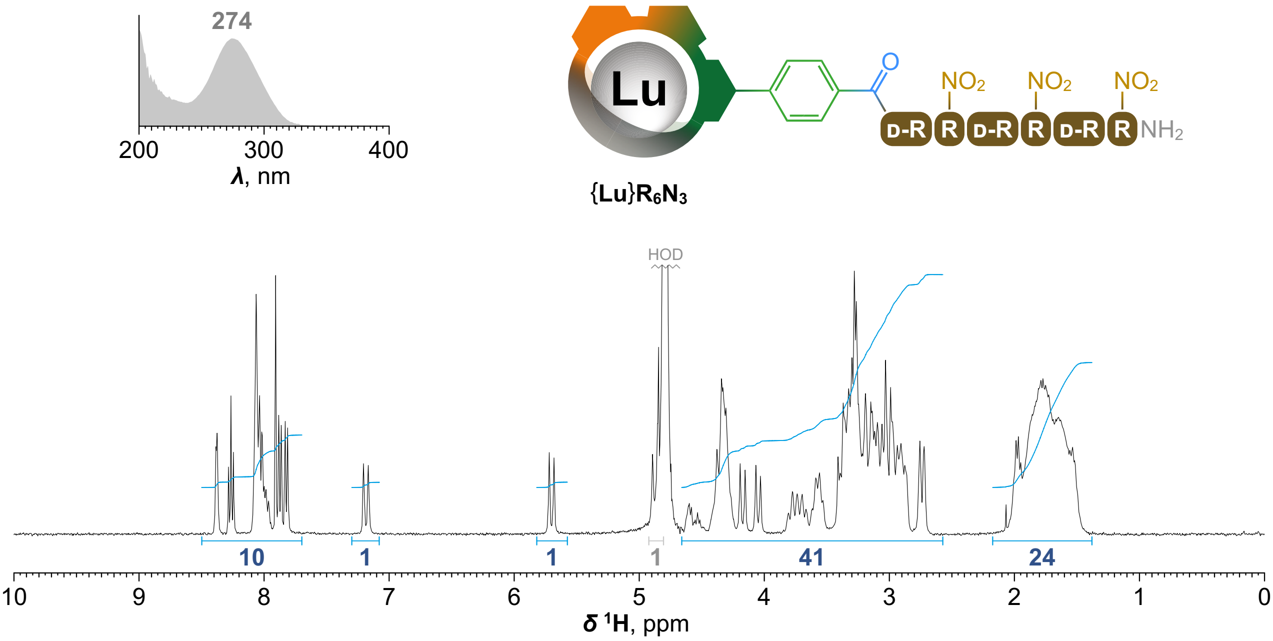

Supplementary Fig. 108. Synthesis and characterization of {Lu}R_6N_3. In a glass vial (4 mL), [**HCO_2CPh{Lu**}]$^+$[TFA]$^-$·1.4TFA·3.2H_2O (15.0 mg; 12.8 µmol; 1.0 equiv.) and H-[D-RR(NO_2)]$_3$-NH_2·3.2TFA·5.3H_2O (31 mg; 13.4 µmol; 1.0 equiv.) were dissolved in DMSO (1.5 mL) followed by addition of solution of NMM (400 mM, 340 µL; 136 µmol; 11 equiv.) in DMF and freshly prepared solution of PyOAP (110 mM; 210 µL; 23.1 µmol; 1.8 equiv.) in DMSO and the resulting yellow solution was stirred for 30 min at RT. Mixture was then directly purified by preparative HPLC (C18; H_2O–MeCN gradient with TFA additive). Fractions with Pbf protected product were joined, evaporated to dryness and twice co-evaporated with MeOH. Residue was dissolved in TFA (3 mL) and the resulting clear mixture was stirred 16 h at RT. Mixture was evaporated to dryness and the residue was suspended in 20 % MeCN (3 mL). Mixture was filtered through syringe microfilter (RC) and the filtrate was directly purified by preparative HPLC (C18; H_2O–MeCN gradient with TFA additive). Fractions with product were joined and directly lyophilized to give product in the form of trifluoroacetate salt as white fluffy solid. **Yield:** 18.4 mg (47%; 2 steps; based on [**HCO_2CPh{Lu**}]$^+$[TFA]$^-$·1.4TFA·3.2H_2O). **NMR (D_2O, pD ~5): ^{1}H** (401.0 MHz) δ_H 1.39–2.07 (^{R}CH_2, m, 24H); 2.63–3.85 (CH_2–N, CH_2–CO, ^{R}CH_2, m, 16+4+12H); 4.04 (CH_2–arom., d, 1H, $^2J_{HH}$ = 14.8); 4.16 (CH_2–arom., d, 1H, $^2J_{HH}$ = 16); 4.21–4.66 (^{R}C*H*, CH_2–arom., m, 6+1H); 4.86 (CH_2–arom., d, 1H, $^2J_{HH}$ = 15.9); 5.69 (CH_2–N_3, d, 1H, $^2J_{HH}$ = 15.3); 7.18 (CH_2–N_3, d, 1H, $^2J_{HH}$ = 15.3); 7.81 (C*H*, dd, 1H, $^3J_{HH}$ = 7.9, $^4J_{HH}$ = 1.2); 7.86 (C*H*, dd, 1H, $^3J_{HH}$ = 7.9, $^4J_{HH}$ = 1.2); 7.90 (C*H*–N_3, s, 1H); 7.95–8.09 (C*H*, m, 5H); 8.25 (C*H*, t, 1H, $^3J_{HH}$ = 7.9); 8.37 (C*H*, d, 1H, $^4J_{HH}$ = 1.6). **MALDI-HRMS**: 1912.8054 [M]$^+$ (theor. [$C_{70}H_{107}N_{37}O_{17}Lu_1$]$^+$ = 1912.8048). **UV absorption:** λ_{max} = 274 nm. **EA** ([$C_{70}H_{107}N_{37}O_{17}Lu_1$]$^+$[TFA]$^-$·3.3TFA·8.8$H_2O$, M_R = 2562): C 36.9 (37.0); H 5.0 (4.8); N 20.2 (20.0); F 9.6 (9.3).

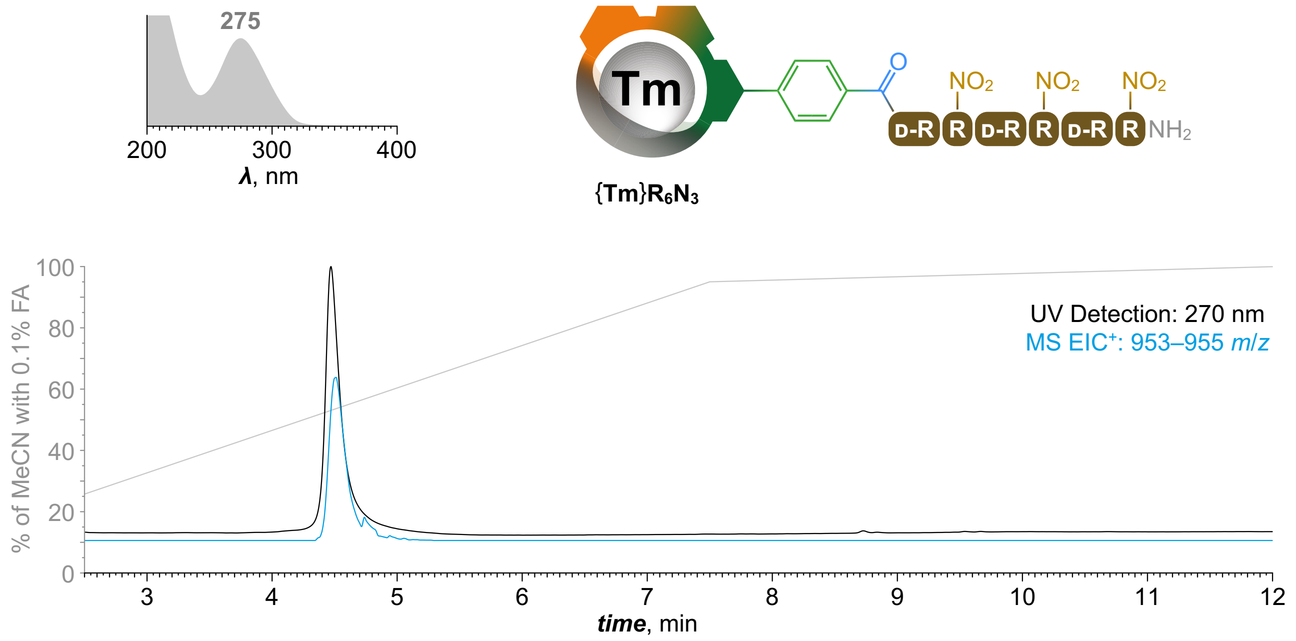

Supplementary Fig. 109. Synthesis and characterization of {Tm}R_6N_3. In a glass vial (4 mL), [**HCO_2CPh{Tm}**]$^+$[TFA]$^-$·1.1TFA·2.6H_2O (10.0 mg; 8.9 µmol; 1.0 equiv.) and H-[D-RR(NO_2)]$_3$-NH_2·3.2TFA·5.3H_2O (21 mg; 9.1 µmol; 1.0 equiv.) were dissolved in DMSO (1.0 mL) followed by addition of solution of NMM (400 mM, 230 µL; 92 µmol; 10 equiv.) in DMF and freshly prepared solution of PyOAP (110 mM; 140 µL; 15.4 µmol; 1.7 equiv.) in DMSO and the resulting yellow solution was stirred for 30 min at RT. Mixture was then directly purified by preparative HPLC (C18; H_2O–MeCN gradient with TFA additive). Fractions with Pbf protected product were joined, evaporated to dryness and twice co-evaporated with MeOH. Residue was dissolved in TFA (3 mL) and the resulting clear mixture was stirred for 16 h at RT. Mixture was evaporated to dryness and the residue was suspended in 20 % MeCN (3 mL). Mixture was filtered through syringe microfilter (RC) and the filtrate was directly purified by preparative HPLC (C18; H_2O–MeCN gradient with TFA additive). Fractions with product were joined and directly lyophilized to give product in the form of trifluoroacetate salt as white fluffy solid. **Yield:** 11.6 mg (51%; 2 steps; based on [**HCO_2CPh{Tm}**]$^+$[TFA]$^-$·1.1TFA·2.6H_2O). **MALDI-HRMS**: 1906.7973 [M]$^+$ (theor. [$C_{70}H_{107}N_{37}O_{17}Tm_1$]$^+$ = 1906.7982). **UV absorption:** λ_{max} = 275 nm. **EA** ([$C_{70}H_{107}N_{37}O_{17}Tm_1$]$^+$[TFA]$^-$·3.2TFA·7.9$H_2O$, M_R = 2528): C 37.3 (37.5); H 5.0 (5.0); N 20.5 (20.2); F 9.5 (9.3).

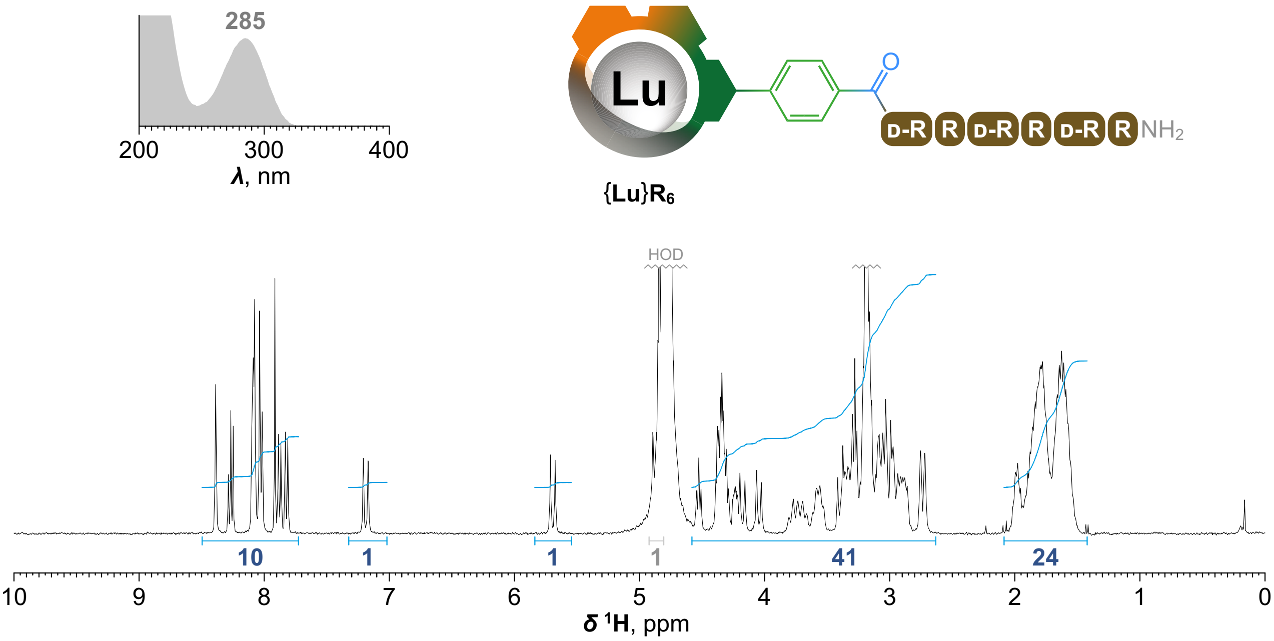

Supplementary Fig. 110. Synthesis and characterization of {Lu}R_6. In a glass vial (4 mL), [**HCO$_2$CPh**{**Lu**}]$^+$[TFA]$^-$·1.4TFA·3.2H_2O (7.1 mg; 6.1 μmol; 1.0 equiv.) and H-[D-R(Pbf)R(Pbf)]$_3$-NH_2·1.0TFA·$x$$H_2O$ (21 mg; ~8.1 μmol; ~1.3 equiv.) were dissolved in DMSO (1 mL) followed by addition of NMM (20 μL; 182 μmol; 30 equiv.) and PyOAP (15.5 mg; 29.7 μmol; 5.0 equiv.) and the resulting yellow solution was stirred for 20 min at RT. Mixture was then directly purified by preparative HPLC (C18; H_2O–MeCN gradient with TFA additive). Fractions with Pbf protected product were joined, evaporated to dryness and twice co-evaporated with MeOH. Residue was dissolved in TFA (3 mL) and the resulting clear mixture was stirred for 16 h at RT. Mixture was evaporated to dryness and the residue was purified by preparative HPLC (C18; H_2O–MeCN gradient with TFA additive). Fractions with product were joined and directly lyophilized to give product in the form of trifluoroacetate salt as white fluffy solid. **Yield:** 13.0 mg (65%; 2 steps; based on [**HCO$_2$CPh**{**Lu**}]$^+$[TFA]$^-$·1.4TFA·3.2H_2O). **NMR (D_2O, pD ~3):** ^{1}**H** (401.0 MHz) δ_H 1.41–2.07 (^{R}CH_2, m, 24H); 2.63–3.88 (CH_2–N, CH_2–CO, ^{R}CH_2, m, 16+4+12H); 4.05 (CH_2–arom., d, 1H, $^2J_{HH}$ = 14.9); 4.18 (CH_2–arom., d, 1H, $^2J_{HH}$ = 15.9); 4.21–4.57 (^{R}CH, CH_2–arom., m, 5+1H); 4.53 (^{R}CH, t, 1H, $^3J_{HH}$ = 7.3); 4.87 (CH_2–arom., d, 1H, $^2J_{HH}$ = 15.9); 5.69 (CH_2–N_3, d, 1H, $^2J_{HH}$ = 15.3); 7.19 (CH_2–N_3, d, 1H, $^2J_{HH}$ = 15.3); 7.82 (CH, dd, 1H, $^3J_{HH}$ = 7.9, $^4J_{HH}$ = 1.2); 7.88 (CH, dd, 1H, $^3J_{HH}$ = 7.9, $^4J_{HH}$ = 1.2); 7.91 (CH–N_3, s, 1H); 8.03 (CH, d, 2H, $^3J_{HH}$ = 8.3); 8.06–8.12 (CH, m, 3H); 8.27 (CH, t, 1H, $^3J_{HH}$ = 7.9); 8.39 (CH, d, 1H, $^4J_{HH}$ = 1.7). **MALDI-HRMS**: 1777.8487 [M]$^+$ (theor. [$C_{70}H_{110}N_{34}O_{11}Lu_1$]$^+$ = 1777.8496). **UV absorption:** λ_{max} = 285 nm. **EA** ([$C_{70}H_{110}N_{34}O_{11}Lu_1$]$^+$[TFA]$^-$·6.7TFA·4.5$H_2O$, M_R = 2737): C 37.5 (37.2); H 4.6 (4.4); N 17.4 (17.2); F 16.0 (15.9).

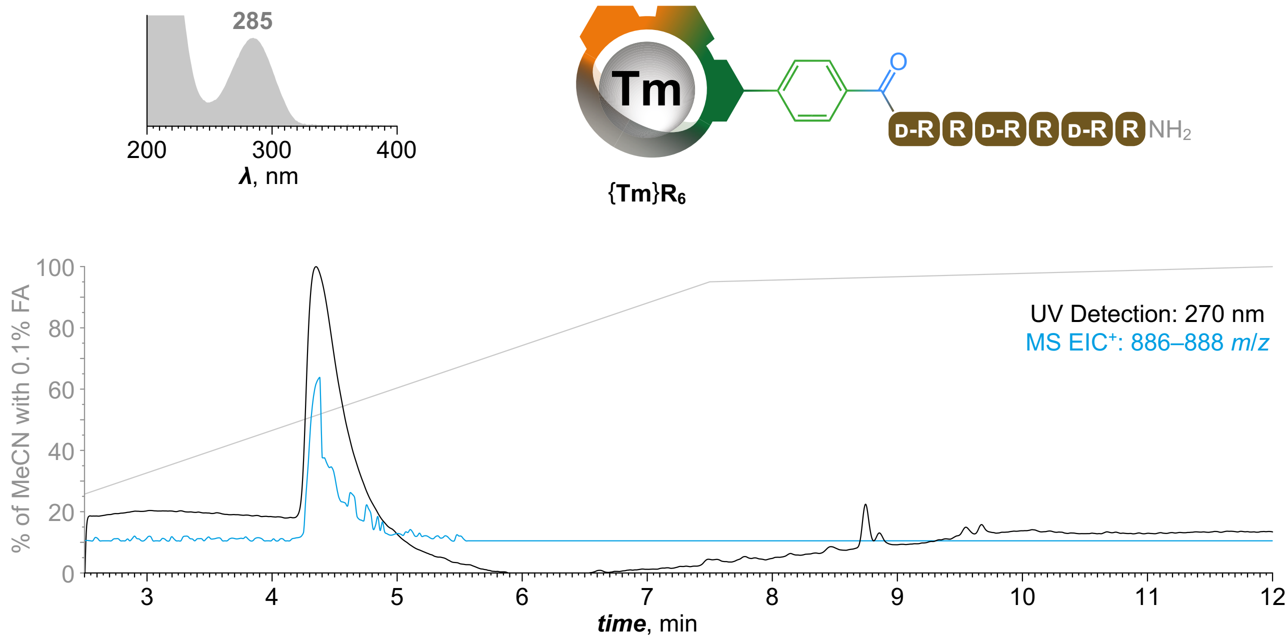

Supplementary Fig. 111. Synthesis and characterization of {Tm}R_6. In a glass vial (4 mL), [**HCO$_2$CPh{Tm}**]$^+$[TFA]$^-$·1.1TFA·2.6H_2O (15.0 mg; 13.4 µmol; 1.0 equiv.) and H-[D-R(Pbf)R(NO_2)]$_3$-NH_2·1.0TFA·$x$$H_2O$·3.2TFA·5.3$H_2O$ (30.9 mg; 13.4 µmol; 1.0 equiv.) were dissolved in DMSO (1.5 mL) followed by addition of solution of NMM (400 mM, 335 µL; 134 µmol; ~10 equiv.) in DMF and freshly prepared solution of PyOAP (110 mM; 210 µL; 23.1 µmol; 1.7 equiv.) in DMSO and the resulting yellow solution was stirred for 30 min at RT. Mixture was then directly purified by preparative HPLC (C18; H_2O–MeCN gradient with TFA additive). Fractions with fully protected product were joined, evaporated to dryness and twice co-evaporated with MeOH. Residue was dissolved in TFA (3 mL) and stirred overnight at RT to remove Pbf groups. Resulting blue solution was evaporated to dryness and the residue was purified by preparative HPLC (C18; H_2O–MeCN gradient with TFA additive). Fractions with partly protected product were joined, and evaporated to dryness. Residue was re dissolved in H_2O (10 mL) and transferred via syringe through septum to a glass flask (25 mL) already filled with Pd/C (30 mg) and argon secured. Hydrogen gas was then left bubbling through the suspension for 2 d at RT. Mixture was then filtered through syringe microfilter (RC), and the filtrate was evaporated to dryness. Residue was purified by preparative HPLC (C18; H_2O–MeCN gradient with TFA additive). Fractions with product were joined and directly lyophilized to give product in the form of trifluoroacetate salt as white fluffy solid. **Yield:** 14.6 mg (40%; 3 steps; based on [**HCO$_2$CPh{Tm}**]$^+$[TFA]$^-$·1.1TFA·2.6H_2O). **MALDI-HRMS**: 1771.8412 [M]$^+$ (theor. [$C_{70}H_{83}N_{34}O_{11}Tm_1$]$^+$ = 1771.8435). **UV absorption:** λ_{max} = 285 nm. **EA** ([$C_{70}H_{110}N_{34}O_{11}Tm_1$]$^+$[TFA]$^-$·7.1TFA·3.3$H_2O$, M_R = 2755): C 37.6 (37.2); H 4.5 (4.3); N 17.3 (17.8); F 16.8 (17.2).

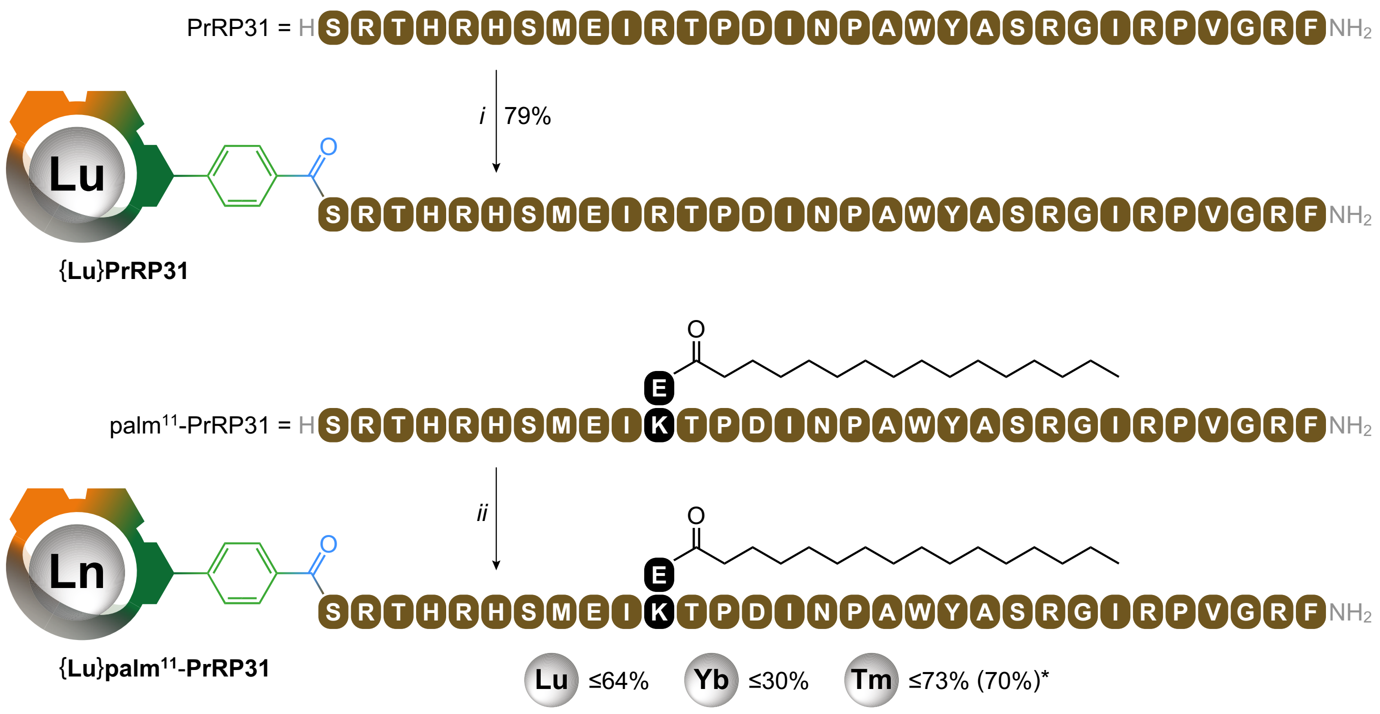

Supplementary Fig. 112. Anti-obesity ClickZip conjugates. ClickZips conjugation to anti-obesity peptides (natural PrRP31 and its lipidized analogue palm[11]-PrRP31) was achived using preactivation of ClickZip carboxylic acid followed by reaction with the *N*-terminus of the particular peptide. Coupling was performed with entirely unprotected forms of the peptides in solution (i.e. no SPPS). Conjugate {**Tm**}**palm[11]-PrRP31** was synthesized twice – each time from different batches of palm[11]-PrRP31. Elemental analysis was performed for one of the batches (denoted by asterisk) – $[C_{215}H_{324}N_{64}O_{51}S_{1}Tm_{1}]^{+}[TFA]^{-}\cdot 7.0TFA\cdot 15.5H_2O$ (M_R = 6013): C 46.1 (45.9); H 6.1 (5.7); N 14.9 (14.6); F 7.6 (7.3). ***Conditions:*** (*i*) $\mathbf{HCO_2CPh}${**Lu**}, PyAOP, NMM, DMSO, DMF, RT; (*ii*) $\mathbf{HCO_2CPh}${**Ln**}, PyAOP, NMM, DMSO, DMF, RT. Yields refer to isolated compounds.

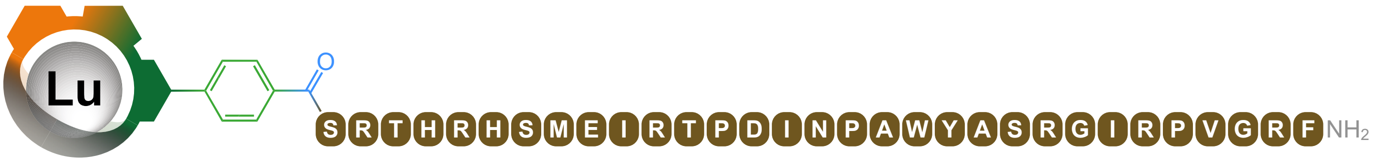

{Lu}PrRP31

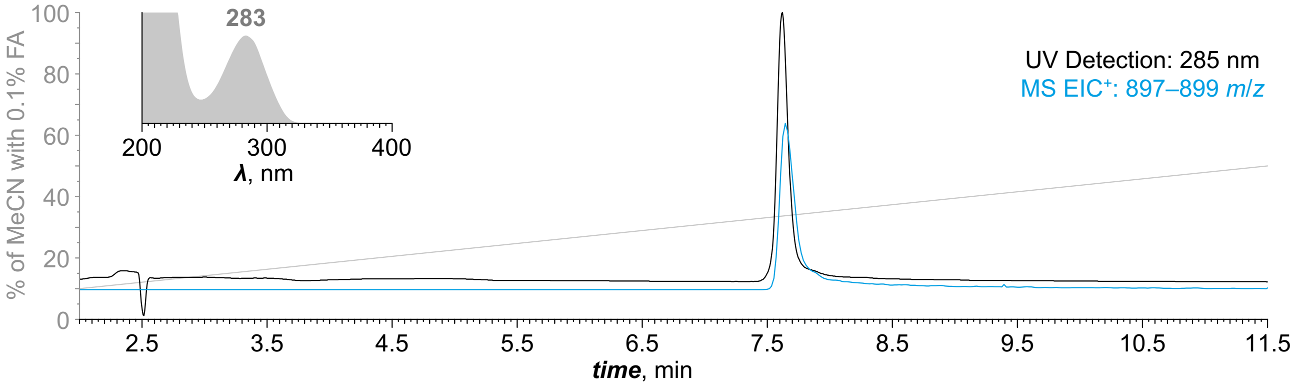

Supplementary Fig. 113. Synthesis and characterization of {Lu}PrRP31. In a plastic Eppendorf test tube (2 mL), [**HCO_2CPh{Lu}**]$^+$[TFA]$^-$·1.4TFA·3.2H_2O (14.1 mg; 12.0 µmol; 1.5 equiv.) was dissolved in DMSO (400 µL) followed by addition of freshly prepared solution of PyOAP (200 mM; 60 µL; 12.0 µmol; 1.5 equiv.) in DMSO and solution of NMM (400 mM, 300 µL; 120 µmol; 15 equiv.) in DMF. The tube was vortexed for 30 seconds and the resulting pale-yellow solution was then quickly added to a stirred solution (in a separate 4 mL glass vial) of PrRP31·10.3TFA·9.2H_2O peptide (40.0 mg; 8.0 µmol; 1.0 equiv.) in DMSO (400 µL). The resulting yellow solution was further stirred for 20 min at RT. Reaction was quenched by addition of neat TFA (18.4 µL; 240 µmol; 30 equiv.) and further diluted with H_2O (2 mL) and 25% MeOH (350 µL). Resulting solution was directly purified by preparative HPLC (C18; H_2O–MeCN gradient with TFA additive). Fractions with pure product were joined and directly lyophilized. The resulting white foam was re-dissolved in H_2O (8 mL) and re-lyophilized to give product in the form of trifluoroacetate salt as white fluffy solid. **Yield:** 37.7 mg (79%; 1 step; based on PrRP31·10.3TFA·9.2H_2O). **Recovery**: 4.4 mg of [**HCO_2CPh{Lu}**]$^+$[TFA]$^-$ (31% of the initial amount used assuming same TFA and water content). **ESI-HRMS**: 898.0306 $[M+4H]^{5+}$ (theor. $[C_{194}H_{291}N_{65}O_{47}S_1Lu_1]^{5+}$ = 898.0296). **UV absorption:** λ_{max} = 283 nm. **EA** ($[C_{194}H_{287}N_{65}O_{47}S_1Lu_1]^+[TFA]^-$·9.5TFA·14.1$H_2O$, M_R = 5939): C 43.5 (43.8); H 5.5 (5.3); N 15.3 (15.1); F 10.1 (9.8).

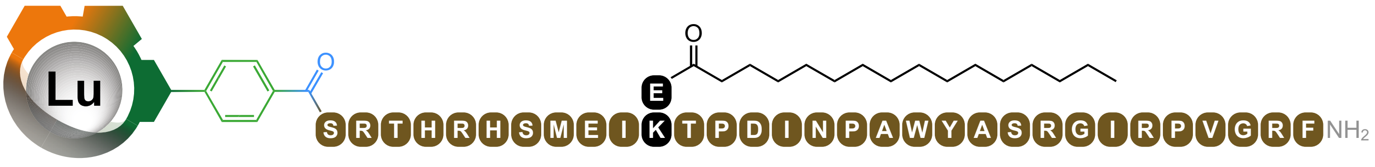

{Lu}palm[11]-PrRP31

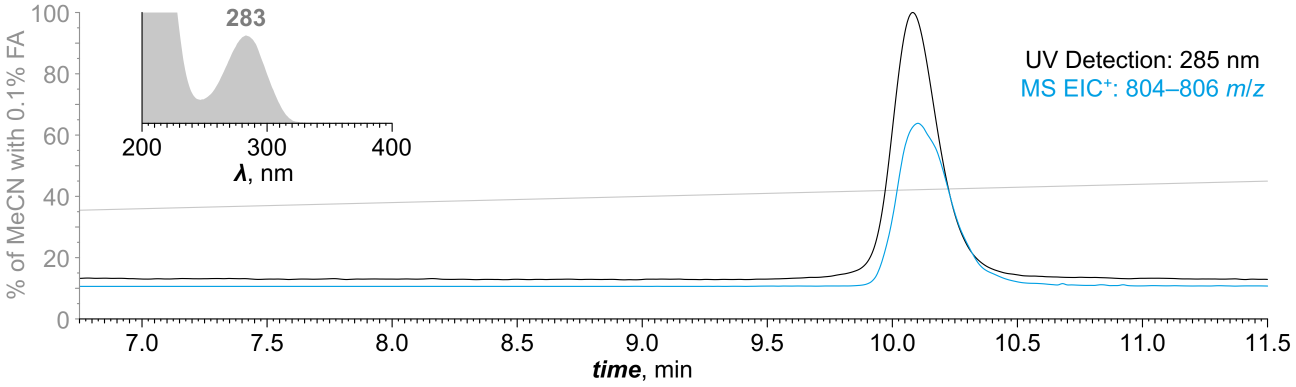

Supplementary Fig. 114. Synthesis and characterization of {Lu}palm^{11}-PrRP31. In a plastic Eppendorf test tube (2 mL), [**HCO_2CPh{Lu}**]$^+$[TFA]$^-$·1.4TFA·3.2H_2O (30 mM in DMSO; 218 µL, 6.6 µmol; 1.5 equiv.) was diluted with freshly prepared solution of PyOAP (200 mM in DMSO; 33 µL; 6.6 µmol; 1.5 equiv.) followed by addition of NMM (400 mM in DMF; 218 µL; 87.2 µmol; 20 equiv.). The tube was vortexed for 30 seconds and the resulting pale-yellow solution was then quickly added to a stirred solution (in a separate 4 mL glass vial) of palm^{11}-PrRP31·7.8TFA·12.4H_2O peptide (20 mM in DMSO; 218 µL; 4.4 µmol; 1.0 equiv.). The resulting yellow solution was quickly briefly vortexed and then further stirred for 30 min at RT. Reaction was quenched by addition of neat TFA (6.7 µL; 87.6 µmol; 20 equiv.) and further diluted with H_2O (2 mL) and 25% MeOH (500 µL). Resulting solution was directly purified by preparative HPLC (C18; H_2O–MeCN gradient with TFA additive). Fractions with pure product were joined and directly lyophilized. The resulting white foam was re-dissolved in H_2O (3 mL) and re-purified by preparative HPLC (C18; H_2O–MeCN gradient with TFA additive). Fractions with pure product were joined and directly lyophilized to give product in the form of trifluoroacetate salt as white fluffy solid. **Yield:** 16.0 mg (≤64% assuming [**M**]$^+$[TFA]$^-$·7.0TFA·$x$$H_2O$; M_R = 5739; 1 step; based on palm^{11}-PrRP31·7.8TFA·12.4H_2O). **Recovery**: 2.9 mg of [**HCO_2CPh{Lu}**]$^+$[TFA]$^-$ (34% of the initial amount used assuming same TFA and water content). **ESI-HRMS**: 965.8860 [M+4H]$^{5+}$ (theor. [$C_{215}H_{328}N_{64}O_{51}S_1Lu_1$]$^{5+}$ = 965.8828). **UV absorption:** λ_{max} = 283 nm.

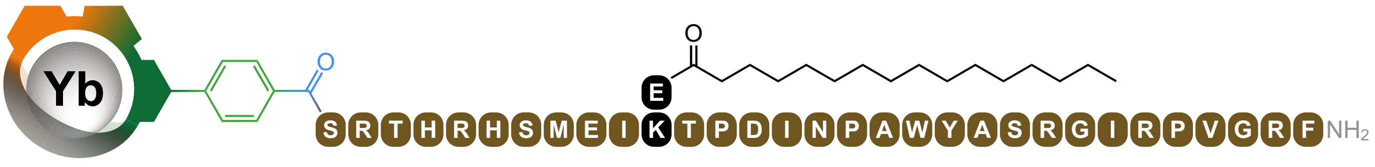

{Yb}palm^{11}-PrRP31

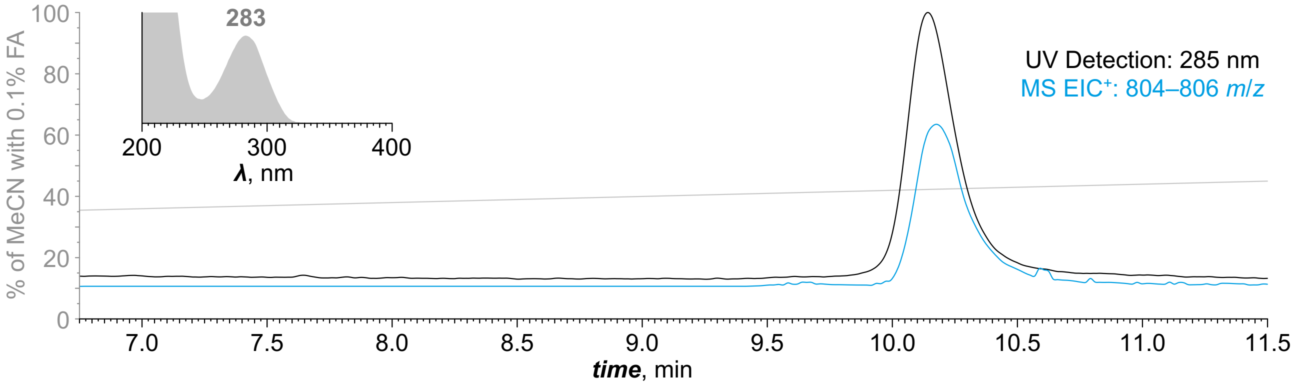

Supplementary Fig. 115. Synthesis and characterization of {Yb}palm^{11}-PrRP31. In a plastic Eppendorf test tube (2 mL), [**HCO_2CPh**{**Yb**}]$^+$[TFA]$^-$·2.1TFA·2.0H_2O (30 mM in DMSO; 95 µL, 2.9 µmol; 1.5 equiv.) was diluted with freshly prepared solution of PyOAP (200 mM in DMSO; 14.2 µL; 2.8 µmol; 1.5 equiv.) followed by addition of NMM (400 mM in DMF; 95 µL; 38.0 µmol; 20 equiv.). The tube was vortexed for 30 seconds and the resulting pale-yellow solution was then quickly added to a stirred solution (in a separate 4 mL glass vial) of palm^{11}-PrRP31·7.8TFA·12.4H_2O peptide (20 mM in DMSO; 95 µL; 1.9 µmol; 1.0 equiv.). The resulting yellow solution was quickly briefly vortexed and then further stirred for 30 min at RT. Reaction was quenched by addition of neat TFA (2.9 µL; 37.9 µmol; 20 equiv.) and further diluted with H_2O (2 mL) and 25% MeOH (500 µL). Resulting solution was directly purified by preparative HPLC (C18; H_2O–MeCN gradient with TFA additive). Fractions with pure product were joined and directly lyophilized. The resulting white foam was re-dissolved in H_2O (3 mL) and re-purified by preparative HPLC (C18; H_2O–MeCN gradient with TFA additive). Fractions with pure product were joined and directly lyophilized to give product in the form of trifluoroacetate salt as white fluffy solid. **Yield:** 3.3 mg (≤30% assuming [**M**]$^+$[TFA]$^-$·7.0TFA·$x$$H_2O$; M_R = 5738; 1 step; based on palm^{11}-PrRP31·7.8TFA·12.4H_2O). **Recovery**: 0.9 mg of [**HCO_2CPh**{**Yb**}]$^+$[TFA]$^-$ (26% of the initial amount used assuming same TFA and water content). **ESI-HRMS**: 965.8854 $[M+4H]^{5+}$ (theor. $[C_{215}H_{328}N_{64}O_{51}S_1Yb_1]^{5+}$ = 965.8840). **UV absorption:** λ_{max} = 283 nm.

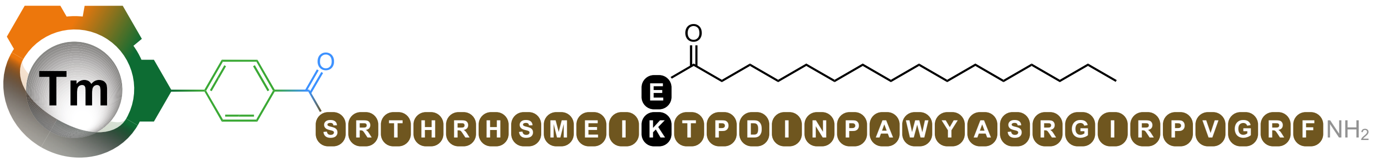

{Tm}palm11-PrRP31

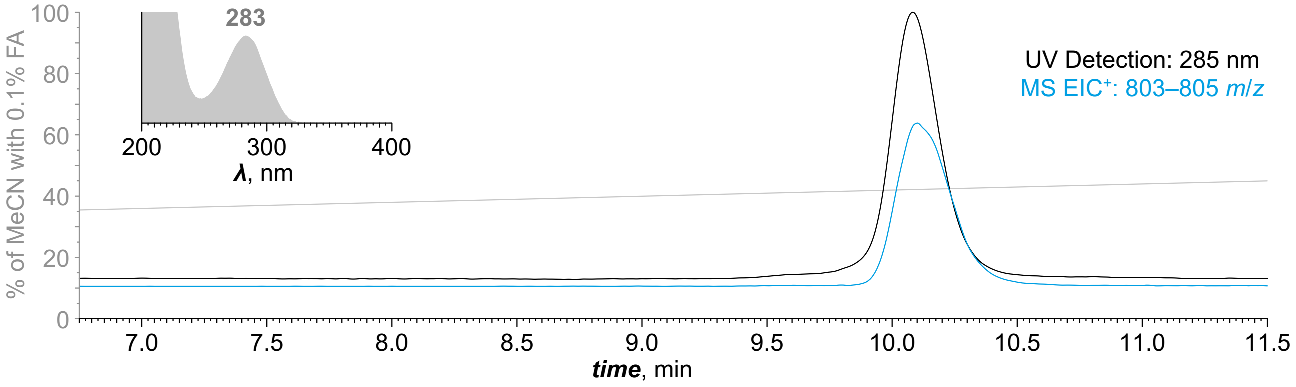

Supplementary Fig. 116. Synthesis and characterization of {Tm}palm^{11}-PrRP31. In a plastic Eppendorf test tube (2 mL), [**HCO_2CPh{Tm}**]$^+$[TFA]$^-$·1.1TFA·2.6H_2O (30 mM in DMSO; 198 µL, 6.0 µmol; 1.5 equiv.) was diluted with freshly prepared solution of PyOAP (200 mM in DMSO; 30 µL; 6.0 µmol; 1.5 equiv.) followed by addition of NMM (400 mM in DMF; 198 µL; 79.2 µmol; 20 equiv.). The tube was vortexed for 30 seconds and the resulting pale-yellow solution was then quickly added to a stirred solution (in a separate 4 mL glass vial) of palm^{11}-PrRP31·7.8TFA·12.4H_2O peptide (20 mM in DMSO; 198 µL; 4.0 µmol; 1.0 equiv.). The resulting yellow solution was quickly briefly vortexed and then further stirred for 30 min at RT. Reaction was quenched by addition of neat TFA (6.0 µL; 78.4 µmol; 20 equiv.) and further diluted with H_2O (2 mL) and 25% MeOH (500 µL). Resulting solution was directly purified by preparative HPLC (C18; H_2O–MeCN gradient with TFA additive). Fractions with pure product were joined and directly lyophilized. The resulting white foam was re-dissolved in H_2O (3 mL) and re-purified by preparative HPLC (C18; H_2O–MeCN gradient with TFA additive). Fractions with pure product were joined and directly lyophilized to give product in the form of trifluoroacetate salt as white fluffy solid. **Yield:** 16.5 mg (≤73% assuming [**M**]$^+$[TFA]$^-$·7.0TFA·$x$$H_2O$; M_R = 5734; 1 step; based on palm^{11}-PrRP31·7.8TFA·12.4H_2O). **Recovery**: 2.9 mg of [**HCO_2CPh{Tm}**]$^+$[TFA]$^-$ (38% of the initial amount used assuming same TFA and water content). **ESI-HRMS**: 964.6831 [M+4H]$^{5+}$ (theor. [$C_{215}H_{328}N_{64}O_{51}S_1Tm_1$]$^{5+}$ = 964.6815). **UV absorption:** λ_{max} = 283 nm.

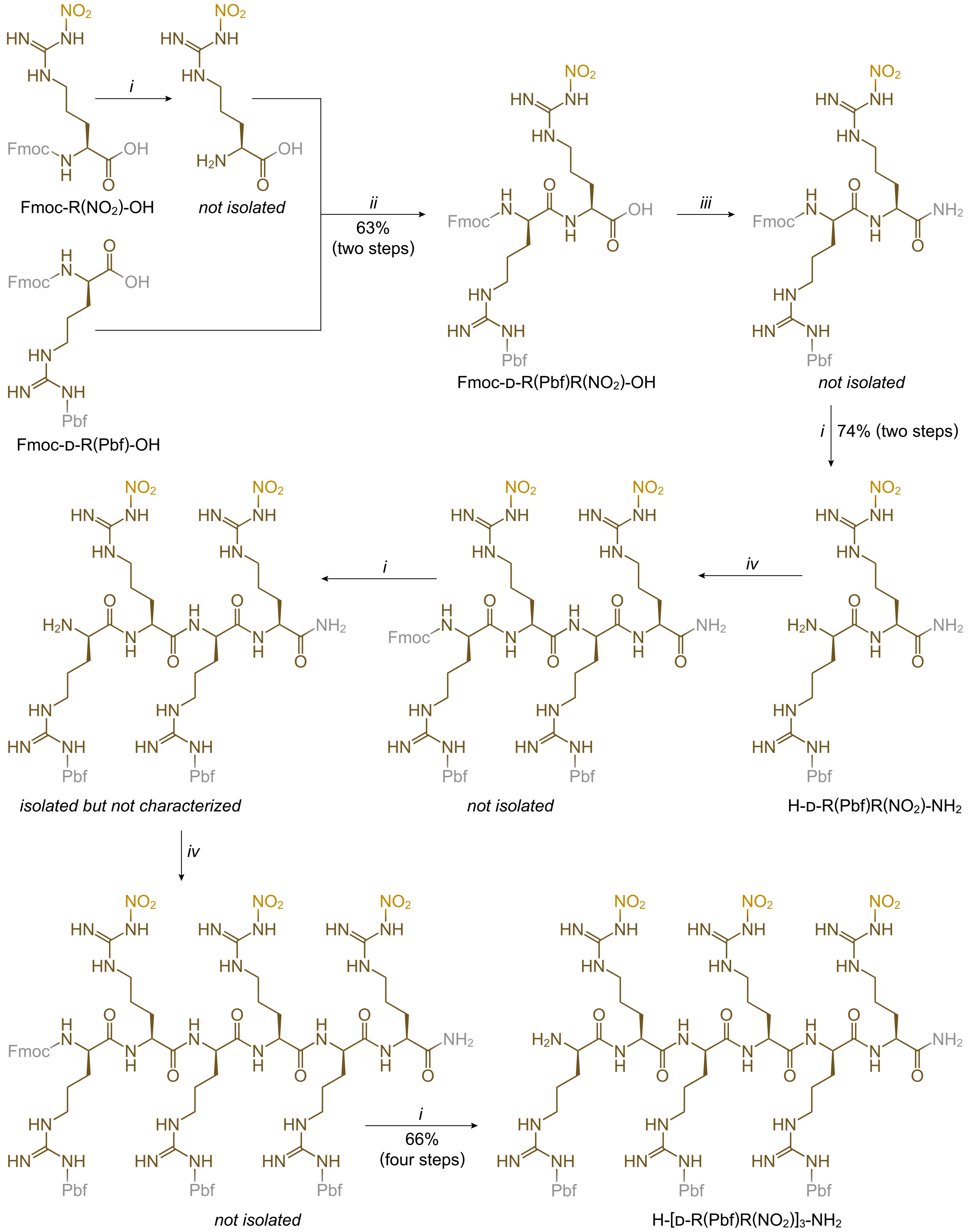

Supplementary Fig. 117. Cell penetrating peptide intermediate. The Pbf/NO_2 protected hexaarginine intermediate H-[D-R(Pbf)R(NO_2)]$_3$-NH_2 with alternating chirality was prepared by eight step synthesis from two arginine building blocks (Fmoc-D-R(Pbf)-OH and Fmoc-R(NO_2)-OH). Entire synthesis was conducted using solution chemistry (i.e. no SPPS) with 31% overal yield. ***Conditions:*** (*i*) DBU, DMSO, RT; (*ii*) PyOAP, DMSO, NMM, RT; (*iii*) NH_4Cl, PyOAP, DMSO, NMM, RT; (*iv*) Fmoc-D-R(Pbf)R(NO_2)-OH, PyOAP, DMSO, NMM, RT.

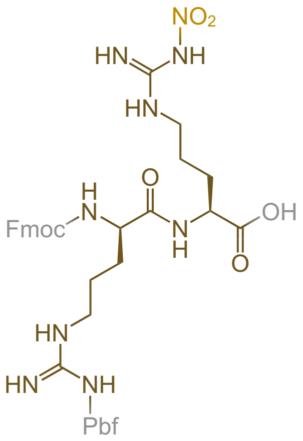

Fmoc-D-R(Pbf)R(NO_2)-OH

Supplementary Fig. 118. Synthesis and characterization of Fmoc-D-R(Pbf)R(NO_2)-OH. In a glass vial #1 (20 mL), Fmoc-R(NO_2)-OH (886 mg; 2.01 mmol; 1.1 equiv.) was dissolved (with the help of vortexing/gentle heating) in DMSO (6 mL) followed by addition of DBU (550 µL; 3.68 mmol; 2.0 equiv.) and the resulting solution was briefly vortexed and further stirred for 2 mins at RT. In a glass vial #2 (20 mL), Fmoc-D-R(Pbf)-OH (1.20 g; 1.85 mmol; 1.0 equiv.) was dissolved (with the help of vortexing/gentle heating) in a mixture of DMSO (9 mL) and NMM (1.0 mL; 9.09 mmol; 4.9 equiv.) followed by addition of solid PyAOP (965 mg; 1.85 mmol; 1.0 equiv.). The mixture was briefly vortexed and the resulting slightly yellow solution was further stirred for 2 mins at RT). Solution from vial #1 was then added in one portion (via syringe) to the intensively stirred solution in vial #2. Resulting bright yellow solution was further gently stirred for 5 mins at RT after which neat TFA (430 µL; 5.62 mmol; 3.0 equiv.) was added. Mixture was then directly purified by flash chromatography (C18; H_2O–MeCN gradient with TFA additive). Fractions with pure product were joined and directly lyophilized. Fractions containing impure product were joined, lyophilized and subsequently re-purified by preparative HPLC (C18; H_2O–MeCN gradient with TFA additive) and re-lyophilized. Both solids were then joined and homogenized to give product as off-white solid. **Yield:** 1.21 g (63%; 2 steps; based on Fmoc-D-R(Pbf)-OH). **ESI-MS** (LC-MS)**:** 850.4 $[M+H]^+$ (theor. $[C_{40}H_{52}N_9O_{10}S_1]^+$ = 850.3). **EA** ($C_{40}H_{51}N_9O_{10}S_1 \cdot 1.4TFA \cdot 1.4H_2O$, M_R = 1034.8): C 49.7 (50.0); H 5.4 (5.1); N 12.2 (11.8); F 7.7 (7.8).

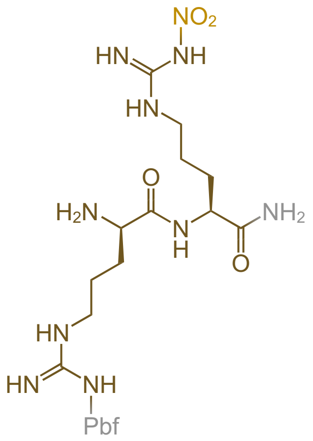

H-D-R(Pbf)R(NO_2)-NH_2

Supplementary Fig. 119. Synthesis and characterization of H-D-R(Pbf)R(NO_2)-NH_2. In a glass vial (20 mL), Fmoc-D-R(Pbf)R(NO_2)-OH·1.4TFA·1.4H_2O (360 mg; 348 µmol; 1.1 equiv.) and NH_4Cl (27 mg; 505 µmol; 1.5 equiv.) were dissolved (with the help of vortexing/gentle heating) in DMSO (2 mL) followed by addition of NMM (140 µL; 1.27 mmol; 3.0 equiv.) and of solid PyAOP (264 mg; 506 µmol; 1.5 equiv.). Mixture was briefly vortexed and the resulting bright yellow solution was further stirred for 5 mins at RT followed by addition of DBU (380 µL; 2.54 mmol; 7.3 equiv.). Mixture was briefly vortexed and the resulting bright yellow solution was further stirred for 2 mins at RT after which neat TFA (160 µL; 2.09 mmol; 6.0 equiv.) was added. Mixture was then directly purified by preparative HPLC (C18; H_2O–MeCN gradient with TFA additive). Fractions with pure product were joined and directly lyophilized to give product as white fluffy solid. **Yield:** 237 mg (74%; 2 steps; based on Fmoc-D-R(Pbf)R(NO_2)-OH·1.4TFA·1.4H_2O). **ESI-MS** (LC-MS)**:** 627.4 $[M+H]^+$ (theor. $[C_{25}H_{42}N_{10}O_7S_1]^+$ = 627.3). **EA** ($C_{25}H_{42}N_{10}O_7S_1$·2.3TFA·1.9H_2O, M_R = 923.2): C 38.5 (38.7); H 5.3 (4.9); N 15.2 (14.8); F 14.2 (14.0).

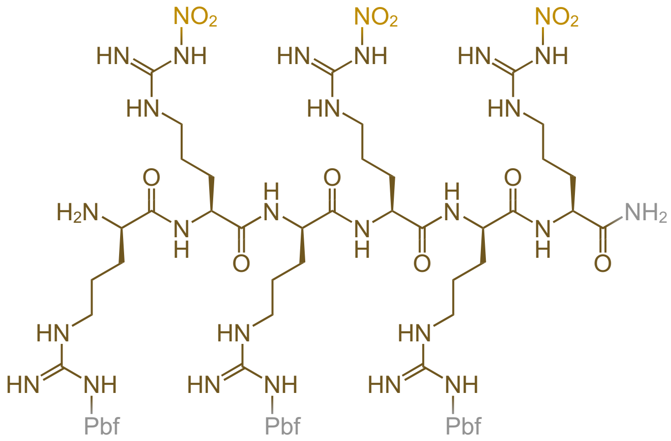

H-[D-R(Pbf)R(NO_2)]$_3$-NH_2

Supplementary Fig. 120. Synthesis and characterization of H-[D-R(Pbf)R(NO_2)]$_3$-NH_2. In a glass vial (20 mL), Fmoc-D-R(Pbf)R(NO_2)-OH·1.4TFA·1.4H_2O (258 mg; 249 µmol; 1.0 equiv.) and H-D-R(Pbf)R(NO_2)-NH_2·2.3TFA·1.9H_2O (222 mg; 240 µmol; 1.0 equiv.) were dissolved (with the help of vortexing/gentle heating) in DMSO (2 mL) followed by addition of NMM (220 µL; 2.00 mmol; 8.3 equiv.) and of solid PyAOP (136 mg; 261 µmol; 1.1 equiv.). Mixture was briefly vortexed and the resulting bright yellow solution was further stirred for 15 mins at RT followed by addition of DBU (221 µL; 1.48 mmol; 6.2 equiv.). Mixture was briefly vortexed and the resulting bright yellow solution was further stirred for 15 mins at RT after which another portion of DBU (36 µL; 241 µmol; 1.0 equiv.) was added. Mixture was briefly vortexed and the resulting bright yellow solution was further stirred for 5 mins at RT after which neat TFA (58 µL; 758 µmol; 3.2 equiv.) was added. Mixture was then directly purified by preparative HPLC (C18; H_2O–MeCN gradient with TFA additive). Fractions with pure product were joined and directly lyophilized. The resulting H-[D-R(Pbf) R(NO_2)]$_2$-NH_2 intermediate (≤238 µmol; 1.0 equiv.) was transferred to a glass vial (20 mL) followed by addition of Fmoc-D-R(Pbf)R(NO_2)-OH·1.4TFA·1.4H_2O (259 mg; 250 µmol; ≥1.1 equiv.), DMSO (2 mL; dissolved with the help of vortexing/gentle heating), NMM (276 µL; 2.51 mmol; ≥10.5 equiv.) and of solid PyAOP (144 mg; 277 µmol; ≥1.2 equiv.). Mixture was briefly vortexed and the resulting bright yellow solution was further stirred for 20 mins at RT followed by addition of DBU (263 µL; 1.76 mmol; ≥7.0 equiv.). Mixture was briefly vortexed and the resulting bright yellow solution was further stirred 5 mins at RT after which neat TFA (77 µL; 1.00 mmol; ≥4.0 equiv.) was added. Mixture was then directly purified by preparative HPLC (C18; H_2O–MeCN gradient with TFA additive). Fractions with pure product were joined and directly lyophilized to give product as white fluffy solid. **Yield:** 368 mg (66%; 4 steps; based on H-D-R(Pbf)R(NO_2)-NH_2·2.3TFA·1.9H_2O). **ESI-MS** (LC-MS)**:** 923.6 $[M+2H]^{2+}$ (theor. $[C_{75}H_{120}N_{28}O_{21}S_3]^{2+}$ = 923.4). **EA** ($C_{75}H_{120}N_{28}O_{21}S_3$·3.2TFA·5.3$H_2O$, M_R = 2306.4): C 42.4 (42.8); H 5.8 (5.4); N 17.0 (16.6); F 7.9 (7.6).

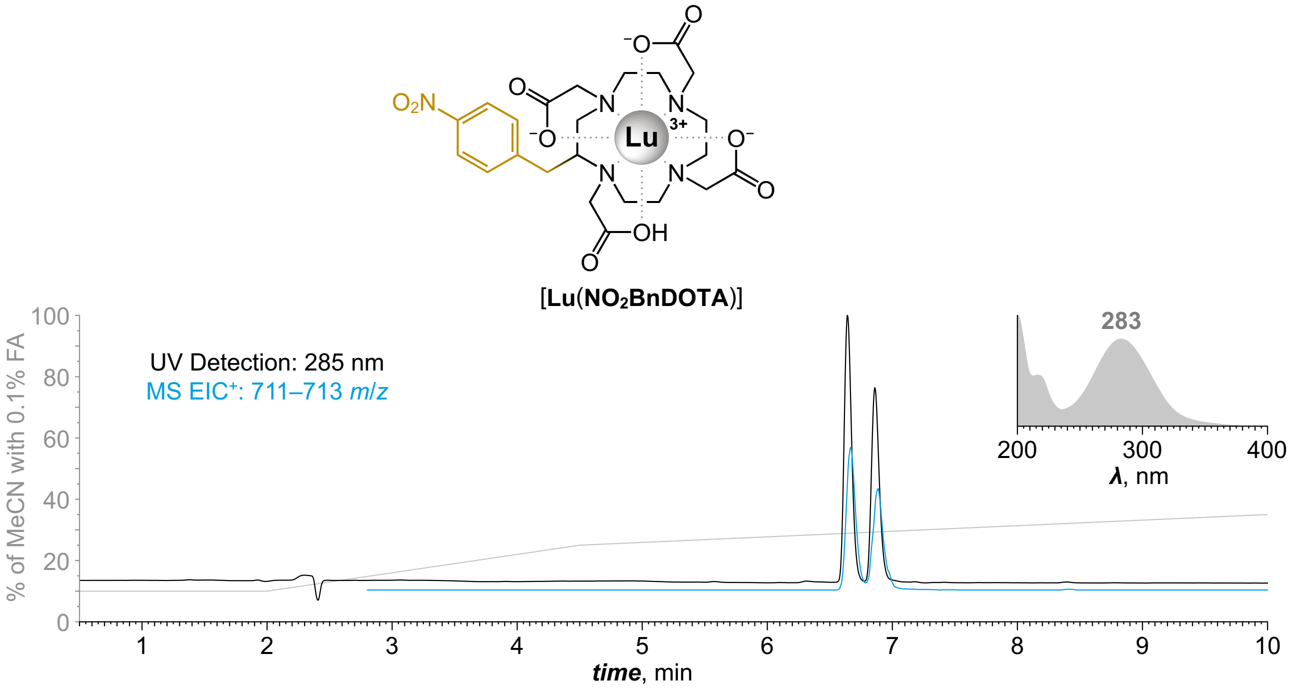

Supplementary Fig. 121. Preparation of [Lu(NO_2BnDOTA)] stock solution. In a glass vial (2 mL), **NO_2BnDOTA**·4HCl (*mixture of enantiomers*; 10 mM; 100 µL; 1.0 µmol; 1.0 equiv.) was mixed with $LuCl_3$ (46.8 mM; 23.5 µL; 1.1 µmol; 1.1 equiv.) and H_2O (59.5 µL) followed by addition of freshly prepared solution of NaOH (400 mM; 17.5 µL; 7.0 µmol; 7.0 equiv.). After the addition, the color changed to pale yellow. Resulting slighly opalescent mixture was stirred for 16 h at RT (full conversion; analysed by LC-MS). The mixture was then vortexed and centrifuged. The supernatant was then transferred to another vial, and the resulting 5.0 mM [**Lu**(**NO_2BnDOTA**)] (*as a mixture of two diastereoisomers*) was used directly for assesment of kinetic inertness. **ESI-MS** (LC-MS)**:** 712.2 $[M+H]^+$ (theor. $[C_{23}H_{31}N_5O_{10}Lu_1]^{2+}$ = 712.1). **UV absorption:** λ_{max} = 283 nm.

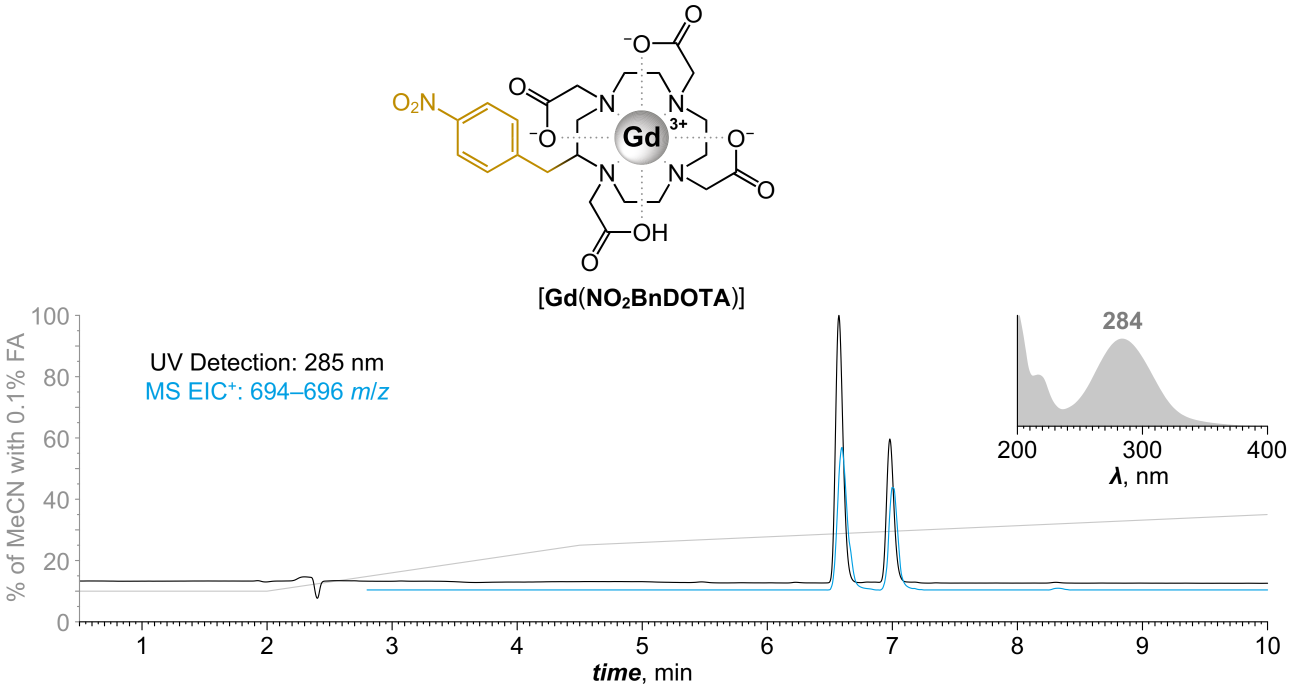

Supplementary Fig. 122. Preparation of [Gd(NO_2BnDOTA)] stock solution. In a glass vial (2 mL), **NO_2BnDOTA**·4HCl (*mixture of enantiomers*; 10 mM; 100 µL; 1.0 µmol; 1.0 equiv.) was mixed with $GdCl_3$ (58.0 mM; 19.0 µL; 1.1 µmol; 1.1 equiv.) and H_2O (63.5 µL) followed by addition of freshly prepared solution of NaOH (400 mM; 17.5 µL; 7.0 µmol; 7.0 equiv.). After the addition, the color changed to pale yellow. Resulting slighly opalescent mixture was stirred for 16 h at RT (full conversion; analysed by LC-MS). The mixture was then vortexed and centrifuged. The supernatant was then transferred to another vial, and the resulting 5.0 mM [**Gd**(**NO_2BnDOTA**)] (*as a mixture of two diastereoisomers*) was used directly for assesment of kinetic inertness. **ESI-MS** (LC-MS)**:** 695.2 $[M+H]^+$ (theor. $[C_{23}H_{31}N_5O_{10}Gd_1]^{2+}$ = 695.1). **UV absorption:** λ_{max} = 284 nm.

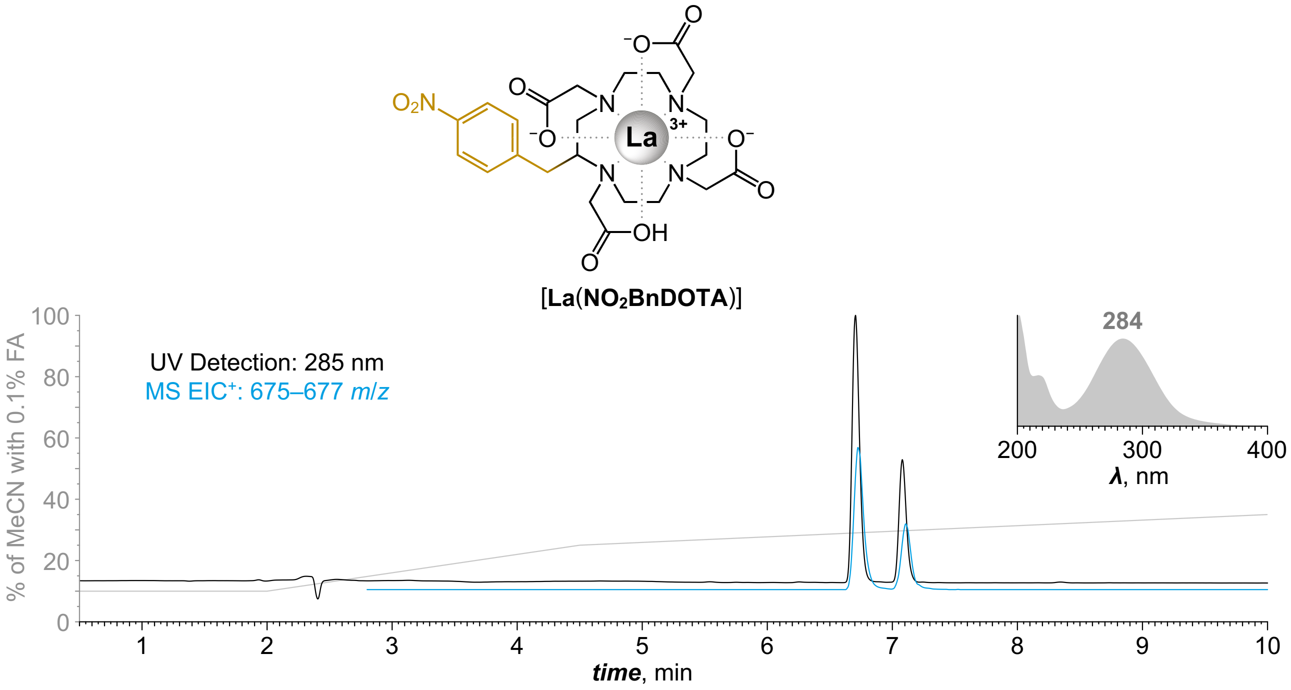

Supplementary Fig. 123. Preparation of [La(NO_2BnDOTA)] stock solution. In a glass vial (2 mL), **NO_2BnDOTA**·4HCl (*mixture of enantiomers*; 10 mM; 100 µL; 1.0 µmol; 1.0 equiv.) was mixed with $LaCl_3$ (100.0 mM; 11.0 µL; 1.1 µmol; 1.1 equiv.) and H_2O (71.5 µL) followed by addition of freshly prepared solution of NaOH (400 mM; 17.5 µL; 7.0 µmol; 7.0 equiv.). After the addition, the color changed to pale yellow. Resulting slighly opalescent mixture was stirred for 16 h at RT (full conversion; analysed by LC-MS). The mixture was then vortexed and centrifuged. The supernatant was then transferred to another vial, and the resulting 5.0 mM [**La**(**NO_2BnDOTA**)] (*as a mixture of two diastereoisomers*) was used directly for assesment of kinetic inertness. **ESI-MS** (LC-MS)**:** 676.1 $[M+H]^+$ (theor. $[C_{23}H_{31}N_5O_{10}La_1]^{2+}$ = 676.1). **UV absorption:** λ_{max} = 284 nm.

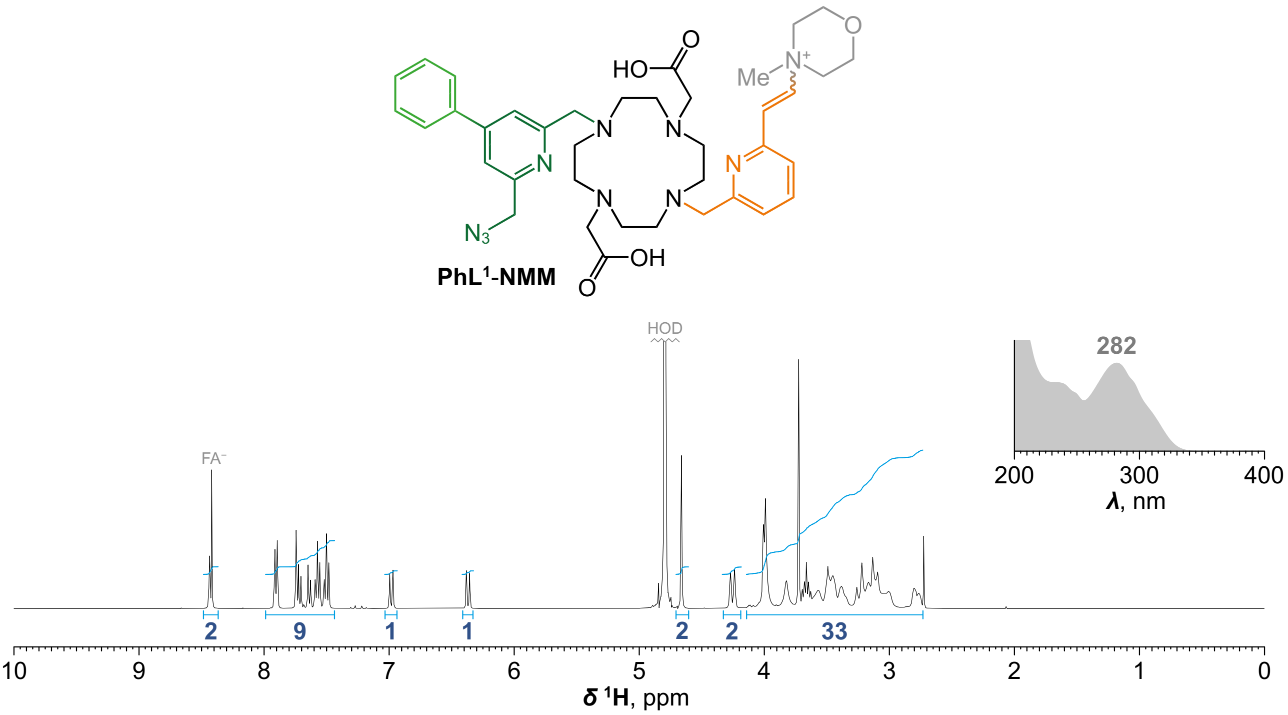

Supplementary Fig. 124. Synthesis and characterization of PhL[1]-NMM adduct. In a glass vial (4 mL), **PhL[1]**·1.3H_2O (10.0 mg; 15.4 μmol; 1.0 equiv.) was dissolved in dry H_2O (3 mL) followed by addition of NMM (85 μL; 774 μmol; 50 equiv.). The resulting solution was stirred for 12 h at 80 °C. The mixture was evaporated to dryness and the residue was purified by preparative HPLC (C18; H_2O–MeCN gradient with FA additive). Fractions with major isomer (the minor isomer was not collected) were combined and directly lyophilized to give product in the form of formate salt as white fluffy solid. **Yield:** 4.8 mg (38%; 1 steps; based on **PhL[1]**·1.3H_2O). **NMR (D_2O): ^{1}H** (401.0 MHz) δ_H 2.73–4.14 (CH_2–N, CH_2–CO, CH_2–arom., CH_2, m, 16+4+4+9H); 4.18–4.33 (CH_2, m, 2H); 4.66 (CH_2–N_3, s, 2H); 6.37 (CH_2–C*H*=CH, d, 1H, $^3J_{HH}$ = 10.4); 6.98 (CH=C*H*–arom., d, 1H, $^3J_{HH}$ = 10.4); 7.45–7.76 (C*H*, m, 7H); 7.87–7.93 (C*H*, m, 2H); 8.41 (*FA*$^-$, s, 1H); 8.44 (C*H*, d, 1H, $^4J_{HH}$ = 1.6). **ESI-HRMS:** 727.4033 $[M]^+$ (theor. $[C_{38}H_{51}N_{10}O_5]^+$ = 727.4038). **UV absorption:** λ_{max} = 282 nm. ($[C_{38}H_{51}N_{10}O_5]^+[FA]^-\cdot 2.7H_2O$, M_R = 820.5): C 57.0 (56.9); H 7.0 (6.7); N 17.0 (16.8).

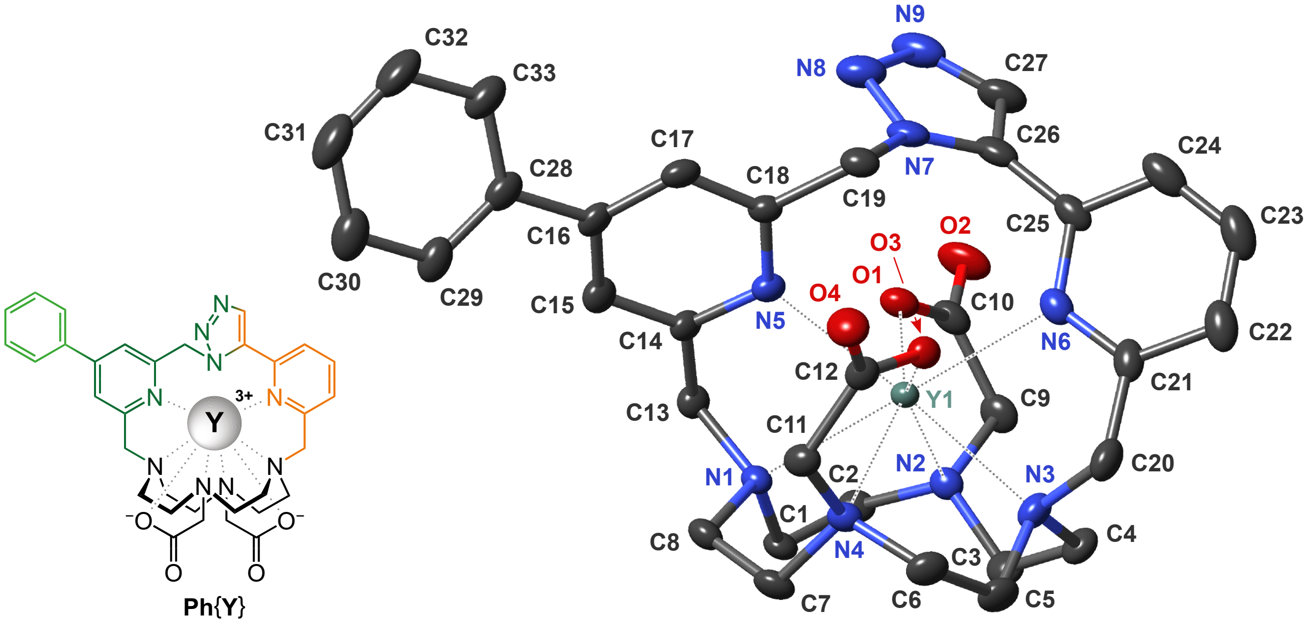

Supplementary Fig. 125. Solid-state structure of Ph{Y} in the solid state. Structure of [**Ph**{**Y**}]$^+$ cation found in the crystal structure of [**Ph**{**Y**}]$^+$[ClO$_4$]$^-$·4.0H$_2$O with twisted square antiprismatic (TSA) conformation of the chelate. The unit cell contains two symmetrically dependent units with opposite chirality (enantiomers Λλλλλ and Δδδδδ) of which only the latter is shown. The perchlorate anion, water molecules as well as carbon-bound hydrogen atoms were omitted for clarity reasons. Thermal ellipsoids were set at 50% probability.

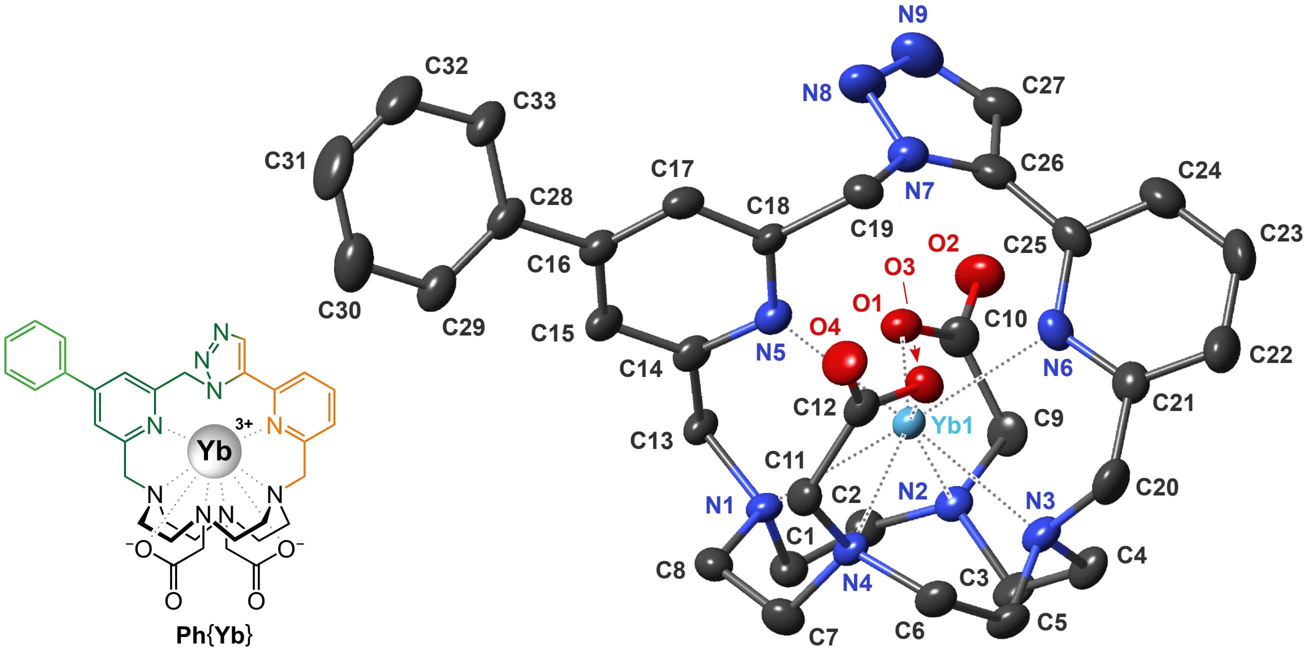

Supplementary Fig. 126. Solid-state structure of Ph{Yb} in the solid state. Structure of [**Ph**{**Yb**}]$^+$ cation found in the crystal structure of [**Ph**{**Yb**}]$^+$[ClO_4]$^-$·7.0H_2O with twisted square antiprismatic (TSA) conformation of the chelate. The unit cell contains two symmetrically dependent units with opposite chirality (enantiomers Λλλλλ and Δδδδδ) of which only the latter is shown. The perchlorate anion, water molecules as well as carbon-bound hydrogen atoms were omitted for clarity reasons. Thermal ellipsoids were set at 50% probability.

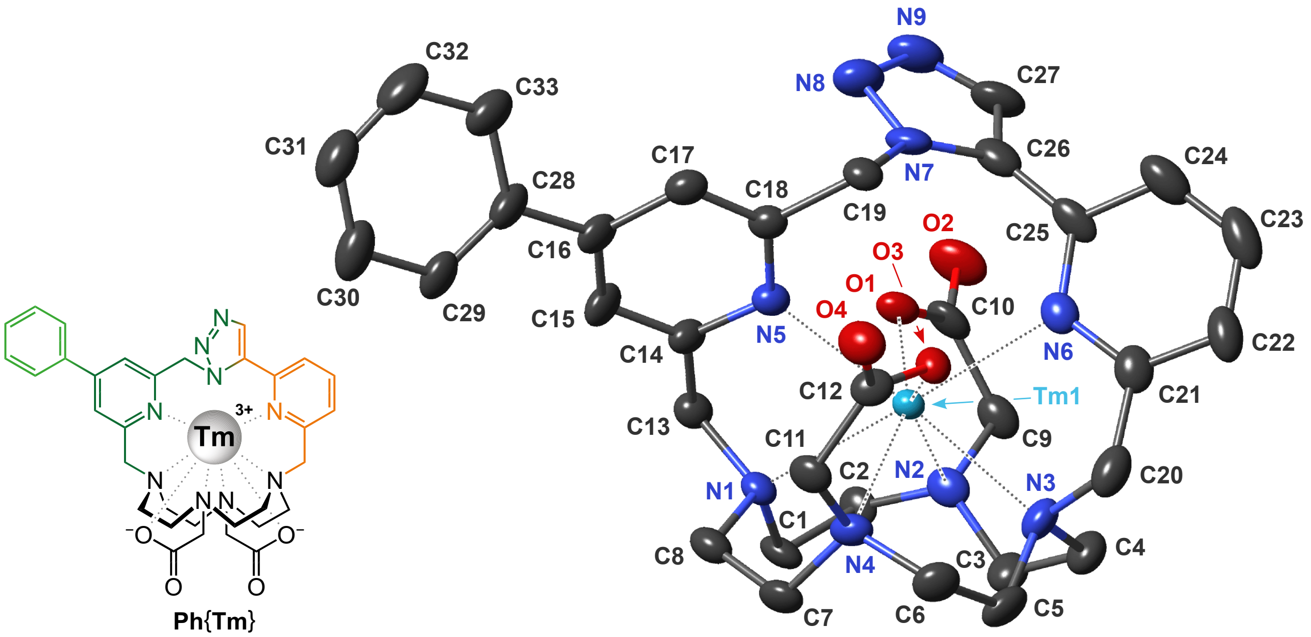

Supplementary Fig. 127. Solid-state structure of Ph{Tm} in the solid state. Structure of [**Ph**{**Tm**}]$^+$ cation found in the crystal structure of [**Ph**{**Tm**}]$^+$[ClO_4]$^-$·4.0H_2O with twisted square antiprismatic (TSA) conformation of the chelate. The unit cell contains two symmetrically dependent units with opposite chirality (enantiomers Λλλλλ and Δδδδδ) of which only the latter is shown. The perchlorate anion, water molecules as well as carbon-bound hydrogen atoms were omitted for clarity reasons. Thermal ellipsoids were set at 50% probability.

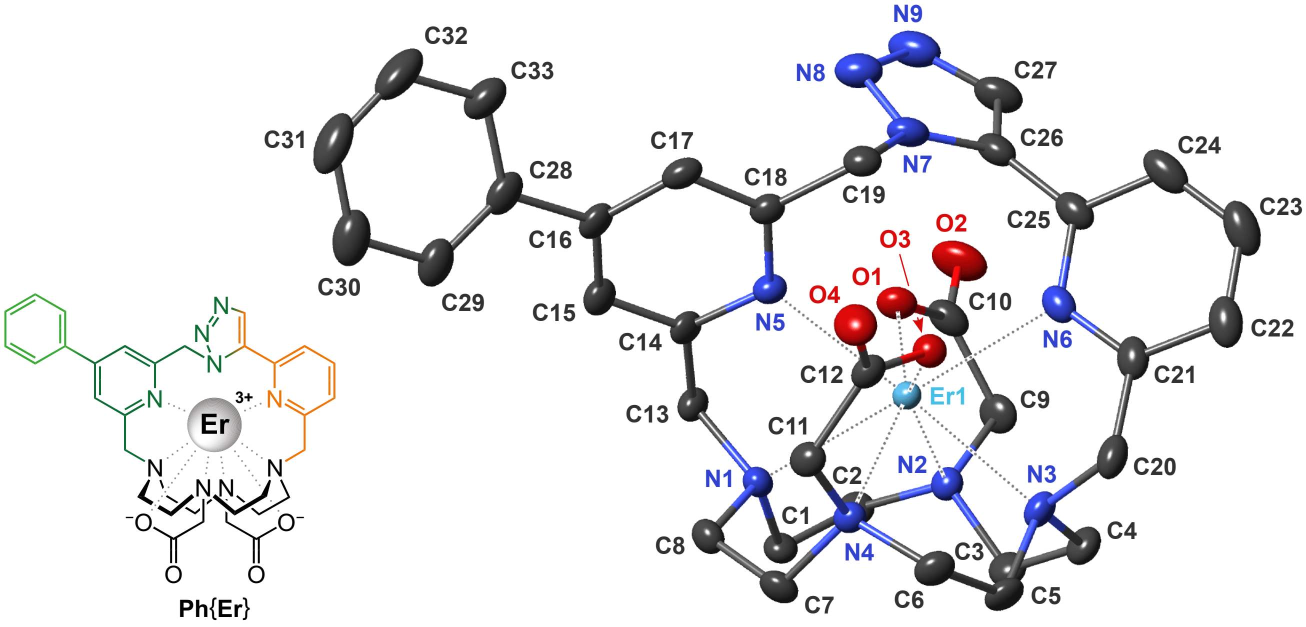

Supplementary Fig. 128. Solid-state structure of Ph{Er} in the solid state. Structure of [**Ph**{**Er**}]$^{+}$ cation found in the crystal structure of [**Ph**{**Er**}]$^{+}$[ClO_4]$^{-}$·4.0H_2O with twisted square antiprismatic (TSA) conformation of the chelate. The unit cell contains two symmetrically dependent units with opposite chirality (enantiomers Λλλλλ and Δδδδδ) of which only the latter is shown. The perchlorate anion, water molecules as well as carbon-bound hydrogen atoms were omitted for clarity reasons. Thermal ellipsoids were set at 50% probability.

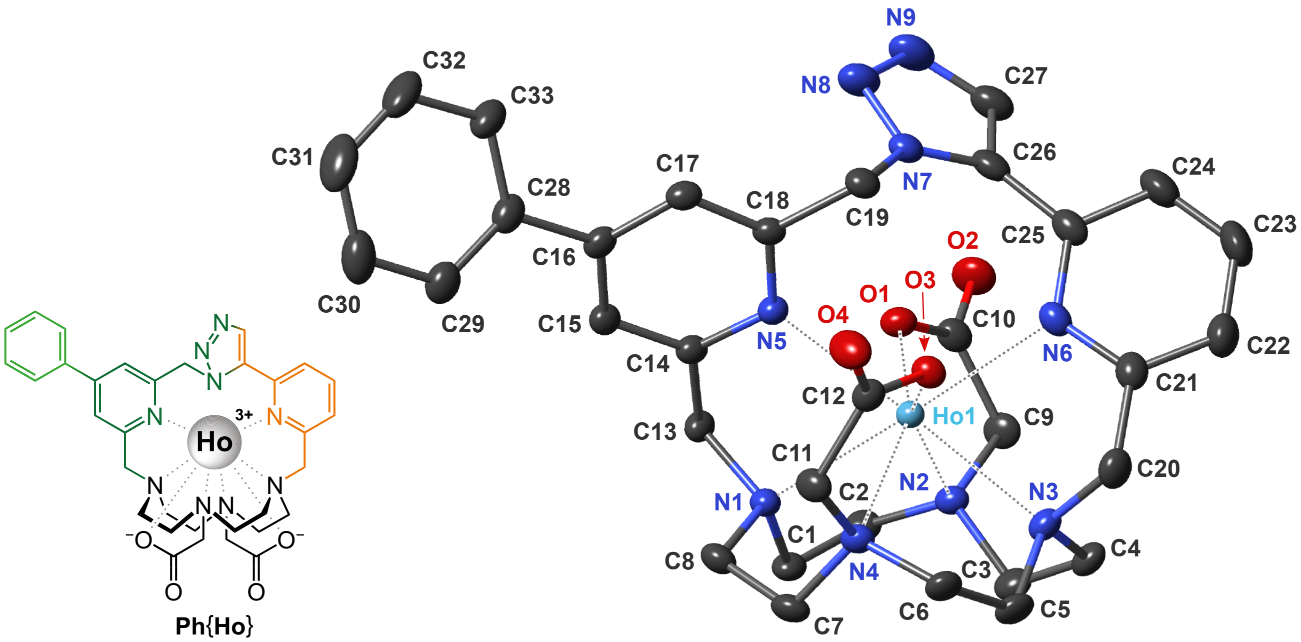

Supplementary Fig. 129. Solid-state structure of Ph{Ho} in the solid state. Structure of [**Ph**{**Ho**}]$^{+}$ cation found in the crystal structure of [**Ph**{**Ho**}]$^{+}$[ClO_4]$^{-}$·7.0H_2O with twisted square antiprismatic (TSA) conformation of the chelate. The unit cell contains two symmetrically dependent units with opposite chirality (enantiomers Λλλλλ and Δδδδδ) of which only the latter is shown. The perchlorate anion, water molecules as well as carbon-bound hydrogen atoms were omitted for clarity reasons. Thermal ellipsoids were set at 50% probability.

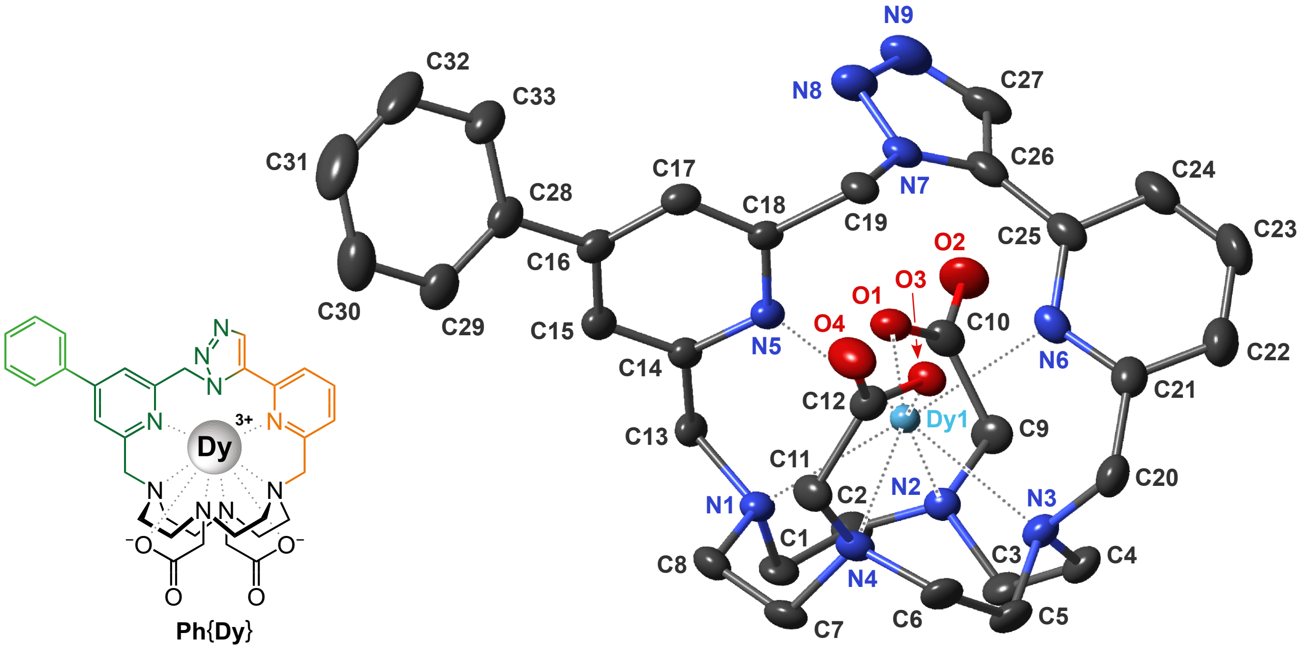

Supplementary Fig. 130. Solid-state structure of Ph{Dy} in the solid state. Structure of [**Ph**{**Dy**}]$^+$ cation found in the crystal structure of [**Ph**{**Dy**}]$^+$[ClO_4]$^-$·7.0H_2O with twisted square antiprismatic (TSA) conformation of the chelate. The unit cell contains two symmetrically dependent units with opposite chirality (enantiomers Λλλλλ and Δδδδδ) of which only the latter is shown. The perchlorate anion, water molecules as well as carbon-bound hydrogen atoms were omitted for clarity reasons. Thermal ellipsoids were set at 50% probability.

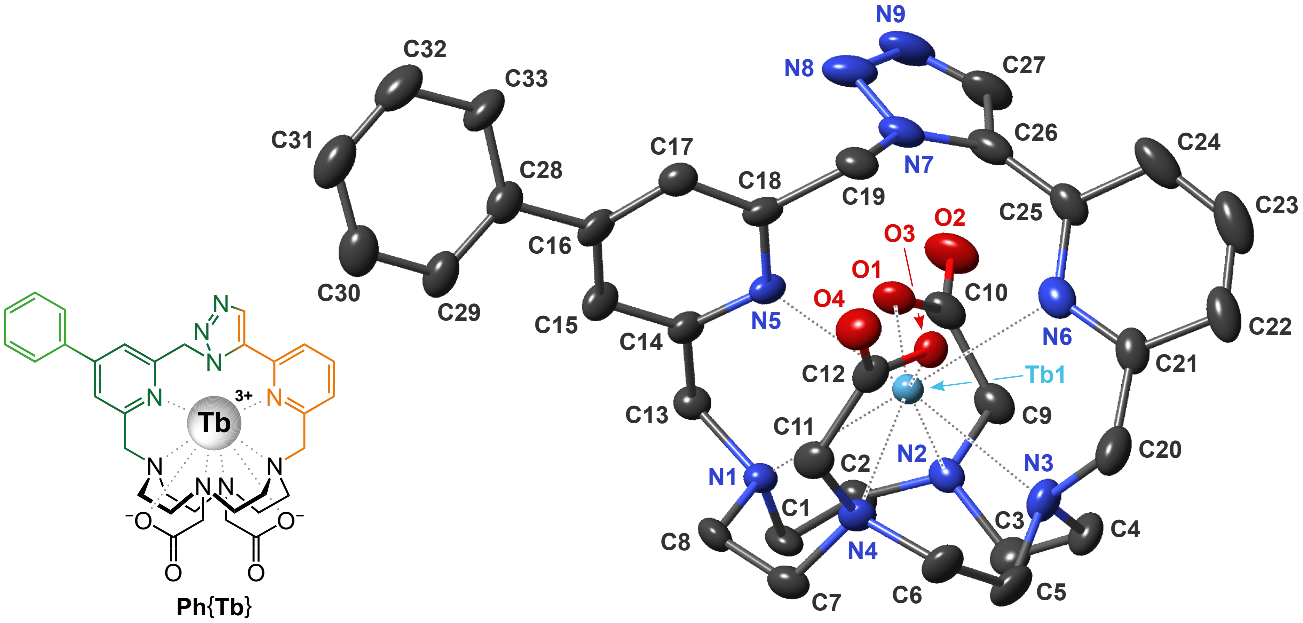

Supplementary Fig. 131. Solid-state structure of Ph{Tb} in the solid state. Structure of [**Ph**{**Tb**}]$^{+}$ cation found in the crystal structure of [**Ph**{**Tb**}]$^{+}$[ClO_4]$^{-}$·4.0H_2O with twisted square antiprismatic (TSA) conformation of the chelate. The unit cell contains two symmetrically dependent units with opposite chirality (enantiomers Λλλλλ and Δδδδδ) of which only the latter is shown. The perchlorate anion, water molecules as well as carbon-bound hydrogen atoms were omitted for clarity reasons. Thermal ellipsoids were set at 50% probability.

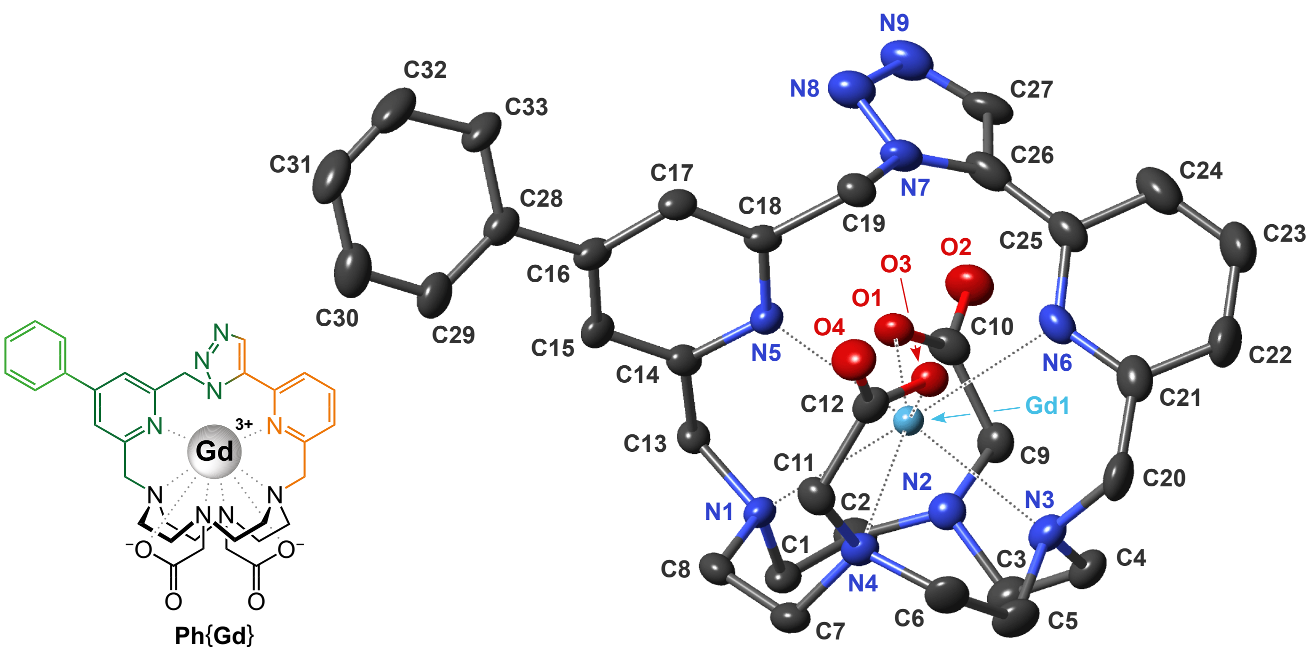

Supplementary Fig. 132. Solid-state structure of Ph{Gd} in the solid state. Structure of [**Ph**{**Gd**}]$^+$ cation found in the crystal structure of [**Ph**{**Gd**}]$^+$[ClO_4]$^-$·7.0H_2O with twisted square antiprismatic (TSA) conformation of the chelate. The unit cell contains two symmetrically dependent units with opposite chirality (enantiomers Λλλλλ and Δδδδδ) of which only the latter is shown. The perchlorate anion, water molecules as well as carbon-bound hydrogen atoms were omitted for clarity reasons. Thermal ellipsoids were set at 50% probability.

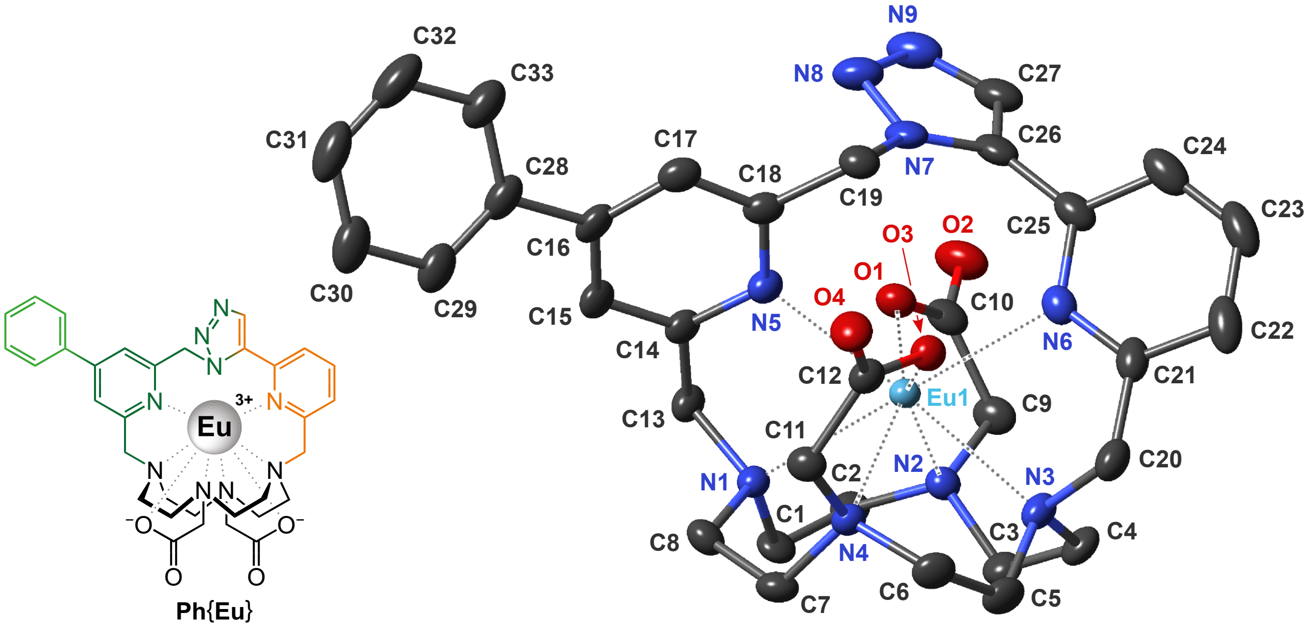

Supplementary Fig. 133. Solid-state structure of Ph{Eu} in the solid state. Structure of [**Ph**{**Eu**}]$^{+}$ cation found in the crystal structure of [**Ph**{**Eu**}]$^{+}$[ClO_4]$^{-}$·$4H_2O$ with twisted square antiprismatic (TSA) conformation of the chelate. The unit cell contains two symmetrically dependent units with opposite chirality (enantiomers Λλλλλ and Δδδδδ) of which only the latter is shown. The perchlorate anion, water molecules as well as carbon-bound hydrogen atoms were omitted for clarity reasons. Thermal ellipsoids were set at 50% probability.

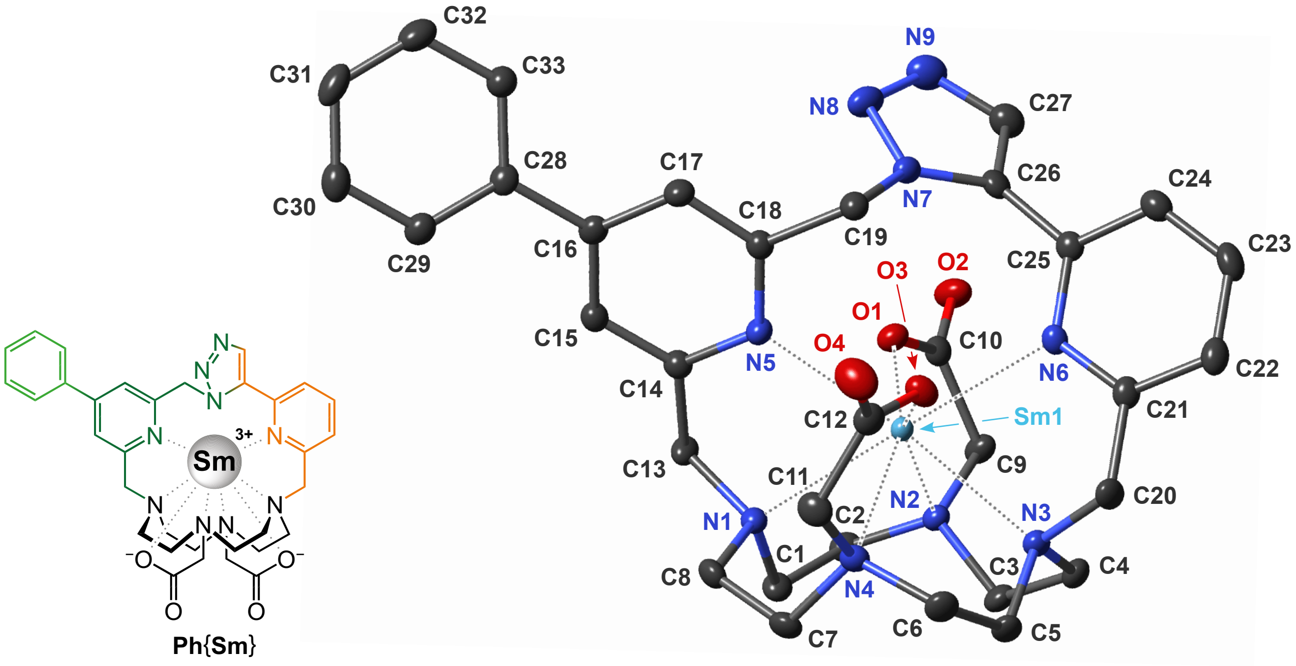

Supplementary Fig. 134. Solid-state structure of Ph{Sm} in the solid state. Structure of [**Ph**{**Sm**}]$^+$ cation found in the crystal structure of [**Ph**{**Sm**}]$^+$[ClO_4]$^-$ with twisted square antiprismatic (TSA) conformation of the chelate. The unit cell contains four symmetrically dependent units with opposite chirality (enantiomers Λλλλλ and Δδδδδ) of which only the latter is shown. The perchlorate anion, water molecules as well as carbon-bound hydrogen atoms were omitted for clarity reasons. Thermal ellipsoids were set at 50% probability.

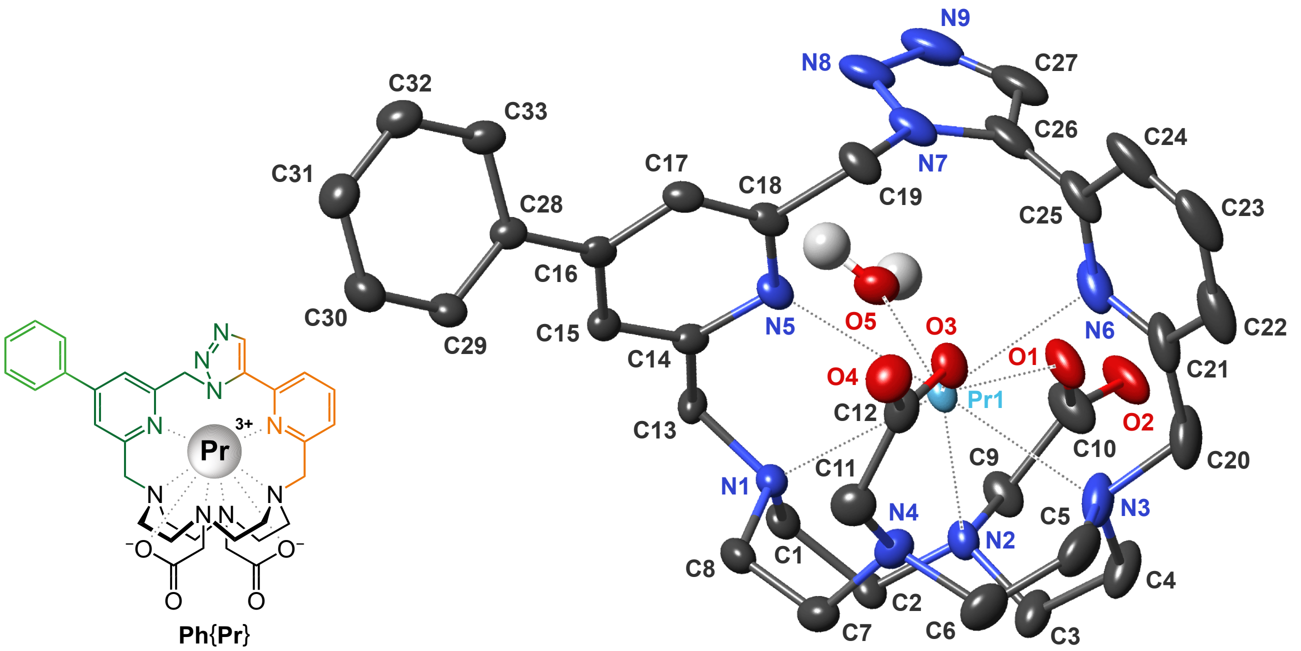

Supplementary Fig. 135. Solid-state structure of Ph{Pr} in the solid state. Structure of [**Ph**{**Pr**}(H_2O)]$^+$ cation found in the crystal structure of [**Ph**{**Pr**}(H_2O)]$^+$[ClO_4]$^-\cdot 6H_2O$ with twisted square antiprismatic (TSA) conformation of the chelate. The unit cell contains eight symmetrically dependent units with opposite chirality (enantiomers Λλλλλ and Δδδδδ) of which only the latter is shown. The presence of one coordinated water molecule on Pr^{III} ion distinguishes **Ph**{**Pr**} from the rest of isostructural series **Ph**{**Sm**}−**Ph**{**Lu**} (including **Ph**{**Y**}). The perchlorate anion, water molecules as well as carbon-bound hydrogen atoms were omitted for clarity reasons. Thermal ellipsoids were set at 50% probability.

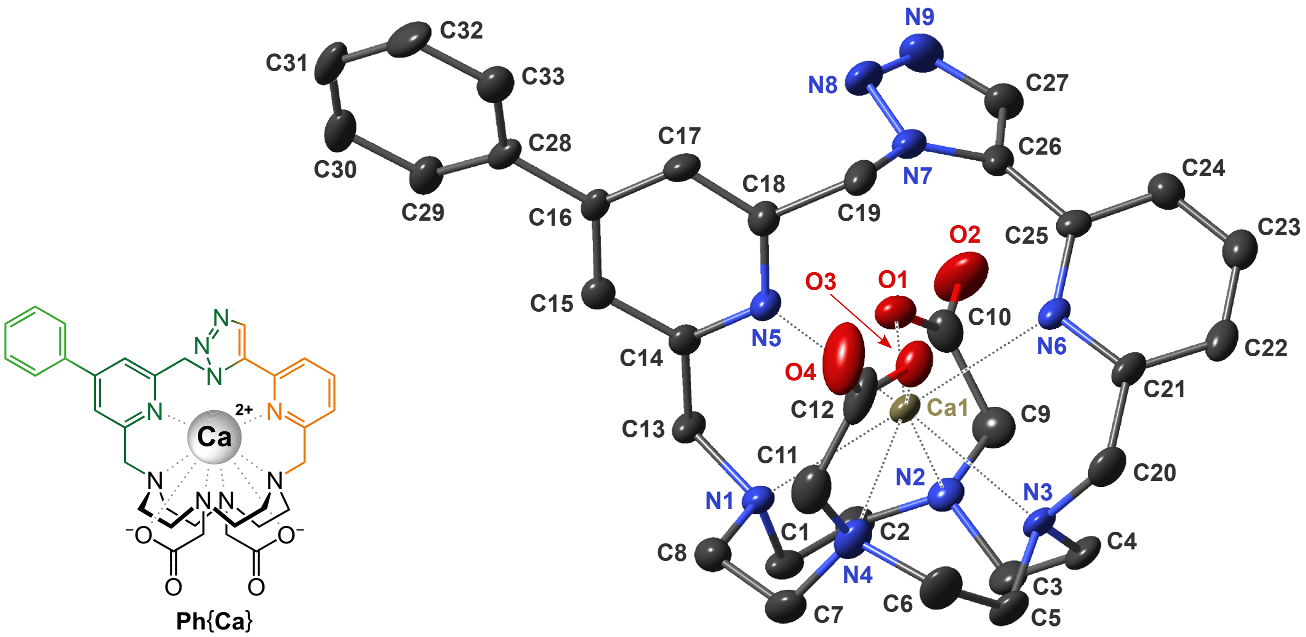

Supplementary Fig. 136. Solid-state structure of Ph{Ca} in the solid state. Structure of [**Ph**{**Ca**}] cation found in the crystal structure of [**Ph**{**Ca**}]·7H_2O with twisted square antiprismatic (TSA) conformation of the chelate. The unit cell contains two symmetrically dependent units with opposite chirality (enantiomers Λλλλλ and Δδδδδ) of which only the latter is shown. The water molecules as well as carbon-bound hydrogen atoms were omitted for clarity reasons. Thermal ellipsoids were set at 50% probability.

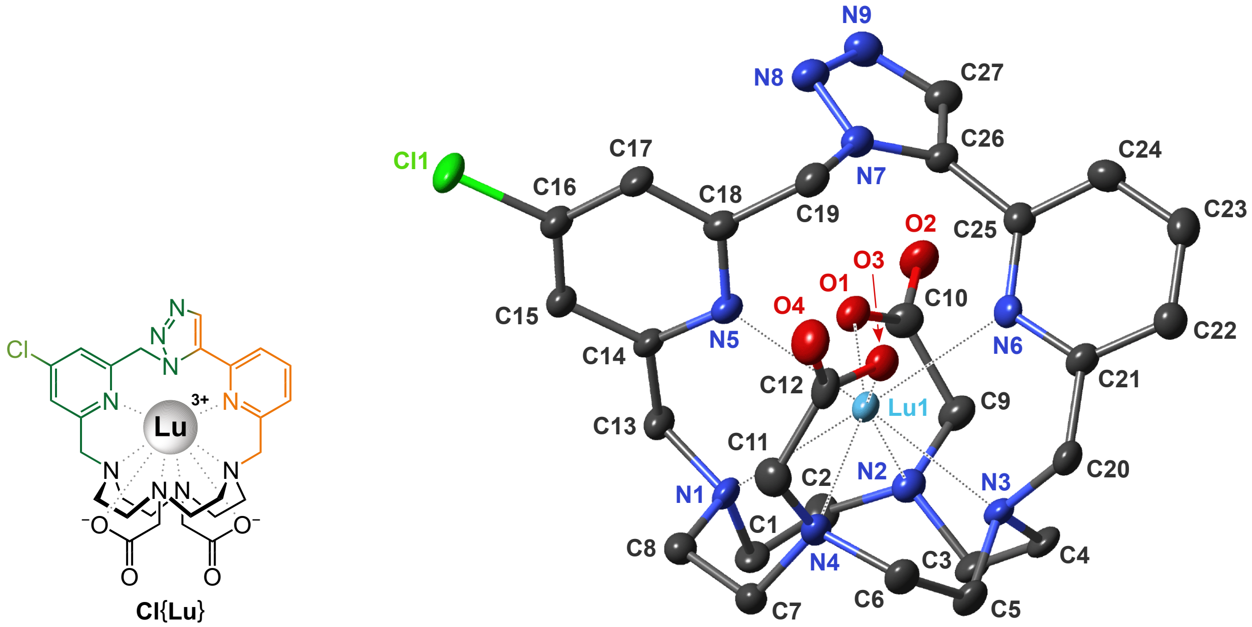

Supplementary Fig. 137. Solid-state structure of Cl{Lu} in the solid state. Structure of [**Cl**{**Lu**}]$^+$ cation found in the crystal structure of [**Cl**{**Lu**}]$^+$[ClO_4]$^-$·2H_2O with twisted square antiprismatic (TSA) conformation of the chelate. The unit cell contains four symmetrically dependent units with opposite chirality (enantiomers Λλλλλ and Δδδδδ) of which only the latter is shown. The perchlorate anion, water molecules as well as carbon-bound hydrogen atoms were omitted for clarity reasons. Thermal ellipsoids were set at 50% probability.

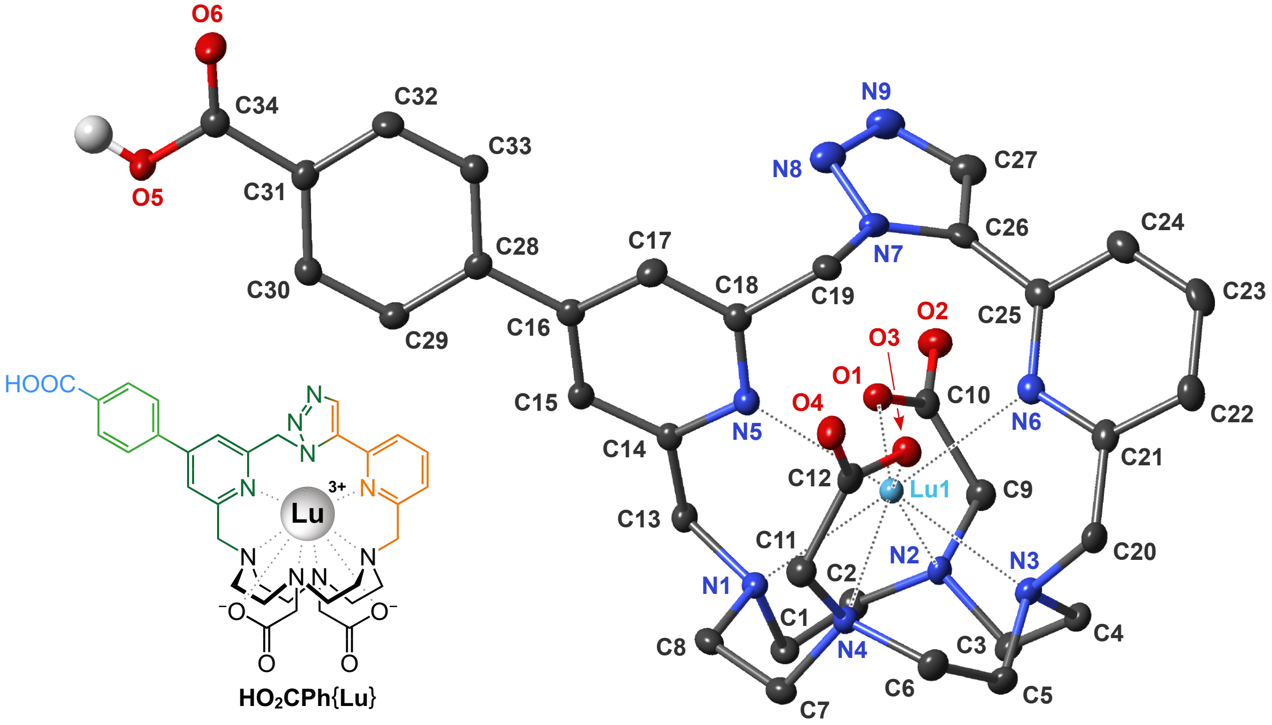

Supplementary Fig. 138. Solid-state structure of HO$_2$CPh{Lu} in the solid state. Structure of [**HO$_2$CPh**{**Lu**}]$^+$ cation (the distant carboxylic group is protonated and does not compensate Lu^{III} ion charge) found in the crystal structure of [**HO$_2$CPh**{**Lu**}]$^+$[ClO$_4$]$^-$ with twisted square antiprismatic (TSA) conformation of the chelate. The unit cell contains four symmetrically dependent units with opposite chirality (enantiomers Λλλλλ and Δδδδδ) of which only the latter is shown. The perchlorate anion, water molecules as well as carbon-bound hydrogen atoms were omitted for clarity reasons. Thermal ellipsoids were set at 50% probability.

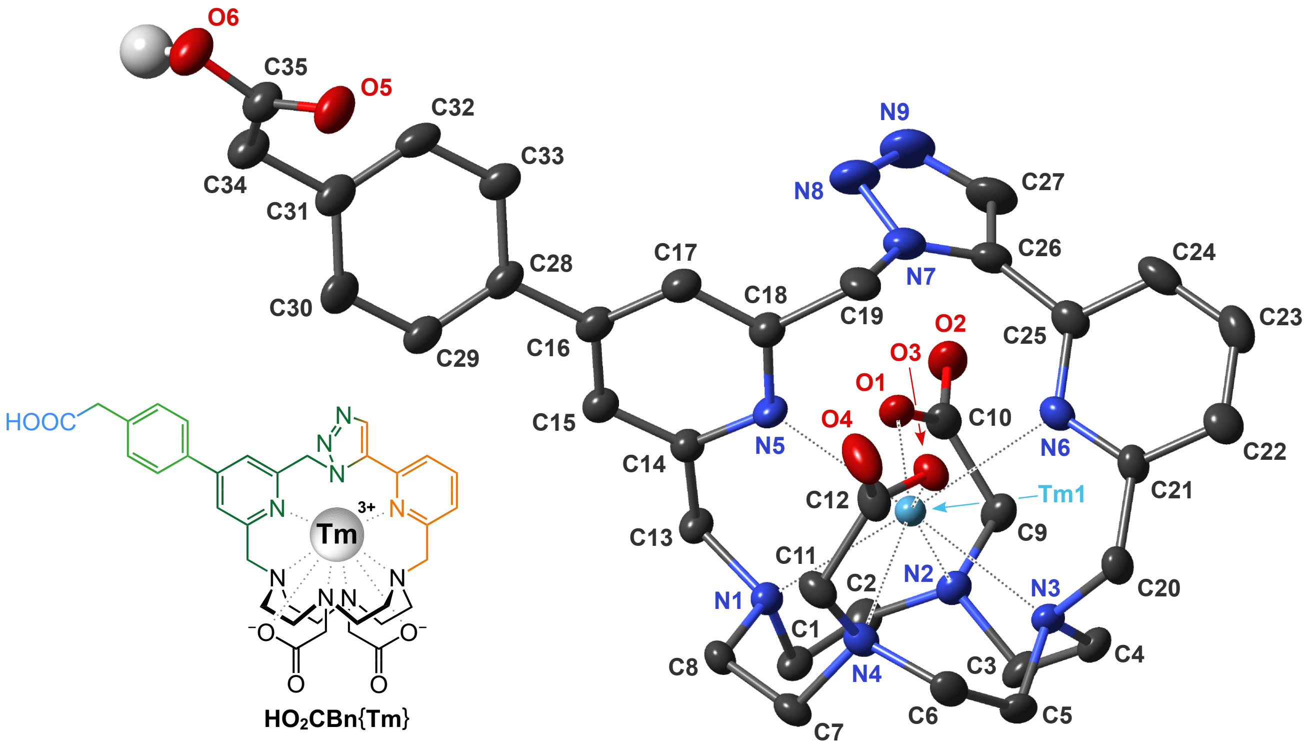

Supplementary Fig. 139. Solid-state structure of HO₂CBn{Tm} in the solid state. Structure of [**HO₂CBn**{**Tm**}]$^{+}$ cation (the distant carboxylic group is protonated and does not compensate Tm^{III} ion charge) found in the crystal structure of [**HO₂CBn**{**Tm**}]$^{+}$[ClO_4]$^{-}$·5H_2O with twisted square antiprismatic (TSA) conformation of the chelate. The unit cell contains eight symmetrically dependent units with opposite chirality (enantiomers Λλλλλ and Δδδδδ) of which only the latter is shown. The perchlorate anion, water molecules as well as carbon-bound hydrogen atoms were omitted for clarity reasons. Thermal ellipsoids were set at 50% probability.

compound	summary formula	M_w	system	space group	*a*, Å	*b*, Å	*c*, Å	*α*, °	*β*, °	*γ*, °	*V*, Å^3
***1,5-cz*-PhL[1]**	$[H_2\mathbf{M}]^{2+}[ClO_4]_2^-\cdot 7H_2O$	952.8	triclinic	$P\bar{1}$	11.810	13.382	15.389	84.551	68.134	75.206	2182.4
***1,4-cz*-PhL[1]**	$[H_2\mathbf{M}]^{2+}[ClO_4]_2^-$	826.7	monoclinic	$P2_1/n$	10.739	26.141	13.032	90	96.702	90	3633.2
**{Lu}**	$[\mathbf{M}]^+[ClO_4]^-\cdot 5H_2O$	911.1	monoclinic	$P2_1/c$	13.878	10.196	24.410	90	97.810	90	3422.1
**Ph{Y}**	$[\mathbf{M}]^+[ClO_4]^-\cdot 4H_2O$	884.1	triclinic	$P\bar{1}$	9.994	10.511	18.218	86.843	88.458	72.565	1822.9
**Ph{Lu}**	$[\mathbf{M}]^+[ClO_4]^-\cdot 7H_2O$	1024.2	triclinic	$P\bar{1}$	9.885	10.767	20.195	80.945	83.146	72.608	2019.7
**Ph{Yb}**	$[\mathbf{M}]^+[ClO_4]^-\cdot 7H_2O$	1022.3	triclinic	$P\bar{1}$	9.898	10.741	20.187	80.972	83.055	72.537	2015.7
**Ph{Tm}**	$[\mathbf{M}]^+[ClO_4]^-\cdot 4H_2O$	964.2	triclinic	$P\bar{1}$	9.975	10.504	18.205	86.873	88.474	72.611	1817.5
**Ph{Er}**	$[\mathbf{M}]^+[ClO_4]^-\cdot 4H_2O$	962.5	triclinic	$P\bar{1}$	9.970	10.574	18.181	86.642	88.353	72.129	1820.9
**Ph{Ho}**	$[\mathbf{M}]^+[ClO_4]^-\cdot 7H_2O$	1014.2	triclinic	$P\bar{1}$	9.919	10.775	20.165	81.034	83.102	72.457	2023.6
**Ph{Dy}**	$[\mathbf{M}]^+[ClO_4]^-\cdot 7H_2O$	1011.8	triclinic	$P\bar{1}$	9.919	10.794	20.182	80.964	83.051	72.454	2028.4
**Ph{Tb}**	$[\mathbf{M}]^+[ClO_4]^-\cdot 4H_2O$	954.2	triclinic	$P\bar{1}$	10.023	10.528	18.218	86.693	88.413	72.534	1830.5
**Ph{Gd}**	$[\mathbf{M}]^+[ClO_4]^-\cdot 7H_2O$	1006.5	triclinic	$P\bar{1}$	9.970	10.775	20.214	80.855	83.071	72.648	2040.1
**Ph{Eu}**	$[\mathbf{M}]^+[ClO_4]^-\cdot 4H_2O$	947.2	triclinic	$P\bar{1}$	9.995	10.481	18.191	86.693	88.139	72.592	1815.0
**Ph{Sm}**	$[\mathbf{M}]^+[ClO_4]^-$	873.5	monoclinic	$P2_1/c$	9.396	20.480	17.346	90	94.484	90	3327.3
**Ph{Pr}**	$[\mathbf{M}(H_2O)]^+[ClO_4]^-\cdot 6H_2O$	990.2	orthorhombic	$Pbca$	10.763	19.393	38.683	90	90	90	8073.9
**Ph{Ca}**	$[\mathbf{M}]\cdot 7H_2O$	789.9	monoclinic	$P2_1/n$	10.690	13.030	27.102	90	92.931	90	3770.0
***1,4-cz*-[Lu(PhL[1])]**	$[\mathbf{M}(H_2O)]^+[ClO_4]^-\cdot 2H_2O\cdot$*i*-PrOH	1012.3	monoclinic	$P2_1/c$	22.850	16.010	24.216	90	112.789	90	8167.5
**Cl{Lu}**	$[\mathbf{M}]^+[ClO_4]^-\cdot 2H_2O$	895.5	monoclinic	$P2_1/n$	9.351	37.130	9.693	90	95.430	90	3351.5
**HO_2CPh{Lu}**	$[\mathbf{M}]^+[ClO_4]^-$	942.1	monoclinic	$P2_1/c$	9.151	21.155	17.355	90	90.841	90	3359.3
**HO_2CBn{Tm}**	$[\mathbf{M}]^+[ClO_4]^-\cdot 6H_2O$	1042.2	monoclinic	$C2/c$	20.712	17.801	25.391	90	113.948	90	8555.9

compound	chirality	*Z*	D_{calc}, g × cm^{-3}	*F*(000)	*μ*, mm^{-1}	$θ_{min}$, °	$θ_{max}$, °	*GOF*	R_1 [$I ≥ 2σ(I)$]	*T*, K	CCDC	radiation
***1,5-cz*-PhL[1]**	n/d	2	1.450	1004	2.09	3.09	65.08	0.94	6.40%	200	**2334554**	Cu/$K_α$[1]
***1,4-cz*-PhL[1]**	n/d	4	1.511	1728	2.28	3.38	67.05	0.90	6.38%	180	**2334555**	Cu/$K_α$[1]
**{Lu}**	Δδδδδ/Λλλλλ (TSA)	4	1.768	1836	3.04	2.49	28.28	1.03	2.10%	100	**2334547**	Mo/$K_α$[2]
**Ph{Y}**	Δδδδδ/Λλλλλ (TSA)	2	1.611	916	3.59	2.43	65.07	0.84	6.34%	180	**2334558**	Cu/$K_α$[1]
**Ph{Lu}**	Δδδδδ/Λλλλλ (TSA)	2	1.684	1040	5.98	2.22	66.59	1.03	3.78%	200	**2334561**	Cu/$K_α$[1]
**Ph{Yb}**	Δδδδδ/Λλλλλ (TSA)	2	1.684	1038	5.59	2.22	66.59	1.06	7.57%	180	**2334552**	Cu/$K_α$[1]
**Ph{Tm}**	Δδδδδ/Λλλλλ (TSA)	2	1.762	976	5.91	2.43	66.59	0.98	8.67%	180	**2334562**	Cu/$K_α$[1]
**Ph{Er}**	Δδδδδ/Λλλλλ (TSA)	2	1.755	974	5.62	2.43	66.59	0.86	3.96%	180	**2334556**	Cu/$K_α$[1]
**Ph{Ho}**	Δδδδδ/Λλλλλ (TSA)	2	1.664	1032	4.95	2.23	65.08	0.97	3.83%	180	**2334559**	Cu/$K_α$[1]
**Ph{Dy}**	Δδδδδ/Λλλλλ (TSA)	2	1.656	1030	11.15	2.22	67.07	1.04	3.05%	180	**2334560**	Cu/$K_α$[1]
**Ph{Tb}**	Δδδδδ/Λλλλλ (TSA)	2	1.731	968	10.83	2.43	67.07	1.05	8.71%	180	**2334553**	Cu/$K_α$[1]
**Ph{Gd}**	Δδδδδ/Λλλλλ (TSA)	2	1.638	1026	11.80	2.22	65.08	0.96	6.47%	180	**2334563**	Cu/$K_α$[1]
**Ph{Eu}**	Δδδδδ/Λλλλλ (TSA)	2	1.733	964	1.88	2.38	25.03	1.08	4.54%	100	**2334544**	Mo/$K_α$[2]
**Ph{Sm}**	Δδδδδ/Λλλλλ (TSA)	4	1.744	1764	1.91	2.56	30.49	1.05	2.15%	100	**2334546**	Mo/$K_α$[2]
**Ph{Pr}**	Δδδδδ/Λλλλλ (TSA)	8	1.629	4064	10.56	4.56	66.60	1.08	3.68%	100	**2334550**	Cu/$K_α$[2]
**Ph{Ca}**	Δδδδδ/Λλλλλ (TSA)	4	1.392	1680	2.04	3.27	68.24	0.96	7.94%	180	**2334557**	Cu/$K_α$[1]
***1,4-cz*-[Lu(PhL[1])]**	Δδδδδ/Λλλλλ (TSA)	8	1.646	4112	5.85	3.40	65.09	1.04	3.52%	100	**2334545**	Cu/$K_α$[2]
**Cl{Lu}**	Δδδδδ/Λλλλλ (TSA)	4	1.769	1784	7.70	4.90	68.24	1.09	6.54%	100	**2334548**	Cu/$K_α$[2]
**HO_2CPh{Lu}**	Δδδδδ/Λλλλλ (TSA)	4	1.863	1888	7.02	3.29	76.92	1.09	2.54%	100	**2334549**	Cu/$K_α$[2]
**HO_2CBn{Tm}**	Δδδδδ/Λλλλλ (TSA)	8	1.559	4064	5.07	3.41	66.60	1.07	5.26%	100	**2334551**	Cu/$K_α$[2]

[1] Data were collected on D8 VENTURE diffractometer (*Bruker*) equipped with a Photon 100 CMOS detector, a multilayer monochromator, and a Cu (Cu/Kα radiation; λ = 1.54178 Å) Incoatec micro-focus sealed tube. The data reduction and absorption correction were performed with Apex3 software (SAINT. Bruker AXS Inc., Madison, Wisconsin, USA, 2015). The positional and anisotropic thermal parameters of non-hydrogen atoms were refined. All hydrogen atoms were located in a difference Fourier map, but those attached to carbon atoms were repositioned geometrically. They were initially refined with soft restraints on the bond lengths and angles to regularize their geometry, then their positions were refined with riding constraints.

[2] Data were collected on XtaLAB Synergy S diffractometer (*Rigaku*) equipped with Mo (Mo/$K_α$ radiation; *λ* = 0.71073 Å) and Cu (Cu/$K_α$ radiation; *λ* = 1.54184 Å) micro-focus X-ray source and Hybrid Pixel Array Detector (HyPix-6000HE). Cryostream 800 (*Oxford Cryosystems*) cooling device was used for data collection. CrysAlis Pro software was used for data collection and cell refinement, data reduction and absorption correction (CrysAlisPRO, Version 1.0.43, Oxford Diffraction/Agilent Technologies UK Ltd, Yarnton, England, 2020.). Data were corrected for absorption effects using empirical absorption correction (spherical harmonics), implemented in SCALE3 ABSPACK scaling algorithm and numerical absorption correction based on gaussian integration over a multifaceted crystal model.

Supplementary Fig. 140. Selected crystalographic parameters. Diffraction analysis was performed on two instruments (marked 1 and 2 in blue color). The table summarizes selected crystallographic parameters for all compounds with additional information on measurement and data processing. All substances crystallized in centrosymmetric space groups. Each of the metal-containing compound consists of a pair(s) of enantiomers with twisted square antiprismatic (TSA) conformation of the chelate (absolute configuration Λλλλλ and Δδδδδ). The absence of metal ion in empty cages ***1,5-cz*-PhL[1]** and ***1,4-cz*-PhL[1]** precludes assignement of absolute chirality. Crystallographic data for structural analysis have been deposited with the Cambridge Crystallographic Data Centre, (CCDCs are in included in the table above). Copies of this information may be obtained free of charge from The Director, CCDC, 12 Union Road, Cambridge CB2 1EY, UK (fax: +44-1223-336033; e-mail: deposit@ccdc.cam.ac.uk or www: http://www.ccdc.cam.ac.uk).

Supplementary References

[1] Sheldrick, G.M. SHELXT - Integrated space-group and crystal structure determination. *Acta Cryst.* A **71**, 3–8 (2015).

[2] Sheldrick, G.M. Crystal structure refinment with SHELXL. *Acta Cryst.* C **71**, 3–8 (2015).

[3] Dolomanov, O. V., Bourhis, L. J., Gildea, R. J., Howard, J. a. K. & Puschmann, H. OLEX2: a complete structure solution, refinement and analysis program. *J. Appl. Cryst.* **42**, 339–341 (2009).

[4] Betteridge, P. W., Carruthers, J. R., Cooper, R. I., Prout, K. & Watkin, D. J. CRYSTALS version 12: software for guided crystal structure analysis. *J. Appl. Cryst.* **36**, 1487–1487 (2003).

[5] Altomare, A. et al. SIR92 – a program for automatic solution of crystal structures by direct methods. *J. Appl. Cryst.* **27**, 435–435 (1994).

[6] Palatinus, L. & Chapuis, G. SUPERFLIP – a computer program for the solution of crystal structures by charge flipping in arbitrary dimensions. *J. Appl. Cryst.* **40**, 786–790 (2007).

[7] Perdew, J. P., Ernzerhof, M. & Burke, K. Rationale for mixing exact exchange with density functional approximations. *J. Chem. Phys.* **105**, 9982–9985 (1996).

[8] Weigend, F. & Ahlrichs, R. Balanced basis sets of split valence, triple zeta valence and quadruple zeta valence quality for H to Rn: Design and assessment of accuracy. *Phys. Chem. Chem. Phys.* **7**, 3297–3305 (2005).

[9] Dolg, M., Stoll, H., Savin, A. & Preuss, H. Energy-adjusted pseudopotentials for the rare earth elements. *Theor. Chim. Acta* **75**, 173–194 (1989).

[10] Dolg, M., Stoll, H. & Preuss, H. A combination of quasirelativistic pseudopotential and ligand field calculations for lanthanoid compounds. *Theor. Chim. Acta* **85**, 441–450 (1993).

[11] Klamt, A. & Schüürmann, G. COSMO: a new approach to dielectric screening in solvents with explicit expressions for the screening energy and its gradient. *J. Chem. Soc., Perkin Trans.* **2**, 799–805 (1993).

[12] Grimme, S., Antony, J., Ehrlich, S. & Krieg, H. A consistent and accurate ab initio parametrization of density functional dispersion correction (DFT-D) for the 94 elements H-Pu. *J. Chem. Phys.* **132**, 154104 (2010).

[13] Dai, L. *et al.* Chiral DOTA chelators as an improved platform for biomedical imaging and therapy applications. *Nat. Commun.* **9**, 857 (2018).

[14] Rodríguez-Rodríguez, A. *et al.* Lanthanide(III) Complexes with a Reinforced Cyclam Ligand Show Unprecedented Kinetic Inertness. *J. Am. Chem. Soc.* **136**, 17954–17957 (2014).

[15] Castro, G. *et al.* Exceptionally Inert Lanthanide(III) PARACEST MRI Contrast Agents Based on an 18-Membered Macrocyclic Platform. *Chem. Eur. J.* **21**, 18662–18670 (2015).
