# Supplementary material for: Ultra-inert lanthanide chelates as mass tags for multiplexed bioanalysis
Source: Nat Commun. 2024 Nov 13;15:9836. doi: 10.1038/s41467-024-53867-1 (PMC11561307; doi:10.1038/s41467-024-53867-1)
Supplement: Supplementary file 2 — Description of Additional Supplementary Files [file 41467_2024_53867_MOESM2_ESM.pdf]

Description of Additional Supplementary Files

File name: Supplementary Data 1
Description: Validation data for analytical LC-MS/MS and ICP-MS methods.

File name: Supplementary Data 2
Description: Source data used in plots and charts. This dataset includes individual data points and calculations of means and standard deviations, where applicable.

File name: Supplementary Data 3
Description: Atomic coordinates of optimized structures calculated using density functional theory (DFT).
