# Supplementary material for: Ultra-inert lanthanide chelates as mass tags for multiplexed bioanalysis
Source: Nat Commun. 2024 Nov 13;15:9836. doi: 10.1038/s41467-024-53867-1 (PMC11561307; doi:10.1038/s41467-024-53867-1)
Supplement: Supplementary file 6 — Reporting Summary [file 41467_2024_53867_MOESM6_ESM.pdf]

nature portfolio

Corresponding author(s): Miloslav Polasek

Last updated by author(s): Sep 30, 2024

Reporting Summary

Nature Portfolio wishes to improve the reproducibility of the work that we publish. This form provides structure for consistency and transparency in reporting. For further information on Nature Portfolio policies, see our Editorial Policies and the Editorial Policy Checklist.

Statistics

For all statistical analyses, confirm that the following items are present in the figure legend, table legend, main text, or Methods section.

n/a	Confirmed	
☐	☒	The exact sample size (*n*) for each experimental group/condition, given as a discrete number and unit of measurement
☐	☒	A statement on whether measurements were taken from distinct samples or whether the same sample was measured repeatedly
☐	☒	The statistical test(s) used AND whether they are one- or two-sided<br>*Only common tests should be described solely by name; describe more complex techniques in the Methods section.*
☒	☐	A description of all covariates tested
☒	☐	A description of any assumptions or corrections, such as tests of normality and adjustment for multiple comparisons
☐	☒	A full description of the statistical parameters including central tendency (e.g. means) or other basic estimates (e.g. regression coefficient) AND variation (e.g. standard deviation) or associated estimates of uncertainty (e.g. confidence intervals)
☐	☒	For null hypothesis testing, the test statistic (e.g. *F*, *t*, *r*) with confidence intervals, effect sizes, degrees of freedom and *P* value noted<br>*Give P values as exact values whenever suitable.*
☒	☐	For Bayesian analysis, information on the choice of priors and Markov chain Monte Carlo settings
☒	☐	For hierarchical and complex designs, identification of the appropriate level for tests and full reporting of outcomes
☒	☐	Estimates of effect sizes (e.g. Cohen's *d*, Pearson's *r*), indicating how they were calculated

Our web collection on statistics for biologists contains articles on many of the points above.

Software and code

Policy information about availability of computer code

Data collection	No software was used
Data analysis	ANOVA test was performed in GraphPad Prism, version 8.4.3.

For manuscripts utilizing custom algorithms or software that are central to the research but not yet described in published literature, software must be made available to editors and reviewers. We strongly encourage code deposition in a community repository (e.g. GitHub). See the Nature Portfolio guidelines for submitting code & software for further information.

Data

Policy information about availability of data

All manuscripts must include a data availability statement. This statement should provide the following information, where applicable:

- Accession codes, unique identifiers, or web links for publicly available datasets
- A description of any restrictions on data availability
- For clinical datasets or third party data, please ensure that the statement adheres to our policy

The crystallographic data for the structures reported in this study have been deposited at the Cambridge Crystallographic Data Centre (CCDC), under the deposition numbers 2334554 (1,5-cz-PhL1), 2334555 (1,4-cz-PhL1), 2334547 ({Lu}), 2334558 (Ph{Y}), 2334561 (Ph{Lu}), 2334552 (Ph{Yb}), 2334562 (Ph{Tm}), 2334556 (Ph{Er}), 2334559 (Ph{Ho}), 2334560 (Ph{Dy}), 2334553 (Ph{Tb}), 2334563 (Ph{Gd}), 2334544 (Ph{Eu}), 2334546 (Ph{Sm}), 2334550 (Ph{Pr}), 2334557 (Ph{Ca}), 2334545 (1,4-cz-[Lu(PhL1)]), 2334548 (Cl{Lu}), 2334549 (HO2CPh{Lu}) and 2334551 (HO2CBn{Tm}). These data can be obtained free of charge from the Centre via its website

(www.ccdc.cam.ac.uk/getstructures). The validation data for analytical methods are provided in the Supplementary Dataset 1. All data used to create plots and charts are provided in numerical form in the Supplementary Dataset 2. Atomic coordinates of the optimized computational models are provided in Supplementary Dataset 3. Additional details for methods, synthesis and characterization of new compounds, and X-ray structures are provided in Supplementary Information. All other data supporting the findings in this study are available within the article and Supplementary Information.

Research involving human participants, their data, or biological material

Policy information about studies with human participants or human data. See also policy information about sex, gender (identity/presentation), and sexual orientation and race, ethnicity and racism.

Reporting on sex and gender	*Use the terms sex (biological attribute) and gender (shaped by social and cultural circumstances) carefully in order to avoid confusing both terms. Indicate if findings apply to only one sex or gender; describe whether sex and gender were considered in study design; whether sex and/or gender was determined based on self-reporting or assigned and methods used.<br>Provide in the source data disaggregated sex and gender data, where this information has been collected, and if consent has been obtained for sharing of individual-level data; provide overall numbers in this Reporting Summary. Please state if this information has not been collected.<br>Report sex- and gender-based analyses where performed, justify reasons for lack of sex- and gender-based analysis.*
Reporting on race, ethnicity, or other socially relevant groupings	*Please specify the socially constructed or socially relevant categorization variable(s) used in your manuscript and explain why they were used. Please note that such variables should not be used as proxies for other socially constructed/relevant variables (for example, race or ethnicity should not be used as a proxy for socioeconomic status).<br>Provide clear definitions of the relevant terms used, how they were provided (by the participants/respondents, the researchers, or third parties), and the method(s) used to classify people into the different categories (e.g. self-report, census or administrative data, social media data, etc.)<br>Please provide details about how you controlled for confounding variables in your analyses.*
Population characteristics	*Describe the covariate-relevant population characteristics of the human research participants (e.g. age, genotypic information, past and current diagnosis and treatment categories). If you filled out the behavioural & social sciences study design questions and have nothing to add here, write "See above."*
Recruitment	*Describe how participants were recruited. Outline any potential self-selection bias or other biases that may be present and how these are likely to impact results.*
Ethics oversight	*Identify the organization(s) that approved the study protocol.*

Note that full information on the approval of the study protocol must also be provided in the manuscript.

Field-specific reporting

Please select the one below that is the best fit for your research. If you are not sure, read the appropriate sections before making your selection.

☒ Life sciences ☐ Behavioural & social sciences ☐ Ecological, evolutionary & environmental sciences

For a reference copy of the document with all sections, see nature.com/documents/nr-reporting-summary-flat.pdf

Life sciences study design

All studies must disclose on these points even when the disclosure is negative.

Sample size	In this study, the primary objective of our animal experiments was to validate analytical methods rather than to investigate biological effects. Consequently, we opted for minimal sample sizes to meet our research goals without unnecessary animal use. The sample sizes were not determined through statistical methods but were instead chosen based on over 30 years of field experience by our scientists. This approach ensured a balance between ethical considerations—minimizing animal use and suffering—and the need for sufficient data to confirm the accuracy and reproducibility of our methods. Specific sample sizes used are detailed in the Methods section and corresponding figure captions, with N = 3 for mice and N = 4 for rats.
Data exclusions	No data were excluded.
Replication	The primary aim of our animal experiments was to validate analytical methods, not to study biological effects. We achieved good reproducibility with our analytical measurements, as demonstrated in Figs. 8 – 9 and detailed through individual data points in Supplementary Dataset 2. The methods themselves underwent thorough validation, as outlined in Supplementary Figs. 29 – 30 and Supplementary Dataset 1. Given these results, we determined that additional verification involving more animals was both unnecessary and unethical, considering our objectives were met with the current setup.
Randomization	Randomization was not relevant, as the performed experiments did not involve comparison of groups.
Blinding	Blinding was not relevant, as the performed experiments did not involve comparison of groups.

Reporting for specific materials, systems and methods

We require information from authors about some types of materials, experimental systems and methods used in many studies. Here, indicate whether each material, system or method listed is relevant to your study. If you are not sure if a list item applies to your research, read the appropriate section before selecting a response.

Materials & experimental systems

n/a	Involved in the study
☒	☐ Antibodies
☐	☒ Eukaryotic cell lines
☒	☐ Palaeontology and archaeology
☐	☒ Animals and other organisms
☒	☐ Clinical data
☒	☐ Dual use research of concern
☒	☐ Plants

Methods

n/a	Involved in the study
☒	☐ ChIP-seq
☒	☐ Flow cytometry
☒	☐ MRI-based neuroimaging

Eukaryotic cell lines

Policy information about cell lines and Sex and Gender in Research

Cell line source(s)	CCRF-CEM cells (CCL-119TM) purchased from ATCC (Manassas, VA, USA) are human T lymphoblasts isolated from the peripheral blood of a 4-year-old Caucasian female with acute lymphoblastic leukemia (ALL).
Authentication	The cell line was authenticated by the manufacturer.
Mycoplasma contamination	Cross-contamination in the laboratory was prevented by renewing of the cells from early passage cryovials every 3 months. The cells were negatively tested for Mycoplasma (MycoAlertTM Mycoplasma detection kit, Lonza).
Commonly misidentified lines (See ICLAC register)	According to ICLAC register, this is not relevant for the CCRF-CEM cells.

Animals and other research organisms

Policy information about studies involving animals; ARRIVE guidelines recommended for reporting animal research, and Sex and Gender in Research

Laboratory animals	Male C57BL/6J mice at age of 6 months and male Wistar rats at age of 3 months were used. All animals were obtained from Charles River (Sulzfeld, Germany).
Wild animals	No wild animals were involved in the study.
Reporting on sex	Sex is reported but not relevant for the study.
Field-collected samples	No field-collected samples were used in the study.
Ethics oversight	The animal experiments followed the ethical guidelines for animal experiments in the Czech Republic Act Nr. 246/1992 and were approved by the Committee for Experiments with Laboratory Animals of Czech Academy of Sciences under the protocol number 80/2020 (experiment name: Relationship between obesity, diabetes and neurodegeneration: new therapeutic potential of prolactin releasing peptide analogues).

Note that full information on the approval of the study protocol must also be provided in the manuscript.

Plants

Seed stocks	*Report on the source of all seed stocks or other plant material used. If applicable, state the seed stock centre and catalogue number. If plant specimens were collected from the field, describe the collection location, date and sampling procedures.*
Novel plant genotypes	*Describe the methods by which all novel plant genotypes were produced. This includes those generated by transgenic approaches, gene editing, chemical/radiation-based mutagenesis and hybridization. For transgenic lines, describe the transformation method, the number of independent lines analyzed and the generation upon which experiments were performed. For gene-edited lines, describe the editor used, the endogenous sequence targeted for editing, the targeting guide RNA sequence (if applicable) and how the editor was applied.*
Authentication	*Describe any authentication procedures for each seed stock used or novel genotype generated. Describe any experiments used to assess the effect of a mutation and, where applicable, how potential secondary effects (e.g. second site T-DNA insertions, mosiacism, off-target gene editing) were examined.*
